# Supplementary material for: A versatile Diels–Alder approach to functionalized hydroanthraquinones
Source: R Soc Open Sci. 2020 Nov 18;7(11):200626. doi: 10.1098/rsos.200626 (PMC7735338; doi:10.1098/rsos.200626)
Supplement: Supporting Information [file rsos200626supp1.docx]

Supporting Information

A versatile Diels-Alder Approach to Functionalized Hydroanthraquinones

Janina Beck,^a^ Olaf Fuhr, ^b^ Martin Nieger ^c^ and Stefan Bräse ^*ad^

^a^ Institute of Organic Chemistry, Karlsruhe Institute of Technology (KIT), Fritz-Haber-Weg 6, 76131 Karlsruhe, Germany. E-mail: braese@kit.edu; Fax: (+49)-721-6084-8581; Tel: (+49)-721-6084-2903.

^b^ Institute of Nanotechnology (INT) and Karlsruhe Nano-Micro Facility (KNMF), Karlsruhe Institute of Technology (KIT), Hermann-von-Helmholtz-Platz 1, 76344 Eggenstein-Leopoldshafen, Germany

^c^ Department of Chemistry, University of Helsinki, P. O. Box 55 (A. I. Virtasen aukio 1), 00014 Helsinki, Finland

^d^ Institute of Biological and Chemical Systems - Functional Molecular Systems (IBCS-FMS), Karlsruhe Institute of Technology (KIT), Hermann-von-Helmholtz Platz 1, 76344 Eggenstein-Leopoldshafen, Germany

Table of contents for Supporting Information

[1. General Information 2](#_Toc36705310)

[2. Synthetic Procedures and Compound Characterization 4](#_Toc36705311)

[3. ^1^H and ^13^C NMR Spectra of the Products 24](#_Toc36705312)

[4. Crystallographic Information 68](#_Toc36705313)

## General Information

### Preparative Work

Reactions carried out under argon atmosphere were conducted using previously flame-dried glassware with standard Schlenk techniques. Liquid reagents and solvents were injected with plastic syringes and stainless-steel cannulas. Routine monitoring of reactions was performed using Silica gel-coated aluminium plates (Merck, silica gel 60, F_254_), which were analyzed under UV light at 254 nm and/or dipped into a solution of phosphomolybdic acid stain (2.5% phosphor molybdic acid, 1.0% Cerium(IV)sulfate tetrahydrate and 6.0% sulfuric acid in H_2_O, dipping solution). Purification by flash chromatography was performed using silica gel, produced by Merck (silica gel 60, 0.040 × 0.063 mm, 260 – 400 mesh ASTM). Solvents for chromatography were commercially acquired in HPLC-grade and mixed after measuring amounts volumetrically. Solvents were removed at 40 °C at the rotavapor.

### Solvents and Chemicals

Solvents of technical quality have been purified by distillation or with the solvent purification system MB SPS5 from MBRAUN before use. Solvents of p.a. quality (*per analysis*) were bought from Sigma Aldrich, Carl Roth or Acros Fisher Scientific and used without previous purification unless otherwise stated. Absolute solvents were either purchased from Carl Roth, Acros or Sigma Aldrich (< 50 ppm H_2_O over molecular sieve). All reagents were commercially acquired (through ABCR, Acros, Alfa Aesar or Sigma Aldrich) and used without further purification.

### Nuclear Magnetic Resonance (NMR) Spectroscopy

^1^H NMR spectra were recorded on a Bruker Avance AV 300 (300 MHz), Bruker Avance 400 (400 MHz) or a Bruker Avance DRX 500 (500 MHz) as solutions at room temperature. Chemical shifts are expressed in parts per million (ppm, δ) downfield from tetramethylsilane (TMS) and are referenced to CHCl_3_ (7.26 ppm) as an internal standard. All coupling constants are absolute values and *J* values are expressed in Hertz (Hz). For the characterization of centrosymmetrical signals, the signal’s median point was chosen, for multiplets the signal range. The description of signals includes: s = singlet, d = doublet, t = triplet, q= quartet, m = multiplet, dd = doublet of doublet, ddd = doublet of dd, dt = doublet of triplet, td = triplet of doublet, bs = broad singlet, m= multiplet. The spectra were analyzed according to first order.

^13^C NMR spectra were recorded on a Bruker DRX 500 (126 MHz) spectrometer. Chemical shifts are expressed in parts per million (ppm, δ) downfield from tetramethylsilane (TMS) and are referenced to CDCl_3_ (77.2 ppm) as an internal standard. The signals for ^1^H were assigned by the multiplets and for ^13^C by DEPT 90- and DEPT 135-spectra (DEPT = Distortionless Enhancement by Polarization Transfer) as well as by obvious chemical shifts. The multiplicity of ^13^C NMR-signals is given as follows: DEPT: + = primary or tertiary (positive DEPT-signal), –= secondary (negative DEPT-signal), C_q_ = quarternary C‑atoms, no DEPT-signal). The spectra were analyzed according to first order.

### Mass Spectrometry (MS)

The measurements for analytical data were performed on a Finnigan MAT 95 instrument using the fast atom bombardment (FAB) method, where 3-nitrobenzyl alcohol (3-NBA) was used as matrix. For the high-resolution mass, the following abbreviations were used: calc. = theoretical calculated mass; found = mass found in the analysis. APCI and ESI experiments were recorded on a Q-Exactive (Orbitrap) mass spectrometer (Thermo Fisher Scientific, San Jose, CA, USA) equipped with a HESI II probe to record high resolution. The tolerated error is 5 ppm of the molecular mass. For the interpretation of the spectra, molecular peaks [M]^+^, peaks of pseudo molecules [M+H]^+^ and characteristic fragment peaks are indicated with their mass to charge ratio (m/z) and their intensity in per cent, relative to the base peak (100%).

### Infrared Spectroscopy (IR)

Infrared spectra were recorded with an ALPHA-T instrument made by Bruker. Solids were measured by attenuated total reflection (Diamond ATR method). Absorption is given in wavenumbers ν [cm^-1^] and was measured in the range from 3600 cm^-1^ to 500 cm^-1^.

### Data availability

The obtained data were deposited in the repository Chemotion (reaction details and compound characterization, <https://www.chemotion-repository.net/home/publications>) and the CCDC (crystal structures, <https://www.ccdc.cam.ac.uk/structures/>). The related DOIs which link to the Chemotion repository submissions and numbers referring to the CCDC entries are added at the end of the characterization section for the individual compounds.

## Synthetic Procedures and Compound Characterization

**(1,4-Dimethoxynaphthalen-2-yl)(2-iodophenyl)methanone (4a)[[1]](#footnote-1)**

According to **GP A** a mixture of trifluoroacetic anhydride (11.2 mL, 16.7 g, 79.5 mmol, 7.10 equiv.), 1,4‑dimethoxynaphthalene (**2**) (2.11 g, 11.2 mmol, 1.00 equiv.) and 2-iodobenzoic acid (**3a**) (2.78 g, 11.2 mmol, 1.00 equiv.) was used. The crude product was purified *via* flash chromatography on silica gel on silica gel (cHex/EtOAc = 4:1). The product **4a** was obtained as a yellow solid (3.14 g, 7.51 mmol, 67%). – *R*_f_ (cHex/EtOAc = 4:1) = 0.30. – ^1^H NMR (500 MHz, CDCl_3_): *δ* = 8.29 (dd, ^3^*J* = 8.3 Hz, ^4^*J* = 1.3 Hz, 1H, C*H*_Ar_), 8.09 (dd, ^3^*J* = 8.3 Hz, ^4^*J* = 1.3 Hz, 1H, C*H*_Ar_), 7.97 (d, ^3^*J* = 8.0 Hz, 1H, C*H*_Ar_), 7.63–7.60 (m, 1H, C*H*_Ar_), 7.59−7.56 (m, 1H, C*H*_Ar_), 7.44–7.38 (m, 2H, C*H*_Ar_), 7.19–7.16 (m, 1H, C*H*_Ar_), 7.11 (s, 1H, C*H*_Ar_), 4.04 (s, 3H, OC*H*_3_), 3.63 (s, 3H, OC*H*_3_) ppm. – ^13^C NMR (126 MHz, CDCl_3_): *δ* = 196.9 (C_q_, 1 × *C*=O), 152.3 (C_q_, 1 × *C*_qAr_), 152.1 (C_q_, 2 × *C*_qAr_), 145.5 (C_q_, 1 × *C*_qAr_), 140.2 (+, 1 × *C*H_Ar_), 131.6 (+, 1 × *C*H_Ar_), 129.6 (+, 1 × *C*H_Ar_), 128.8 (C_q_, 1 × *C*_qAr_), 128.3 (+, 1 × *C*H_Ar_), 127.8 (+, 1 × *C*H_Ar_), 127.3 (+, 1 × *C*H_Ar_), 125.7 (C_q_, 1 × *C*_qAr_), 123.6 (+, 1 × *C*H_Ar_), 122.7 (+, 1 × *C*H_Ar_), 103.2 (+, 1 × *C*H_Ar_), 92.4 (C_q_, 1 × *C*_qAr_), 64.3 (+, 1 × O*C*H_3_), 56.0 (+, 1 × O*C*H_3_) ppm. – IR (ATR): ṽ = 3047 (vw), 2993 (w), 2930 (w), 2836 (w), 1650 (m), 1577 (m), 1460 (m), 1366 (m), 1271 (m), 1206 (m), 1091 (m), 1047 (m), 1017 (m), 999 (m), 955 (m), 858 (m), 803 (m), 783 (w), 761 (m), 746 (m), 688 (m), 665 (m), 648 (m), 634 (m), 449 (w) cm^–1^. – MS (FAB, 3-NBA), *m*/*z*(%): 418 (100) [M]^+^, 419 (95) [M+H]^+^. – HRMS (EI, C_19_H_15_^127^IO_3_): calc. 418.0066; found 418.0066. – X‑Ray: The structure of **4a** could be confirmed by single crystal X‑Ray diffraction (see 4 Crystallographic Information, CCDC 1992878).
Additional reaction details and data obtained from the characterization of the target compound can be accessed at: <https://dx.doi.org/10.14272/reaction/SA-FUHFF-UHFFFADPSC-VIFKGORYWD-UHFFFADPSC-NUHFF-NUHFF-NUHFF-ZZZ>.
Analytical data is in accordance with previously published literature.^1^

**(2-Bromo-4,5-dimethoxyphenyl)(1,4-dimethoxynaphthalen-2-yl)methanone (4d)**

According to **GP A** a mixture of trifluoroacetic anhydride (2.8 mL, 4.20 g, 20.0 mmol, 10.0 equiv.), 1,4‑dimethoxynaphthalene (**2**) (376 mg, 2.00 mmol, 1.00 equiv.) and 2-bromo-4,5-dimethoxybenzoic acid (**3d**) (2.78 g, 11.2 mmol, 1.00 equiv.) was used. The crude product was purified *via* flash chromatography on silica gel (cHex/EtOAc = 4:1). The product **4d** was obtained as a yellow solid (799 mg, 1.85 mmol, 77%). – *R*_f_ (cHex/EtOAc = 4:1) = 0.27. – ^1^H NMR (400 MHz, CDCl_3_): *δ* = 8.32–8.27 (m, 1H, C*H*_Ar_), 8.15–8.10 (m, 1H, C*H*_Ar_), 7.64–7.54 (m, 2H, C*H*_Ar_), 7.09 (s, 1H, C*H*_Ar_), 7.07 (s, 1H, C*H*_Ar_), 7.00 (s, 1H, C*H*_Ar_), 4.02 (s, 3H, OC*H*_3_), 3.95 (s, 3H, OC*H*_3_), 3.83 (s, 3H, OC*H*_3_), 3.70 (s, 3H, OC*H*_3_) ppm. – ^13^C NMR (101 MHz, CDCl_3_): *δ* = 195.3 (C_q_, 1 × *C*=O), 152.0 (C_q_, 1 × *C*_qAr_), 151.4 (C_q_, 1 × *C*_qAr_), 151.3 (C_q_, 1 × *C*_qAr_), 148.1 (C_q_, 1 × *C*_qAr_), 133.7 (C_q_, 1 × *C*_qAr_), 129.1 (C_q_, 1 × *C*_qAr_), 128.8 (C_q_, 1 × *C*_qAr_), 128.0 (+, 1 × *C*H_Ar_), 127.3 (+, 1 × *C*H_Ar_), 126.9 (C_q_, 1 × *C*_qAr_), 123.5 (+, 1 × *C*H_Ar_), 122.7 (+, 1 × *C*H_Ar_), 116.1 (+, 1 × *C*H_Ar_), 113.3 (+, 1 × *C*H_Ar_), 112.2 (C_q_, 1 × *C*_qAr_), 103.3 (+, 1 × *C*H_Ar_), 64.1 (+, 1 × O*C*H_3_), 56.4 (+, 1 × O*C*H_3_), 56.3 (+, 1 × O*C*H_3_), 56.0 (+, 1 × O*C*H_3_) ppm. – IR (ATR): ṽ = 2932 (vw), 1634 (w), 1591 (w), 1504 (w), 1439 (w), 1402 (w), 1378 (w), 1335 (w), 1254 (w), 1209 (w), 1182 (w), 1157 (w), 1121 (w), 1095 (w), 1053 (w), 1029 (w), 994 (w), 965 (w), 864 (w), 839 (w), 772 (w), 738 (w), 713 (w), 649 (vw), 626 (w), 551 (vw), 430 (vw) cm^–1^. – MS (FAB, 3‑NBA), *m*/*z* (%): 433/431 (66/68) [M+H]^+^, 432/430 (100/90) [M]^+^, 351 (12) [M–Br]^+^. – HRMS (FAB, C_21_H_19_^79^BrO_5_): calc. 430.0416; found 430.0417. – X‑Ray: The structure of **4d** could be confirmed by single crystal X‑Ray diffraction (see 4 Crystallographic Information, CCDC 1992879).
Additional reaction details and data obtained from the characterization of the target compound can be accessed at: <https://dx.doi.org/10.14272/reaction/SA-FUHFF-UHFFFADPSC-ANVMBMPZHQ-UHFFFADPSC-NUHFF-NUHFF-NUHFF-ZZZ>.

***N*-(4-Bromo-3-(1,4-dimethoxy-2-naphthoyl)phenyl)acetamide (4e)**

According to **GP A** a mixture of trifluoroacetic anhydride (2.1 mL, 3.15 g, 15.0 mmol, 10.0 equiv.), 1,4‑dimethoxynaphthalene (**2**) (376 mg, 2.00 mmol, 1.00 equiv.) and 5-acetamido-2-bromobenzoic acid (**3e**) (464 mg, 1.80 mmol, 1.20 equiv.) was used. The crude product was purified *via* flash chromatography on silica gel (cHex/EtOAc = 1:1). The product **4e** was obtained as a yellow solid (560 mg, 1.31 mmol, 65%). – *R*_f_ (cHex/EtOAc = 1:1) = 0.32. – ^1^H NMR (500 MHz, CDCl_3_): *δ* = 8.28 (dd, ^3^*J* = 8.2 Hz, ^4^*J* = 1.3 Hz, 1H, C*H*_Ar_), 8.08 (dd, ^3^*J* = 8.2 Hz, ^4^*J* = 1.3 Hz, 1H, C*H*_Ar_), 7.70 (dd, ^3^*J* = 8.7 Hz, ^4^*J* = 2.6 Hz, 1H, C*H*_Ar_), 7.63–7.55 (m, 3H, C*H*_Ar_), 7.43 (d, ^4^*J*= 2.6 Hz, 1H, C*H*_Ar_), 7.36 (brs, 1H, N*H*), 7.07 (s, 1H, C*H*_Ar_), 4.02 (s, 3H, OC*H*_3_), 3.65 (s, 3H, OC*H*_3_), 2.13 (s, 3H, C*H*_3_) ppm. – ^13^C NMR (126 MHz, CDCl_3_): *δ* = 195.2 (C_q_, 1 × *C*=O), 168.5 (C_q_, 1 × *C*=O), 152.5 (C_q_, 1 × *C*_qAr_), 152.2 (C_q_, 1 × *C*_qAr_), 142.8 (C_q_, 1 × *C*_qAr_), 137.3 (C_q_, 1 × *C*_qAr_), 134.0 (+, 1 × *C*H_Ar_), 129.7 (C_q_, 1 × *C*_qAr_), 128.8 (C_q_, 1 × *C*_qAr_), 128.4 (+, 1 × *C*H_Ar_), 127.4 (+, 1 × *C*H_Ar_), 126.0 (C_q_, 1 × *C*_qAr_), 123.6 (+, 1 × *C*H_Ar_), 122.8 (+, 1 × *C*H_Ar_), 122.7 (+, 1 × *C*H_Ar_), 120.6 (+, 1 × *C*H_Ar_), 113.9 (C_q_, 1 × *C*_qAr_), 102.8 (+, 1 × *C*H_Ar_), 64.3 (+, 1 × O*C*H_3_), 56.0 (+, 1 × O*C*H_3_), 24.7 (+, 1 × *C*H_3_) ppm. – IR (ATR): ṽ = 3306 (w), 2935 (w), 2250 (vw), 1664 (m), 1593 (m), 1531 (m), 1459 (m), 1394 (m), 1366 (s), 1309 (m), 1242 (m), 1207 (m), 1163 (w), 1117 (m), 1095 (s), 1047 (m), 1028 (m), 993 (m), 963 (w), 907 (m), 821 (w), 800 (w), 769 (m), 727 (s), 647 (m), 587 (w), 483 (w), 415 (w) cm^–1^. – MS (FAB, 3-NBA), *m*/*z*(%): 430/428 (56/55) [M+H]^+^, 429/427 (69/56) [M]^+^. – HRMS (FAB, C_21_H_18_O_4_N^79^Br): calc. 427.0419; found 427.0421. – X‑Ray: The structure of **4e** could be confirmed by single crystal X‑Ray diffraction (see 4 Crystallographic Information, CCDC 1992880).
Additional reaction details and data obtained from the characterization of the target compound can be accessed at: <https://dx.doi.org/10.14272/reaction/SA-FUHFF-UHFFFADPSC-OPAWPZCMGG-UHFFFADPSC-NUHFF-NUHFF-NUHFF-ZZZ>.

***N*-(4-Bromo-3-(1,4-dimethoxy-2-naphthoyl)phenyl)-2,2,2-trifluoroacetamide (4f)**

According to **GP A** a mixture of trifluoroacetic anhydride (1.4 mL, 2.10 g, 10.0 mmol, 10.0 equiv.), 1,4‑dimethoxynaphthalene (**2**) (188 mg, 1.00 mmol, 1.00 equiv.) and 5-amino-2-bromobenzoic acid (**3f**) (216 mg, 1.00 mmol, 1.00 equiv.) was used. The crude product was purified *via* flash chromatography on silica gel (cHex/EtOAc = 9:1). The product **4f** was obtained as a yellow solid (341 mg, 707 µmol, 71%). – *R*_f_ (cHex/EtOAc = 9:1) = 0.20. − ^1^H NMR (400 MHz, CDCl_3_): *δ* = 8.45 (brs, 1H, N*H*), 8.28 (d, ^3^*J* = 8.3 Hz, 1H, C*H*_Ar_), 8.06 (d, ^3^*J* = 8.3 Hz, 1H, C*H*_Ar_), 7.72 (dd, ^3^*J* = 8.8 Hz, ^4^*J* = 2.6 Hz, 1H, C*H*_Ar_), 7.64–7.53 (m, 4H, C*H*_Ar_), 7.07 (s, 1H, C*H*_Ar_), 4.01 (s, 3H, OC*H*_3_), 3.62 (s, 3H, OC*H*_3_) ppm. − ^13^C NMR (101 MHz, CDCl_3_): *δ* = 194.9 (C_q_, 1 × *C*=O), 155.1 (q, ^2^*J* = 38.0 Hz, 1 × *C*OCF_3_), 152.9 (C_q_, 1 × *C*_qAr_), 152.3 (C_q_, 2 × *C*_qAr_), 143.5 (C_q_, 1 × *C*_qAr_), 134.6 (C_q_, 1 × *C*_qAr_), 134.3 (+, 1 × *C*H_Ar_), 129.9 (C_q_, 1 × *C*_qAr_), 128.7 (+, 1 × *C*H_Ar_), 127.5 (+, 1 × *C*H_Ar_), 125.6 (C_q_, 1 × *C*_qAr_), 123.6 (+, 1 × *C*H_Ar_), 123.3 (+, 1 × *C*H_Ar_), 122.9 (+, 1 × *C*H_Ar_), 121.3 (+, 1 × *C*H_Ar_), 116.3 (C_q_, 1 × *C*_qAr_), 115.7 (q, ^1^*J* = 287.0 Hz, 1 × *C*F_3_), 102.4 (+, 1 × *C*H_Ar_), 64.3 (+, 1 × O*C*H_3_), 56.0 (+, 1 × O*C*H_3_) ppm. – IR (ATR): ṽ = 3067 (vw), 2944 (vw), 2843 (vw), 1723 (w), 1618 (w), 1578 (w), 1541 (w), 1458 (w), 1405 (w), 1371 (m), 1297 (w), 1225 (w), 1168 (w), 1119 (w), 1096 (w), 1045 (w), 1029 (w), 994 (w), 965 (w), 901 (vw), 877 (vw), 856 (w), 834 (w), 796 (w), 765 (w), 685 (w), 651 (w), 598 (w), 479 (w), 430 (vw), 389 (w) cm^–1^. – MS (APCI), *m*/*z* (%): 482/484 (97/100) [M+H]^+^. – HRMS (APCI, C_21_H_16_^79^BrF_3_NO_4_): calc. 482.0215; found 482.0198. − X‑Ray: The structure of **4f** could be confirmed by single crystal X‑Ray diffraction (see 4 Crystallographic Information, CCDC 1992881).
Additional reaction details and data obtained from the characterization of the target compound can be accessed at: <https://dx.doi.org/10.14272/reaction/SA-FUHFF-UHFFFADPSC-IOGOYECUJC-UHFFFADPSC-NUHFF-NUHFF-NUHFF-ZZZ>.

**2-(2-Iodobenzoyl)naphthalene-1,4-dione (5a)[[2]](#footnote-2)**

Following **GP B** the crude product was obtained from CAN (14.5 g, 26.5 mmol, 3.70 equiv.) and (1,4-dimethoxynaphthalen-2-yl)(2-iodophenyl)methanone (**4a**) (3.00 g, 7.17 mmol, 1.00 equiv.). The product **5a** was isolated as an orange solid (2.78 g, 7.17 mmol, quant.). – *R*_f_ (cHex/EtOAc = 4:1) = 0.25. – ^1^H NMR (300 MHz, CDCl_3_): *δ* = 8.14–8.10 (m, 2H, C*H*_Ar_), 7.96 (dd, ^3^*J* = 7.8 Hz, 1H, C*H*_Ar_), 7.83–7.80 (m, 2H, C*H*_Ar_), 7.57 (d, ^3^*J* = 7.8 Hz, 1H, C*H*_Ar_), 7.48 (dd, ^3^*J* = 7.8 Hz, 1H, C*H*_Ar_), 7.24 (dd, ^3^*J* = 7.8 Hz, 1H, C*H*_Ar_), 7.18 (s, 1H, C=C*H*) ppm. − ^13^C NMR (101 MHz, CDCl_3_): *δ* = 193.6 (C_q_, 1 × *C*=O), 185.0 (C_q_, 1 × *C*=O), 182.7 (C_q_, 1 × *C*=O), 145.0 (C_q_, 1 × *C*_qAr_), 142.0 (C_q_, 1 × *C*_qAr_), 140.9 (+, 1 × C=*C*H), 137.8 (+, 1 × *C*H_Ar_), 134.8 (+, 1 × *C*H_Ar_), 134.5 (+, 1 × *C*H_Ar_), 133.3 (+, 1 × *C*H_Ar_), 132.1 (C_q_, 1 × *C*_qAr_), 131.9 (C_q_, 1 × *C*_qAr_), 131.1 (+, 1 × *C*H_Ar_), 128.5 (+, 1 × *C*H_Ar_), 127.1 (+, 1 × *C*H_Ar_), 126.6 (+, 1 × *C*H_Ar_), 93.1 (C_q_, 1 × *C*_qAr_) ppm. – IR (ATR): ṽ = 3041 (vw), 1653 (m), 1579 (w), 1424 (w), 1282 (w), 1252 (w), 1014 (w), 973 (w), 943 (w), 773 (w), 748 (w), 691 (w), 673 (w), 633 (w), 591 (w), 453 (vw), 413 (vw) cm^–1^. – MS (FAB, 3‑NBA), *m*/*z*(%): 390 (62) [M+H]^+^, 389 (69) [M]^+^. – HRMS (EI, C_17_H_10_^127^IO_3_): calc. 388.9675; found 388.9677. − X‑Ray: The structure of **5a** could be confirmed by single crystal X‑Ray diffraction (see 4 Crystallographic Information, CCDC 1992180).
Additional reaction details and data obtained from the characterization of the target compound can be accessed at: <https://dx.doi.org/10.14272/reaction/SA-FUHFF-UHFFFADPSC-CIZDEMMLYC-UHFFFADPSC-NUHFF-NUHFF-NUHFF-ZZZ>.
Analytical data is in accordance with previously published literature.^2^

**2-(2-Bromo-4,5-dimethoxybenzoyl)naphthalene-1,4-dione (5d)**

Following **GP B** the crude product was obtained from CAN (620 mg, 1.12 mmol, 3.70 equiv.) and (2-bromo-4,5-dimethoxyphenyl)(1,4-dimethoxynaphthalen-2-yl)methanone (**4d**) (130 mg, 300 µmol, 1.00 equiv.). The product **5d** was isolated as an orange solid (88.0 mg, 220 µmol, 73%). – *R*_f_ (cHex/EtOAc = 4:1) = 0.22. − ^1^H NMR (400 MHz, CDCl_3_): *δ* = 8.15−8.10 (m, 2H, C*H*_Ar_), 7.83−7.79 (m, 2H, C*H*_Ar_), 7.39 (s, 1H, C*H*_Ar_), 7.10 (s, 1H, C*H*_Ar_), 7.01 (s, 1H, C=C*H*), 3.95 (s, 3H, OC*H*_3_), 3.94 (s, 3H, OC*H*_3_) ppm. − ^13^C NMR (101 MHz, CDCl_3_): *δ* = 191.2 (C_q_, 1 × *C*=O), 185.1 (C_q_, 1 × *C*=O), 183.0 (C_q_, 1 × *C*=O), 153.4 (C_q_, 1 × *C*_qAr_), 148.9 (C_q_, 1 × *C*_qAr_), 147.9 (C_q_, 1 × *C*_qAr_), 136.1 (+, 1 × *C*H_Ar_), 134.6 (+, 1 × *C*H_Ar_), 134.5 (+, 1 × *C*H_Ar_), 132.2 (C_q_, 1 × *C*_qAr_), 132.0 (C_q_, 1 × *C*_qAr_), 130.5 (C_q_, 1 × *C*_qAr_), 127.0 (+, 1 × *C*H_Ar_), 126.6 (+, 1 × *C*H_Ar_), 116.2 (+, 1 × *C*H_Ar_), 114.8 (C_q_, 1 × *C*_qAr_), 113.7 (+, 1 × *C*H_Ar_), 56.6 (+, 1 × O*C*H_3_), 56.4 (+, 1 × O*C*H_3_) ppm. − IR (ATR): ṽ = 3009 (vw), 3045 (vw), 2925 (vw), 2840 (vw), 1659 (m), 1581 (m), 1506 (m), 1435 (w), 1377 (w), 1344 (w), 1294 (w), 1255 (m), 1211 (m), 1174 (m), 1131 (w), 1012 (m), 909 (w), 882 (w), 848 (w), 783 (w), 759 (m), 719 (w), 655 (w), 631 (w), 589 (w), 563 (w), 454 (vw), 423 (vw) cm^–1^. – MS (FAB, 3‑NBA), *m*/*z* (%): 401/403 (4/7) [M+H]^+^. – HRMS (FAB, C_19_H_14_^79^BrO_5_): calc. 401.0025; found 401.0025. − X‑Ray: The structure of **5d** could be confirmed by single crystal X‑Ray diffraction (see 4 Crystallographic Information, CCDC 1992882).
Additional reaction details and data obtained from the characterization of the target compound can be accessed at: <https://dx.doi.org/10.14272/reaction/SA-FUHFF-UHFFFADPSC-LQLVWTVLQD-UHFFFADPSC-NUHFF-NUHFF-NUHFF-ZZZ>.

**2-(2-Bromo-4,5-dihydroxybenzoyl)naphthalene-1,4-dione (5e)**

BBr_3_ (7.50 mL, 7.48 mmol, 1.87 g, 10.0 equiv., 1 m in CH_2_Cl_2_) was dropwise added to a solution of 2-(2-bromo-4,5-dimethoxybenzoyl)naphthalene-1,4-dione (**5d**) (300 mg, 750 µmol, 1.00 equiv.) in 21.0 mL CH_2_Cl_2_ at –78 °C over the course of 0.5 h. The resulting solution was allowed to warm to room temperature and was stirred for 4 h. The reaction mixture was poured into ice cold H_2_O (30 mL) and extracted with CH_2_Cl_2_ (2 × 30 mL). The combined organic phases were washed with brine (35 mL), dried over Na_2_SO_4_ and the solvent was removed under reduced pressure. After flash chromatography on silica gel (cHex/EtOAc = 1:1) the product **5e** was obtained as a dark red oil (33.0 mg, 88.4 µmol, 12%). – *R*_f_ (cHex/EtOAc = 1:1) = 0.26. − ^1^H NMR (500 MHz, MeOD): *δ* = 8.15–8.08 (m, 2H, CH_Ar_), 7.88 (dd, ^3^J = 5.5 Hz, ^4^J = 2.8 Hz, 2H, CH_Ar_), 7.31 (s, 1H, C=CH), 7.03 (s, 1H, CH_Ar_), 7.01 (s, 1H, CH_Ar_) ppm. Missing signals (2H, O*H*). − ^13^C NMR (126 MHz, MeOD): *δ* = 192.0 (C_q_, 1 × *C*=O), 186.1 (C_q_, 1 × *C*=O), 184.3 (C_q_, 1 × *C*=O), 152.7 (C_q_, 1 × *C*_qAr_), 149.4 (C_q_, 1 × *C*_qAr_), 146.4 (C_q_, 1 × *C*_qAr_), 136.4 (+, 1 × *C*H_Ar_), 135.6 (+, 1 × *C*H_Ar_), 135.5 (+, 1 × *C*H_Ar_), 133.4 (C_q_, 1 × *C*_qAr_), 133.2 (C_q_, 1 × *C*_qAr_), 129.8 (C_q_, 1 × *C*_qAr_), 127.5 (+, 1 × *C*H_Ar_), 127.2 (+, 1 × *C*H_Ar_), 121.6 (+, 1 × *C*H_Ar_), 119.7 (+, 1 × *C*H_Ar_), 113.7 (C_q_, 1 × *C*_qAr_) ppm. − IR (ATR): ṽ = 3306 (w), 2922 (w), 2851 (w), 1660 (w), 1585 (m), 1503 (w), 1414 (w), 1347 (w), 1276 (m), 1179 (m), 1039 (w), 875 (w), 812 (vw), 767 (w), 719 (w), 624 (vw), 577 (w), 446 (vw) cm^–1^. – HRMS (FAB, C_17_H_7_^2^H_2_O_5_^79^Br): calc. 373.9753; found 373.9755. The HRMS was measured from the MeOD NMR sample, therefore the two OH protons exchanged with deuterium.
Additional reaction details and data obtained from the characterization of the target compound can be accessed at: <https://dx.doi.org/10.14272/reaction/SA-FUHFF-UHFFFADPSC-BCJAVVNVCV-UHFFFADPSC-NUHFF-NUHFF-NUHFF-ZZZ>.

***N*-(4-Bromo-3-(1,4-dioxo-1,4-dihydronaphthalene-2-carbonyl)phenyl)acetamide (5f)**

Following **GP B** the crude product was obtained from CAN (2.51 g, 4.58 mmol, 3.70 equiv.) and *N*-(4-bromo-3-(1,4-dimethoxy-2-naphthoyl)phenyl)acetamide (**4e**) (530 mg, 1.24 mmol, 1.00 equiv.). The product **5f** was isolated as an orange solid (389 mg, 977 µmol, 79%). – *R*_f_ (cHex/EtOAc = 1:1) = 0.47. − ^1^H NMR (500 MHz, CDCl_3_): *δ* = 8.14−8.07 (m, 2H, C*H*_Ar_), 7.84−7.80 (m, 3H, C*H*_Ar_), 7.69 (d, ^4^*J* = 2.7 Hz, 1H, C*H*_Ar_), 7.53 (d, ^3^*J* = 8.8 Hz, 1H, C*H*_Ar_), 7.51 (brs, 1H, N*H*), 7.18 (s, 1H, C=C*H*), 2.19 (s, 3H, COC*H*_3_) ppm. − ^13^C NMR (126 MHz, CDCl_3_): *δ* = 192.3 (C_q_, 1 × *C*=O), 185.0 (C_q_, 1 × *C*=O), 182.7 (C_q_, 1 × *C*=O), 168.7 (C_q_, 1 × *C*=O), 145.7 (C_q_, 1 × *C*_qAr_), 139.2 (C_q_, 1 × *C*_qAr_), 138.1 (C_q_, 1 × *C*_qAr_), 137.5 (+, 1 × *C*H_Ar_), 134.8 (+, 1 × *C*H_Ar_), 134.6 (+, 1 × *C*H_Ar_), 134.5 (+, 1 × *C*H_Ar_), 132.2 (C_q_, 1 × *C*_qAr_), 131.8 (C_q_, 1 × *C*_qAr_), 127.1 (+, 1 × *C*H_Ar_), 126.6 (+, 1 × *C*H_Ar_), 124.7 (+, 1 × *C*H_Ar_), 121.7 (+, 1 × *C*H_Ar_), 114.9 (C_q_, 1 × *C*_q_), 24.8 (+, 1 × CO-*C*H_3_) ppm. − IR (ATR): ṽ = 3367 (vw), 2926 (vw), 1654 (w), 1581 (w), 1530 (w), 1466 (w), 1392 (w), 1295 (w), 1250 (w), 1233 (w), 1099 (vw), 1046 (vw), 1013 (w), 892 (vw), 826 (vw), 776 (w), 758 (w), 723 (vw), 661 (vw), 574 (vw), 487 (vw), 416 (vw) cm^–1^. – MS (FAB, 3-NBA), *m*/*z* (%): 398 (5) [M+H]^+^, 307 (35), 154 (100). – HRMS (FAB, C_19_H_13_O_4_N^79^Br): calc. 398.0028; found 398.0027.
Additional reaction details and data obtained from the characterization of the target compound can be accessed at: <https://dx.doi.org/10.14272/reaction/SA-FUHFF-UHFFFADPSC-MWUPABMFGF-UHFFFADPSC-NUHFF-NUHFF-NUHFF-ZZZ>.

***N*-(4-Bromo-3-(1,4-dioxo-1,4-dihydronaphthalene-2-carbonyl)phenyl)-2,2,2-trifluoroacetamide (5g)**

Following **GP B** the crude product was obtained from CAN (1.43 g, 2.61 mmol, 3.70 equiv.) and *N*-(4-bromo-3-(1,4-dimethoxy-2-naphthoyl)phenyl)-2,2,2-trifluoroacetamide (**4f**) (340 mg, 710 µmol, 1.00 equiv.). The product **5g** was isolated as an orange solid (296 mg, 655 µmol, 92%). – *R*_f_ (cHex/EtOAc = 4:1) = 0.33. − ^1^H NMR (400 MHz, CDCl_3_): *δ* = 8.19–8.12 (m, 2H, NH, CH_Ar_), 8.11–8.07 (m, 1H, CH_Ar_), 7.86 (dd, ^3^J = 8.7 Hz, ^4^J = 2.8 Hz, 1H, CH_Ar_), 7.83–7.81 (m, 2H, CH_Ar_), 7.78 (d, ^4^J = 2.8 Hz, 1H, CH_Ar_), 7.63 (d, ^3^J = 8.7 Hz, 1H, CH_Ar_), 7.27 (s, 1H, C=CH) ppm. − ^13^C NMR (101 MHz, CDCl_3_): *δ* = 191.9 (C_q_, 1 × *C*=O), 184.9 (C_q_, 1 × *C*=O), 182.8 (C_q_, 1 × *C*=O), 145.3 (C_q_, 1 × *C*_qAr_), 140.0 (C_q_, 1 × *C*_qAr_), 138.0 (+, 1 × C=*C*H), 135.4 (C_q_, 1 × *C*_qAr_), 134.9 (+, 2 × *C*H_Ar_), 134.7 (+, 1 × *C*H_Ar_), 132.2 (C_q_, 1 × *C*_qAr_), 131.7 (C_q_, 1 × *C*_qAr_), 127.1 (+, 1 × *C*H_Ar_), 126.7 (+, 1 × *C*H_Ar_), 125.0 (+, 1 × *C*H_Ar_), 122.5 (+, 1 × *C*H_Ar_), 117.3 (C_q_, 1 × *C*_qAr_) ppm. Missing signals (C_q_, 1 × *C*=O, 1 × *C*OCF_3_, 1 × *C*F_3_). – IR (ATR): ṽ = 3350 (vw), 1727 (w), 1654 (w), 1583 (w), 1542 (w), 1469 (w), 1406 (w), 1349 (vw), 1299 (w), 1136 (m), 1047 (w), 881 (w), 830 (w), 777 (w), 760 (w), 724 (w), 670 (w), 632 (w), 580 (w), 495 (w), 458 (vw) cm^–1^. – MS (APCI), *m*/*z* (%): 452/454 (97/100) [M+H]^+^. – HRMS (APCI, C_19_H_10_^79^BrF_3_NO_4_): calc. 451.9745; found 451.9729.
Additional reaction details and data obtained from the characterization of the target compound can be accessed at: <https://dx.doi.org/10.14272/reaction/SA-FUHFF-UHFFFADPSC-YREQDOIZJY-UHFFFADPSC-NUHFF-NUHFF-NUHFF-ZZZ>.

**Cycloaddition product of 2‑(2‑iodobenzoyl)naphthalene-1,4-dione (5a)** **and isoprene (6b) (8/9ab)**

According to **GP C** the cycloaddition was performed with 2‑(2‑iodobenzoyl)naphthalene-1,4-dione (**5a**) (388 mg, 1.00 mmol, 1.00 equiv.) and isoprene (**6b**) (0.50 mL, 341 mg, 5.00 mmol, 5.00 equiv.) in dry CH_2_Cl_2_ (2.0 mL). After 3 h the crude product was purified *via* flash chromatography on silica gel (cHex/EtOAc = 8:1) to obtain an off-white solid (289 mg, 633 µmol, 63%). The products **8/9ab** were isolated as a non-separable mixture in a 7.1 : 1 ratio as estimated by ^1^H NMR. Both possible regioisomers are drawn, as the exact structure of the products could not be resolved by analysis of the NMR spectra. Below the analytics of the major product are given. – *R*_f_ (cHex/EtOAc = 6:1) = 0.38. – ^1^H NMR (400 MHz, CDCl_3_): *δ* = 8.15 (dd, ^3^*J* = 7.6 Hz, ^4^*J* = 1.5 Hz, 1H, C*H*_Ar_), 8.02 (dd, ^3^*J* = 7.6 Hz, ^4^*J* = 1.5 Hz, 1H, C*H*_Ar_), 7.86 (dd, ^3^*J* = 8.0 Hz, ^4^*J* = 1.6 Hz, 1H, C*H*_Ar_), 7.75 (dtd, *J* = 21.0 Hz, ^3^*J* = 7.4 Hz, ^4^*J* = 1.5 Hz, 2H, C*H*_Ar_), 7.36–7.25 (m, 1H, C*H*_Ar_), 7.16 (dd, ^3^*J* = 7.7 Hz, ^4^*J* = 1.7 Hz, 1H, C*H*_Ar_), 7.07 (td, ^3^*J* = 7.7 Hz, ^4^*J* = 1.7 Hz, 1H, C*H*_Ar_), 5.34 (ddd, ^3^*J* = 4.8, 3.3 Hz, ^4^*J* = 1.6 Hz, 1H, C=C*H*), 3.72 (dd, ^3^*J* = 8.4, 7.1 Hz, 1H, C*H*), 2.90 (ddq, ^2^*J* = 17.5 , ^3^*J* = 3.9 Hz, ^4^*J* = 1.9 Hz, 1H, C*H*H), 2.46 (dq, ^2^*J* = 17.5 Hz, ^3^*J* = 2.3 Hz, 1H, C*H*H), 2.25 (d, ^3^*J* = 7.4 Hz, 2H, C*H*_2_), 1.65 (s, 3H, C*H*_3_) ppm. – ^13^C NMR (101 MHz, CDCl_3_): *δ* = 200.8 (C_q_, 1 × *C*=O), 196.1 (C_q_, 1 × *C*=O), 194.5 (C_q_, 1 × *C*=O), 142.5 (C_q_, 1 × *C*_qAr_), 140.9 (+, 1 × *C*H_Ar_), 135.2 (+, 1 × *C*H_Ar_), 134.3 (+, 1 × *C*H_Ar_), 133.0 (C_q_, 1 × *C*_qAr_), 132.0 (C_q_, 1 × *C*_qAr_), 131.6 (+, 1 × *C*H_Ar_), 127.5 (+, 1 × *C*H_Ar_), 127.4 (+, 1 × *C*H_Ar_), 127.1 (+, 1 × *C*H_Ar_), 126.9 (+, 1 × *C*H_Ar_), 117.9 (+, 1 × C=*C*H), 93.2 (C_q_, 1 × *C*_qAr_), 67.6 (C_q_, 1 × *C*_q_), 50.3 (+, 1 × *C*H), 30.4 (–, 1 × *C*H_2_), 28.9 (–, 1 × *C*H_2_), 23.2 (+, 1 × *C*H_3_) ppm. Signal missing (C_q_, 1 × *C*_q_). – IR (ATR): ṽ = 2910 (vw), 1676 (m), 1592 (w), 1423 (w), 1317 (vw), 1273 (w), 1253 (w), 1218 (w), 1161 (vw), 1061 (vw), 1010 (w), 967 (vw), 936 (w), 897 (vw), 794 (w), 761 (w), 738 (w), 677 (w), 637 (vw), 588 (w), 519 (vw), 430 (vw) cm^–1^. – MS (FAB, 3‑NBA), *m*/*z* (%): 457 (23) [M+H]^+^, 231 (100), 225 (21), 154 (66). – HRMS (FAB, C_22_H_18_IO_3_): calc. 457.0295; found 457.0294.
Additional reaction details and data obtained from the characterization of the target compound can be accessed at: <https://dx.doi.org/10.14272/reaction/SA-FUHFF-UHFFFADPSC-HTUXSVMUHU-UHFFFADPSC-NUHFF-NUWWY-NUHFF-ZZZ>.

**Cycloaddition product of 2‑(2‑bromobenzoyl)naphthalene-1,4-dione (5b) and isoprene (6b) (8/9bb)**

According to **GP C** the cycloaddition ran with 2‑(2‑bromobenzoyl)naphthalene-1,4-dione (**5b**) (190 mg, 560 µmol, 1.00 equiv.) and isoprene (**6b**) (0.28 mL, 190 mg, 2.79 mmol, 5.00 equiv.) in dry CH_2_Cl_2_ (2.0 mL). After 3 h the crude product was purified *via* flash chromatography on silica gel (cHex/EtOAc = 9:1 🡒 7:1) to obtain an off-white solid (121 mg, 296 µmol, 53%). The products **8/9bb** were isolated as a non-separable mixture in in a 7.7 : 1 ratio as estimated by ^1^H NMR. Both possible regioisomers are drawn, as the exact structure of the products could not be resolved by analysis of the NMR spectra. Below the analytics of the major product are given. – *R*_f_ (cHex/EtOAc = 9:1) = 0.32. – ^1^H NMR (400 MHz, CDCl_3_): *δ* = 8.14 (dd, ^3^*J* = 7.6 Hz, ^4^*J* = 1.5 Hz, 1H, C*H*_Ar_), 8.03 (dd, ^3^*J* = 7.6 Hz, ^4^*J* = 1.5 Hz, 1H, C*H*_Ar_), 7.83–7.70 (m, 2H, C*H*_Ar_), 7.60–7.52 (m, 1H, C*H*_Ar_), 7.34–7.13 (m, 3H, C*H*_Ar_), 5.34–5.30 (m, 1H, C=C*H*), 3.74 (t, ^3^*J* = 7.6 Hz, 1H, C*H*), 2.93–2.85 (m, 1H, C*H*H), 2.51–2.44 (m, 1H, C*H*H), 2.30–2.25 (m, 2H, C*H*_2_), 1.64 (s, 3H, C*H*_3_) ppm. – ^13^C NMR (101 MHz, CDCl_3_): *δ* = 200.1 (C_q_, 1 × *C*=O), 196.2 (C_q_, 1 × *C*=O), 194.5 (C_q_, 1 × *C*=O), 139.1 (C_q_, 1 × *C*_qAr_), 135.3 (+, 1 × *C*H_Ar_), 134.3 (+, 1 × *C*H_Ar_), 134.2 (C_q_, 1 × *C*_qAr_), 134.0 (+, 1 × *C*H_Ar_), 133.1 (C_q_, 1 × *C*_qAr_), 132.2 (C_q_, 1 × *C*_qAr_), 131.5 (+, 1 × *C*H_Ar_), 127.4 (+, 1 × *C*H_Ar_), 127.2 (+, 1 × *C*H_Ar_), 127.1 (+, 1 × *C*H_Ar_), 126.8 (+, 1 × *C*H_Ar_), 120.0 (C_q_, 1 × *C*_q_), 117.8 (+, 1 × C=*C*H), 67.8 (C_q_, 1 × *C*_q_), 50.0 (+, 1 × *C*H), 30.2 (–, 1 × *C*H_2_), 28.7 (–, 1 × *C*H_2_), 23.14 (+, 1 × *C*H_3_) ppm. – IR (ATR): ṽ = 2911 (w), 1679 (s), 1592 (m), 1427 (m), 1373 (w), 1274 (m), 1254 (s), 1218 (s), 1161 (w), 1052 (w), 1017 (m), 968 (w), 936 (m), 897 (w), 794 (m), 761 (m), 738 (s), 683 (m), 639 (w), 589 (m), 549 (w), 459 (w), 431 (w) cm^–1^. – MS (FAB, 3‑NBA), *m*/*z* (%): 409/411 (16/14) [M+H]^+^, 408/410 (2/6) [M]^+^, 225 (43), 183/185 (100/98). – HRMS (FAB, C_21_H_16_^79^BrO_3_N): calc. 409.0314; found 409.0314.
Additional reaction details and data obtained from the characterization of the target compound can be accessed at: <https://dx.doi.org/10.14272/reaction/SA-FUHFF-UHFFFADPSC-UWSTXCXPGI-UHFFFADPSC-NUHFF-NUWWY-NUHFF-ZZZ>.

**(4a*R*,9a*R*)-4a-(2-Bromobenzoyl)-2,3-dimethyl-1,4,4a,9a-tetrahydroanthracene-9,10-dione (8bc)**

According to **GP C** the cycloaddition was performed with 2‑(2‑bromobenzoyl)naphthalene-1,4-dione (**5b**) (341 mg, 1.00 mmol, 1.00 equiv.) and 2,3-dimethylbuta-1,3-diene (**6c**) (0.34 mL, 246 mg, 3.00 mmol, 3.00 equiv.) in dry CH_2_Cl_2_ (5.0 mL). After 3 h the crude product was purified *via* flash chromatography on silica gel (cHex/EtOAc = 9:1) to obtain product **8bc** as a yellow solid (373 mg, 881 µmol, 88%). – *R*_f_ (cHex/EtOAc = 9:1) = 0.29. – ^1^H NMR (500 MHz, CDCl_3_): *δ* = 8.14 (dd, ^3^*J* = 7.6 Hz, ^4^*J* = 1.4 Hz, 1H, C*H*_Ar_), 8.03 (dd, ^3^*J* = 7.7 Hz, ^4^*J* = 1.4 Hz, 1H, C*H*_Ar_), 7.78 (td, ^3^*J* = 7.5 Hz, ^4^*J* = 1.5 Hz, 1H, C*H*_Ar_), 7.74 (td, ^3^*J* = 7.5 Hz, ^4^*J* = 1.5 Hz, 1H, C*H*_Ar_), 7.59–7.56 (m, 1H, C*H*_Ar_), 7.31–7.23 (m, 3H, C*H*_Ar_), 3.73 (dd, ^3^*J* = 8.5, 6.7 Hz, 1H, C*H*), 2.79 (dt, ^2^*J* = 16.8 Hz, ^3^*J* = 2.2 Hz, 1H, C*H*H), 2.45–2.22 (m, 3H, C*H*H, C*H*_2_), 1.60 (s, 3H, C*H*_3_), 1.51 (s, 3H, C*H*_3_) ppm. – ^13^C NMR (125 MHz, CDCl_3_): *δ* = 200.2 (C_q_, 1 × *C*=O), 196.5 (C_q_, 1 × *C*=O), 194.9 (C_q_, 1 × *C*=O), 139.2 (C_q_, 1 × *C*_qAr_), 135.2 (+, 1 × *C*H_Ar_), 134.4 (C_q_, 1 × *C*_qAr_), 134.3 (+, 1 × *C*H_Ar_), 134.1 (+, 1 × *C*H_Ar_), 133.2 (C_q_, 1 × *C*_qAr_), 131.4 (+, 1 × *C*H_Ar_), 127.5 (+, 1 × *C*H_Ar_), 127.4 (+, 1 × *C*H_Ar_), 127.2 (+, 1 × *C*H_Ar_), 126.7 (+, 1 × *C*H_Ar_), 124.2 (C_q_, 1 × *C*_qAr_), 122.9 (C_q_, 1 × *C*_q_), 120.0 (C_q_, 1 × *C*_q_), 68.6 (C_q_, 1 × *C*_q_), 50.2 (+, 1 × *C*H), 34.4 (–, 1 × *C*H_2_), 31.6 (–, 1 × *C*H_2_), 19.1 (+, 1 × *C*H_3_), 18.8 (+, 1 × *C*H_3_) ppm. – IR (ATR): ṽ = 1694 (vs), 1679 (vs), 1589 (m), 1273 (m), 1256 (vs), 1247 (vs), 1214 (m), 1201 (s), 1057 (m), 1027 (m), 972 (m), 887 (m), 790 (m), 765 (s), 738 (vs), 711 (s), 688 (m), 674 (m), 635 (m), 586 (s), 551 (m), 439 (m), 422 (m), 408 (w), 394 (w) cm^–1^. − MS (FAB, 3‑NBA), *m*/*z* (%): 423/425 (24/21) [M+H]^+^, 239 (100), 183/185 (92/89). – HRMS (FAB, C_23_H_20_O_3_^79^Br): calc. 423.0596; found 423.0597. – X‑Ray: The structure of **8bc** could be confirmed by single crystal X‑Ray diffraction (see 4 Crystallographic Information, CCDC 1992883).
Additional reaction details and data obtained from the characterization of the target compound can be accessed at: <https://dx.doi.org/10.14272/reaction/SA-FUHFF-UHFFFADPSC-VQHSHKAJCS-UHFFFADPSC-NUHFF-NUHFF-NUHFF-ZZZ>.

**Cycloaddition products of 2-(2-iodobenzoyl)naphthalene-1,4-dione (73a) and 1‑(trimethylsiloxy)-1,3-butadiene (6d) (8ad**, **9ad)**

According to **GP C** the cycloaddition was performed with 2‑(2‑iodobenzoyl)naphthalene-1,4-dione (**5a**) (97.0 mg, 250 µmol, 1.00 equiv.) and 1‑(trimethylsiloxy)-1,3-butadiene (**6d**) (0.22 mL, 178 mg, 1.25 mmol, 5.00 equiv.) in dry CH_2_Cl_2_ (1.0 mL). After 3 h the crude product was purified *via* flash chromatography on silica gel (cHex/EtOAc = 6:1) to obtain isomers **8ad** (26.5 mg, 50.0 µmol, 20%) and **9ad** (90.1 mg, 170 µmol, 68%) as yellow solids.

**9ad**: *R*_f_ (cHex/EtOAc = 9:1) = 0.31. – ^1^H NMR (400 MHz, CDCl_3_): *δ* = 8.19–8.12 (m, 1H, C*H*_Ar_), 8.07–8.01 (m, 1H, C*H*_Ar_), 7.97 (dd, ^3^*J* = 7.9, ^4^*J* = 1.2 Hz, 1H, C*H*_Ar_), 7.82–7.64 (m, 3H, C*H*_Ar_), 7.33 (td, ^3^*J* = 7.6 Hz, ^4^*J* = 1.2 Hz, 1H, C*H*_Ar_), 7.09 (td, ^3^*J* = 7.7 Hz, ^4^*J* = 1.6 Hz, 1H, C*H*_Ar_), 5.92 (ddd, ^3^*J* = 10.2, 4.6 Hz, ^4^*J* = 2.7 Hz, 1H, C=C*H*), 5.72 (ddt, ^3^*J* = 10.0, 5.2 Hz, ^4^*J* = 2.3 Hz, 1H, C=C*H*), 4.97 (d, ^3^*J* = 5.2 Hz, 1H, C=CH-C*H*), 3.84 (d, ^3^*J* = 7.0 Hz, 1H, CH_2_-C*H*), 3.18 (ddt, ^2^*J* = 19.1 Hz, ^3^*J* = 4.2 Hz, ^4^*J* = 1.5 Hz, 1H, CH*H*), 2.27–2.06 (m, 1H, CH*H*), –0.34 (s, 9H, SiC*H*_3_) ppm. – ^13^C NMR (101 MHz, CDCl_3_): *δ* = 198.8 (C_q_, 1 × *C*=O), 196.9 (C_q_, 1 × *C*=O), 195.0 (C_q_, 1 × *C*=O), 142.4 (+, 1 × *C*H_Ar_), 141.3 (C_q_, 1 × *C*_qAr_), 138.0 (C_q_, 1 × *C*_qAr_), 135.5 (C_q_, 1 × *C*_qAr_), 134.8 (+, 1 × *C*H_Ar_), 133.6 (+, 1 × *C*H_Ar_), 132.2 (+, 1 × *C*H_Ar_), 130.1 (+, 1 × C=*C*H), 128.1 (+, 1 × *C*H_Ar_), 127.8 (+, 1 × *C*H_Ar_), 127.3 (+, 1 × *C*H_Ar_), 127.1 (+, 1 × C=*C*H), 126.1 (+, 1 × *C*H_Ar_), 95.3 (C_q_, 1 × *C*_qAr_), 70.2 (C_q_, 1 × *C*_q_), 68.5 (+, 1 × *C*H), 46.4 (+, 1 × *C*H), 21.6 (–, 1 × *C*H_2_), –0.2 (+, 3 × Si*C*H_3_) ppm. – IR (ATR): ṽ = 2952 (vw), 1700 (w), 1672 (w), 1593 (w), 1422 (w), 1322 (vw), 1248 (m), 1229 (w), 1076 (w), 1053 (w), 1013 (w), 934 (w), 887 (w), 838 (m), 750 (w), 735 (w), 693 (w), 637 (w), 593 (w), 482 (vw), 449 (vw) cm^–1^. − MS (FAB, 3‑NBA), *m*/*z* (%): 531 (13) [M+H]^+^, 389 (28), 231 (100). – HRMS (FAB, C_24_H_24_^127^IO_4_Si): calc. 531.0489; found 531.0488. – X‑Ray: The structure of **9ad** could be confirmed by single crystal X‑Ray diffraction (see 4 Crystallographic Information, CCDC 1992181).
Additional reaction details and data obtained from the characterization of the target compound can be accessed at: <https://dx.doi.org/10.14272/reaction/SA-FUHFF-UHFFFADPSC-ZUBFTCRJYO-UHFFFADPSC-NUHFF-NRFRK-NUHFF-ZZZ>.

**8ad**: *R*_f_ (cHex/EtOAc = 9:1) = 0.29. – ^1^H NMR (400 MHz, CDCl_3_): *δ* = 8.08 (dd, ^3^*J* = 7.8 Hz, ^4^*J* = 1.2 Hz, 1H, C*H*_Ar_), 7.94 (d, ^3^*J* = 7.8 Hz, 1H, C*H*_Ar_), 7.85 (dd, ^2^*J* = 16.4, ^3^*J* = 7.9 Hz, 2H, C*H*_Ar_), 7.75 (t, ^3^*J* = 7.5 Hz, 1H, C*H*_Ar_), 7.66 (t, ^3^*J* = 7.6 Hz, 1H, C*H*_Ar_), 7.38 (t, ^3^*J* = 7.7 Hz, 1H, C*H*_Ar_), 7.08 (t, ^3^*J* = 7.6 Hz, 1H, C*H*_Ar_), 6.09–5.94 (m, 1H, C=C*H*), 5.82 (ddd, ^3^*J* = 10.5, 5.1, ^4^*J* = 2.5 Hz, 1H, C=C*H*), 5.38 (d, ^3^*J* = 5.2 Hz, 1H, C=CH-C*H*), 3.88 (dd, ^3^*J* = 11.9, 6.6 Hz, 1H, CH_2_-C*H*), 2.53 (dt, ^2^*J* = 18.8 Hz, ^3^*J* = 5.7 Hz, 1H, CH*H*), 2.05 (dd, ^2^*J* = 18.7 Hz, ^3^*J* = 12.2 Hz, 1H, CH*H*), –0.03 (s, 9H, C*H*_3_) ppm. – ^13^C NMR (101 MHz, CDCl_3_): *δ* = 197.0 (C_q_, 1 × *C*=O), 195.5 (C_q_, 1 × *C*=O), 193.1 (C_q_, 1 × *C*=O), 141.8 (+, 1 × *C*H_Ar_), 140.6 (C_q_, 1 × *C*_qAr_), 135.1 (+, 1 × *C*H_Ar_), 134.3 (C_q_, 1 × *C*_qAr_), 133.9 (+, 1 × *C*H_Ar_), 133.6 (C_q_, 1 × *C*_qAr_), 131.9 (+, 1 × *C*H_Ar_), 128.0 (+, 1 × *C*H_Ar_), 127.5 (+, 1 × C=*C*H), 127.2 (+, 1 × *C*H_Ar_), 127.1 (+, 1 × *C*H_Ar_), 127.0 (+, 1 × *C*H_Ar_), 126.6 (+, 1 × C=*C*H), 94.9 (C_q_, 1 × *C*_qAr_), 73.3 (C_q_, 1 × *C*_q_), 65.9 (+, 1 × *C*H), 46.0 (+, 1 × *C*H), 27.8 (–, 1 × *C*H_2_), 0.4 (+, 3 × Si*C*H_3_) ppm. – IR (ATR): ṽ = 2952 (vw), 1700 (w), 1672 (w), 1593 (w), 1422 (w), 1322 (vw), 1248 (m), 1229 (w), 1076 (w), 1053 (w), 1013 (w), 934 (w), 887 (w), 838 (m), 750 (w), 735 (w), 693 (w), 637 (w), 593 (w), 482 (vw), 449 (vw) cm^–1^. – MS (FAB, 3-NBA), *m*/*z* (%): 459 (39) [M−TMS+H]^+^, 458 (71) [M−TMS]^+^, 441 (8) [M−OTMS]^+^, 415 (100). – HRMS (FAB, C_24_H_23_^127^IO_4_Si): calc. 530.0410; found 530.0411. – X‑Ray: The structure of **8ad** could be confirmed by single crystal X‑Ray diffraction (see 4 Crystallographic Information, CCDC 1992884).
Additional reaction details and data obtained from the characterization of the target compound can be accessed at: <https://dx.doi.org/10.14272/reaction/SA-FUHFF-UHFFFADPSC-ZUBFTCRJYO-UHFFFADPSC-NUHFF-NRFRK-NUHFF-ZZZ>.

**Cycloaddition products of 2-(2-bromobenzoyl)naphthalene-1,4-dione (5b) and 1‑(trimethylsiloxy)-1,3-butadiene (6d) (8bd**, **9bd)**

According to **GP C** the cycloaddition was performed with 2‑(2‑bromobenzoyl)naphthalene-1,4-dione (**5b**) (85.3 mg, 250 µmol, 1.00 equiv.) and 1‑(trimethylsiloxy)-1,3-butadiene (**6d**) (0.22 mL, 178 mg, 1.25 mmol, 5.00 equiv.) in dry CH_2_Cl_2_ (1.0 mL). After 4 h the crude product was purified *via* flash chromatography on silica gel (cHex/EtOAc = 9:1) to obtain isomers **8bd** (24.1 mg, 50.0 µmol, 20%) and **9bd** (73.7 mg, 153 µmol, 61%) as yellow solids.

**9bd**: *R_f_* (cHex/EtOAc = 9:1) = 0.36. – ^1^H NMR (400 MHz, CDCl_3_): *δ* = 8.17–8.12 (m, 1H, C*H*_Ar_), 8.06–8.00 (m, 1H, C*H*_Ar_), 7.81–7.69 (m, 3H, C*H*_Ar_), 7.62 (dd, ^3^*J* = 7.8 Hz, ^4^*J* = 1.3 Hz, 1H, C*H*_Ar_), 7.45–7.18 (m, 2H, C*H*_Ar_), 5.90 (ddd, ^3^*J* = 10.1, 4.5 Hz, ^4^*J* = 2.8 Hz, 1H, C=C*H*), 5.67 (ddt, ^3^*J* = 10.0, 5.0 Hz, ^4^*J* = 2.3 Hz, 1H, C=C*H*), 4.94 (d, ^3^*J* = 5.2 Hz, 1H, C=CH-C*H*), 3.89 (d, ^3^*J* = 7.1 Hz, 1H, CH_2_‑C*H*), 3.22–3.16 (m, 1H, C*H*H), 2.30–2.06 (m, 1H, CH*H*), –0.35 (s, 9H, Si(C*H*_3_)_3_) ppm. – ^13^C NMR (101 MHz, CDCl_3_): *δ* = 197.9 (C_q_, 1 × *C*=O), 196.6 (C_q_, 1 × *C*=O), 194.8 (C_q_, 1 × *C*=O), 138.6 (C_q_, 1 × *C*_qAr_), 137.6 (C_q_, 1 × *C*_qAr_), 135.1 (C_q_, 1 × *C*_qAr_), 134.8 (+, 1 × *C*H_Ar_), 134.4 (+, 1 × *C*H_Ar_), 133.3 (+, 1 × *C*H_Ar_), 131.7 (+, 1 × *C*H_Ar_), 130.1 (+, 1 × C=*C*H), 128.3 (+, 1 × *C*H_Ar_), 126.9 (+, 1 × *C*H_Ar_), 126.8 (+, 1 × *C*H_Ar_), 126.5 (+, 1 × C=*C*H), 125.8 (+, 1 × *C*H_Ar_), 121.2 (C_q_, 1 × *C*_qAr_), 70.2 (C_q_, 1 × *C*_q_), 68.2 (+, 1 × *C*H), 45.9 (+, 1 × *C*H), 21.1 (–, 1 × *C*H_2_), –0.6 (+, 3 × Si*C*H_3_) ppm. – IR (ATR): ṽ = 3066 (vw), 3033 (vw), 2955 (vw), 2898 (vw), 1701 (m), 1672 (w), 1593 (w), 1425 (w), 1324 (vw), 1249 (m), 1160 (vw), 1076 (w), 1056 (w), 1021 (w), 935 (w), 885 (w), 839 (m), 751 (w), 737 (w), 688 (w), 641 (w), 596 (vw), 542 (vw), 483 (vw), 456 (vw), 437 (vw) cm^–1^. – MS (FAB, 3-NBA), *m*/*z* (%): 483 (42) [M+H]^+^, 183 (100) [M−C_17_H_19_O_3_Si]^+^, 393 (13) [M−OTMS]^+^. – HRMS (FAB, C_24_H_24_^79^BrO_4_Si): calc. 483.0627, found 483.0629. – X‑Ray: The structure of **9bd** could be confirmed by single crystal X‑Ray diffraction (see 4 Crystallographic Information, CCDC 1992885).
Additional reaction details and data obtained from the characterization of the target compound can be accessed at: <https://dx.doi.org/10.14272/reaction/SA-FUHFF-UHFFFADPSC-LRHPRUPFRY-UHFFFADPSC-NUHFF-NRFRK-NUHFF-ZZZ>.

**8bd**: *R*_f_ (cHex/EtOAc = 9:1) = 0.30. – ^1^H NMR (400 MHz, CDCl_3_): *δ* = 8.07 (dd, ^3^*J* = 7.8 Hz, ^4^*J* = 1.4 Hz, 1H, C*H*_Ar_), 7.93 (dd, ^3^*J* = 7.8 Hz, ^4^*J* = 1.4 Hz, 1H, C*H*_Ar_), 7.79 (dd, ^3^*J* = 7.8 Hz, ^4^*J* = 1.7 Hz, 1H, C*H*_Ar_), 7.74 (td, ^3^*J* = 7.6 Hz, ^4^*J* = 1.4 Hz, 1H, C*H*_Ar_), 7.66 (td, ^3^*J* = 7.5 Hz, ^4^*J* = 1.4 Hz, 1H, C*H*_Ar_), 7.53 (dd, ^3^*J* = 7.9 Hz, ^4^*J* = 1.2 Hz, 1H, C*H*_Ar_), 7.34 (td, ^3^*J* = 7.6 Hz, ^4^*J* = 1.3 Hz, 1H, C*H*_Ar_), 7.29–7.22 (m, 1H, C*H*_Ar_), 6.01 (ddd, ^3^*J* = 9.8, 5.0 Hz, ^4^*J* = 2.4 Hz, 1H, C=C*H*), 5.82 (ddd, ^3^*J* = 10.0, 4.8 Hz, ^4^*J* = 2.4 Hz, 1H, C=C*H*), 5.36 (dd, ^3^*J* = 5.3 Hz, ^4^*J* = 1.0 Hz, 1H, C=CH-C*H*), 3.87 (dd, ^3^*J* = 11.8, 6.6 Hz, 1H, CH_2_-C*H*), 2.58–2.50 (m, 1H, CH*H*), 2.13–1.97 (m, 1H, CH*H*), –0.03 (s, 9H, Si(C*H*_3_)_3_) ppm. – ^13^C NMR (101 MHz, CDCl_3_): *δ* = 196.9 (C_q_, 1 × *C*=O), 196.1 (C_q_, 1 × *C*=O), 193.6 (C_q_, 1 × *C*=O), 138.5 (C_q_, 1 × *C*_qAr_), 135.6 (+, 1 × *C*H_Ar_), 135.2 (+, 1 × *C*H_Ar_), 134.8 (C_q_, 1 × *C*_qAr_), 134.4 (+, 1 × *C*H_Ar_), 134.2 (C_q_, 1 × *C*_qAr_), 132.5 (+, 1 × *C*H_Ar_), 128.8 (+, 1 × C=*C*H), 128.0 (+, 1 × *C*H_Ar_), 127.7 (+, 1 × *C*H_Ar_), 127.5 (+, 1 × *C*H_Ar_), 127.4 (+, 1 × C=*C*H), 127.1 (+, 1 × *C*H_Ar_), 122.1 (C_q_, 1 × *C*_qAr_), 74.2 (C_q_, 1 × *C*_q_), 66.3 (+, 1 × *C*H), 46.6 (+, 1 × *C*H), 28.4 (–, 1 × *C*H_2_), 0.9 (+, 3 × Si*C*H_3_) ppm. – IR (ATR): ṽ = 2922 (vw), 1698 (w), 1678 (w), 1593 (w), 1467 (vw), 1430 (vw), 1249 (w), 1230 (w), 1072 (w), 1052 (w), 1024 (w), 996 (vw), 945 (w), 890 (w), 841 (w), 763 (w), 683 (w), 641 (vw), 593 (vw), 515 (vw), 394 (vw) cm^–1^. – MS (FAB, 3-NBA), *m*/*z* (%): 484/486 (38/32) [M+H]^+^, 483/485 (100/88) [M]^+^. – HRMS (FAB, C_24_H_24_^79^BrO_4_Si): calc. 483.0627; found 483.0625. – X‑Ray: The structure of **8bd** could be confirmed by single crystal X‑Ray diffraction (see 4 Crystallographic Information, CCDC 1992886).
Additional reaction details and data obtained from the characterization of the target compound can be accessed at: <https://dx.doi.org/10.14272/reaction/SA-FUHFF-UHFFFADPSC-LRHPRUPFRY-UHFFFADPSC-NUHFF-NRFRK-NUHFF-ZZZ>.

**Cycloaddition products of 2-(2-iodobenzoyl)naphthalene-1,4-dione (5a) and (*E*)‑trimethyl((2-methylbuta-1,3-dien-1-yl)oxy)silane (6e) (8ae**, **9ae)**

According to **GP C** the cycloaddition was performed with 2‑(2‑iodobenzoyl)naphthalene-1,4-dione (**5a**) (233 mg, 600 µmol, 1.00 equiv.) and (*E*)‑trimethyl((2-methylbuta-1,3-dien-1-yl)oxy)silane (**6e**) (0.34 mL, 281 mg, 1.80 mmol, 3.00 equiv.) in in dry CH_2_Cl_2_ (2.0 mL). After 4 h the crude product was purified *via* flash chromatography on silica gel (cHex/EtOAc = 9:1) to obtain isomers **9ae** (157 mg, 288 µmol, 48%) and **8ae** (84.9 mg, 156 µmol, 26%) as yellow solids.

**9ae**: *R*_f_ (cHex/EtOAc = 9:1) = 0.34. – ^1^H NMR (400 MHz, CDCl_3_): *δ* = 8.16–8.14 (m, 1H, C*H*_Ar_), 8.07–8.05 (m, 2H, C*H*_Ar_), 7.96 (dd, ^3^*J* = 7.9 Hz, ^4^*J* = 1.1 Hz, 1H, C*H*_Ar_), 7.70–7.80 (m, 2H, C*H*_Ar_), 7.41 (td, ^3^*J* = 7.6 Hz, ^4^*J* = 1.2 Hz, 1H, C*H*_Ar_), 7.08 (td, ^3^*J* = 7.6 Hz, ^4^*J* = 1.6 Hz, 1H, C*H*_Ar_), 5.63–5.62 (m, 1H, C=C*H*), 4.68 (s, 1H, C*H*), 3.95 (d, ^3^*J* = 7.8 Hz, 1H, COC*H*), 3.32–3.28 (m, 1H, CH*H*), 3.28–3.24 (m, 1H, CH*H*), 1.29 (d, ^4^*J* = 1.9 Hz, 3H, C*H*_3_), –0.38 (s, 9H, Si(C*H*_3_)_3_) ppm. – ^13^C NMR (101 MHz, CDCl_3_): *δ* = 198.4 (C_q_, 1 × *C*=O), 197.4 (C_q_, 1 × *C*=O), 194.9 (C_q_, 1 × *C*=O), 141.5 (+, 1 × *C*H_Ar_), 141.4 (C_q_, 1 × *C*_qAr_), 137.3 (C_q_, 1 × *C*_qAr_), 134.5 (C_q_, 1 × *C*_qAr_), 134.4 (+, 1 × *C*H_Ar_), 133.0 (+, 1 × *C*H_Ar_), 132.8 (C_q_, 1 × *C*_q_), 131.0 (+, 1 × *C*H_Ar_), 127.9 (+, 1 × *C*H_Ar_), 126.9 (+, 1 × *C*H_Ar_), 126.8 (+, 1 × *C*H_Ar_), 126.2 (+, 1 × *C*H_Ar_), 125.8 (+, 1 × C=*C*H), 95.1 (C_q_, 1 × *C*_qAr_), 72.6 (+, 1 × *C*H), 71.5 (C_q_, 1 × *C*_q_), 45.0 (+, 1 × CO-*C*H), 21.6 (+, 1 × *C*H_3_), 20.7 (–, 1 × *C*H_2_), –0.7 (+, 3 × Si*C*H_3_) ppm. – IR (ATR): ṽ = 3059 (vw), 2955 (vw), 2927 (vw), 2881 (vw), 1692 (m), 1667 (m), 1590 (w), 1422 (w), 1247 (m), 1084 (m), 1011 (w), 872 (m), 841 (m), 802 (w), 768 (m), 751 (m), 735 (m), 715 (m), 607 (m), 541 (w), 453 (w), 390 (w) cm^–1^. – MS (FAB, 3-NBA), *m*/*z* (%): 545 (34) [M+H]^+^, 455 (100) [M–OTMS]^+^. – HRMS (FAB, C_25_H_26_^127^IO_4_Si): calc. 545.0645; found 545.0643. – X‑Ray: The structure of **9ae** could be confirmed by single crystal X‑Ray diffraction (see 4 Crystallographic Information, CCDC 1992887).
Additional reaction details and data obtained from the characterization of the target compound can be accessed at: <https://dx.doi.org/10.14272/reaction/SA-FUHFF-UHFFFADPSC-PHEQJDFJHL-UHFFFADPSC-NUHFF-NUWAF-NUHFF-ZZZ>.

**8ae**: *R*_f_ (cHex/EtOAc = 9:1) = 0.26. – ^1^H NMR (400 MHz, CDCl_3_): *δ* = 7.96 (td, ^3^*J* = 7.5 Hz, ^4^*J* = 1.4 Hz, 2H, C*H*_Ar_), 7.85 (dd, ^3^*J* = 7.9 Hz, ^4^*J* = 1.6 Hz, 1H, C*H*_Ar_), 7.74 (dd, ^3^*J* = 8.0 Hz, ^4^*J* = 1.1 Hz, 1H, C*H*_Ar_), 7.66 (td, ^3^*J* = 7.5 Hz, ^4^*J* = 1.3 Hz, 2H, C*H*_Ar_), 7.42 (td, ^3^*J* = 7.6 Hz, ^4^*J* = 1.2 Hz, 1H, C*H*_Ar_), 7.08 (ddd, ^3^*J* = 8.0, 7.4 Hz, ^4^*J* = 1.6 Hz, 1H, C*H*_Ar_), 5.47 (ddt, ^3^*J* = 4.4, 3.0 Hz, ^4^*J* = 1.5 Hz, 1H, C=C*H*), 5.22 (s, 1H, C(OTMS)*H*), 3.93 (dd, ^3^*J* = 10.9, 7.0 Hz, 1H, COC*H*), 2.63–2.39 (m, 1H, CH*H*), 2.12–1.99 (m, 1H, CH*H*), 1.95 (s, 3H, C*H*_3_), 0.06 (s, 9H, Si(C*H*_3_)_3_) ppm. – ^13^C NMR (101 MHz, CDCl_3_): *δ* = 198.7 (C_q_, 1 × *C*=O), 196.2 (C_q_, 1 × *C*=O), 192.2 (C_q_, 1 × *C*=O), 141.5 (+, 1 × *C*H_Ar_), 140.7 (C_q_, 1 × *C*_qAr_), 137.3 (C_q_, 1 × *C*_qAr_), 135.6 (C_q_, 1 × *C*_qAr_), 135.3 (+, 1 × *C*H_Ar_), 134.9 (+, 1 × *C*H_Ar_), 134.4 (+, 1 × *C*H_Ar_), 133.7 (C_q_, 1 × *C*_q_), 132.1 (+, 1 × *C*H_Ar_), 129.1 (+, 1 × *C*H_Ar_), 127.7 (+, 1 × *C*H_Ar_), 127.5 (+, 1 × *C*H_Ar_), 122.0 (+, 1 × C=*C*H), 95.0 (C_q_, 1 × *C*_qAr_), 74.1 (C_q_, 1 × *C*_q_), 71.4 (+, 1 × *C*H), 45.9 (+, 1 × CO*C*H), 28.4 (–, 1 × *C*H_2_), 22.4 (+, 1 × *C*H_3_), 1.0 (+, 3 × Si*C*H_3_) ppm. – IR (ATR): ṽ = 2957 (w), 2857 (w), 1693 (m), 1586 (m), 1427 (w), 1299 (m), 1246 (m), 1220 (m), 1145 (w), 1112 (w), 1056 (s), 1006 (m), 955 (m), 928 (w), 895 (m), 839 (s), 771 (m), 744 (m), 699 (s), 639 (w), 602 (m), 570 (m), 503 (m), 395 (w) cm^–1^. – MS (FAB, 3-NBA), *m*/*z* (%): 472 (3) [M–TMS]^+^, 309 (25), 251 (26), 197 (43), 135 (100). – HRMS (FAB, C_25_H_26_O_4_^127^ISi): calc. 545.0645; found 545.0647. – X‑Ray: The structure of **8ae** could be confirmed by single crystal X‑Ray diffraction (see 4 Crystallographic Information, CCDC 1992182).
Additional reaction details and data obtained from the characterization of the target compound can be accessed at: <https://dx.doi.org/10.14272/reaction/SA-FUHFF-UHFFFADPSC-PHEQJDFJHL-UHFFFADPSC-NUHFF-NUWAF-NUHFF-ZZZ>.

**(1*S*,4a*R*,9a*S*)-1-((*tert*-Butyldimethylsilyl)oxy)-9a-(2-iodobenzoyl)-2-methyl-1,4,4a,9a-tetrahydroanthracene-9,10-dione (9af) and (1*S*,4a*S*,9a*R*)-1-((*tert*-butyldimethylsilyl)oxy)-9a-(2-iodobenzoyl)-2-methyl-1,4,4a,9a-tetrahydroanthracene-9,10-dione (8af)**

According to **GP C** the cycloaddition was performed with 2‑(2‑iodobenzoyl)naphthalene-1,4-dione (**5a**) (200 mg, 515 µmol, 1.00 equiv.) and (*E*)‑*tert*-butyldimethyl((2-methylbuta-1,3-dien-1-yl)oxy)silane (**6f**) (512 mg, 2.58 mmol, 5.00 equiv.) in dry CH_2_Cl_2_ (7.0 mL). After 6 h the crude product was purified *via* flash chromatography on silica gel (cHex/EtOAc = 9:1) to obtain **9af** (114 mg, 194 µmol, 38%) and **8af** (86.8 mg, 148 µmol, 29%) both as yellow solids.

**9af**: *R*_f_ (cHex/EtOAc = 9:1) = 0.42. – ^1^H NMR (500 MHz, CDCl_3_): *δ* = 8.16–8.08 (m, 2H, C*H*_Ar_), 8.03 (dd, ^3^*J* = 7.5 Hz, ^4^*J* = 1.7 Hz, 1H, C*H*_Ar_), 7.96 (dd, ^3^*J* = 7.9 Hz, ^4^*J* = 1.1 Hz, 1H, C*H*_Ar_), 7.78–7.71 (m, 2H, C*H*_Ar_), 7.41 (td, ^3^*J* = 7.6 Hz, ^4^*J* = 1.2 Hz, 1H, C*H*_Ar_), 7.07 (td, ^3^*J* = 7.6 Hz, ^4^*J* = 1.6 Hz, 1H, C*H*_Ar_), 5.64–5.61 (m, 1H, C=C*H*), 4.76 (s, 1H, C(OTBDMS)*H*), 3.95 (d, ^3^*J* = 8.0 Hz, 1H, COC*H*), 3.39–3.26 (m, 1H, C*H*H), 2.30–2.22 (m, 1H, C*H*H), 1.28 (s, 3H, C*H*_3_), 0.50 (s, 9H, SiC(C*H*_3_)_3_), –0.12 (s, 3H, SiC*H*_3_), –0.70 (s, 3H, SiC*H*_3_) ppm. – ^13^C NMR (125 MHz, CDCl_3_): *δ* = 198.8 (C_q_, 1 × *C*=O), 197.4 (C_q_, 1 × *C*=O), 195.0 (C_q_, 1 × *C*=O), 142.0 (+, 1 × *C*H_Ar_), 141.9 (C_q_, 1 × *C*_qAr_), 137.4 (C_q_, 1 × *C*_qAr_), 134.9 (C_q_, 1 × *C*_qAr_), 134.7 (+, 1 × *C*H_Ar_), 133.4 (+, 1 × *C*H_Ar_), 133.3 (C_q_, 1 × *C*_q_), 131.3 (+, 1 × *C*H_Ar_), 128.1 (+, 1 × *C*H_Ar_), 127.7 (+, 1 × *C*H_Ar_), 127.1 (+, 1 × *C*H_Ar_), 127.0 (+, C=*C*H), 126.4 (+, 1 × *C*H_Ar_), 95.5 (C_q_, 1 × *C*_qAr_), 73.6 (+, 1 × *C*H), 71.9 (C_q_, 1 × *C*_q_), 45.3 (+, 1 × *C*H), 25.4 (+, 3 × *C*H_3_), 22.2 (+, 1 × *C*H_3_), 21.0 (–, 1 × *C*H_2_), 18.2 (C_q_, 1 × *C*_q_), –4.7 (+, 1 × Si*C*H_3_), –4.8 (+, 1 × Si*C*H_3_) ppm. – IR (ATR): ṽ = 2921 (w), 2849 (w), 1697 (w), 1672 (w), 1593 (vw), 1457 (vw), 1424 (vw), 1248 (w), 1219 (w), 1074 (w), 1050 (w), 1009 (w), 930 (vw), 885 (vw), 871 (vw), 836 (w), 781 (w), 765 (w), 735 (w), 717 (w), 701 (w), 679 (w), 637 (vw), 605 (w), 541 (vw), 452 (vw), 416 (vw) cm^–1^. – MS (FAB, 70 eV), *m*/*z* (%): 587 (62) [M+H]^+^, 586 (100) [M]^+^, 529 (82) [M–CMe_3_]^+^, 455 (35) [M–OTBDMS]^+^. – HRMS (EI, C_28_H_31_IO_4_Si): calc. 586.1031; found 586.1033. – X‑Ray: The structure of **9af** could be confirmed by single crystal X‑Ray diffraction (see 4 Crystallographic Information, CCDC 1992183).
Additional reaction details and data obtained from the characterization of the target compound can be accessed at: <https://dx.doi.org/10.14272/reaction/SA-FUHFF-UHFFFADPSC-UMSRHQGWSN-UHFFFADPSC-NUHFF-NHVYL-NUHFF-ZZZ>.

**8af**: *R*_f_ (cHex/EtOAc = 9:1) = 0.33. – ^1^H NMR (500 MHz, CDCl_3_): *δ* = 7.99 (d, ^3^*J* = 7.6 Hz, 1H, C*H*_Ar_), 7.88 (d, ^3^*J* = 7.6 Hz, 1H, C*H*_Ar_), 7.83 (dd, ^3^*J* = 7.6 Hz, ^4^*J* = 1.6 Hz, 1H, C*H*_Ar_), 7.76–7.70 (m, 2H, C*H*_Ar_), 7.65 (td, ^3^*J* = 7.5 Hz, ^4^*J =* 1.3 Hz, 1H, C*H*_Ar_), 7.40 (td, ^3^*J* = 7.6 Hz, ^4^*J =* 1.2 Hz, 1H, C*H*_Ar_), 7.06 (td, ^3^*J* = 7.7 Hz, ^4^*J =* 1.6 Hz, 1H, C*H*_Ar_), 5.46 (s, 1H, C=C*H*), 5.32 (s, 1H, C(OTBDMS)*H*), 4.03 (dd, ^3^*J* = 10.8, 7.7 Hz, 1H, COC*H*), 2.53–2.61 (m, 1H, C*H*H), 2.10–1.99 (m, 1H, C*H*H), 1.96 (s, 3H, C*H*_3_), 0.86 (s, 9H, SiC(C*H*_3_)_3_), 0.19 (s, 3H, SiC*H*_3_), –0.08 (s, 3H, SiC*H*_3_) ppm. – ^13^C NMR (125 MHz, CDCl_3_): *δ* = 198.4 (C_q_, 1 × *C*=O), 196.4 (C_q_, 1 × *C*=O), 192.1 (C_q_, 1 × *C*=O), 141.4 (+, 1 × *C*H_Ar_), 141.0 (C_q_, 1 × *C*_qAr_), 135.5 (C_q_, 1 × *C*_qAr_), 135.3 (C_q_, 1 × *C*_q_), 134.9 (+, 1 × *C*H_Ar_), 134.4 (+, 1 × *C*H_Ar_), 133.6 (1 × CH=*C*_q_), 132.0 (+, 1 × *C*H_Ar_), 128.7 (+, 1 × *C*H_Ar_), 127.8 (+, 1 × *C*H_Ar_), 127.5 (+, 1 × *C*H_Ar_), 127.2 (+, 1 × C=*C*H), 122.5 (+, 1 × *C*H_Ar_), 94.7 (C_q_, 1 × *C*_qAr_), 74.4 (+, 1 × *C*H), 71.3 (C_q_, 1 × *C*_q_), 45.0 (+, 1 × *C*H), 28.2 (+, 3 × *C*H_3_), 26.1 (+, 1 × *C*H_3_), 23.0 (–, 1 × *C*H_2_), 18.7 (C_q_, 1 × *C*_q_), –3.5 (+, 1 × Si*C*H_3_), –3.8 (+, 1 × Si*C*H_3_) ppm. – IR (ATR): ṽ = 2924 (w), 2850 (w), 1697 (m), 1672 (w), 1593 (w), 1456 (w), 1423 (w), 1248 (m), 1220 (w), 1074 (m), 1050 (m), 1006 (w), 930 (w), 885 (w), 836 (m), 782 (m), 765 (m), 735 (m), 716 (w), 701 (w), 679 (w), 637 (w), 605 (w), 540 (w), 453 (w), 414 (w) cm^–1^. – MS (FAB, 70 eV), *m*/*z* (%): 587 (7) [M+H]^+^, 529 (16) [M–C(CH_3_)_3_]^+^, 455 (4)
[M–OTBDMS]^+^, 389 (11), 231 (100). – HRMS (EI, C_28_H_31_IO_4_Si): calc. 586.1109; found 586.1111. – X-Ray: The structure of **8af** could be confirmed by single-crystal X-Ray diffraction (see 4 Crystallographic Information, CCDC 1992184).
Additional reaction details and data obtained from the characterization of the target compound can be accessed at: <https://dx.doi.org/10.14272/reaction/SA-FUHFF-UHFFFADPSC-UMSRHQGWSN-UHFFFADPSC-NUHFF-NHVYL-NUHFF-ZZZ>.

**Cycloaddition products of 2‑(2‑bromobenzoyl)naphthalene-1,4-dione (5b) and (*E*)-*tert*-butyldimethyl((2-methylbuta-1,3-dien-1-yl)oxy)silane (6f)**

According to **GP C** the cycloaddition was performed with 2‑(2‑bromobenzoyl)naphthalene-1,4-dione (**5b**) (500 mg, 1.47 mmol, 1.00 equiv.) and (*E*)‑*tert*-butyldimethyl((2-methylbuta-1,3-dien-1-yl)oxy)silane (**6f**) (1.16 g, 5.86 mmol, 4.00 equiv.) in dry CH_2_Cl_2_ (7.0 mL). After 4 h the crude product was purified *via* flash chromatography on silica gel (cHex/EtOAc = 12:1) to obtain **9bf** (419 mg, 776 µmol, 53%) and **8bf** (215 mg, 399 µmol, 27%) both as yellow solids. The exact structure of isomer **9bf** could be verified by X-ray analysis while the exact structure of the other isolated isomer could not be resolved by analysis of the NMR spectra.

**9bf**: *R*_f_ (cHex/EtOAc = 12:1) = 0.34. − ^1^H NMR (500 MHz, CDCl_3_): *δ* = 8.16–8.09 (m, 2H, C*H*_Ar_), 8.04–8.00 (m, 1H, C*H*_Ar_), 7.74 (pd, ^3^*J* = 7.3 Hz, ^4^*J* = 1.6 Hz, 2H, C*H*_Ar_), 7.62 (dd, ^3^*J* = 8.0 Hz, ^4^*J* = 1.2 Hz, 1H, C*H*_Ar_), 7.39 (td, ^3^*J* = 7.6 Hz, ^4^*J* = 1.2 Hz, 1H, C*H*_Ar_), 7.28–7.23 (m, 1H, C*H*_Ar_), 5.62–5.58 (m, 1H, C=C*H*), 4.74 (s, 1H, C(OTBDMS)*H*), 3.97 (d, ^3^*J* = 8.0 Hz, 1H, COC*H*), 3.39–3.24 (m, 1H, C*H*H), 2.23–2.14 (m, 1H, C*H*H),
1.28–1.26 (m, 3H, C*H*_3_), 0.50 (s, 9H, SiC(C*H*_3_)_3_), –0.13 (s, 3H, SiC*H*_3_), –0.70 (s, 3H, SiC*H*_3_) ppm. − ^13^C NMR (126 MHz, CDCl_3_): *δ* = 198.2 (C_q_, 1 × *C*=O), 197.3 (C_q_, 1 × *C*=O), 195.1 (C_q_, 1 × *C*=O), 139.3 (C_q_, 1 × *C*_qAr_), 137.3 (C_q_, 1 × *C*_qAr_), 134.9 (C_q_, 1 × *C*_qAr_), 134.6 (+, 1 × *C*H_Ar_), 134.5 (+, 1 × *C*H_Ar_), 133.3 (+, 1 × *C*H_Ar_), 133.0 (C_q_, 1 × *C*_q_), 131.3 (+, 1 × *C*H_Ar_), 128.5 (+, 1 × *C*H_Ar_), 127.6 (+, 1 × *C*H_Ar_), 127.0 (+, 1 × C=*C*H), 126.5 (+, 1 × *C*H_Ar_), 126.3 (+, 1 × *C*H_Ar_), 121.4 (C_q_, 1 × *C*_qAr_), 73.6 (+, 1 × *C*(OTBDMS)H), 72.0 (C_q_, 1 × *C*_q_), 45.1 (+, 1 × CO*C*H), 25.4 (+, 3 × *C*H_3_), 22.2 (+, 1 × *C*H_3_), 20.9 (–, 1 × *C*H_2_), 18.1 (C_q_, 1 × *C*_q_), –4.8 (+, 1 × Si*C*H_3_),
–4.9 (+, 1 × Si*C*H_3_) ppm. – IR (ATR): ṽ = 1701 (vs), 1672 (s), 1594 (w), 1425 (w), 1252 (vs), 1220 (m), 1075 (vs), 1051 (vs), 1014 (m), 870 (m), 858 (m), 837 (vs), 806 (m), 783 (vs), 764 (vs), 751 (m), 737 (vs), 718 (s), 703 (s), 684 (s), 639 (w), 606 (m), 582 (w), 541 (m), 460 (m), 439 (w), 415 (m), 392 (m) cm^–1^. – MS (FAB, 3-NBA), *m*/*z*(%): 539/541 (13/11) [M+H]^+^, 281 (27), 207 (30), 185 (100). – HRMS (FAB, C_28_H_32_^79^BrO_4_Si): calc. 539.1253; found 539.1252. – X‑Ray: The structure of **9bf** could be confirmed by single crystal X‑Ray diffraction (see 4 Crystallographic Information, CCDC 1992888).
Additional reaction details and data obtained from the characterization of the target compound can be accessed at: <https://dx.doi.org/10.14272/reaction/SA-FUHFF-UHFFFADPSC-AYOHXYGZVK-UHFFFADPSC-NUHFF-NHVYL-NUHFF-ZZZ>.

**8/9bf**: *R*_f_ (cHex/EtOAc = 12:1) = 0.26. − ^1^H NMR (500 MHz, CDCl_3_): *δ* = 8.00 (dd, ^3^*J* = 7.7 Hz, ^4^*J* = 1.3 Hz, 1H, C*H*_Ar_), 7.87 (dd, ^3^*J* = 7.7 Hz, ^4^*J* = 1.3 Hz, 1H, C*H*_Ar_), 7.78 (dd, ^3^*J* = 7.8 Hz, ^4^*J* = 1.7 Hz, 1H, C*H*_Ar_), 7.72 (td, ^3^*J* = 7.6 Hz, ^4^*J* = 1.4 Hz, 1H, C*H*_Ar_), 7.65 (td, ^3^*J* = 7.5 Hz, ^4^*J* = 1.3 Hz, 1H, C*H*_Ar_), 7.41 (dd, ^3^*J* = 8.1 Hz, ^4^*J* = 1.2 Hz, 1H, C*H*_Ar_), 7.37 (td, ^3^*J* = 7.6 Hz, ^4^*J* = 1.2 Hz, 1H, C*H*_Ar_), 7.26–7.22 (m, 1H, C*H*_Ar_), 5.48–5.44 (m, 1H, C=C*H*), 5.31 (s, 1H, C(OTBDMS)*H*), 4.02 (dd, ^3^*J* = 10.7, 7.8 Hz, 1H, COC*H*), 2.63–2.56 (m, 1H, C*H*H),
2.08–2.00 (m, 1H, C*H*H), 1.98–1.95 (m, 3H, C*H*_3_), 0.87 (s, 9H, SiC(C*H*_3_)_3_), 0.18 (s, 3H, SiC*H*_3_), –0.02 (s, 3H, SiC*H*_3_) ppm. − ^13^C NMR (126 MHz, CDCl_3_): *δ* = 197.6 (C_q_, 1 × *C*=O), 196.4 (C_q_, 1 × *C*=O), 192.1 (C_q_, 1 × *C*=O), 138.0 (C_q_, 1 × *C*_qAr_), 135.3 (C_q_, 1 × *C*_qAr_), 135.2 (+, 1 × *C*H_Ar_), 134.9 (C_q_, 1 × *C*_qAr_), 134.3 (+, 1 × *C*H_Ar_), 134.2 (+, 1 × *C*H_Ar_), 133.5 (C_q_, 1 × *C*_q_), 131.9 (+, 1 × *C*H_Ar_), 128.8 (+, 1 × *C*H_Ar_), 127.6 (+, 1 × *C*H_Ar_), 127.3 (+, 1 × *C*H_Ar_), 126.6 (+, 1 × *C*H_Ar_), 122.7 (+, 1 × C=*C*H), 121.0 (C_q_, 1 × *C*_qAr_), 74.8 (C_q_, 1 × *C*_q_), 71.0 (+, 1 × *C*(OTBDMS)H), 44.6 (+, 1 × CO*C*H), 28.1 (–, 1 × *C*H_2_), 26.1 (+, 3 × *C*H_3_), 23.0 (+, 1 × *C*H_3_), 18.8 (C_q_, 1 × *C*_q_), –3.5 (+, 1 × Si*C*H_3_), –3.8 (+, 1 × Si*C*H_3_) ppm. – IR (ATR): ṽ = 2925 (w), 2851 (w), 1708 (s), 1693 (vs), 1591 (m), 1462 (w), 1428 (w), 1265 (s), 1231 (vs), 1071 (vs), 1057 (vs), 1006 (s), 953 (m), 932 (m), 888 (vs), 858 (m), 834 (vs), 810 (vs), 768 (vs), 722 (s), 673 (s), 646 (m), 602 (s), 568 (m), 533 (m), 395 (w) cm^–1^. – MS (FAB, 3-NBA), *m*/*z*(%): 539/541 (7/6) [M+H]^+^, 483 (15), 281 (19), 185 (100). – HRMS (FAB, C_28_H_32_^79^BrO_4_Si): calc. 539.1253; found 539.1252.
Additional reaction details and data obtained from the characterization of the target compound can be accessed at: <https://dx.doi.org/10.14272/reaction/SA-FUHFF-UHFFFADPSC-AYOHXYGZVK-UHFFFADPSC-NUHFF-NHVYL-NUHFF-ZZZ>.

**(4a*R*,9a*R*)-4a-(2-Iodobenzoyl)-2-((triisopropylsilyl)oxy)-1,4,4a,9a-tetrahydroanthracene-9,10-dione (8ag)**

According to **GP C** the cycloaddition was performed with 2‑(2‑iodobenzoyl)naphthalene-1,4-dione (**5a**) (233 mg, 600 µmol, 1.00 equiv.) and (buta-1,3-dien-2-yloxy)triisopropylsilane (**6g**) (0.33 mL, 272 mg, 1.20 mmol, 2.00 equiv.) in dry CH_2_Cl_2_ (3 mL) at 40 °C. After 3.5 h the crude product was purified *via* flash chromatography on silica gel (cHex/EtOAc = 10:1) to obtain product **8ag** as a yellow solid (274 mg, 446 µmol, 75%). – *R*_f_ (cylohexane/EtOAc = 14:1) = 0.29. – ^1^H NMR (400 MHz, CDCl_3_): *δ* = 8.17 (dd, ^3^*J* = 7.5 Hz, ^4^*J* = 1.6 Hz, 1H, C*H*_Ar_), 8.03 (dd, ^3^*J* = 7.5 Hz, ^4^*J* = 1.6 Hz, 1H, C*H*_Ar_), 7.84 (dd, ^3^*J* = 7.9 Hz, ^4^*J* = 1.1 Hz, 1H, C*H*_Ar_), 7.82–7.68 (m, 2H, C*H*_Ar_), 7.33–7.26 (m, 1H, C*H*_Ar_), 7.14 (dd, ^3^*J* = 7.8 Hz, ^4^*J* = 1.7 Hz, 1H, C*H*_Ar_), 7.06 (td, ^3^*J* = 7.7 Hz, ^4^*J* = 1.7 Hz, 1H, C*H*_Ar_), 4.83–4.71 (m, 1H, C=C*H*), 3.80 (dd, ^3^*J* = 9.8, 6.6 Hz, 1H, C*H*), 3.03–2.96 (m, 1H, C*H*H), 2.56–2.22 (m, 3H, CH*H*, C*H*_2_), 1.15–0.93 (m, 21H, 3×C*H*, 6×C*H*_3_) ppm. – ^13^C NMR (101 MHz, CDCl_3_): *δ* = 200.7 (C_q_, 1 × C=O), 195.4 (C_q_, 1 × C=O), 193.8 (C_q_, 1 × C=O), 148.0 (C_q_, 1 × C_qAr_), 142.7 (C_q_, 1 × C_qAr_), 140.8 (+, 1 × CH_Ar_), 135.3 (+, 1 × CH_Ar_), 134.3 (+, 1 × CH_Ar_), 134.2 (C_q_, 1 × C_qAr_), 133.1 (C_q_, 1 × C_q_), 131.6 (+, 1 × CH_Ar_), 127.4 (+, 1 × CH_Ar_), 127.4 (+, 1 × CH_Ar_), 127.3 (+, 1 × CH_Ar_), 126.9 (+, 1 × CH_Ar_), 99.4 (+, 1 × C=CH), 93.0 (C_q_, 1 × C_qAr_), 67.9 (C_q_, 1 × C_q_), 50.7 (+, 1 × CH), 30.3 (–, 1 × CH_2_), 27.8 (–, 1 × CH_2_), 18.0 (+, 6 × CH_3_), 12.6 (+, 3 × SiCH) ppm. – IR (ATR): ṽ = 2890 (w), 2862 (w), 1689 (w), 1668 (m), 1590 (w), 1460 (w), 1376 (w), 1287 (w), 1222 (m), 1201 (m), 1061 (w), 1014 (w), 881 (w), 853 (m), 797 (w), 767 (w), 753 (w), 736 (m), 692 (m), 633 (m), 501 (w), 431 (w), 402 (w) cm^–1^. – MS (FAB, 3-NBA), *m*/*z*(%): 614 (10) [M]^+^, 615 (31) [M+H]^+^, 231 (100) [M−C_23_H_31_O_3_Si]^+^, 383 (99) [M−C_7_H_4_IO]^+^. – HRMS (FAB, C_30_H_36_^127^IO_4_Si): calc. 615.1422; found 615.1424. – X‑Ray: The structure of **8ag** could be confirmed by single crystal X‑Ray diffraction (see 4 Crystallographic Information, CCDC 1992889).
Additional reaction details and data obtained from the characterization of the target compound can be accessed at: <https://dx.doi.org/10.14272/reaction/SA-FUHFF-UHFFFADPSC-GYGDJDFWRC-UHFFFADPSC-NUHFF-NXQUO-NUHFF-ZZZ>.

**(4a*R*,9a*R*)-2-((*tert*-Butyldiphenylsilyl)oxy)-4a-(2-iodobenzoyl)-1,4,4a,9a-tetrahydroanthracene-9,10-dione (8ah)**

According to **GP C** the cycloaddition was performed with 2‑(2‑iodobenzoyl)naphthalene-1,4-dione (**5a**) (311 mg, 800 µmol, 1.00 equiv.) and (buta-1,3-dien-2-yloxy)(*tert*-butyl)diphenylsilane (**6h**) (1.3 mL, 1.23 g, 4.00 mmol, 5.00 equiv.) in dry CH_2_Cl_2_ (4.0 mL). After 5 h the crude product was purified *via* flash chromatography on silica gel (cHex/EtOAc = 9:1) to obtain product **8ah** as a yellow solid (401 mg, 576 mmol, 72%). − *R*_f_ (cHex/EtOAc = 8:1) = 0.34. − ^1^H NMR (400 MHz, CDCl_3_): *δ* = 8.17−8.11 (m, 1H, C*H*_Ar_), 8.05−7.99 (m, 1H, C*H*_Ar_), 7.84 (d, ^3^*J* = 7.9 Hz, 1H, C*H*_Ar_), 7.82−7.68 (m, 3H, C*H*_Ar_), 7.68−7.58 (m, 3H, C*H*_Ar_), 7.41−7.32 (m, 6H, C*H*_Ar_), 7.24−7.22 (m, 1H, C*H*_Ar_), 7.10 (dd, ^3^*J* = 7.8 Hz, ^4^*J* = 1.6 Hz, 1H, C*H*_Ar_), 7.04 (td, ^3^*J* = 7.7 Hz, ^4^*J* = 1.6 Hz, 1H, C*H*_Ar_), 4.60−4.50 (m, 1H, C=C*H*), 3.76 (dd, ^3^*J* = 9.4, 6.6 Hz, 1H, C*H*), 2.77 (dd, ^2^*J* = 16.5 Hz, ^3^*J* = 4.8 Hz, 1H, C*H*H), 2.56−2.44 (m, 1H, CH*H*), 2.40−2.22 (m, 2H, C*H_2_*), 0.99 (s, 9H, 3 × C*H_3_*) ppm. − ^13^C NMR (101 MHz, CDCl_3_): *δ* = 200.8 (C_q_, 1 × *C*=O), 195.2 (C_q_, 1 × *C*=O), 193.7 (C_q_, 1 × *C*=O), 147.7 (C_q_, 1 × *C*_qAr_), 142.5 (+, 1 × *C*H_Ar_), 140.8 (+, 1 × *C*H_Ar_), 135.5 (+, 3 × *C*H_Ar_), 135.2 (C_q_, 1 × *C*_qAr_), 134.9 (+, 1 × *C*H_Ar_), 134.2 (C_q_, 1 × *C*_qAr_), 134.2 (C_q_, 1 × *C*_qAr_), 133.2 (C_q_, 1 × *C*_qAr_), 133.0 (C_q_, 1 × *C*_qAr_), 131.5 (+, 1 × *C*H_Ar_), 129.9 (+, 2 × *C*H_Ar_), 129.8 (C_q_, 1 × *C*_q_), 127.9 (+, 1 × *C*H_Ar_), 127.8 (+, 2 × *C*H_Ar_), 127.8 (+, 2 × *C*H_Ar_), 127.5 (+, 1 × *C*H_Ar_), 127.4 (+, 1 × *C*H_Ar_), 127.2 (+, 1 × *C*H_Ar_), 126.9 (+, 1 × *C*H_Ar_), 101.4 (+, 1 × C=*C*H), 67.6 (C_q_, 1 × *C*_q_), 50.4 (+, 1 × *C*H), 30.0 (–, 1 × *C*H_2_), 27.7 (–, 1 × *C*H_2_), 26.6 (+, 3 × *C*H_3_), 19.3 (C_q_, 1 × *C*_q_) ppm. – IR (ATR): ṽ = 2890 (w), 2862 (w), 1689 (w), 1668 (m), 1590 (w), 1460 (w), 1376 (w), 1287 (w), 1222 (m), 1201 (m), 1061 (w), 1014 (w), 881 (w), 853 (m), 797 (w), 767 (w), 753 (w), 736 (m), 692 (m), 633 (m), 501 (w), 431 (w), 402 (w) cm^–1^. – MS (FAB, 3-NBA), *m*/*z* (%): 697 (20) [M+H]^+^, 696 (11) [M]^+^, 466 (28), 465 (67), 231 (79), 197 (46), 135 (100). – HRMS (FAB, C_37_H_34_O_4_^127^ISi): calc. 697.1271; found 697.1272.
Additional reaction details and data obtained from the characterization of the target compound can be accessed at: <https://dx.doi.org/10.14272/reaction/SA-FUHFF-UHFFFADPSC-VGCVCLYBTD-UHFFFADPSC-NUHFF-NLYQR-NUHFF-ZZZ>.

**(4a*S*,9a*S*)-4a-(2-Bromobenzoyl)-2-((*tert*-butyldiphenylsilyl)oxy)-1,4,4a,9a-tetrahydroanthracene-9,10-dione (8bh)**

According to **GP C** the cycloaddition was performed with 2‑(2‑bromo­benzoyl)­naphthalene-1,4-dione (**5b**) (273 mg, 800 µmol, 1.00 equiv.) and (buta-1,3-dien-2-yloxy)(*tert*-butyl)diphenylsilane (**6h**) (1.3 mL, 1.23 g, 4.00 mmol, 5.00 equiv.) in dry CH_2_Cl_2_ (4.0 mL). After 5 h the crude product was purified *via* flash chromatography on silica gel (cHex/EtOAc = 9:1) to obtain product **8bh** as a yellow solid (411 mg, 632 mmol, 79%). − *R*_f_ (cHex/EtOAc = 9:1) = 0.37. − ^1^H NMR (500 MHz, CDCl_3_): *δ* = 8.13 (dd, ^3^*J* = 7.5 Hz, ^4^*J* = 1.4 Hz, 1H, C*H*_Ar_), 8.02 (dd, ^3^*J* = 7.5 Hz, ^4^*J* = 1.4 Hz, 1H, C*H*_Ar_), 7.80−7.72 (m, 2H, C*H*_Ar_), 7.65−7.61 (m, 4H, C*H*_Ar_), 7.57−7.53 (m, 1H, C*H*_Ar_), 7.40−7.31 (m, 6H, C*H*_Ar_), 7.21 (dd, ^3^*J* = 5.8 Hz, ^4^*J* = 3.5 Hz, 2H, C*H*_Ar_), 7.14 (dd, ^3^*J* = 5.8 Hz, ^4^*J* = 3.5 Hz, 1H, C*H*_Ar_), 4.52−4.50 (m, 1H, C=C*H*), 3.78 (dd, ^3^*J* = 8.8, 6.6 Hz, 1H, C*H*), 2.76−2.71 (m, 1H, C*H*H), 2.52−2.47 (m, 1H, C*H*H), 2.41−2.31 (m, 2H, CH*H*, CH*H*), 0.99 (s, 9H, 3 × C*H*_3_) ppm. − ^13^C NMR (126 MHz, CDCl_3_): *δ* = 200.0 (C_q_, 1 × *C*=O), 195.2 (C_q_, 1 × *C*=O), 193.7 (C_q_, 1 × *C*=O), 147.8 (C_q_, 1 × *C*_qAr_), 138.9 (C_q_, 1 × *C*_qAr_), 135.4 (+, 2 × *C*H_Ar_), 135.4 (+, 2 × *C*H_Ar_), 135.1 (+, 1 × *C*H_Ar_), 134.2 (+, 1 × *C*H_Ar_), 134.0 (C_q_, 1 × *C*_qAr_), 133.8 (+, 1 × *C*H_Ar_), 133.0 (C_q_, 1 × *C*_qAr_), 132.8 (C_q_, 1 × *C*_qAr_), 132.5 (C_q_, 1 × *C*_q_), 131.3 (+, 1 × *C*H_Ar_), 129.8 (+, 2 × *C*H_Ar_), 127.7 (+, 2 × *C*H_Ar_), 127.7 (+, 2 × *C*H_Ar_), 127.4 (+, 1 × *C*H_Ar_), 127.3 (+, 1 × *C*H_Ar_), 127.1 (+, 1 × *C*H_Ar_), 126.6 (+, 1 × *C*H_Ar_), 119.8 (C_q_, 1 × *C*_qAr_), 101.0 (+, 1 × C=*C*H), 67.5 (C_q_, 1 × *C*_q_), 50.0 (+, 1 × *C*H), 29.5 (–, 1 × *C*H_2_), 27.4 (–, 1 × *C*H_2_), 26.4 (+, 3 × *C*H_3_), 19.2 (C_q_, 1 × *C*_q_) ppm. – IR (ATR): ṽ = 3070 (vw), 2930 (vw), 2856 (vw), 1679 (w), 1591 (w), 1470 (vw), 1427 (w), 1372 (vw), 1255 (w), 1241 (w), 1219 (w), 1194 (w), 1111 (w), 1062 (w), 1019 (w), 939 (w), 893 (vw), 851 (w), 821 (w), 796 (vw), 761 (vw), 736 (w), 699 (m), 646 (w), 612 (w), 550 (vw), 492 (w) cm^–1^. – MS (FAB, 3‑NBA), *m*/*z* (%): 649/651 (20/20) [M+H]^+^, 648/650 (7/14) [M]^+^, 466 (41), 465 (100), 185 (48), 183 (51). – HRMS (FAB, C_37_H_34_O_4_^79^BrSi): calc. 649.1410; found 649.1408. – X‑Ray: The structure of **8bh** could be confirmed by single crystal X‑Ray diffraction (see 4 Crystallographic Information, CCDC 1992185).
Additional reaction details and data obtained from the characterization of the target compound can be accessed at: <https://dx.doi.org/10.14272/reaction/SA-FUHFF-UHFFFADPSC-FOIXCRBFVL-UHFFFADPSC-NUHFF-NLYQR-NUHFF-ZZZ>.

**Cycloaddition product of 2‑(2‑bromo-5-methoxybenzoyl)naphthalene-1,4-dione (5c) and isoprene (6b) (8/9bb)**

According to **GP C** the cycloaddition was performed with 2‑(2‑bromo-5-methoxybenzoyl)naphthalene-1,4-dione (**5c**) (148 mg, 400 µmol, 1.00 equiv.) and isoprene (**6b**) (0.20 mL, 136 mg, 2.00 mmol, 5.00 equiv.) in dry CH_2_Cl_2_ (2.0 mL). After 3 h the crude product was purified *via* flash chromatography on silica gel (cHex/EtOAc = 6:1) to obtain a light yellow solid (89.6 mg, 204 µmol, 51%). The products **8/9bb** were isolated as a non-separable mixture in in a ∼ 10 : 1 ratio as estimated by ^1^H NMR. Both possible regioisomers are drawn, as the exact structure of the products could not be resolved by analysis of the NMR spectra. Below the analytics of the major product are given. − *R*_f_ (cHex/EtOAc = 6:1) = 0.27. − ^1^H NMR (500 MHz, CDCl_3_): *δ* = 8.13 (dd, ^3^*J* = 7.6 Hz, ^4^*J* = 1.5 Hz, 1H, C*H*_Ar_), 8.02 (dd, ^3^*J* = 7.6 Hz, ^4^*J* = 1.4 Hz, 1H, C*H*_Ar_), 7.80–7.70 (m, 2H, C*H*_Ar_), 7.41 (d, ^3^*J* = 8.8 Hz, 1H, C*H*_Ar_), 6.78 (dd, ^3^*J* = 8.8 Hz, ^4^*J* = 3.0 Hz, 1H, C*H*_Ar_), 6.72 (d, ^4^*J* = 3.0 Hz, 1H, C*H*_Ar_), 5.33–5.31 (m, 1H, C=C*H*), 3.75–3.72 (m, 4H, C*H*, OC*H*_3_), 2.92–2.87 (m, 1H, C*H*H), 2.51–2.45 (m, 1H, CH*H*), 2.30–2.23 (m, 2H, C*H*_2_), 1.63 (d, ^4^*J* = 1.9 Hz, 3H, C*H*_3_) ppm. − ^13^C NMR (126 MHz, CDCl_3_): *δ* = 199.9 (C_q_, 1 × *C*=O), 196.2 (C_q_, 1 × *C*=O), 194.5 (C_q_, 1 × *C*=O), 158.1 (C_q_, 1 × *C*_qAr_), 139.7 (C_q_, 1 × *C*_qAr_), 135.2 (+, 1 × *C*H_Ar_), 134.7 (+, 1 × *C*H_Ar_), 134.3 (+, 1 × *C*H_Ar_), 134.2 (C_q_, 1 × *C*_qAr_), 133.1 (C_q_, 1 × *C*_qAr_), 132.2 (C_q_, 1 × *C*_qAr_), 127.3 (+, 1 × *C*H_Ar_), 127.1 (+, 1 × *C*H_Ar_), 117.8 (+, 1 × C=*C*H), 117.2 (+, 1 × *C*H_Ar_), 113.3 (+, 1 × *C*H_Ar_), 110.0 (C_q_, 1 × *C*_q_), 67.7 (C_q_, 1 × *C*_q_), 55.7 (+, 1 × O*C*H_3_), 49.9 (+, 1 × *C*H), 30.2 (–, 1 × *C*H_2_), 28.6 (–, 1 × *C*H_2_), 23.1 (+, 1 × *C*H_3_) ppm. – IR (ATR): ṽ = 2932 (w), 2836 (w), 1679 (vs), 1591 (s), 1568 (s), 1465 (s), 1275 (vs), 1242 (vs), 1200 (vs), 1176 (vs), 1065 (m), 1023 (vs), 969 (m), 936 (s), 807 (vs), 739 (vs), 687 (m), 599 (s), 561 (m), 483 (w), 431 (m) cm^–1^. – MS (FAB, 3‑NBA), *m*/*z* (%): 439/441 (9/8) [M+H]^+^, 225 (41), 215 (100). – HRMS (FAB, C_23_H_20_^79^BrO_4_Si): calc. 439.0545; found 439.0544.
Additional reaction details and data obtained from the characterization of the target compound can be accessed at: <https://dx.doi.org/10.14272/reaction/SA-FUHFF-UHFFFADPSC-XQBNRFKTLB-UHFFFADPSC-NUHFF-NUGKD-NUHFF-ZZZ>.

**(4a*R*,9a*R*)-4a-(2-Bromo-5-methoxybenzoyl)-2,3-dimethyl-1,4,4a,9a-tetrahydroanthracene-9,10-dione (8cc)**

According to **GP C** the cycloaddition was performed with 2‑(2‑bromo-5-methoxybenzoyl)naphthalene-1,4-dione (**5c**) (74.2 mg, 200 µmol, 1.00 equiv.) and 2,3-dimethylbuta-1,3-diene (**6c**) (0.12 mL, 82.2 mg, 1.00 mmol, 5.00 equiv.) in dry CH_2_Cl_2_ (5.0 mL). After 3 h the crude product was purified *via* flash chromatography on silica gel (cHex/EtOAc = 6:1) to obtain product **8cc** as a yellow solid (58.9 mg, 130 µmol, 65%). − *R*_f_ (cHex/EtOAc = 6:1) = 0.43. − ^1^H NMR (500 MHz, CDCl_3_): *δ* = 8.17–8.11 (m, 1H, C*H*_Ar_), 8.07–8.01 (m, 1H, C*H*_Ar_), 7.78 (td, ^3^*J* = 7.5 Hz, ^4^*J* = 1.5 Hz, 1H, C*H*_Ar_), 7.74 (td, ^3^*J* = 7.5 Hz, ^4^*J* = 1.5 Hz, 1H, C*H*_Ar_), 7.47–7.41 (m, 1H, C*H*_Ar_), 6.83–6.74 (m, 2H, C*H*_Ar_), 3.77 (s, 3H, OC*H*_3_), 3.72 (dd, ^3^*J* = 8.4, 6.7 Hz, 1H, C*H*), 2.86–2.74 (m, 1H, C*H*H), 2.41 (d, ^2^*J* = 16.7 Hz, 1H, CH*H*), 2.35–2.23 (m, 2H, C*H*_2_), 1.59 (s, 3H, C*H*_3_), 1.54 (s, 3H, C*H*_3_) ppm. − ^13^C NMR (126 MHz, CDCl_3_): *δ* = 200.0 (C_q_, 1 × *C*=O), 196.5 (C_q_, 1 × *C*=O), 194.8 (C_q_, 1 × *C*=O), 158.1 (C_q_, 1 × *C*_qAr_), 139.8 (C_q_, 1 × *C*_qAr_), 135.2 (+, 1 × *C*H_Ar_), 134.7 (+, 1 × *C*H_Ar_), 134.3 (C_q_, 1 × *C*_qAr_), 134.3 (+, 1 × *C*H_Ar_), 133.2 (C_q_, 1 × *C*_qAr_), 127.3 (+, 1 × *C*H_Ar_), 127.2 (+, 1 × *C*H_Ar_), 124.2 (C_q_, 1 × *C*_qAr_), 122.9 (C_q_, 1 × *C*_q_), 117.1 (+, 1 × *C*H_Ar_), 113.4 (+, 1 × *C*H_Ar_), 110.1 (C_q_, 1 × *C*_q_), 68.6 (C_q_, 1 × *C*_q_), 55.7 (+, 1 × O*C*H_3_), 50.2 (+, 1 × *C*H), 34.3 (–, 1 × *C*H_2_), 31.6 (–, 1 × *C*H_2_), 19.1 (+, 1 × *C*H_3_), 18.8 (+, 1 × *C*H_3_) ppm. – IR (ATR): ṽ = 3070 (w), 3003 (w), 2912 (w), 2837 (w), 1681 (vs), 1591 (vs), 1568 (s), 1463 (s), 1442 (m), 1402 (w), 1390 (w), 1310 (m), 1276 (vs), 1242 (vs), 1220 (s), 1201 (vs), 1176 (s), 1123 (w), 1089 (w), 1062 (m), 1021 (vs), 1009 (s), 976 (w), 932 (w), 914 (w), 849 (w), 823 (m), 779 (m), 751 (vs), 728 (vs), 715 (s), 683 (w), 667 (m), 647 (w), 601 (m), 560 (w), 540 (w), 477 (w), 439 (w), 391 (w) cm^–1^. – MS (FAB, 3-NBA), *m*/*z* (%): 453/455 (20/17) [M+H]^+^, 239 (80), 215 (98) [C_8_H_8_BrO_2_]^+^, 213 (100). – HRMS (FAB, C_24_H_22_O_4_^79^Br): calc. 453.0701; found 453.0702.
Additional reaction details and data obtained from the characterization of the target compound can be accessed at: <https://dx.doi.org/10.14272/reaction/SA-FUHFF-UHFFFADPSC-UKTGPOJWCW-UHFFFADPSC-NUHFF-NUHFF-NUHFF-ZZZ>.

**Cycloaddition products of 2‑(2‑bromo-5-methoxybenzoyl)naphthalene-1,4-dione (5c) and 1-(trimethylsiloxy)-1,3-butadiene (6d) (8/9cd)**

According to **GP** **C** the cycloaddition was performed with 2‑(2‑bromo-5-methoxybenzoyl)naphthalene-1,4-dione (**5c**) (260 mg, 700 µmol, 1.00 equiv.) and 1‑(trimethylsiloxy)-1,3-butadiene (**6d**) (0.61 mL, 498 mg, 3.50 mmol, 5.00 equiv.) in dry CH_2_Cl_2_ (4.0 mL). After 4 h the crude product was purified *via* flash chromatography on silica gel (cHex/EtOAc = 6:1) to obtain isomers **8/9cd** (ratio ∼ 5.6 : 1) as a yellow solid (total yield: 226 mg, 441 µmol, 63%). Both possible regioisomers are drawn, as the exact structure of the products could not be resolved by analysis of the NMR spectra.

**8/9cd F1**: *R*_f_ (cHex/EtOAc = 6:1) = 0.34. − ^1^H NMR (500 MHz, CDCl_3_): *δ* = 8.18–8.12 (m, 1H, C*H*_Ar_), 8.06–8.00 (m, 1H, C*H*_Ar_), 7.73 (ddd, ^3^*J* = 6.9, 6.1 Hz, ^4^*J* = 1.7 Hz, 2H, C*H*_Ar_), 7.48 (d, ^3^*J* = 8.8 Hz, 1H, C*H*_Ar_), 7.42 (d, ^4^*J* = 3.0 Hz, 1H, C*H*_Ar_), 6.82 (dd, ^3^*J* = 8.8 Hz, ^4^*J* = 3.0 Hz, 1H, C*H*_Ar_), 5.91 (ddd, ^3^*J* = 10.1, 4.5 Hz, ^4^*J* = 2.8 Hz, 1H, C=C*H*), 5.66 (ddt, ^3^*J* = 10.0, 5.0 Hz, ^4^*J* = 2.3 Hz, 1H, C=C*H*), 4.94 (d, ^3^*J* = 5.2 Hz, 1H, C*H*), 3.93 (d, ^3^*J* = 7.1 Hz, 1H, C*H*), 3.76 (s, 3H, OC*H*_3_), 3.20 (dddd, ^2^*J* = 19.4 Hz, ^3^*J* = 4.6 Hz, ^4^*J* = 2.2, 1.1 Hz, 1H, C*H*H), 2.32–2.12 (m, 1H, CH*H*), –0.34 (s, 9H, Si(C*H*_3_)_3_) ppm. − ^13^C NMR (126 MHz, CDCl_3_): *δ* = 197.8 (C_q_, 1 × *C*=O), 196.7 (C_q_, 1 × *C*=O), 194.9 (C_q_, 1 × *C*=O), 158.2 (C_q_, 1 × *C*_qAr_), 139.3 (C_q_, 1 × *C*_qAr_), 137.5 (C_q_, 1 × *C*_qAr_), 135.3 (+, 1 × *C*H_Ar_), 135.1 (C_q_, 1 × *C*_qAr_), 134.4 (+, 1 × *C*H_Ar_), 133.3 (+, 1 × *C*H_Ar_), 130.3 (+, 1 × =*C*H), 126.8 (+, 1 × *C*H_Ar_), 126.4 (+, 1 × =*C*H), 125.8 (+, 1 × *C*H_Ar_), 117.8 (+, 1 × *C*H_Ar_), 113.8 (+, 1 × *C*H_Ar_), 111.3 (C_q_, 1 × *C*_qAr_), 70.3 (C_q_, 1 × *C*_q_), 68.2 (+, 1 × *C*H), 55.7 (+, 1 × O*C*H_3_), 45.8 (+, 1 × *C*H), 21.1 (–, 1 × *C*H_2_), –0.6 (+, 3 × Si*C*H_3_) ppm. – IR (ATR): ṽ = 2956 (w), 1701 (vs), 1673 (s), 1591 (m), 1568 (w), 1463 (m), 1421 (w), 1391 (w), 1309 (w), 1273 (s), 1247 (vs), 1218 (s), 1194 (s), 1176 (m), 1077 (vs), 1043 (vs), 1017 (s), 969 (w), 943 (m), 864 (vs), 840 (vs), 783 (s), 751 (vs), 737 (s), 722 (s), 693 (vs), 601 (m), 472 (w), 438 (w), 380 (w) cm^–1^. – MS (FAB, 3-NBA), *m*/*z* (%): 513/515 (17/16) [M+H]^+^, 371/373 (27/27), 299 (56), 213/215 (100/98). – HRMS (FAB, C_25_H_26_O_5_^79^BrSi): calc. 513.0733; found 513.0735.
Additional reaction details and data obtained from the characterization of the target compound can be accessed at: <https://dx.doi.org/10.14272/reaction/SA-FUHFF-UHFFFADPSC-TWNQMZQUUQ-UHFFFADPSC-NUHFF-NNMJJ-NUHFF-ZZZ>.

**8/9cd F2**: *R*_f_ (cHex/EtOAc = 6:1) = 0.24. − ^1^H NMR (500 MHz, CDCl_3_): *δ* = 8.10–8.03 (m, 1H, C*H*_Ar_), 7.97–7.90 (m, 1H, C*H*_Ar_), 7.74 (td, ^3^*J* = 7.5 Hz, ^4^*J* = 1.3 Hz, 1H, C*H*_Ar_), 7.66 (td, ^3^*J* = 7.5 Hz, ^4^*J* = 1.3 Hz, 1H, C*H*_Ar_), 7.42–7.34 (m, 2H, C*H*_Ar_), 6.81 (dd, ^3^*J* = 8.8 Hz, ^4^*J* = 3.0 Hz, 1H, C*H*_Ar_), 6.02 (dddd, ^3^*J* = 9.8, 5.3 Hz, ^4^*J* = 2.6, 1.8 Hz, 1H, C=C*H*), 5.83 (ddd, ^3^*J* = 10.0, 4.8 Hz, ^4^*J* = 2.4 Hz, 1H, C=C*H*), 5.44–5.37 (m, 1H, C*H*), 3.87 (dd, ^3^*J* = 11.8, 6.6 Hz, 1H, C*H*), 3.83 (s, 3H, OC*H*_3_), 2.54 (dddd, ^2^*J* = 18.9 Hz, ^3^*J* = 6.6, 4.8 Hz, ^4^*J* = 1.8 Hz, 1H, C*H*H), 2.11–2.01 (m, 1H, C*H*H), 0.02 (s, 9H, Si(C*H*_3_)_3_) ppm. − ^13^C NMR (126 MHz, CDCl_3_): *δ* = 196.4 (C_q_, 1 × *C*=O), 195.8 (C_q_, 1 × *C*=O), 193.2 (C_q_, 1 × *C*=O), 158.4 (C_q_, 1 × *C*_qAr_), 138.5 (C_q_, 1 × *C*_qAr_), 135.4 (+, 1 × *C*H_Ar_), 135.3 (+, 1 × *C*H_Ar_), 134.6 (C_q_, 1 × *C*_qAr_), 134.2 (+, 1 × *C*H_Ar_), 133.9 (C_q_, 1 × *C*_qAr_), 127.7 (+, 1 × C=*C*H), 127.4 (+, 1 × *C*H_Ar_), 127.2 (+, 1 × *C*H_Ar_), 127.1 (+, 1 × C=*C*H), 117.9 (+, 1 × *C*H_Ar_), 114.5 (+, 1 × *C*H_Ar_), 111.8 (C_q_, 1 × *C*_qAr_), 73.8 (C_q_, 1 × *C*_q_), 66.1 (+, 1 × *C*H), 55.9 (+, 1 × O*C*H_3_), 46.1 (+, 1 × *C*H), 28.1 (–, 1 × *C*H_2_), 0.7 (+, 3 × Si*C*H_3_) ppm. – IR (ATR): ṽ = 2955 (w), 1698 (s), 1677 (vs), 1592 (m), 1568 (m), 1463 (m), 1402 (w), 1288 (s), 1266 (vs), 1244 (vs), 1218 (vs), 1197 (s), 1174 (s), 1072 (s), 1051 (vs), 1017 (s), 943 (s), 894 (vs), 839 (vs), 782 (s), 742 (vs), 714 (s), 686 (s), 585 (s), 492 (m) cm^–1^. – MS (FAB, 3‑NBA), *m*/*z* (%): 513/515 (9/8) [M+H]^+^, 371/373 (27/27), 213/215 (100/97). – HRMS (FAB, C_25_H_26_O_5_^79^BrSi): calc. 513.0733; found 513.0731.
Additional reaction details and data obtained from the characterization of the target compound can be accessed at: <https://dx.doi.org/10.14272/reaction/SA-FUHFF-UHFFFADPSC-TWNQMZQUUQ-UHFFFADPSC-NUHFF-NNMJJ-NUHFF-ZZZ>.

**Cycloaddition product of 2‑(2‑bromo-4,5-dimethoxybenzoyl)naphthalene-1,4-dione (5d) and isoprene (6b) (8/9db)**

According to **GP C** the cycloaddition was performed with 2‑(2‑bromo-4,5-dimethoxybenzoyl)naphthalene-1,4-dione (**5d**) (120 mg, 300 µmol, 1.00 equiv.) and isoprene (**6b**) (0.15 mL, 102 mg, 1.50 mmol, 5.00 equiv.) in dry CH_2_Cl_2_ (1.0 mL). After 4 h the crude product was purified *via* flash chromatography on silica gel (cHex/EtOAc = 6:1) to obtain a yellow solid (98.6 mg, 210 µmol, 70%). The products **8/9db** were isolated as a non-separable mixture in a ∼ 6.7 : 1 ratio as estimated by ^1^H NMR. Both possible regioisomers are drawn, as the exact structure of the products could not be resolved by analysis of the NMR spectra. Below the analytics of the major product are given. − *R*_f_ (cHex/EtOAc = 6:1) = 0.33. − ^1^H NMR (400 MHz, CDCl_3_): *δ* = 8.15 (dd, ^3^*J* = 7.6 Hz, ^4^*J* = 1.4 Hz, 1H, C*H*_Ar_), 8.00 (dd, ^3^*J* = 7.5 Hz, ^4^*J* = 1.4 Hz, 1H, C*H*_Ar_), 7.78 (td, ^3^*J* = 7.6 Hz, ^4^*J* = 1.6 Hz, 1H, C*H*_Ar_), 7.72 (td, ^3^*J* = 7.6 Hz, ^4^*J* = 1.6 Hz, 1H, C*H*_Ar_), 6.99 (s, 1H, C*H*_Ar_), 6.88 (s, 1H, C*H*_Ar_), 5.36−5.33 (m, 1H, C=C*H*), 3.87 (s, 3H, OC*H*_3_), 3.83 (s, 3H, OC*H*_3_), 3.75 (t, ^3^*J* = 7.7 Hz, 1H, C*H*), 3.01−2.95 (m, 1H, C*H*H), 2.47−2.39 (m, 1H, CH*H*), 2.27 (d, ^3^*J* = 7.5 Hz, 2H, C*H*_2_), 1.65 (s, 3H, C*H*_3_) ppm. − ^13^C NMR (101 MHz, CDCl_3_): *δ* = 198.8 (C_q_, 1 × *C*=O), 196.3 (C_q_, 1 × *C*=O), 195.5 (C_q_, 1 × *C*=O), 151.0 (C_q_, 1 × *C*_qAr_), 147.5 (C_q_, 1 × *C*_qAr_), 135.3 (+, 1 × *C*H_Ar_), 134.3 (+, 1 × *C*H_Ar_), 134.2 (C_q_, 1 × *C*_qAr_), 133.3 (C_q_, 1 × *C*_qAr_), 132.3 (C_q_, 1 × *C*_qAr_), 130.8 (C_q_, 1 × *C*_q_), 127.2 (+, 1 × *C*H_Ar_), 127.2 (+, 1 × *C*H_Ar_), 117.8 (+, 1 × C=*C*H), 116.7 (+, 1 × *C*H_Ar_), 111.9 (C_q_, 1 × *C*_qAr_), 110.9 (+, 1 × *C*H_Ar_), 67.9 (C_q_, 1 × *C*_q_), 56.4 (+, 1 × O*C*H_3_), 56.3 (+, 1 × O*C*H_3_), 50.3 (+, 1 × *C*H), 30.3 (–, 1 × *C*H_2_), 29.3 (–, 1 × *C*H_2_), 23.1 (+, 1 × *C*H_3_) ppm. − IR (ATR): ṽ = 2842 (vw), 2931 (vw), 1676 (w), 1593 (w), 1505 (w), 1439 (w), 1370 (w), 1329 (w), 1256 (m), 1201 (w), 1170 (w), 1050 (w), 1023 (w), 937 (w), 851 (w), 786 (w), 742 (w), 681 (vw), 593 (vw), 429 (vw) cm^–1^. – MS (FAB, 3‑NBA), *m*/*z* (%): 468/470 (3/5) [M]^+^, 469/471 (10/9) [M+H]^+^, 245 (37). – HRMS (FAB, C_24_H_22_O_5_^79^Br): calc. 469.0651; found 469.0650.
Additional reaction details and data obtained from the characterization of the target compound can be accessed at: <https://dx.doi.org/10.14272/reaction/SA-FUHFF-UHFFFADPSC-PWQZDRACEZ-UHFFFADPSC-NUHFF-NGJAF-NUHFF-ZZZ>.

**(4a*R*,9a*R*)-4a-(2-Bromo-4,5-dimethoxybenzoyl)-2,3-dimethyl-1,4,4a,9a-tetrahydroanthracene-9,10-dione (8dc)**

According to **GP C** the cycloaddition was performed with 2‑(2‑bromo-4,5-dimethoxybenzoyl)naphthalene-1,4-dione (**5d**) (100 mg, 250 µmol, 1.00 equiv) and 2,3-dimethylbuta-1,3-diene (**6c**) (0.15 mL, 103 mg, 1.25 mmol, 5.00 equiv.) in dry CH_2_Cl_2_ (1.5 mL). After 3 h the crude product was purified *via* flash chromatography on silica gel (cHex/EtOAc = 3:1) to obtain product **8dc** as a yellow solid (47.1 mg, 97.5 µmol, 39%). − *R*_f_ (cHex/EtOAc = 3:1) = 0.31. − ^1^H NMR (500 MHz, CDCl_3_): *δ* = 8.13 (dd, ^3^*J* = 7.6 Hz, ^4^*J* = 1.4 Hz, 1H, C*H*_Ar_), 8.00 (dd, ^3^*J* = 7.7 Hz, ^4^*J* = 1.3 Hz, 1H, C*H*_Ar_), 7.77 (td, ^3^*J* = 7.5 Hz, ^4^*J* = 1.4 Hz, 1H, C*H*_Ar_), 7.72 (td, ^3^*J* = 7.5 Hz, ^4^*J* = 1.4 Hz, 1H, C*H*_Ar_), 7.00 (s, 1H, C*H*_Ar_), 6.91 (s, 1H, C*H*_Ar_), 3.87 (s, 3H, OC*H*_3_), 3.83 (s, 3H, OC*H*_3_), 3.74–3.69 (m, 1H, C*H*), 2.94–2.77 (m, 1H, C*H*H), 2.43–2.23 (m, 3H, CH*H* + C*H*_2_), 1.59 (s, 3H, C*H*_3_), 1.55 (s, 3H, C*H*_3_) ppm. − ^13^C NMR (126 MHz, CDCl_3_): *δ* = 198.9 (C_q_, 1 × *C*=O), 196.5 (C_q_, 1 × *C*=O), 195.6 (C_q_, 1 × *C*=O), 150.8 (C_q_, 1 × *C*_qAr_), 147.4 (C_q_, 1 × *C*_qAr_), 135.2 (C_q_, 1 × *C*_qAr_), 134.3 (+, 2 × *C*H_Ar_), 133.3 (C_q_, 1 × *C*_qAr_), 130.9 (C_q_, 1 × *C*_qAr_), 127.2 (+, 1 × *C*H_Ar_), 127.1 (+, 1 × *C*H_Ar_), 124.1 (C_q_, 1 × *C*_qAr_), 122.9 (C_q_, 1 × *C*_q_), 116.7 (+, 1 × *C*H_Ar_), 111.8 (C_q_, 1 × *C*_q_), 110.9 (+, 1 × *C*H_Ar_), 68.7 (C_q_, 1 × *C*_q_), 56.4 (+, 1 × O*C*H_3_), 56.3 (+, 1 × O*C*H_3_), 50.4 (+, 1 × *C*H), 34.9 (–, 1 × *C*H_2_), 31.6 (–, 1 × *C*H_2_), 19.1 (+, 1 × *C*H_3_), 18.8 (+, 1 × *C*H_3_) ppm. – IR (ATR): ṽ = 2911 (w), 1676 (vs), 1592 (s), 1504 (vs), 1439 (m), 1370 (m), 1329 (m), 1256 (vs), 1200 (vs), 1169 (vs), 1057 (s), 1024 (s), 858 (w), 788 (m), 749 (vs) cm^–1^. – MS (FAB, 3‑NBA), *m*/*z* (%): 483/485 (9/7) [M+H]^+^, 243/245 (80/80) [C_9_H_9_BrO_3_]. – HRMS (FAB, C_25_H_24_O_5_^79^Br): calc. 483.0807; found 483.0808.
Additional reaction details and data obtained from the characterization of the target compound can be accessed at: <https://dx.doi.org/10.14272/reaction/SA-FUHFF-UHFFFADPSC-FDOSUKYERH-UHFFFADPSC-NUHFF-NUHFF-NUHFF-ZZZ>.

**(4a*R*,9a*R*)-4a-(2-Bromo-4,5-dihydroxybenzoyl)-2,3-dimethyl-1,4,4a,9a-tetrahydroanthracene-9,10-dione (8ec)**

According to **GP C** the cycloaddition was performed with 2-(2-bromo-4,5-dihydroxybenzoyl)naphthalene-1,4-dione (**5e**) (56.0 mg, 150 µmol, 1.00 equiv.) and 2,3-dimethylbuta-1,3-diene (**6c**) (0.09 mL, 61.6 mg, 750 µmol, 5.00 equiv.) in dry CH_2_Cl_2_ (2.0 mL). After 4 h the crude product was purified *via* flash chromatography on silica gel (cHex/EtOAc = 3:1) to obtain product **8ec** as a yellow solid (36.9 mg, 81.0 µmol, 54%). − *R*_f_ (cHex/EtOAc = 3:1) = 0.08. − ^1^H NMR (500 MHz, CDCl_3_): *δ* = 8.13 (d, ^3^*J* = 7.7 Hz, 1H, C*H*_Ar_), 7.97 (d, ^3^*J* = 7.7 Hz, 1H, C*H*_Ar_), 7.77 (t, ^3^*J* = 7.5 Hz, 1H, C*H*_Ar_), 7.71 (t, ^3^*J* = 7.5 Hz, 1H, C*H*_Ar_), 7.06 (s, 1H, C*H*_Ar_), 6.93 (s, 1H, C*H*_Ar_), 3.68 (dd, ^3^*J* = 8.6, 6.6 Hz, 1H, C*H*), 2.83 (d, ^2^*J* = 17.1 Hz, 1H, CH*H*), 2.37−2.22 (m, 3H, CH*H* + C*H*_2_), 1.59 (s, 3H, C*H*_3_), 1.56 (s, 3H, C*H*_3_) ppm. Missing signals (2H, 2 × O*H*). − ^13^C NMR (126 MHz, CDCl_3_): *δ* = 199.0 (C_q_, 1 × *C*=O), 196.9 (C_q_, 1 × *C*=O), 195.8 (C_q_, 1 × *C*=O), 146.6 (C_q_, 1 × *C*_qAr_), 142.1 (C_q_, 1 × *C*_qAr_), 135.3 (+, 1 × *C*H_Ar_), 134.4 (+, 1 × *C*H_Ar_), 134.3 (C_q_, 1 × *C*_qAr_), 133.3 (C_q_, 1 × *C*_qAr_), 130.9 (C_q_, 1 × *C*_qAr_), 127.3 (+, 1 × *C*H_Ar_), 127.2 (+, 1 × *C*H_Ar_), 124.0 (C_q_, 1 × *C*_qAr_), 123.0 (C_q_, 1 × *C*_q_), 120.8 (+, 1 × *C*H_Ar_), 114.8 (+, 1 × *C*H_Ar_), 111.4 (C_q_, 1 × *C*_q_), 68.7 (C_q_, 1 × *C*_q_), 50.5 (+, 1 × *C*H), 34.9 (–, 1 × *C*H_2_), 31.6 (–, 1 × *C*H_2_), 19.1 (+, 1 × *C*H_3_), 18.8 (+, 1 × *C*H_3_) ppm. – IR (ATR): ṽ = 3338 (vw), 2923 (w), 1678 (w), 1590 (w), 1501 (w), 1415 (w), 1257 (w), 1173 (w), 1093 (w), 1043 (w), 1021 (w), 944 (vw), 863 (vw), 797 (w), 753 (w), 728 (vw), 675 (vw), 634 (vw), 608 (vw), 560 (vw), 448 (vw), 399 (vw) cm^–1^. – MS (APCI), *m*/*z* (%): 455/457 (97/100) [M+H]^+^. – HRMS (APCI, C_23_H_20_^79^BrO_5_): calc. 455.0494; found 455.0476.
Additional reaction details and data obtained from the characterization of the target compound can be accessed at: <https://dx.doi.org/10.14272/reaction/SA-FUHFF-UHFFFADPSC-GNLDMLVTJD-UHFFFADPSC-NUHFF-NLDCB-NUHFF-ZZZ>.

**Cycloaddition product of *N*-(4-bromo-3-(1,4-dioxo-1,4-dihydronaphthalene-2-carbonyl)phenyl)acetamide (5f) and isoprene (6b) (8/9fb)**

According to **GP C** the cycloaddition was performed with *N*‑(4‑bromo-3-(1,4-dioxo-1,4-dihydronaphthalene-2-carbonyl)­phenyl)­acetamide (**5f**) (79.6 mg, 200 µmol, 1.00 equiv.) and isoprene (**6b**) (0.06 mL, 40.9 mg, 600 µmol, 3.00 equiv.) in dry CH_2_Cl_2_ (1.0 mL). After 4 h the crude product was purified *via* flash chromatography on silica gel (cHex/EtOAc = 1:2) to obtain a yellow solid (35.4 mg, 76.0 µmol, 38%). The products **8/9fb** were isolated as a non-separable mixture in a ∼ 7.7 : 1 ratio as estimated by ^1^H NMR. Both possible regioisomers are drawn, as the exact structure of the products could not be resolved by analysis of the NMR spectra. Below the analytics of the major product are given. − *R*_f_ (cHex/EtOAc = 1:2) = 0.30. − ^1^H NMR (500 MHz, CDCl_3_): *δ* = 8.09 (dd, ^3^*J* = 7.7 Hz, ^4^*J* = 1.3 Hz, 1H, C*H*_Ar_), 8.03 (brs, 1H, N*H*), 7.99 (dd, ^3^*J* = 7.7 Hz, ^4^*J* = 1.4 Hz, 1H, C*H*_Ar_), 7.74 (td, ^3^*J* = 7.5 Hz, ^4^*J* = 1.4 Hz, 1H, C*H*_Ar_), 7.67 (td, ^3^*J* = 7.5 Hz, ^4^*J* = 1.4 Hz, 1H, C*H*_Ar_), 7.54 (t, ^4^*J* = 1.5 Hz, 1H, C*H*_Ar_), 7.42 (d, ^4^*J* = 1.5 Hz, 2H, C*H*_Ar_), 5.27 (tq, ^4^*J* = 3.5, 1.7 Hz, 1H, C=C*H*), 3.74 (dd, ^3^*J* = 8.2, 6.6 Hz, 1H, C*H*), 2.84 (ddq, ^2^*J* = 17.4 Hz, ^3^*J* = 4.1 Hz, ^4^*J* = 1.9 Hz, 1H, C*H*H), 2.49 (ddq, ^2^*J* = 17.4 Hz, ^3^*J* = 4.2 Hz, ^4^*J* = 2.1 Hz, 1H, CH*H*), 2.40–2.19 (m, 2H, C*H*_2_), 2.13 (s, 3H, COC*H*_3_), 1.62 (d, ^4^*J* = 2.0 Hz, 3H, CH=CC*H*_3_) ppm. − ^13^C NMR (126 MHz, CDCl_3_): *δ* = 200.2 (C_q_, 1 × *C*=O), 196.4 (C_q_, 1 × *C*=O), 194.6 (C_q_, 1 × *C*=O), 169.0 (C_q_, 1 × *C*_qAr_), 139.1 (C_q_, 1 × *C*_qAr_), 137.1 (C_q_, 1 × *C*_qAr_), 135.3 (+, 1 × *C*H_Ar_), 134.4 (+, 1 × *C*H_Ar_), 134.3 (+, 1 × *C*H_Ar_), 134.1 (C_q_, 1 × *C*_qAr_), 133.0 (C_q_, 1 × *C*_qAr_), 132.3 (C_q_, 1 × *C*_q_), 127.6 (+, 1 × *C*H_Ar_), 127.0 (+, 1 × *C*H_Ar_), 122.4 (+, 1 × *C*H_Ar_), 118.2 (+, 1 × *C*H_Ar_), 117.7 (+, 1 × C=*C*H), 113.7 (C_q_, 1 × *C*_q_), 67.7 (C_q_, 1 × *C*_q_), 49.9 (+, 1 × *C*H), 29.9 (–, 1 × *C*H_2_), 28.6 (–, 1 × *C*H_2_), 24.6 (+, 1 × CO*C*H_3_), 23.2 (+, 1 × C=C*C*H_3_) ppm. Missing signal (C_q_, 1 × *C*=O). – IR (ATR): ṽ = 3306 (w), 2912 (w), 1677 (vs), 1581 (vs), 1530 (vs), 1466 (vs), 1391 (vs), 1373 (s), 1252 (vs), 1187 (vs), 1047 (s), 1016 (vs), 936 (s), 909 (m), 802 (vs), 773 (s), 728 (vs), 688 (s), 647 (s), 591 (s), 561 (s), 484 (s), 418 (s) cm^–1^. – MS (FAB, 3‑NBA), *m*/*z* (%): 466/468 (19/20) [M+H]^+^, 242 (97), 240 (100). – HRMS (FAB, C_24_H_21_O_4_N^79^Br): calc. 466.0654; found 466.0652.
Additional reaction details and data obtained from the characterization of the target compound can be accessed at: <https://dx.doi.org/10.14272/reaction/SA-FUHFF-UHFFFADPSC-YMLGBYYTIH-UHFFFADPSC-NUHFF-NRQYZ-NUHFF-ZZZ>.

***N*-(4-Bromo-3-((4a*R*,9a*R*)-2,3-dimethyl-9,10-dioxo-1,4,4a,9,9a,10-hexahydroanthracene-4a-carbonyl)phenyl)acetamide (8fc)**

According to **GP C** the cycloaddition was performed with *N*-(4-bromo-3-(1,4-dioxo-1,4-dihydro­naphthalene-2-carbonyl)phenyl)acetamide (**5f**) (119 mg, 300 µmol, 1.00 equiv.) and 2,3‑dimethylbuta-1,3-diene (**6c**) (0.10 mL, 73.9 mg, 900 µmol, 3.00 equiv.) in dry CH_2_Cl_2_ (1.0 mL). After 5 h the crude product was purified *via* flash chromatography on silica gel (cHex/EtOAc = 1:1) to obtain product **8fc** as a yellow solid (98.0 mg, 204 µmol, 68%). − *R*_f_ (cHex/EtOAc = 1:1) = 0.30. − ^1^H NMR (500 MHz, CDCl_3_): *δ* = 8.13 (d, ^3^*J* = 7.6 Hz, 1H, C*H*_Ar_), 8.06 (d, ^3^*J* = 7.5 Hz, 1H, C*H*_Ar_), 7.76 (dt, ^3^*J* = 21.3, 7.4 Hz, 2H, C*H*_Ar_), 7.52−7.47 (m, 3H, C*H*_Ar_), 7.40 (brs, 1H, N*H*), 3.74 (t, ^3^*J* = 7.3 Hz, 1H, C*H*), 2.77 (d, ^2^*J* = 17.1 Hz, 1H, C*H*H), 2.42−2.36 (m, 2H, C*H*H + C*H*H), 2.33−2.23 (m, 1H, C*H*H), 2.18 (s, 3H, COC*H_3_*), 1.59 (s, 3H, C*H_3_*), 1.49 (s, 3H, C*H_3_*) ppm. − ^13^C NMR (126 MHz, CDCl_3_): *δ* = 200.0 (C_q_, 1 × *C*=O), 196.6 (C_q_, 1 × *C*=O), 195.2 (C_q_, 1 × *C*=O), 168.6 (C_q_, 1 × *C*=O), 139.3 (C_q_, 1 × *C*_qAr_), 136.8 (C_q_, 1 × *C*_qAr_), 135.3 (+, 1 × *C*H_Ar_), 134.4 (+, 1 × *C*H_Ar_), 134.3 (+, 1 × *C*H_Ar_), 133.1 (C_q_, 1 × *C*_qAr_), 127.6 (+, 1 × *C*H_Ar_), 127.1 (+, 1 × *C*H_Ar_), 124.4 (C_q_, 1 × *C*_qAr_), 122.8 (C_q_, 1 × *C*_qAr_), 122.3 (+, 1 × *C*H_Ar_), 118.3 (+, 1 × *C*H_Ar_), 114.0 (C_q_, 1 × *C*_q_), 68.5 (C_q_, 1 × *C*_q_), 50.1 (+, 1 × *C*H), 34.3 (–, 1 × *C*H_2_), 31.3 (–, 1 × *C*H_2_), 24.8 (+, 1 × *C*H_3_), 19.1 (+, 1 × *C*H_3_), 18.9 (+, 1 × *C*H_3_), 14.3 (C_q_, 1 × *C*_q_) ppm. − IR (ATR): ṽ = 3310 (vw), 3265 (vw), 3186 (vw), 3111 (vw), 3065 (vw), 2928 (vw), 2858 (vw), 1678 (m), 1591 (w), 1538 (m), 1471 (w), 1370 (w), 1317 (w), 1247 (m), 1199 (w), 1012 (w), 886 (w), 856 (w), 835 (w), 779 (w), 755 (w), 718 (w), 592 (w), 558 (w), 486 (w), 421 (w), 388 (w) cm^–1^. – MS (FAB, 3‑NBA), *m*/*z* (%): 480 (13) [M+H]^+^, 240/242 (100/95). – HRMS (FAB, C_25_H_23_O_4_N^79^Br): calc. 480.0810; found. 480.0811. − X‑Ray: The structure of **8fc** could be confirmed by single crystal X‑Ray diffraction (see 4 Crystallographic Information, CCDC 1992890).
Additional reaction details and data obtained from the characterization of the target compound can be accessed at: <https://dx.doi.org/10.14272/reaction/SA-FUHFF-UHFFFADPSC-BBXGNCLCSR-UHFFFADPSC-NUHFF-NUHFF-NUHFF-ZZZ>.

**Cycloaddition product of *N*-(4-bromo-3-(1,4-dioxo-1,4-dihydronaphthalene-2-carbonyl)phenyl)-2,2,2-trifluoroacetamide (5g) and isoprene (6b) (8/9gb)**

According to **GP C** the cycloaddition was performed with *N*‑(4‑bromo-3-(1,4-dioxo-1,4-dihydronaphthalene-2-carbonyl)­phenyl)-2,2,2-trifluoroacetamide (**5g**) (90.4 mg, 200 µmol, 1.00 equiv.) and isoprene (**6b**) (0.06 mL, 40.9 mg, 600 µmol, 3.00 equiv.) in dry CH_2_Cl_2_ (1.0 mL). After 3 h the crude product was purified *via* flash chromatography on silica gel (cHex/EtOAc = 4:1) to obtain a light yellow solid (82.2 mg, 158 µmol, 79%). The products **8/9gb** were isolated as a non-separable mixture in in a ∼ 7.7 : 1 ratio as estimated by ^1^H NMR. Both possible regioisomers are drawn, as the exact structure of the products could not be resolved by analysis of the NMR spectra. Below the analytics of the major product are given. − *R*_f_ (cHex/EtOAc = 4:1) = 0.32. − ^1^H NMR (500 MHz, CDCl_3_): *δ* = 8.73 (brs, 1H, N*H*), 8.08 (dd, ^3^*J* = 7.6 Hz, ^4^*J* = 1.4 Hz, 1H, C*H*_Ar_), 7.99 (dd, ^3^*J* = 7.6 Hz, ^4^*J* = 1.4 Hz, 1H, C*H*_Ar_), 7.76 (td, ^3^*J* = 7.5 Hz, ^4^*J* = 1.5 Hz, 1H, C*H*_Ar_), 7.72 (td, ^3^*J* = 7.5 Hz, ^4^*J* = 1.5 Hz, 1H, C*H*_Ar_), 7.67 (d, ^3^*J* = 2.4 Hz, 1H, C*H*_Ar_), 7.57–7.48 (m, 2H, C*H*_Ar_), 5.25 (tt, ^3^*J* = 3.8 Hz, ^4^*J* = 1.7 Hz, 1H, C=C*H*), 3.79 (dd, ^3^*J* = 7.7, 6.4 Hz, 1H, C*H*), 2.84–2.78 (m, 1H, C*H*H), 2.53–2.48 (m, 1H, CH*H*), 2.42–2.30 (m, 1H, C*H*H), 2.25 (dd, ^3^*J* = 18.2, 6.4 Hz, 1H, C*H*H), 1.63 (s, 3H, C*H*_3_) ppm. − ^13^C NMR (126 MHz, CDCl_3_): *δ* = 200.1 (C_q_, 1 × *C*=O), 196.6 (C_q_, 1 × *C*=O), 194.9 (C_q_, 1 × *C*=O), 155.4 (q, ^2^*J* = 38.1 Hz, 1 × *C*OCF_3_), 139.5 (C_q_, 1 × *C*_qAr_), 135.4 (+, 1 × *C*H_Ar_), 134.8 (+, 1 × *C*H_Ar_), 134.6 (+, 1 × *C*H_Ar_), 134.3 (C_q_, 1 × *C*_qAr_), 134.1 (C_q_, 1 × *C*_qAr_), 132.8 (C_q_, 1 × *C*_qAr_), 132.8 (C_q_, 1 × *C*_qAr_), 127.7 (+, 1 × *C*H_Ar_), 127.0 (+, 1 × *C*H_Ar_), 123.3 (+, 1 × *C*H_Ar_), 119.4 (+, 1 × *C*H_Ar_), 117.4 (+, 1 × C=*C*H), 115.6 (q, ^1^*J* = 288.0 Hz, 1 × *C*F_3_), 67.6 (C_q_, 2 × *C*_q_), 49.8 (+, 1 × *C*H), 29.6 (–, 1 × *C*H_2_), 28.7 (–, 1 × *C*H_2_), 23.2 (+, 1 × *C*H_3_) ppm. − IR (ATR): ṽ = 3298 (w), 2917 (w), 1684 (vs), 1589 (s), 1545 (m), 1468 (m), 1276 (s), 1252 (vs), 1210 (vs), 1150 (vs), 1047 (s), 936 (w), 899 (w), 824 (m), 800 (w), 738 (s), 725 (m), 686 (w), 591 (w), 562 (w), 484 (w), 435 (w) cm^–1^. – MS (FAB, 3‑NBA), *m*/*z* (%): 520/522 (27/23) [M+H]^+^, 294/296 (100/98), 225 (71), 209 (26). – HRMS (FAB, C_24_H_18_O_4_N^79^BrF_3_): calc. 520.0371; found 520.0370.
Additional reaction details and data obtained from the characterization of the target compound can be accessed at: <https://dx.doi.org/10.14272/reaction/SA-FUHFF-UHFFFADPSC-CYVQCLLXOP-UHFFFADPSC-NUHFF-NGMWY-NUHFF-ZZZ>.

***N*-(4-Bromo-3-((4a*S*,9a*S*)-2,3-dimethyl-9,10-dioxo-1,4,4a,9,9a,10-hexahydroanthracene-4a-carbonyl)phenyl)-2,2,2-trifluoroacetamide (8gc)**

According to **GP C** the cycloaddition was performed with *N*-(4-bromo-3-(1,4-dioxo-1,4-dihydronaphthalene-2-carbonyl)phenyl)-2,2,2-trifluoroacetamide (**5g**) (181 mg, 400 µmol, 1.00 equiv.) and 2,3-dimethylbuta-1,3-diene (**6c**) (0.14 mL, 98.6 mg, 1.20 mmol, 3.00 equiv.) in dry CH_2_Cl_2_ (4.0 mL). After 5 h the crude product was purified *via* flash chromatography on silica gel (cHex/EtOAc = 4:1) to obtain product **8gc** as a yellow solid (180 mg, 336 µmol, 84%). − *R*_f_ (cHex/EtOAc = 4:1) = 0.37. − ^1^H NMR (500 MHz, CDCl_3_): *δ* = 8.15 (brs, 1H, N*H*), 8.12 (dd, ^3^*J* = 7.5 Hz, ^4^*J* = 1.5 Hz, 1H, C*H*_Ar_), 8.07 (dd, ^3^*J* = 7.5 Hz, ^4^*J* = 1.5 Hz, 1H, C*H*_Ar_), 7.78 (dtd, ^3^*J* = 17.0, 7.5 Hz, ^4^*J* = 1.5 Hz, 2H, C*H*_Ar_), 7.65 (d, ^3^*J* = 2.5 Hz, 1H, C*H*_Ar_), 7.60 (d, ^3^*J* = 8.7 Hz, 1H, C*H*_Ar_), 7.55 (dd, ^3^*J* = 8.7 Hz, ^4^*J* = 2.5 Hz, 1H, C*H*_Ar_), 3.80 (t, ^3^*J* = 6.9 Hz, 1H, C*H*), 2.73 (d, ^2^*J* = 17.1 Hz, 1H, C*H*H), 2.49−2.40 (m, 2H, 2 × C*H*H), 2.34−2.24 (m, 1H, C*H*H), 1.61 (s, 3H, C*H*_3_), 1.45 (s, 3H, C*H*_3_) ppm. − ^13^C NMR (126 MHz, CDCl_3_): *δ* = 199.9 (C_q_, 1 × *C*=O), 196.5 (C_q_, 1 × *C*=O), 195.5 (C_q_, 1 × *C*=O), 155.1 (q, ^2^*J* = 38.1 Hz, 1 × CO*C*F_3_), 139.8 (C_q_, 1 × *C*_qAr_), 135.4 (+, 1 × *C*H_Ar_), 134.9 (+, 1 × *C*H_Ar_), 134.5 (+, 1 × *C*H_Ar_), 134.3 (C_q_, 1 × *C*_qAr_), 134.0 (C_q_, 1 × *C*_qAr_), 132.9 (C_q_, 1 × *C*_qAr_), 127.7 (+, 1 × *C*H_Ar_), 127.1 (+, 1 × *C*H_Ar_), 125.1 (C_q_, 1 × *C*_qAr_), 123.0 (+, 1 × *C*H_Ar_), 122.5 (C_q_, 1 × *C*_q_), 119.4 (+, 1 × *C*H_Ar_), 116.8 (C_q_, 1 × *C*_q_), 115.6 (q, ^1^*J* = 289.0 Hz, 1 × *C*F_3_), 68.2 (C_q_, 1 × *C*_q_), 49.8 (+, 1 × *C*H), 34.4 (–, 1 × *C*H_2_), 30.8 (–, 1 × *C*H_2_), 19.1 (+, 1 × *C*H_3_), 19.0 (+, 1 × *C*H_3_) ppm. – IR (ATR): ṽ = 3361 (vw), 2911 (vw), 1699 (w), 1673 (w), 1588 (w), 1543 (vw), 1466 (vw), 1402 (vw), 1282 (w), 1258 (w), 1239 (w), 1135 (w), 1057 (w), 875 (vw), 831 (w), 793 (vw), 757 (vw), 741 (w), 728 (vw), 671 (vw), 645 (vw), 597 (vw), 566 (vw), 477 (vw), 434 (vw), 402 (vw) cm^–1^. – MS (FAB, 3-NBA), *m*/*z* (%): 534/536 (25/21) [M+H]^+^, 294 (68), 239 (87). – HRMS (FAB, C_25_H_20_O_4_N^79^BrF_3_): calc. 534.0528; found. 534.0530. − X‑Ray: The structure of **8gc** could be confirmed by single crystal X‑Ray diffraction (see 4 Crystallographic Information, CCDC 1992891).
Additional reaction details and data obtained from the characterization of the target compound can be accessed at: <https://dx.doi.org/10.14272/reaction/SA-FUHFF-UHFFFADPSC-LWPRQCGBQO-UHFFFADPSC-NUHFF-NUHFF-NUHFF-ZZZ>.

**Cycloaddition of *N*-(4-bromo-3-(1,4-dioxo-1,4-dihydronaphthalene-2-carbonyl)phenyl)-2,2,2-trifluoroacetamide (5g) and 1‑(trimethylsiloxy)-1,3-butadiene (6d) (8/9gd)**

According to **GP C** the cycloaddition was performed with *N*‑(4‑bromo-3-(1,4-dioxo-1,4-dihydronaphthalene-2-carbonyl)­phenyl)-2,2,2-trifluoroacetamide (**5g**) (136 mg, 300 µmol, 1.00 equiv.) and 1‑(tri­methyl­siloxy)-1,3-butadiene (**6d**) (0.26 mL, 213 mg, 1.50 mmol, 5.00 equiv.) in dry CH_2_Cl_2_ (2.0 mL). After 4 h the crude product was purified *via* flash chromatography on silica gel (cHex/EtOAc = 5:1) to obtain isomers **9gd** and **8gd** (ratio ∼ 3.7 : 1) as a yellow solid (total yield: 136 mg, 228 µmol, 76%). The exact structure of **9gd** could be verified by X-ray analysis. For **8gd** two possible isomers are drawn as the exact structure of the product could not be resolved by analysis of the NMR spectra.

**9gd**: *R*_f_ (cHex/EtOAc = 5:1) = 0.39. − ^1^H NMR (500 MHz, CDCl_3_): *δ* = 8.21 (d, ^3^*J* = 2.6 Hz, 1H, C*H*_Ar_),
8.15–8.12 (m, 1H, C*H*_Ar_), 8.10 (brs, 1H, N*H*), 8.07–8.02 (m, 1H, C*H*_Ar_), 7.80–7.70 (m, 2H, C*H*_Ar_), 7.63 (d, ^3^*J* = 8.7 Hz, 1H, C*H*_Ar_), 7.56 (dd, ^3^*J* = 8.7 Hz, ^4^*J* = 2.7 Hz, 1H, C*H*_Ar_), 5.95 (ddd, ^3^*J* = 10.1, 4.5 Hz, ^4^*J* = 2.9 Hz, 1H, C=C*H*), 5.62 (ddt, ^3^*J* = 10.0, 5.0 Hz, ^4^*J* = 2.3 Hz, 1H, C=C*H*), 4.90 (d, ^3^*J* = 5.3 Hz, 1H, C(OTMS)*H*), 4.01 (d, ^3^*J* = 7.2 Hz, 1H, COC*H*), 3.28–3.23 (m, 1H, C*H*H), 2.32–2.17 (m, 1H, CH*H*), –0.34 (s, 9H, Si(C*H*_3_)_3_) ppm. − ^13^C NMR (126 MHz, CDCl_3_): *δ* = 197.8 (C_q_, 1 × *C*=O), 197.4 (C_q_, 2 × *C*=O), 194.8 (C_q_, 1 × *C*=O), 155.0 (q, ^2^*J* = 38.0 Hz, 1 × *C*OCF_3_), 139.8 (C_q_, 1 × *C*_qAr_), 137.5 (C_q_, 1 × *C*_qAr_), 135.4 (+, 1 × *C*H_Ar_), 135.0 (C_q_, 1 × *C*_qAr_), 134.7 (+, 1 × *C*H_Ar_), 134.0 (C_q_, 1 × *C*_qAr_), 133.4 (+, 1 × *C*H_Ar_), 131.2 (+, 1 × C=*C*H), 127.0 (+, 1 × *C*H_Ar_), 126.2 (+, 1 × C=*C*H), 125.8 (+, 1 × *C*H_Ar_), 123.4 (+, 1 × *C*H_Ar_), 120.5 (+, 1 × *C*H_Ar_), 118.1 (C_q_, 1 × *C*_qAr_), 115.6 (q, ^1^*J* = 290.0 Hz, 1 × *C*F_3_), 70.7 (C_q_, 1 × *C*_q_), 68.0 (+, 1 × *C*H), 45.7 (+, 1 × *C*H), 21.2 (–, 1 × *C*H_2_), −0.6 (+, 3 × Si*C*H_3_) ppm. – IR (ATR): ṽ = 1732 (s), 1701 (m), 1687 (vs), 1664 (s), 1592 (w), 1545 (s), 1472 (w), 1279 (s), 1254 (vs), 1187 (vs), 1157 (vs), 1079 (s), 1061 (vs), 1044 (s), 909 (w), 867 (vs), 841 (vs), 765 (m), 738 (s), 691 (vs), 670 (m), 611 (m), 497 (m), 475 (m), 416 (m) cm^–1^. – MS (FAB, 3-NBA) *m*/*z* (%): 594/596 (28/24) [M+H]^+^, 299 (36), 294/296 (91/87). – HRMS (FAB, C_26_H_24_O_5_N^79^BrF_3_Si): calc. 594.0559; found 594.0561. − X‑Ray: The structure of **9gd** could be confirmed by single crystal X‑Ray diffraction (see 4 Crystallographic Information, CCDC 1992892).
Additional reaction details and data obtained from the characterization of the target compound can be accessed at: <https://dx.doi.org/10.14272/reaction/SA-FUHFF-UHFFFADPSC-SMNBKPYPJX-UHFFFADPSC-NUHFF-NDPHN-NUHFF-ZZZ>.

**8gd**: *R*_f_ (cHex/EtOAc = 5:1) = 0.31. − ^1^H NMR (500 MHz, CDCl_3_): *δ* = 8.26 (brs, 1H, N*H*), 8.08 (s, 1H, C*H*_Ar_), 8.05 (d, ^3^*J* = 7.8 Hz, 1H, C*H*_Ar_), 7.97 (d, ^3^*J* = 7.8 Hz, 1H, C*H*_Ar_), 7.76 (td, ^3^*J* = 7.6 Hz, ^4^*J* = 1.3 Hz, 1H, C*H*_Ar_), 7.68 (td, ^3^*J* = 7.6 Hz, ^4^*J* = 1.3 Hz, 1H, C*H*_Ar_), 7.54 (s, 2H, C*H*_Ar_), 6.04−6.00 (m, 1H, C=C*H*), 5.85−5.81 (m, 1H, C=C*H*), 5.38 (d, ^3^*J* = 5.2 Hz, 1H, C(OTMS)*H*), 3.90 (dd, ^3^*J* = 11.6, 6.6 Hz, 1H, COC*H*), 2.58−2.52 (m, 1H, C*H*H), 2.12−2.05 (m, 1H, CH*H*), 0.01 (s, 9H, Si(C*H*_3_)_3_) ppm. − ^13^C NMR (126 MHz, CDCl_3_): *δ* = 196.2 (C_q_, 1 × *C*=O), 196.0 (C_q_, 1 × *C*=O), 192.8 (C_q_, 1 × *C*=O), 155.2 (q, ^2^*J* = 38.0 Hz, 1 × *C*OCF_3_), 138.9 (C_q_, 1 × *C*_qAr_), 135.5 (+, 1 × *C*H_Ar_), 135.5 (+, 1 × *C*H_Ar_), 134.6 (C_q_, 1 × *C*_qAr_), 134.4 (+, 1 × *C*H_Ar_), 134.1 (C_q_, 1 × *C*_qAr_), 133.8 (C_q_, 1 × *C*_qAr_), 127.6 (+, 1 × C=*C*H), 127.5 (+, 1 × *C*H_Ar_), 127.4 (+, 1 × *C*H_Ar_), 127.2 (+, 1 × C=*C*H), 123.7 (+, 1 × *C*H_Ar_), 120.4 (+, 1 × *C*H_Ar_), 118.4 (C_q_, 1 × *C*_qAr_), 115.7 (q, ^1^*J* = 291.0 Hz, 1 × *C*F_3_), 73.7 (C_q_, 1 × *C*_q_), 66.1 (+, 1 × *C*H), 46.1 (+, 1 × *C*H), 28.0 (–, 1 × *C*H_2_), 0.6 (+, 3 × Si*C*H_3_) ppm. – IR (ATR): ṽ = 3289 (w), 1730 (s), 1713 (s), 1683 (vs), 1587 (s), 1545 (w), 1468 (m), 1404 (w), 1290 (s), 1248 (vs), 1218 (vs), 1203 (vs), 1154 (vs), 1137 (vs), 1072 (vs), 1051 (vs), 1021 (s), 945 (m), 894 (vs), 836 (vs), 782 (s), 745 (vs), 718 (vs), 701 (s), 645 (m), 608 (s), 578 (s), 492 (s), 401 (s) cm^–1^. – MS (FAB, 3-NBA) *m*/*z* (%): 594/596 (15/13) [M+H]^+^, 452/454 (20/22), 299 (40), 294/296 (100/96). – HRMS (FAB, C_26_H_24_O_5_N^79^BrF_3_Si): calc. 594.0559; found 594.0561.
Additional reaction details and data obtained from the characterization of the target compound can be accessed at: <https://dx.doi.org/10.14272/reaction/SA-FUHFF-UHFFFADPSC-SMNBKPYPJX-UHFFFADPSC-NUHFF-NDPHN-NUHFF-ZZZ>.

## ^1^H and ^13^C NMR Spectra of the Products


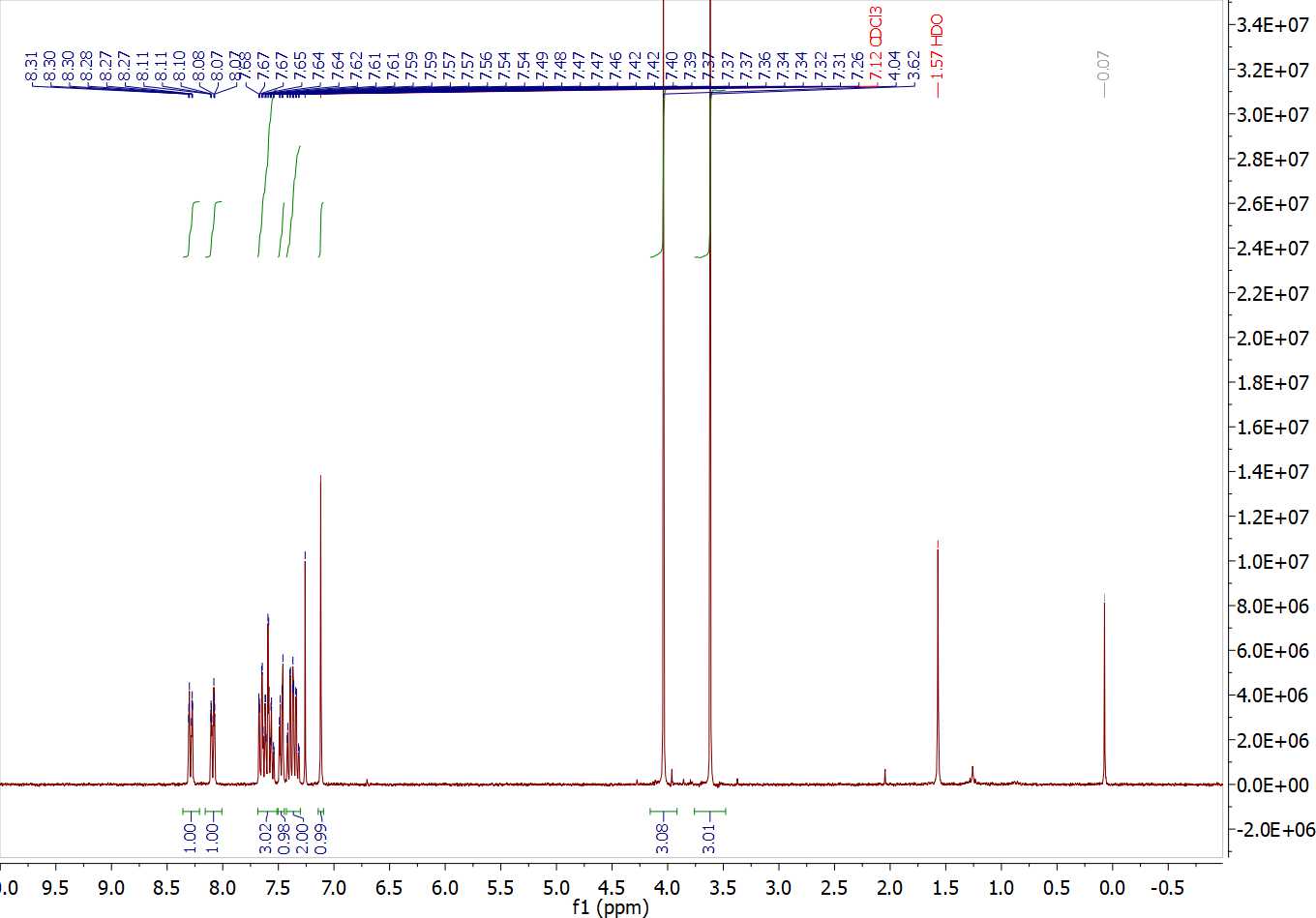


**Figure 1** ^1^H NMR (400 MHz, CDCl_3_) **4b** Br dienophile precursor.


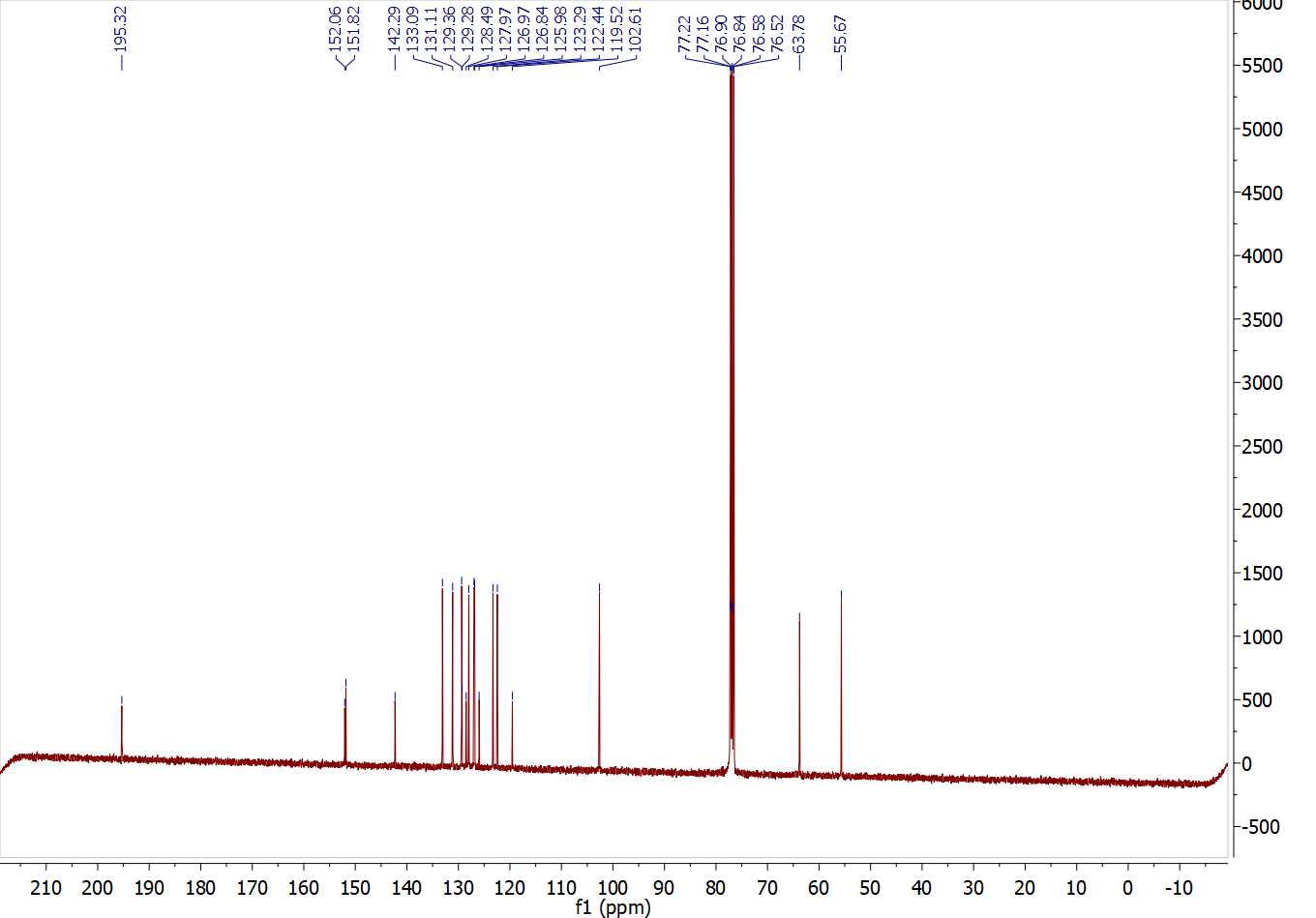


**Figure 2** ^13^C NMR (101 MHz, CDCl_3_) **4b** Br dienophile precursor.


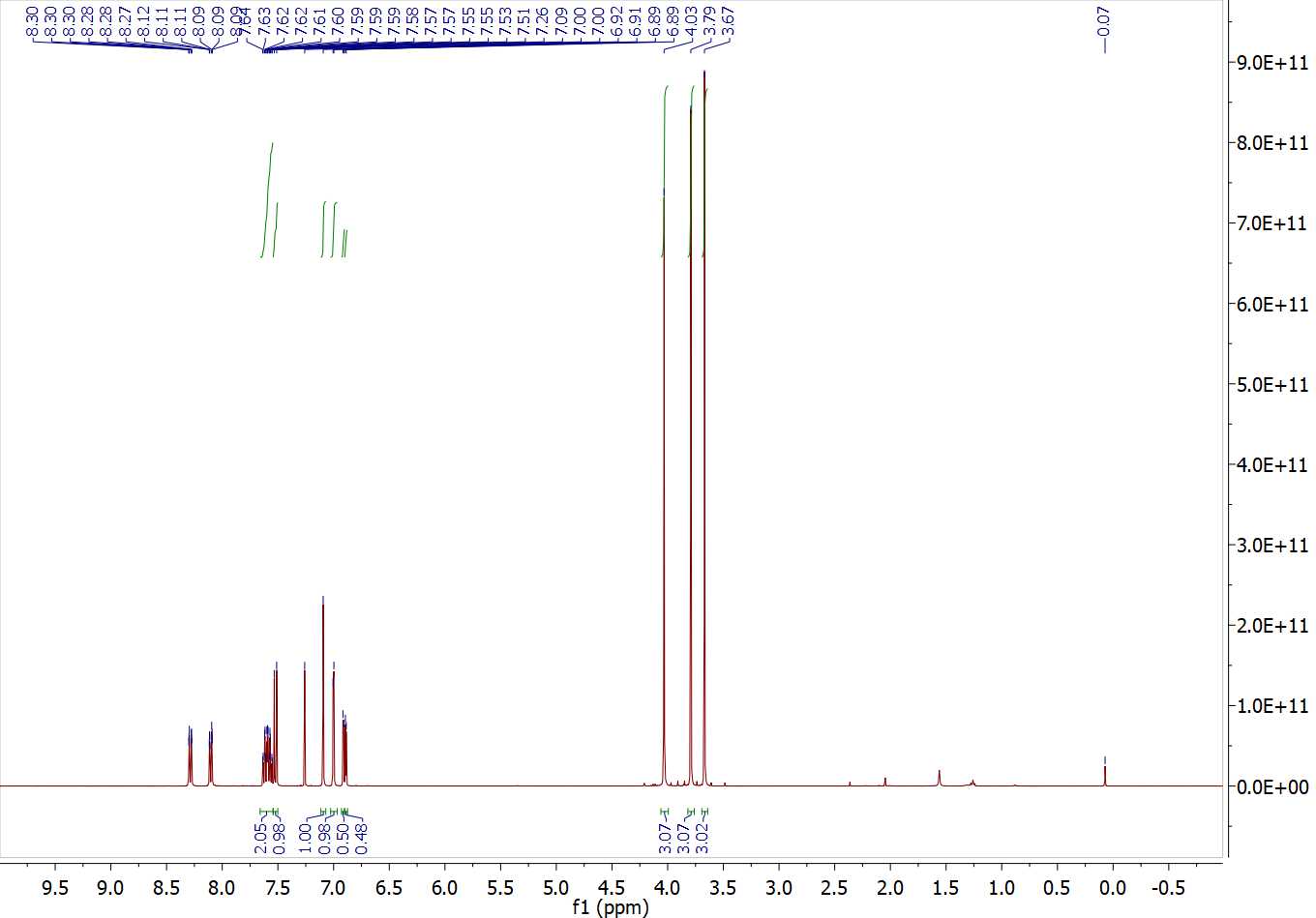


**Figure 3** ^1^H NMR (400 MHz, CDCl_3_) **4c** Br MeO dienophile precursor.


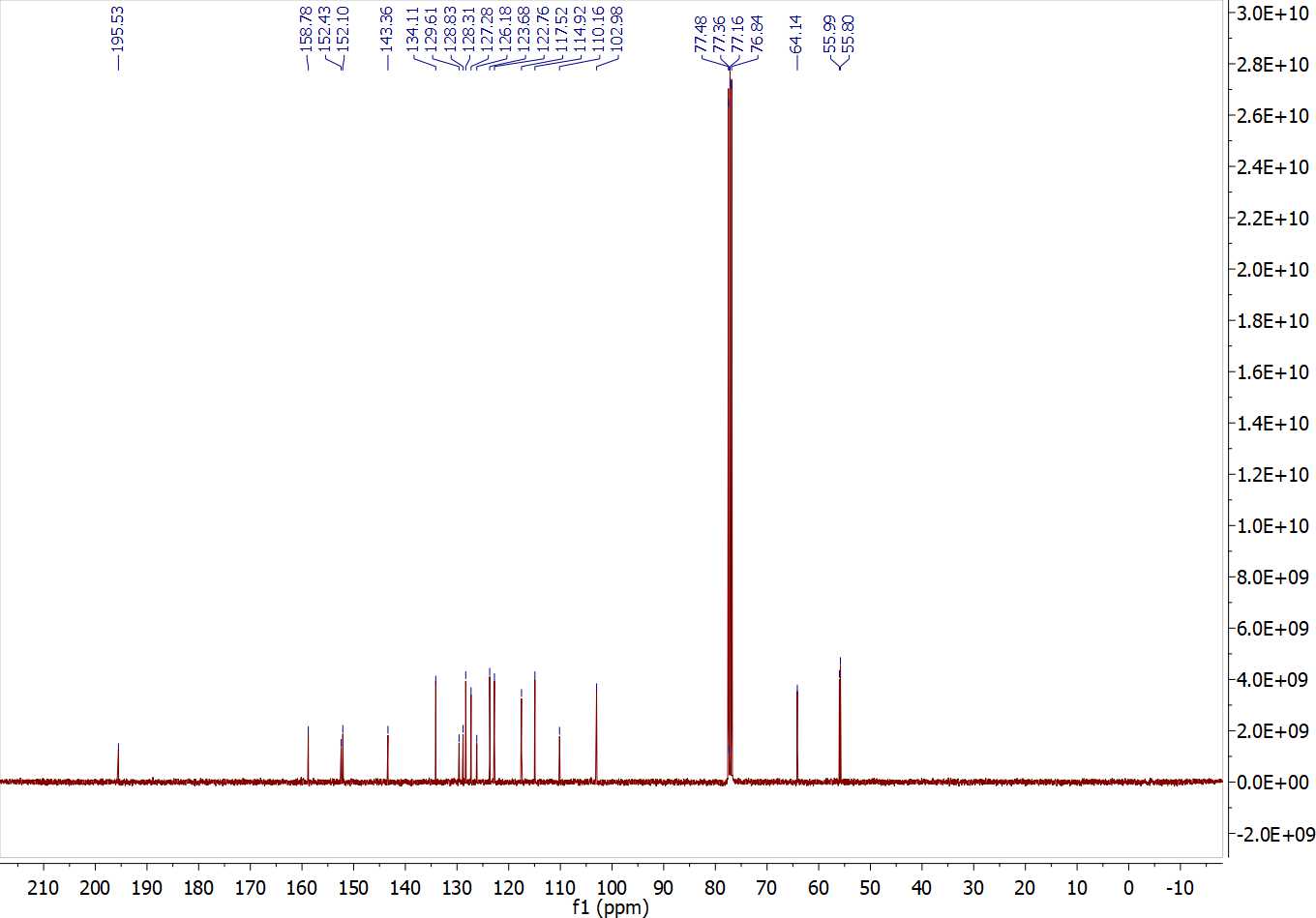


**Figure 4** ^13^C NMR (101 MHz, CDCl_3_) **4c** Br MeO dienophile precursor.


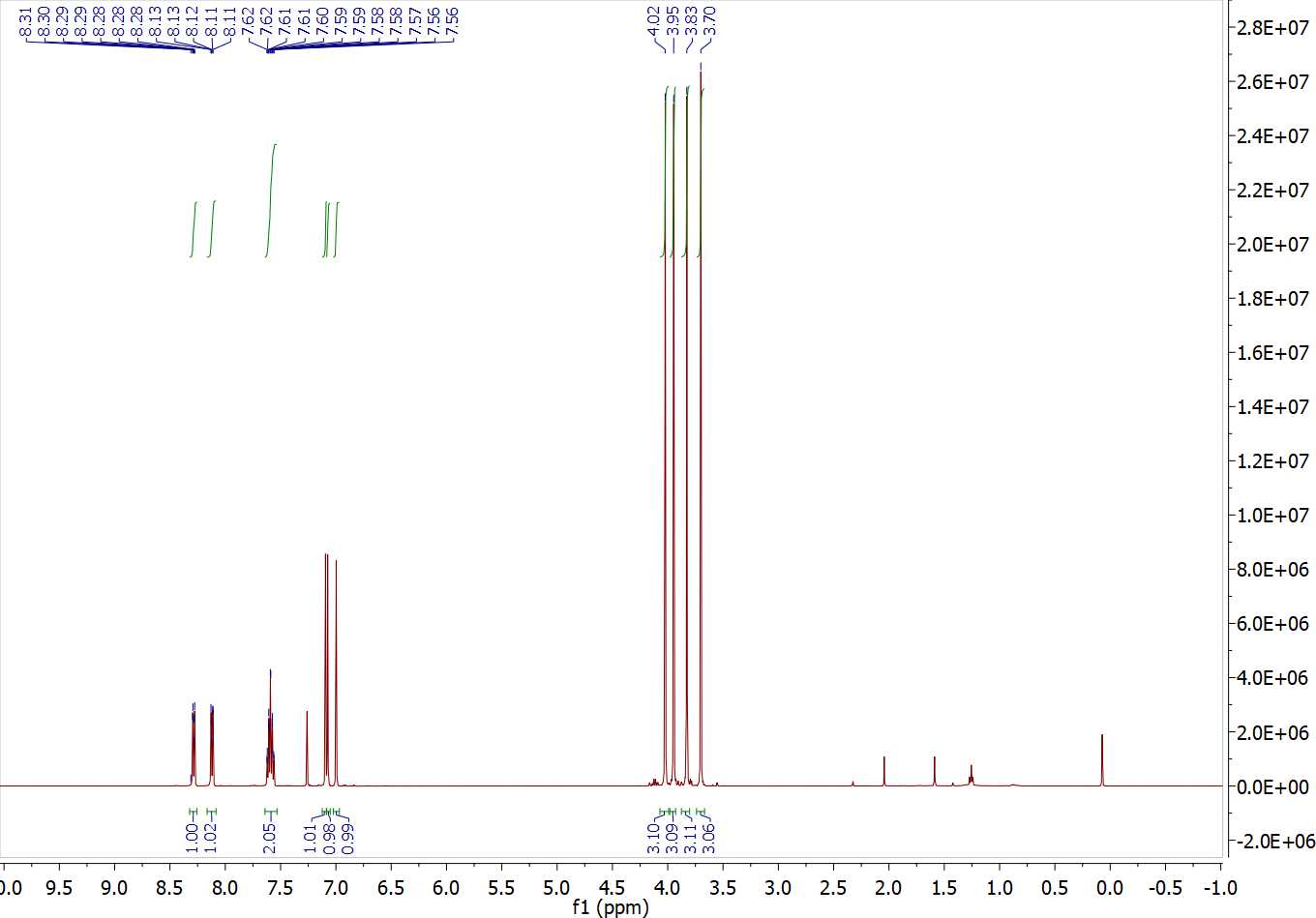


**Figure 5** ^1^H NMR (400 MHz, CDCl_3_) **4d** Br DiMeO dienophile precursor.


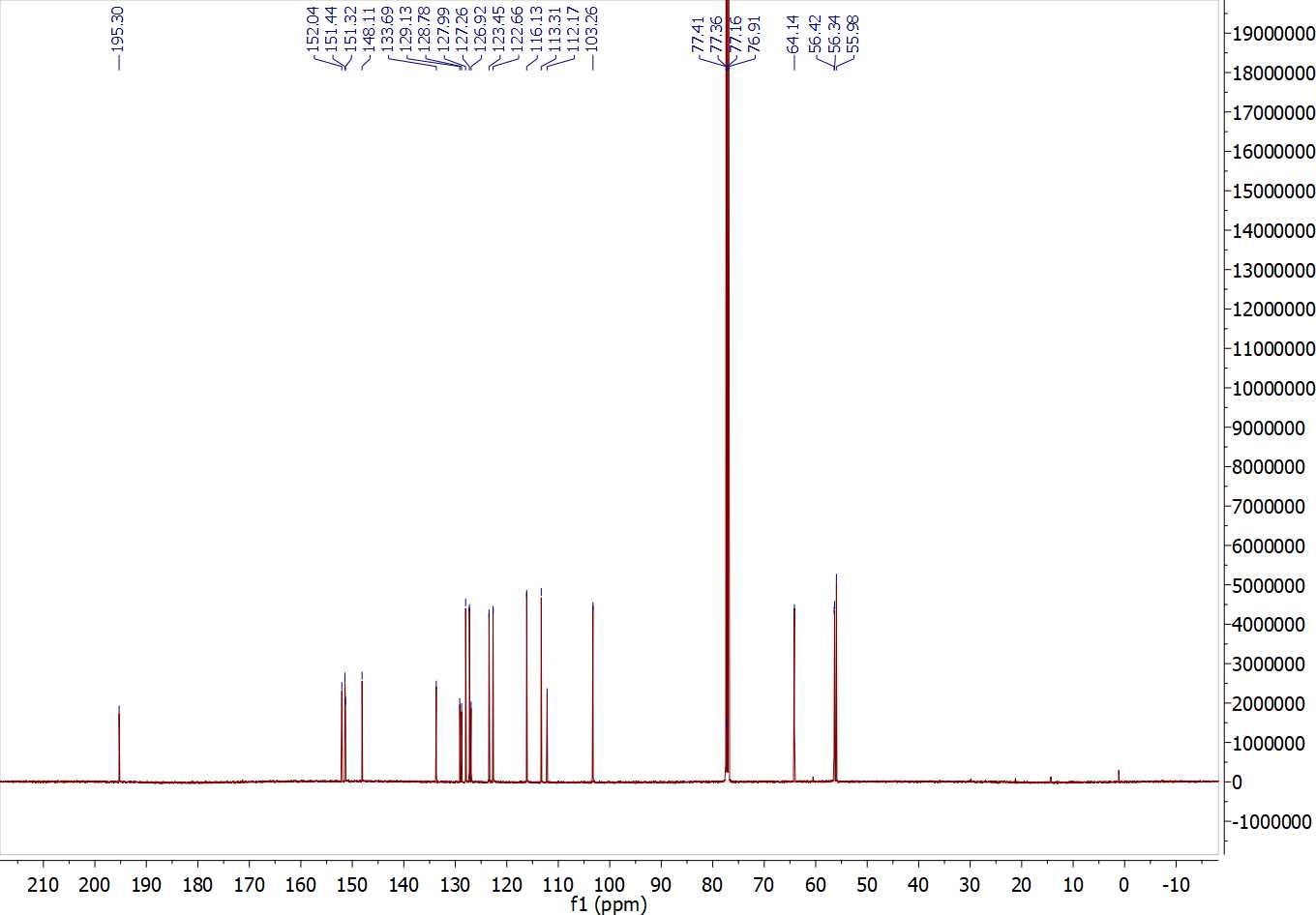


**Figure 6** ^13^C NMR (101 MHz, CDCl_3_) **4d** Br DiMeO dienophile precursor.


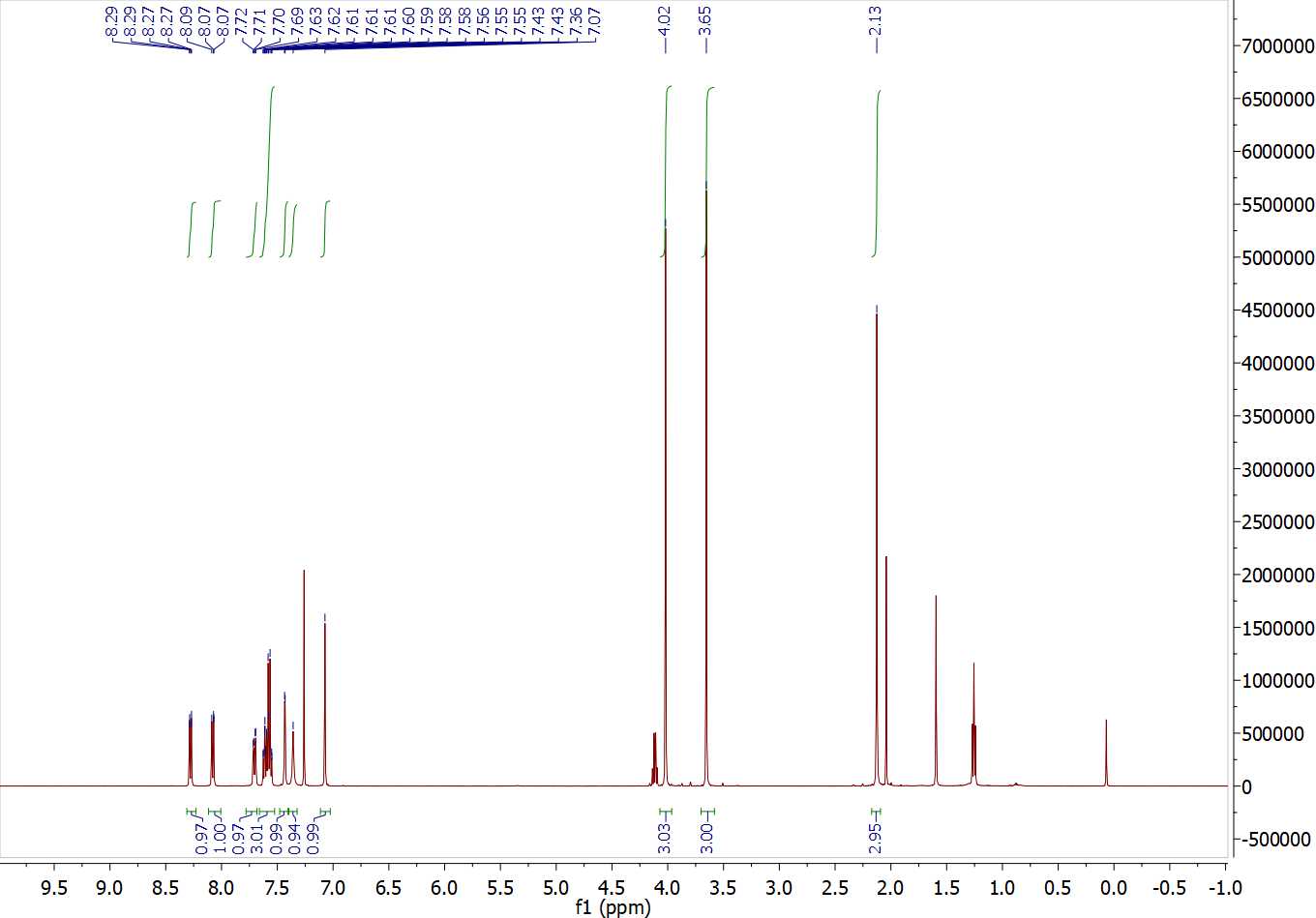


**Figure 7** ^1^H NMR (500 MHz, CDCl_3_) **4e** Br NAc dienophile precursor.


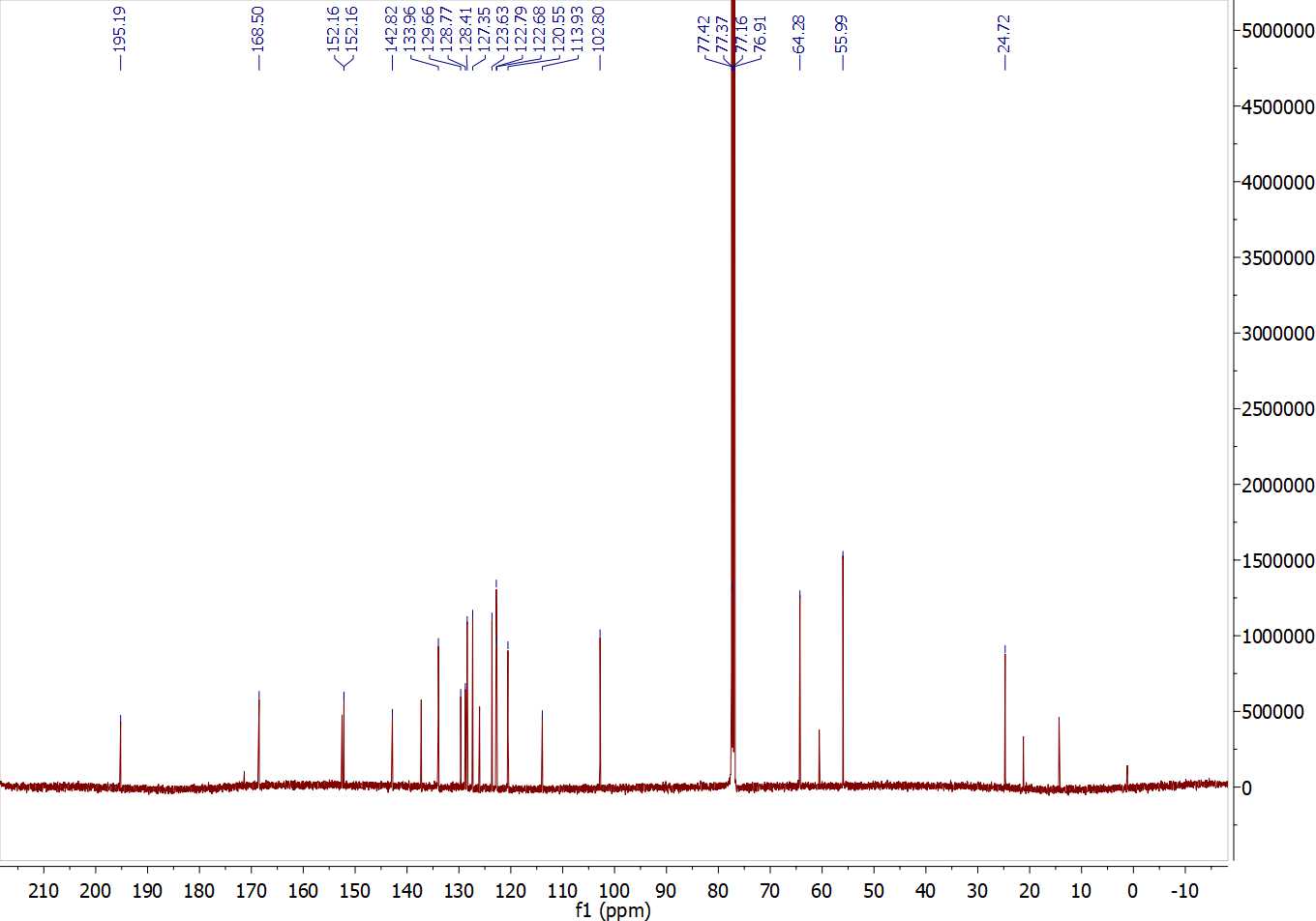


**Figure 8** ^13^C NMR (126 MHz, CDCl_3_) **4e** Br NAc dienophile precursor.


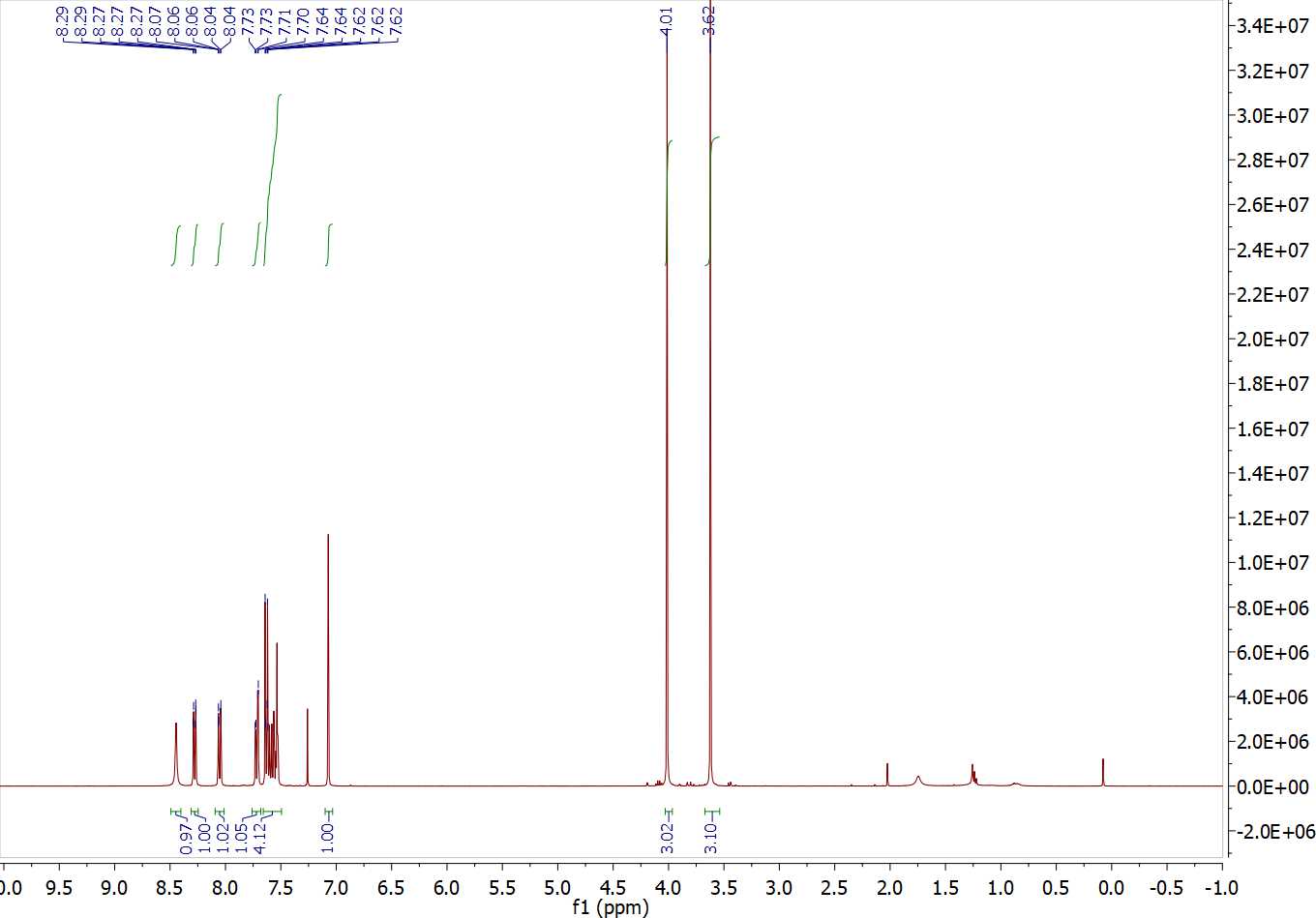


**Figure 9** ^1^H NMR (400 MHz, CDCl_3_) **4f** Br CF_3_ dienophile precursor.


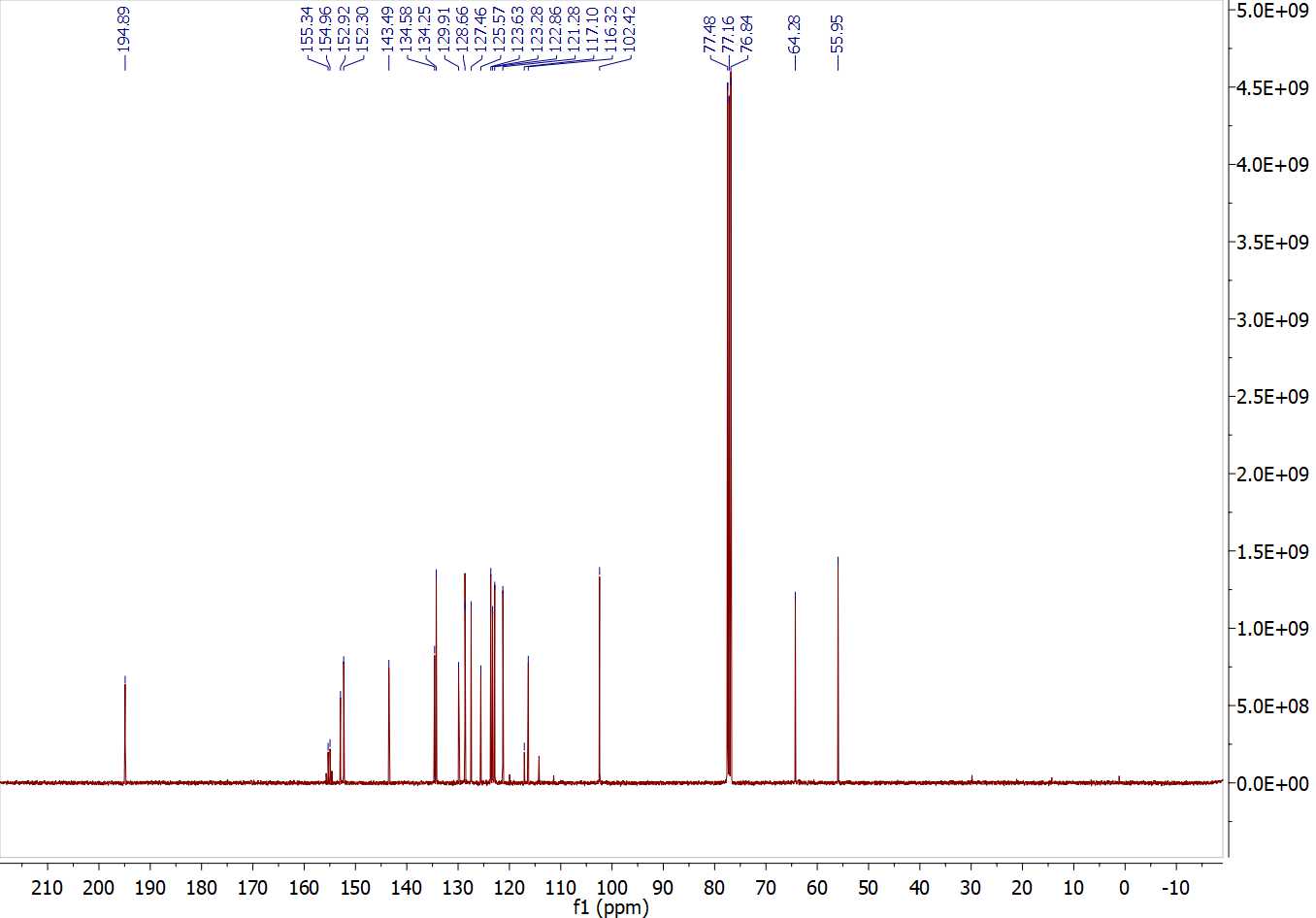


**Figure 10** ^13^C NMR (101 MHz, CDCl_3_) **4f** Br CF_3_ dienophile precursor.


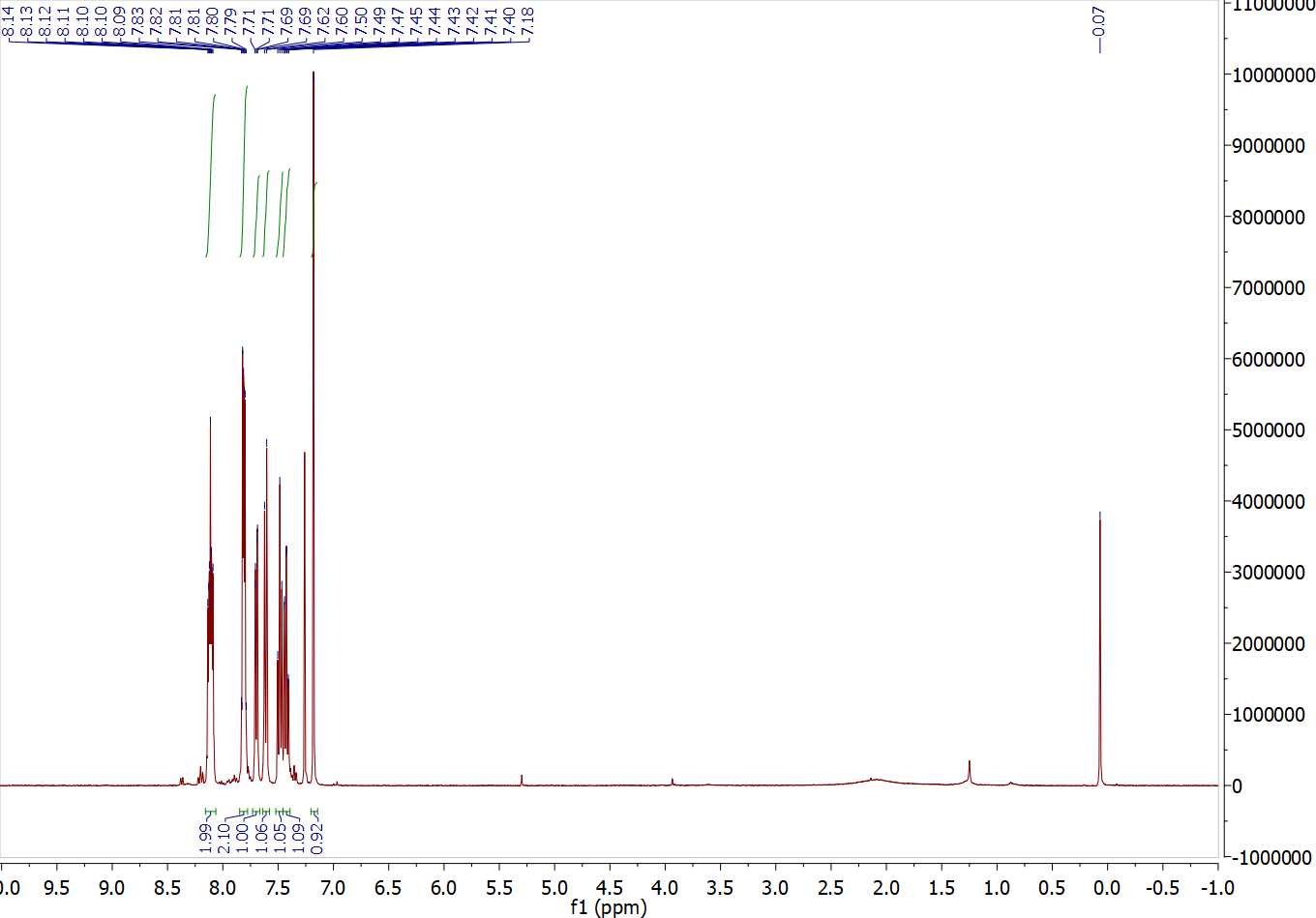


**Figure 11** ^1^H NMR (400 MHz, CDCl_3_) **5b** Br dienophile.


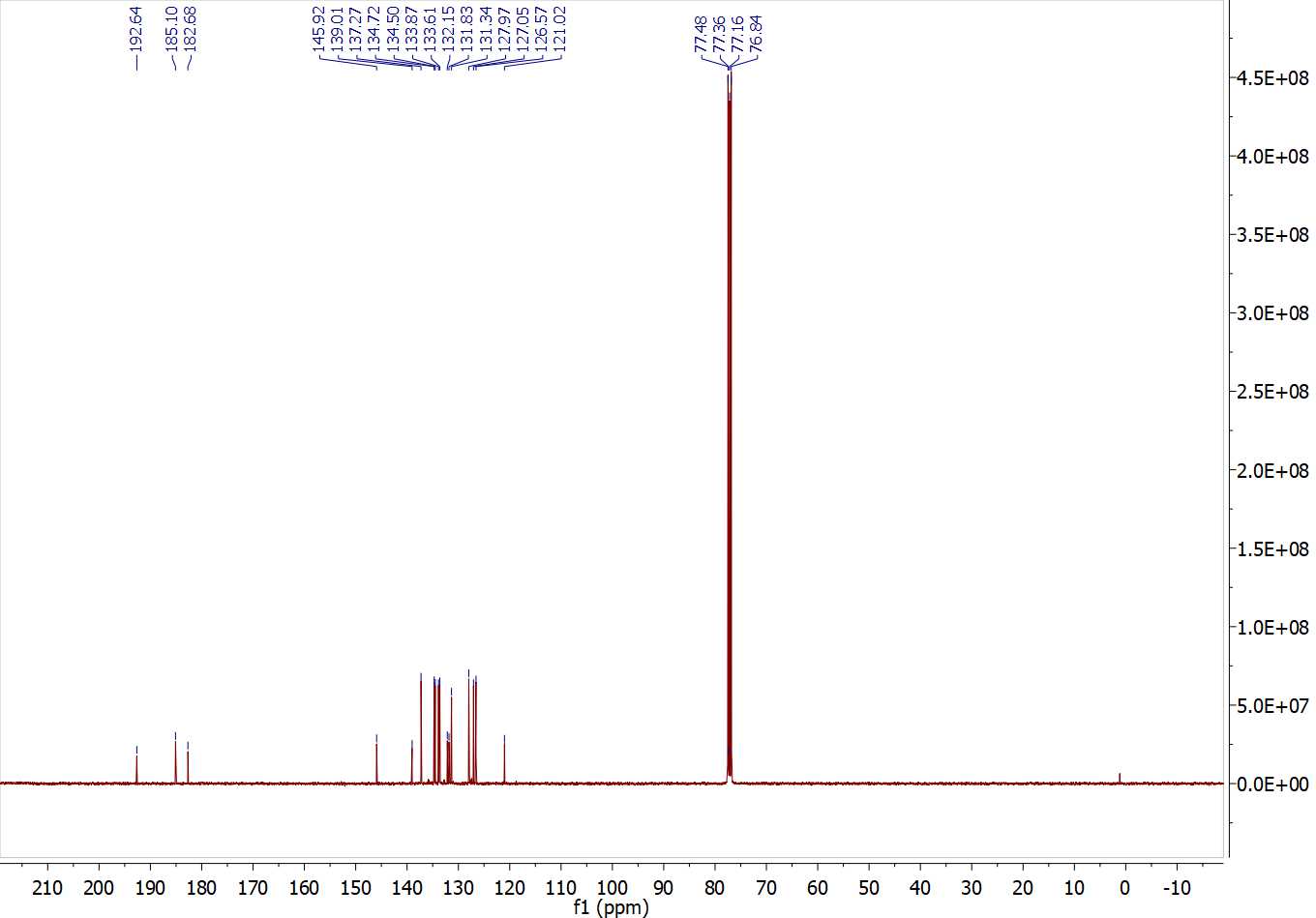


**Figure 12** ^13^C NMR (101 MHz, CDCl_3_) **5b** Br dienophile.


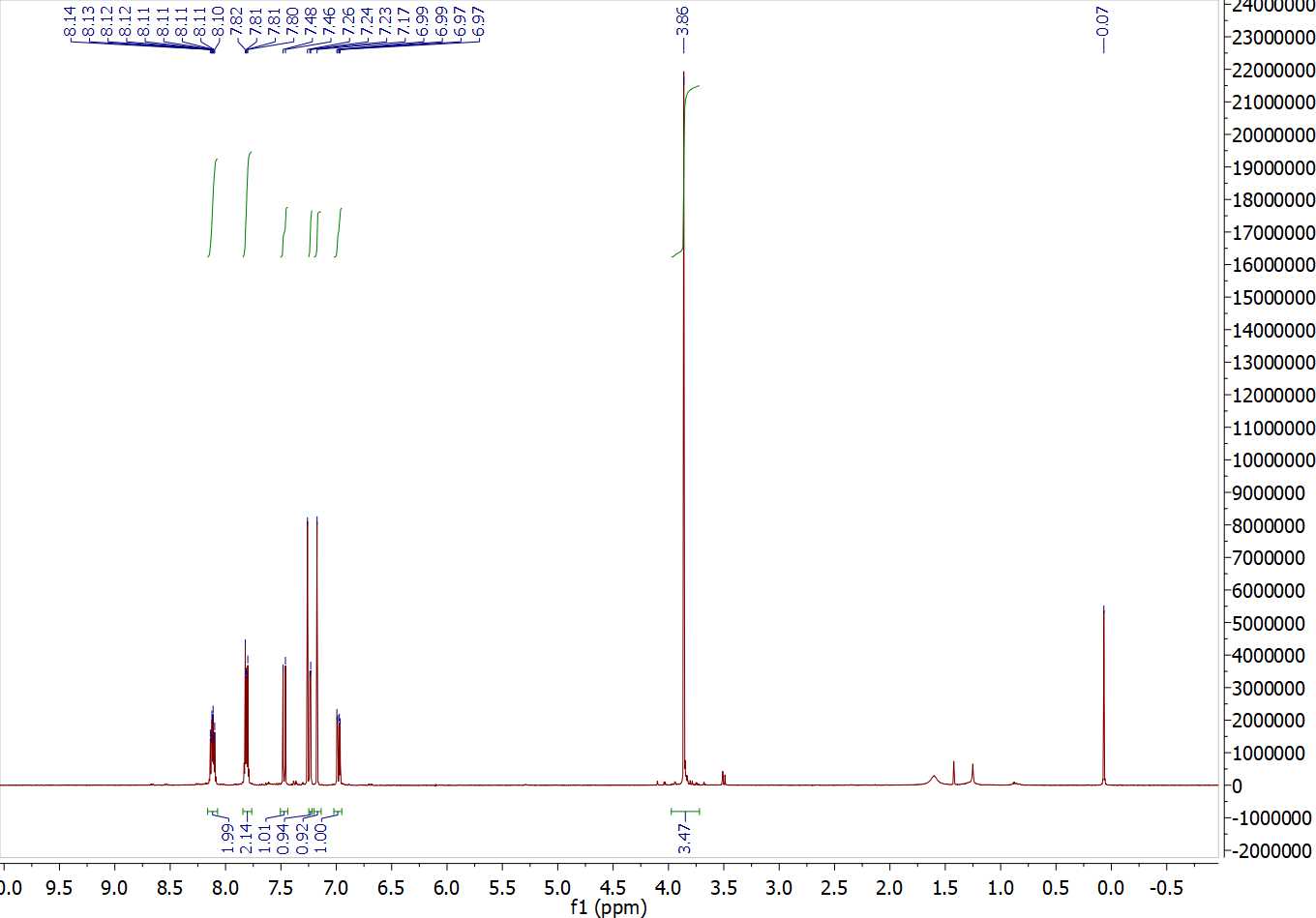


**Figure 13** ^1^H NMR (400 MHz, CDCl_3_) **5c** Br MeO dienophile.


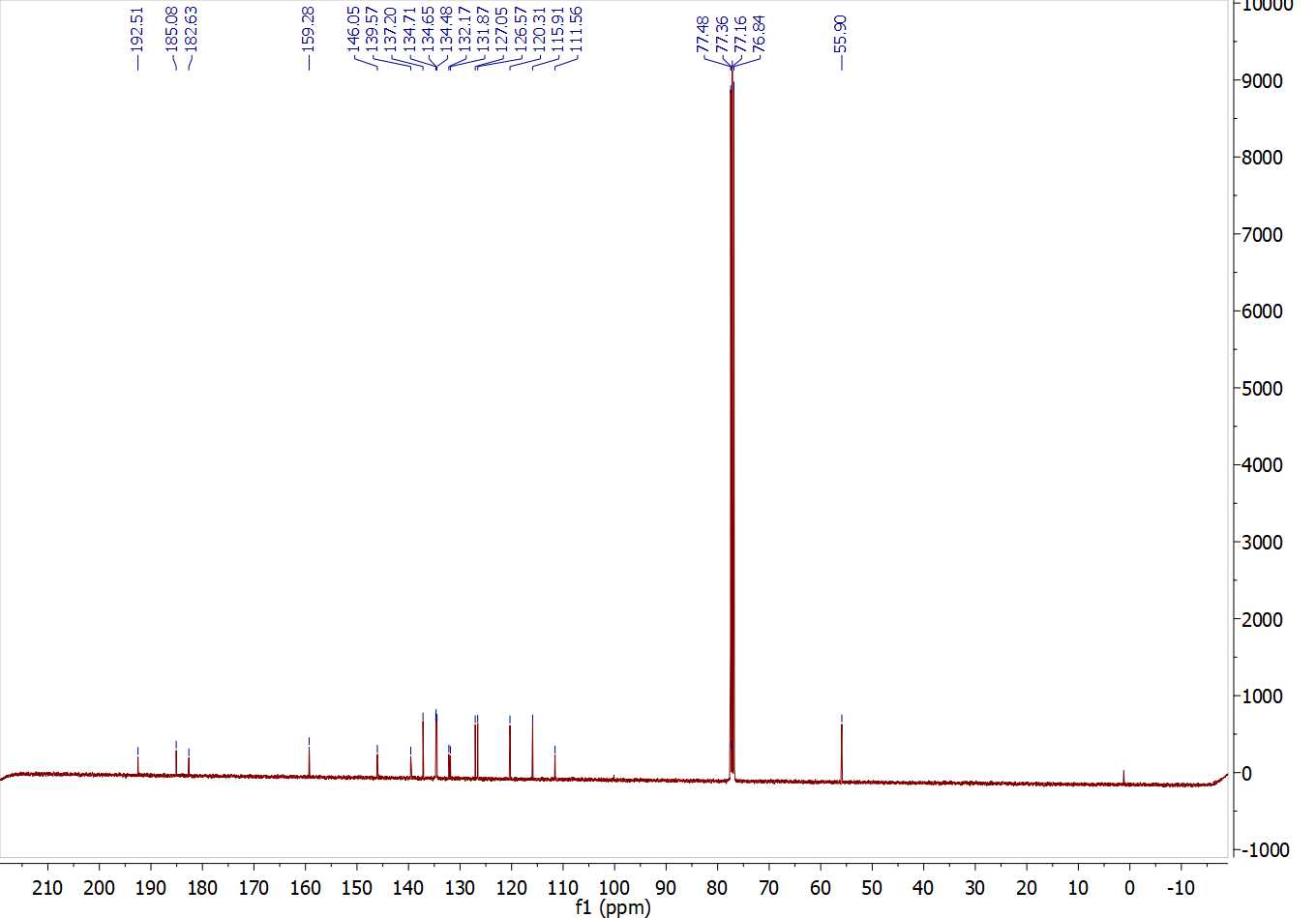


**Figure 14** ^13^C NMR (101 MHz, CDCl_3_) **5c** Br MeO dienophile.


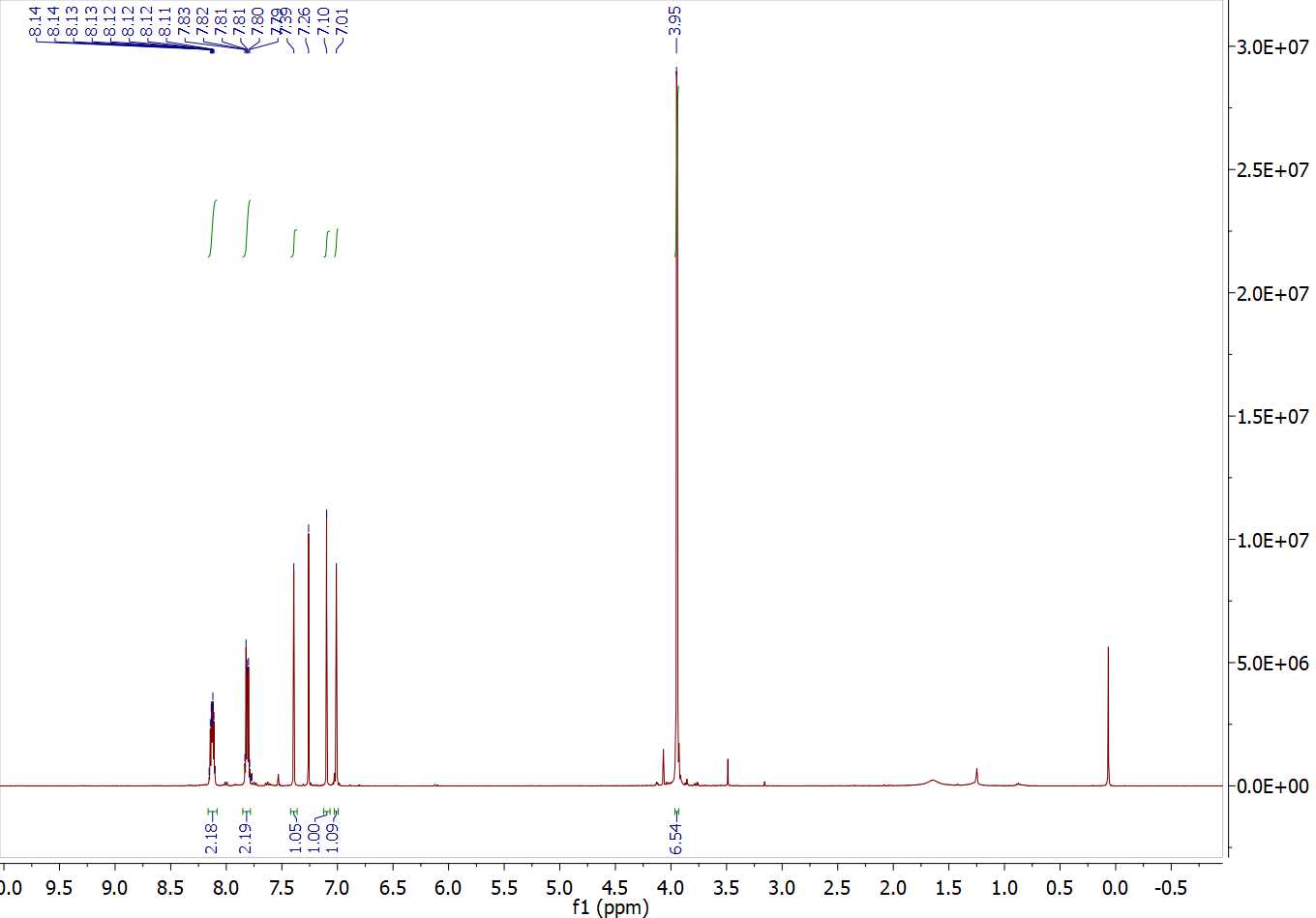


**Figure 15** ^1^H NMR (400 MHz, CDCl_3_) **5d** Br DiMeO dienophile.


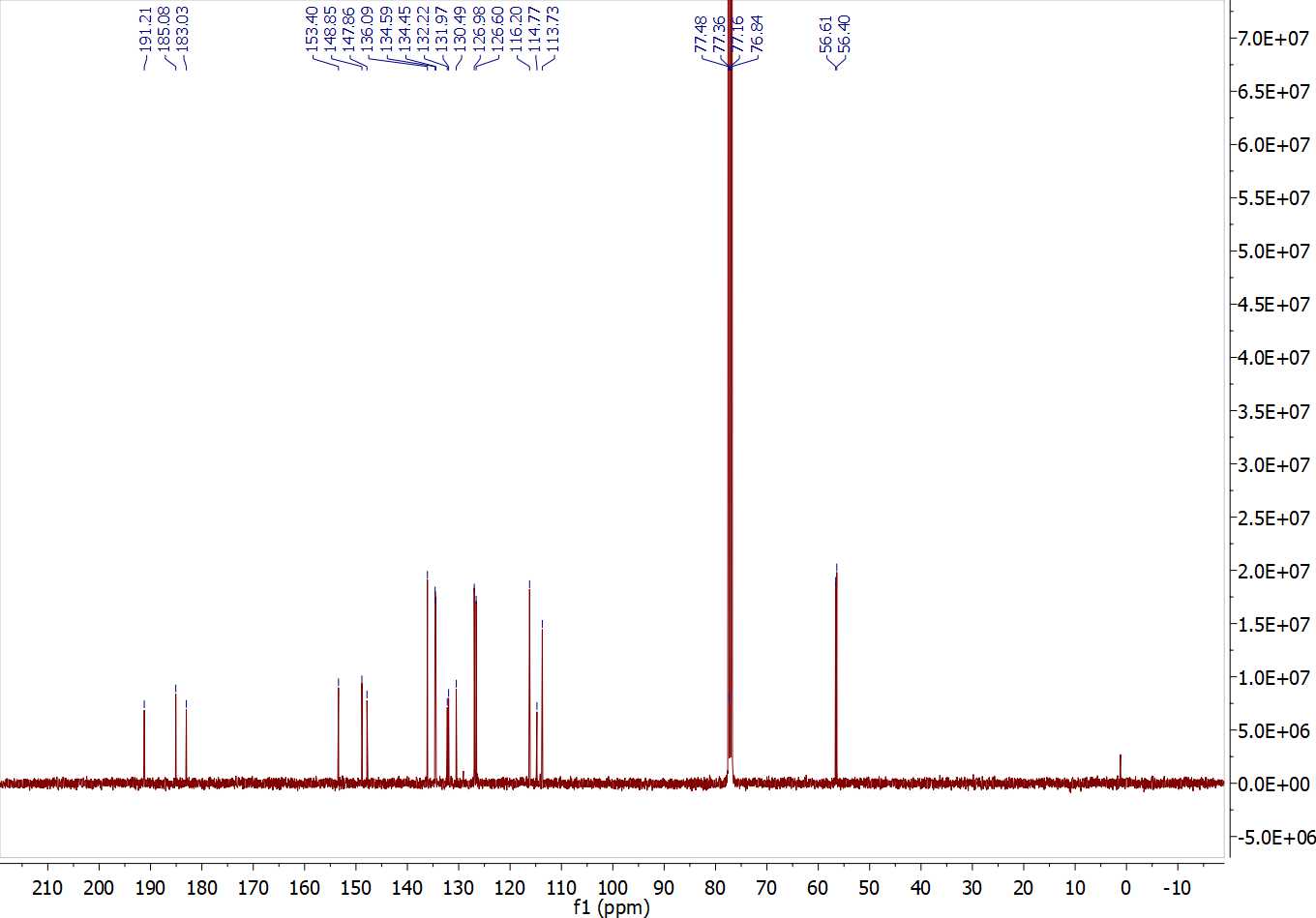


**Figure 16** ^13^C NMR (101 MHz, CDCl_3_) **5d** Br DiMeO dienophile.


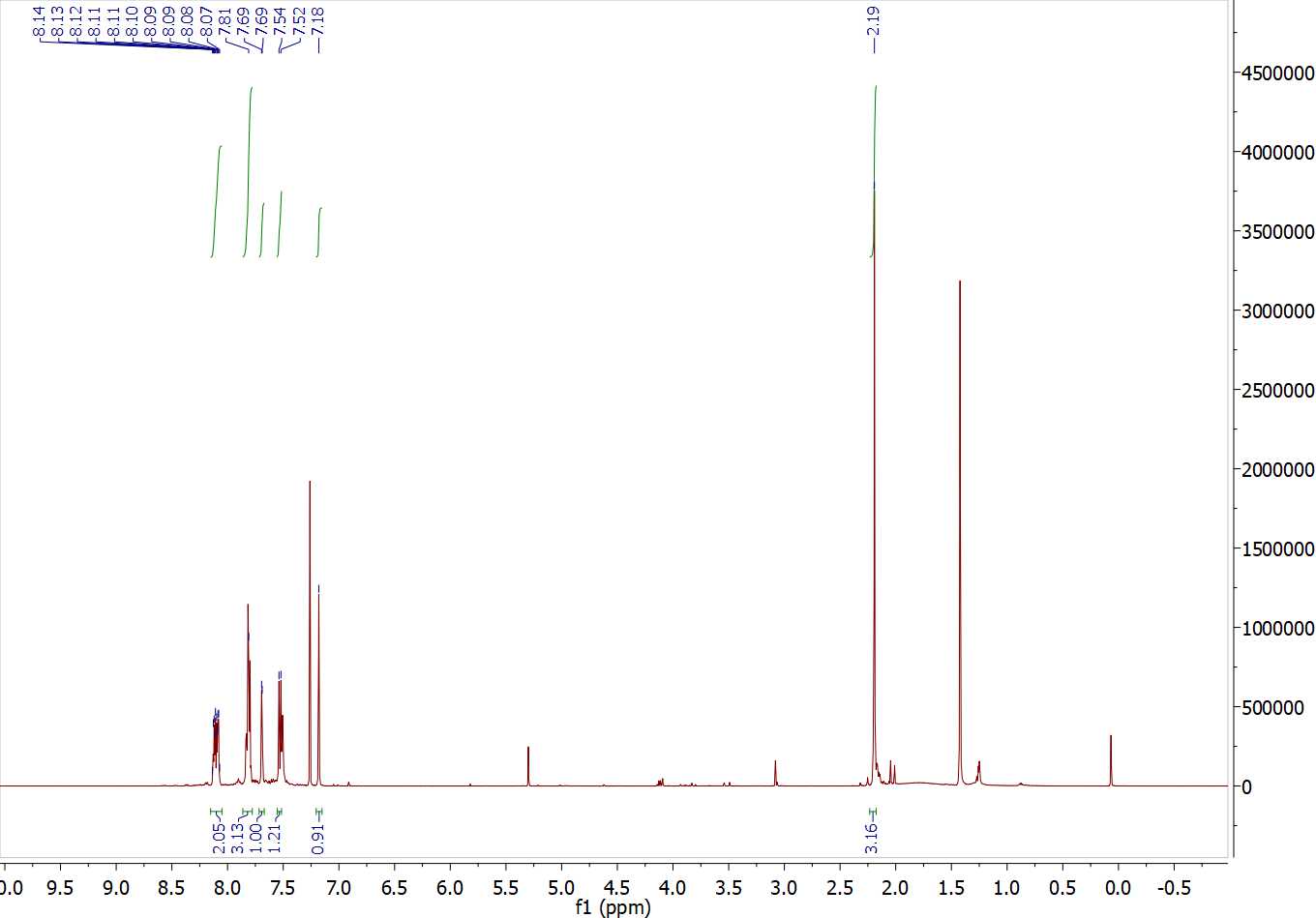


**Figure 17** ^1^H NMR (500 MHz, CDCl_3_) **5f** Br NAc dienophile.


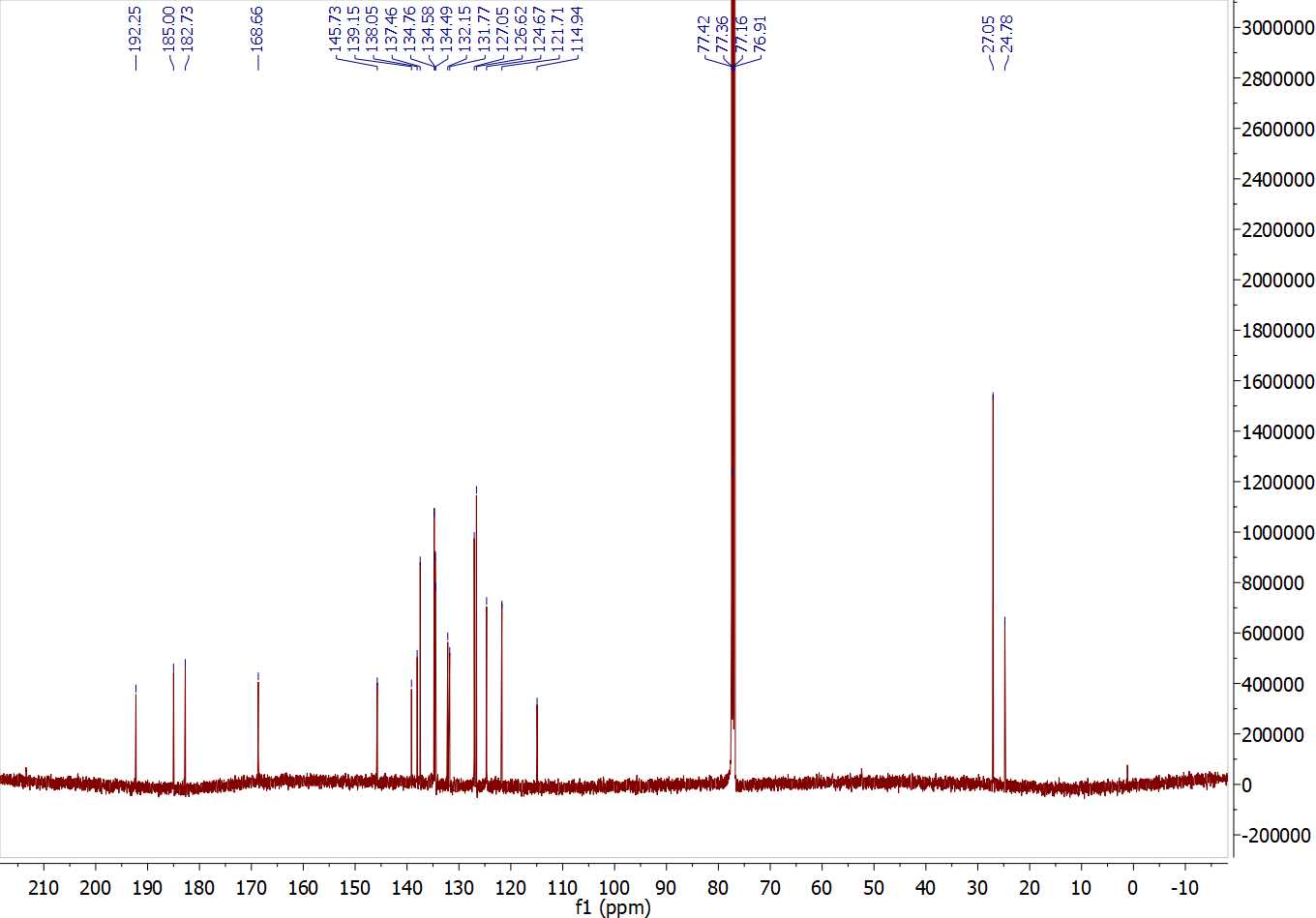


**Figure 18** ^13^C NMR (126 MHz, CDCl_3_) **5f** Br NAc dienophile.


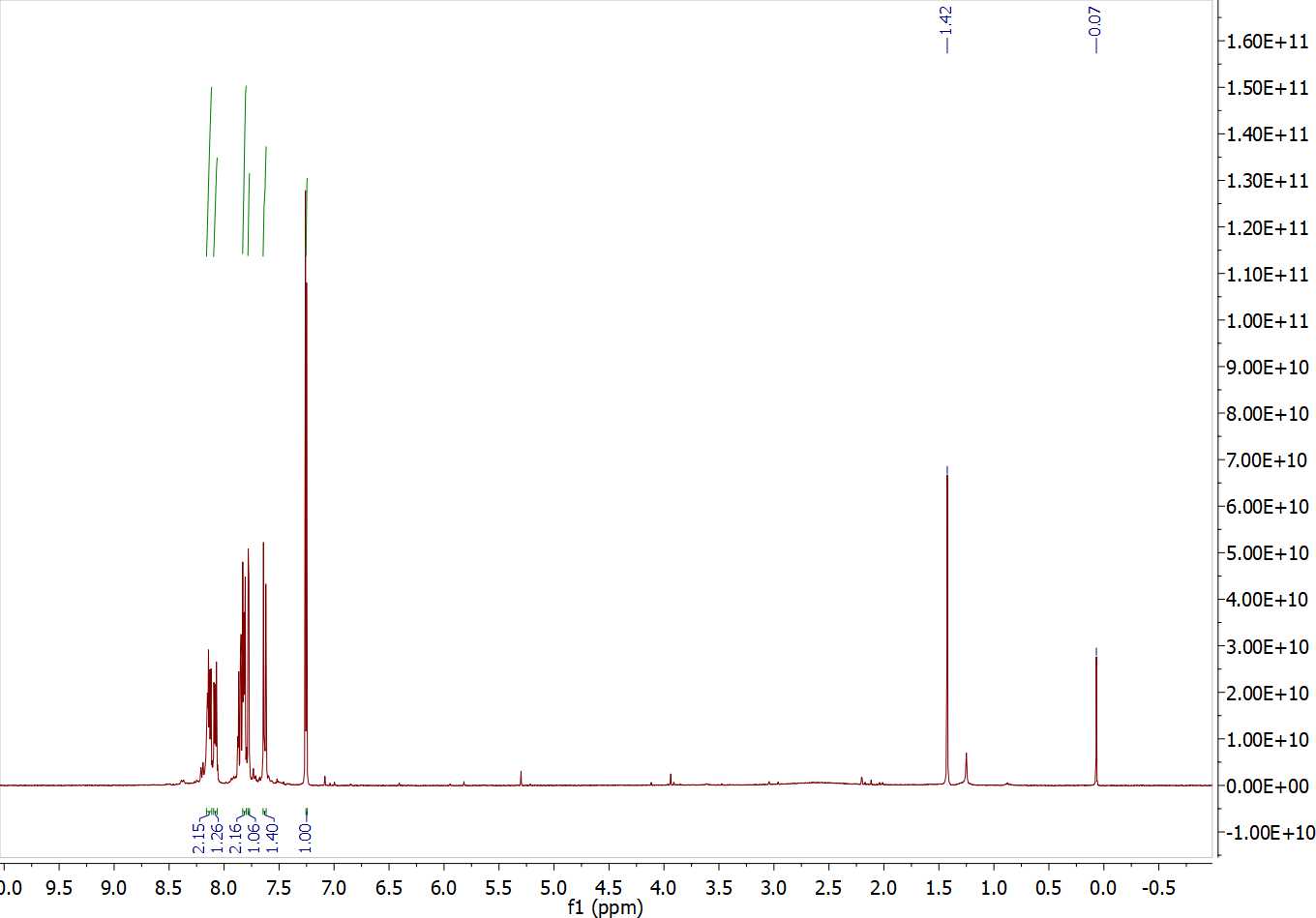


**Figure 19** ^1^H NMR (400 MHz, CDCl_3_) **5g** Br CF_3_ dienophile.


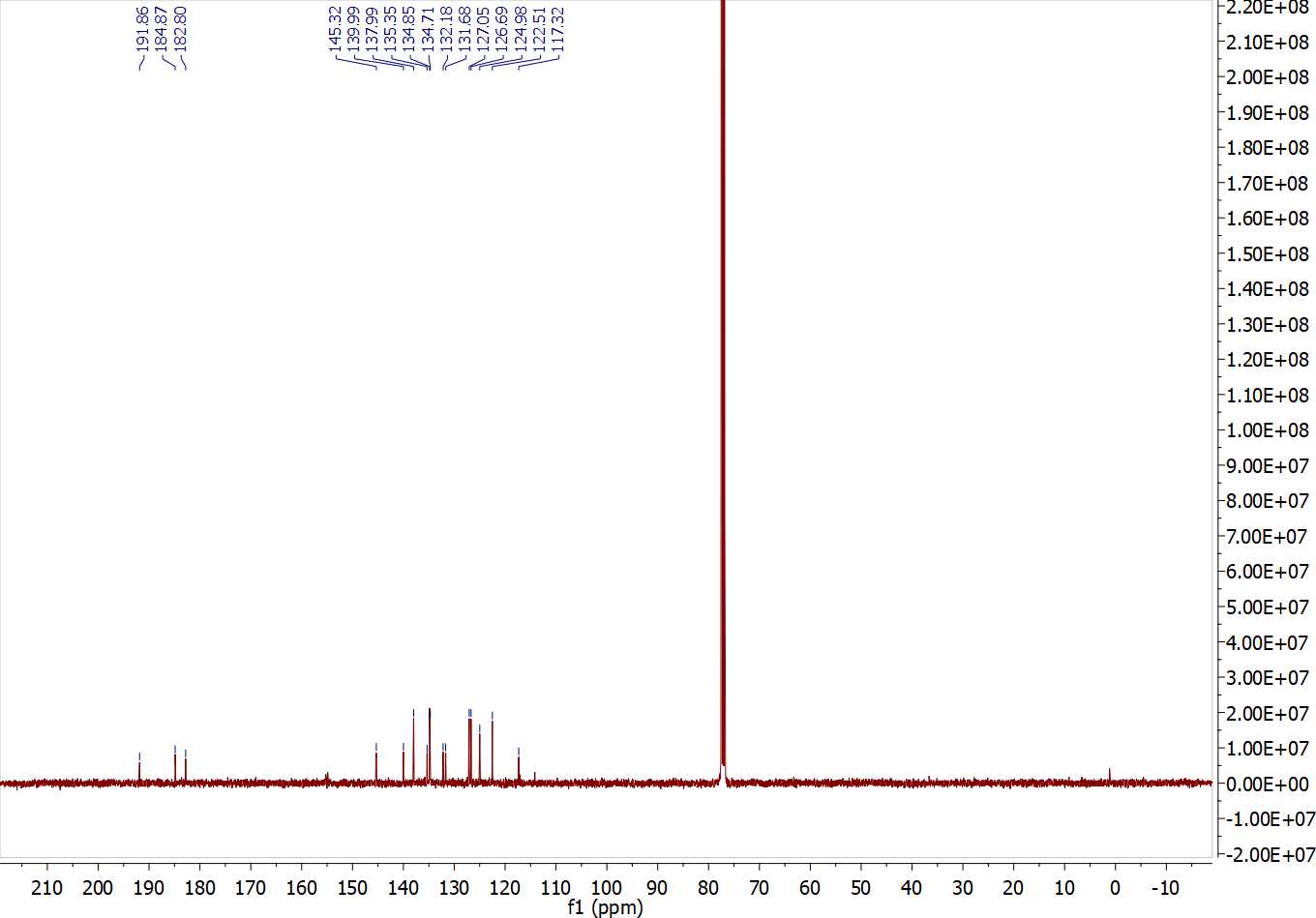


**Figure 20** ^13^C NMR (101 MHz, CDCl_3_) **5g** Br CF_3_ dienophile.


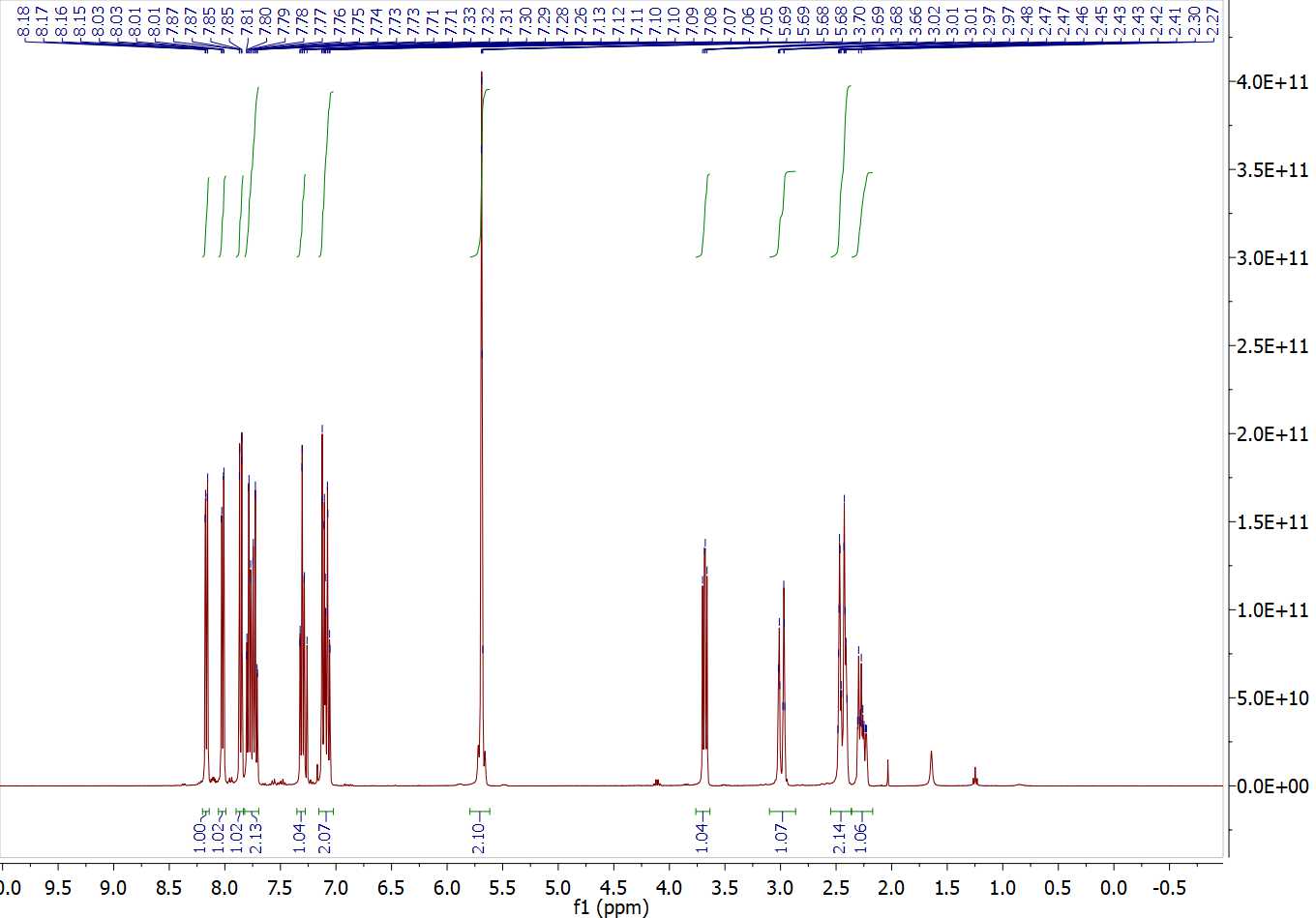


**Figure 21** ^1^H NMR (400 MHz, CDCl_3_) **8aa** Iodine Sulfolene DA.


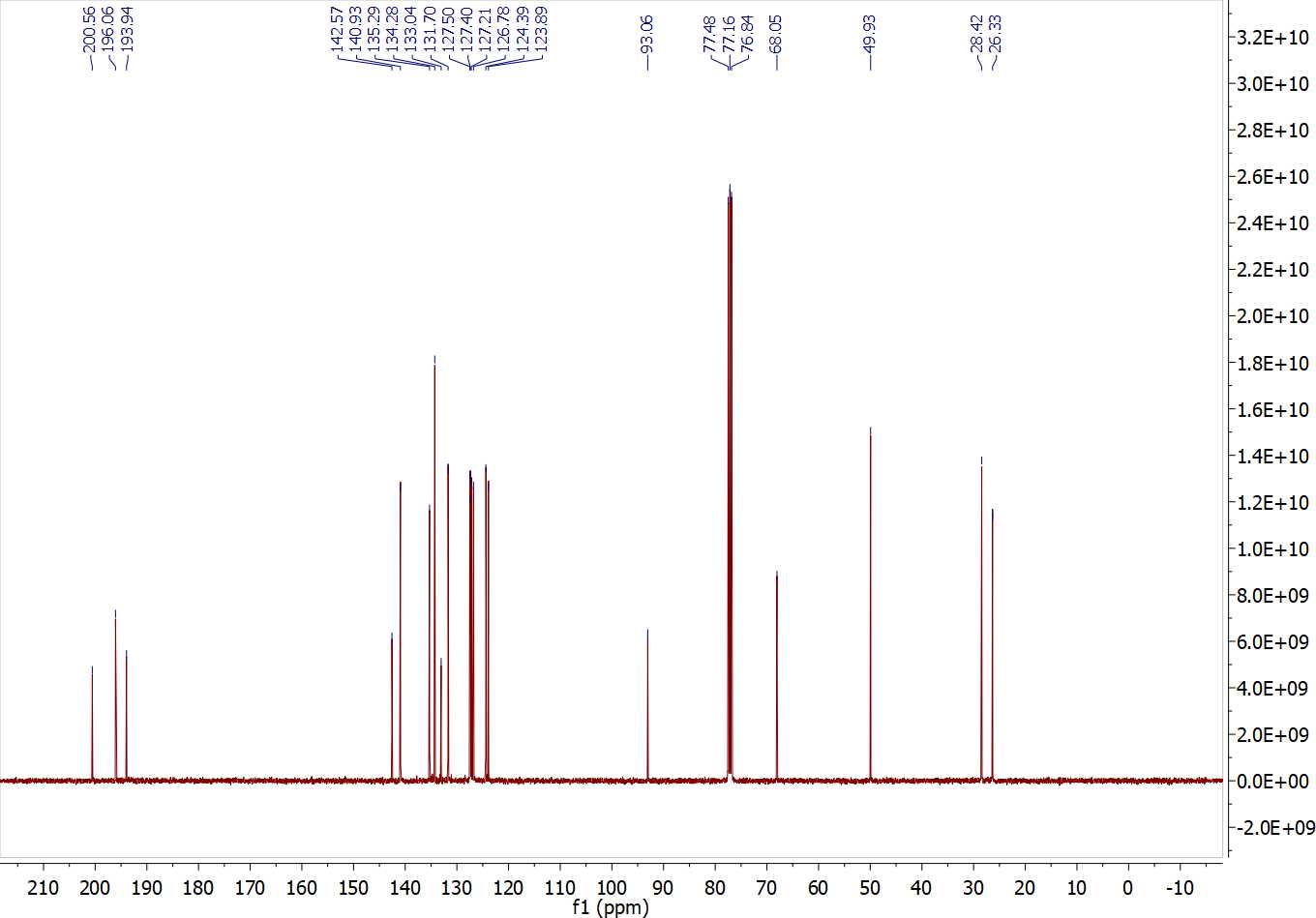


**Figure 22** ^13^C NMR (101 MHz, CDCl_3_) **8aa** Iodine Sulfolene DA.


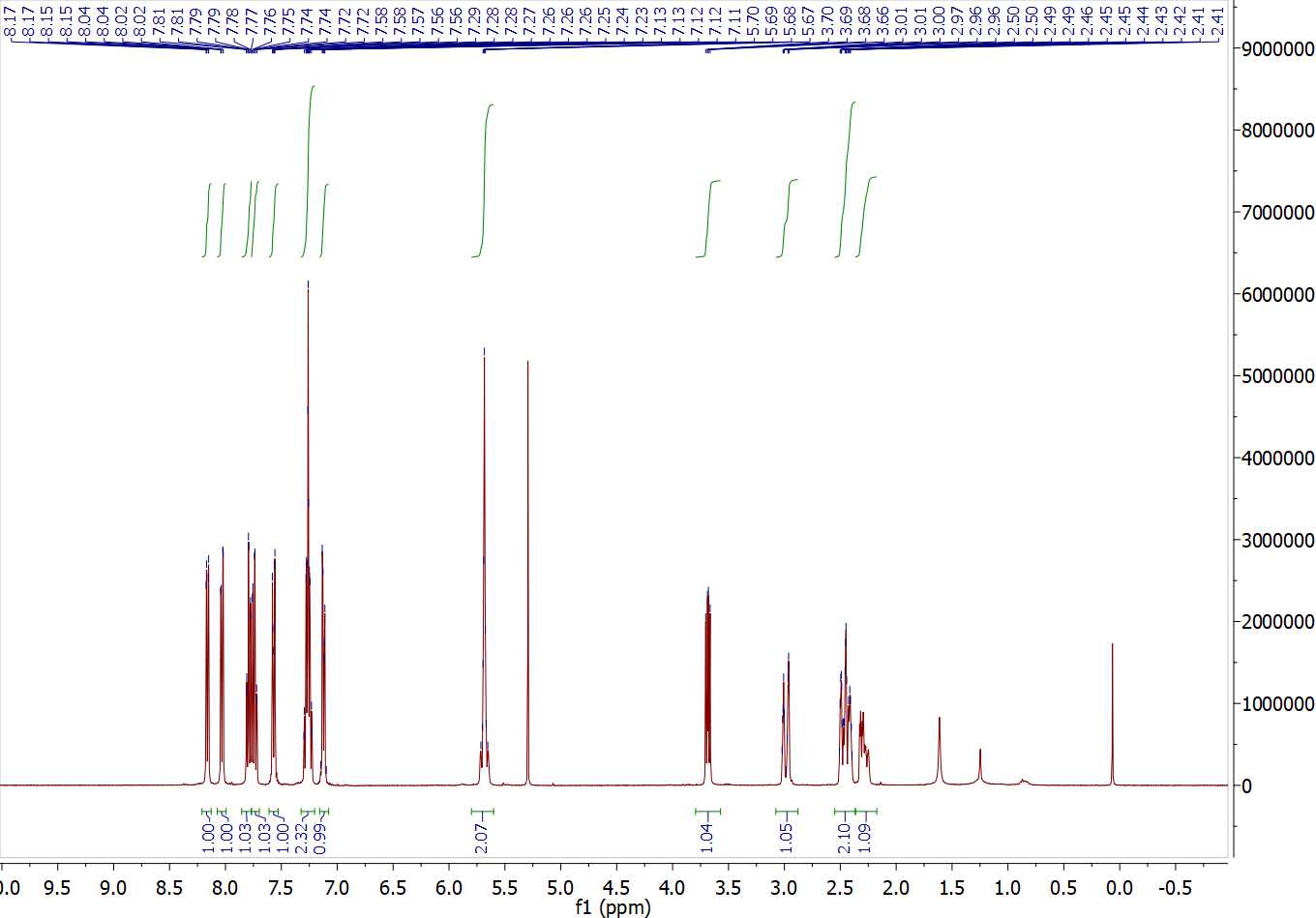


**Figure 23** ^1^H NMR (400 MHz, CDCl_3_) **8ba** Br Sulfolene DA.


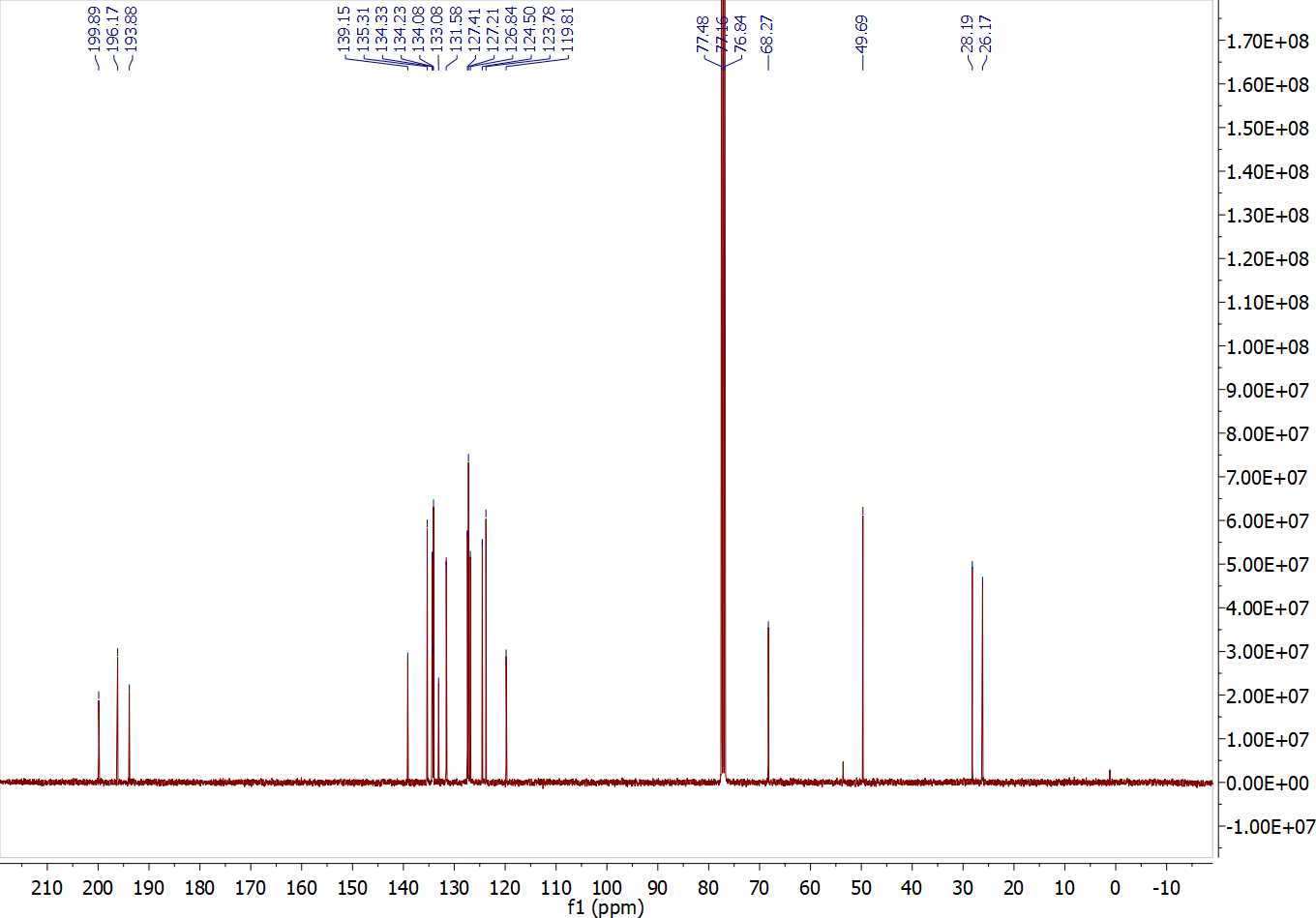


**Figure 24** ^13^C NMR (101 MHz, CDCl_3_) **8ba** Br Sulfolene DA.


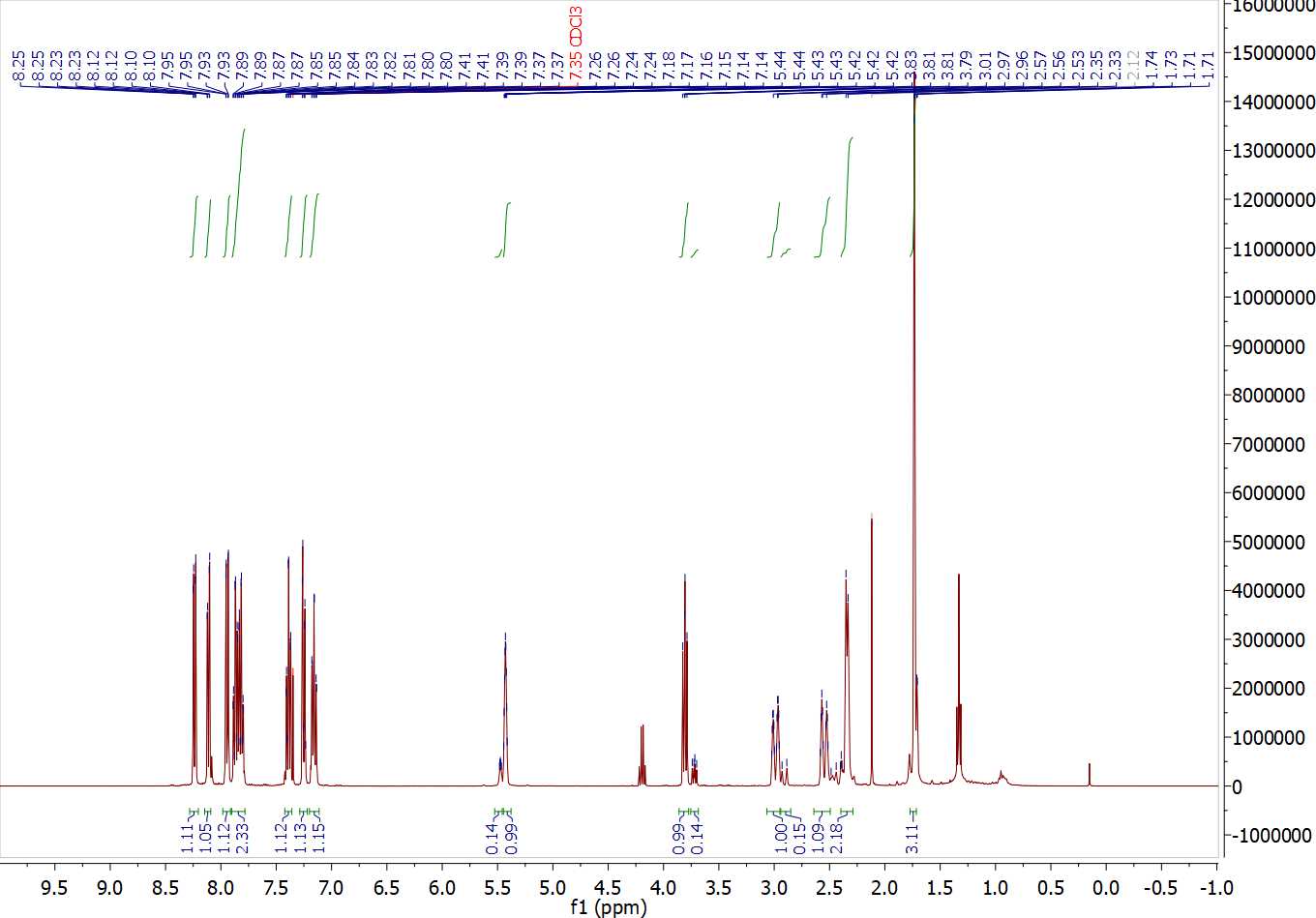


**Figure 25** ^1^H NMR (400 MHz, CDCl_3_) **8ab** Iodine Isoprene DA.


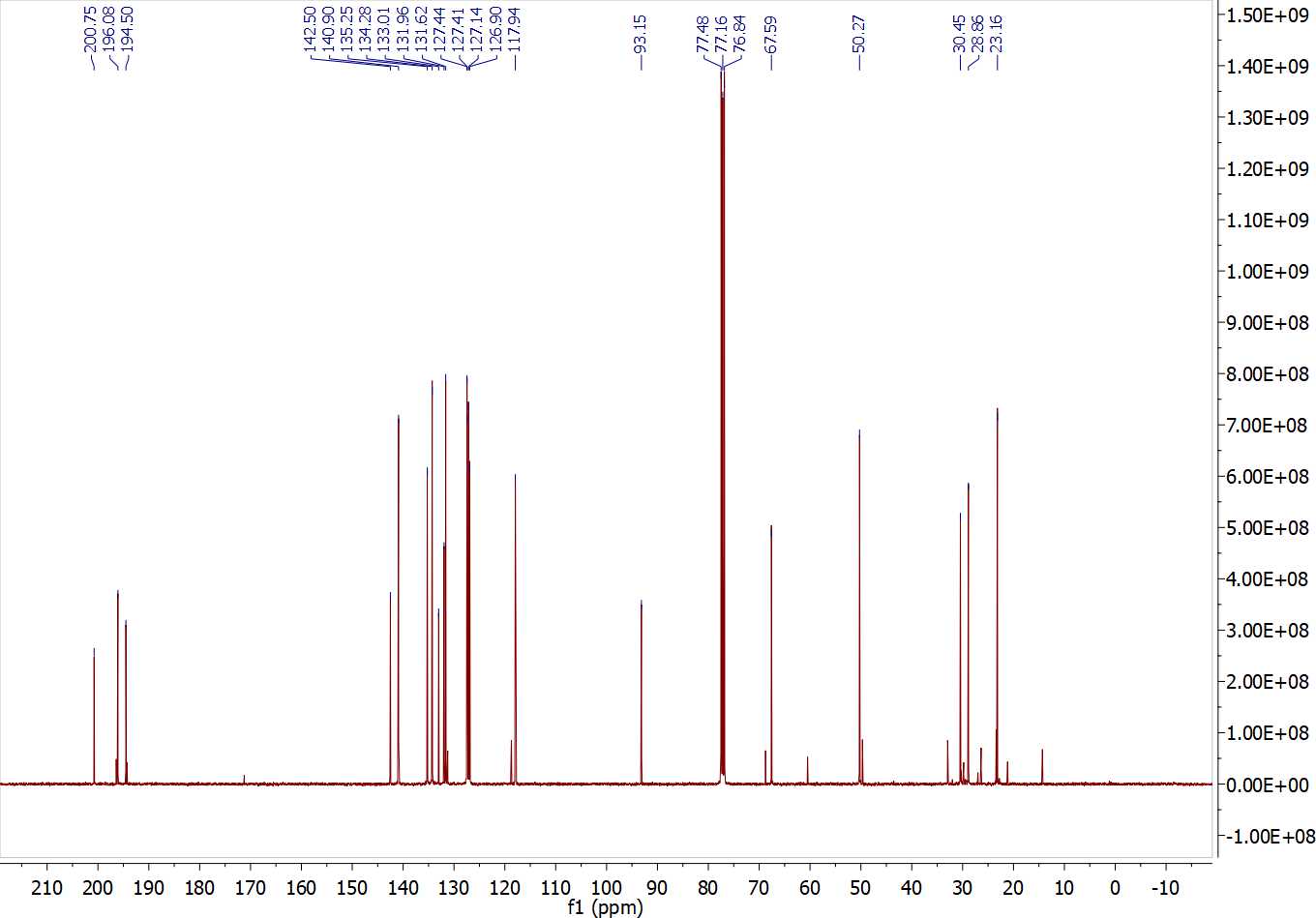


**Figure 26** ^13^C NMR (101 MHz, CDCl_3_) **8ab** Iodine Isoprene DA.


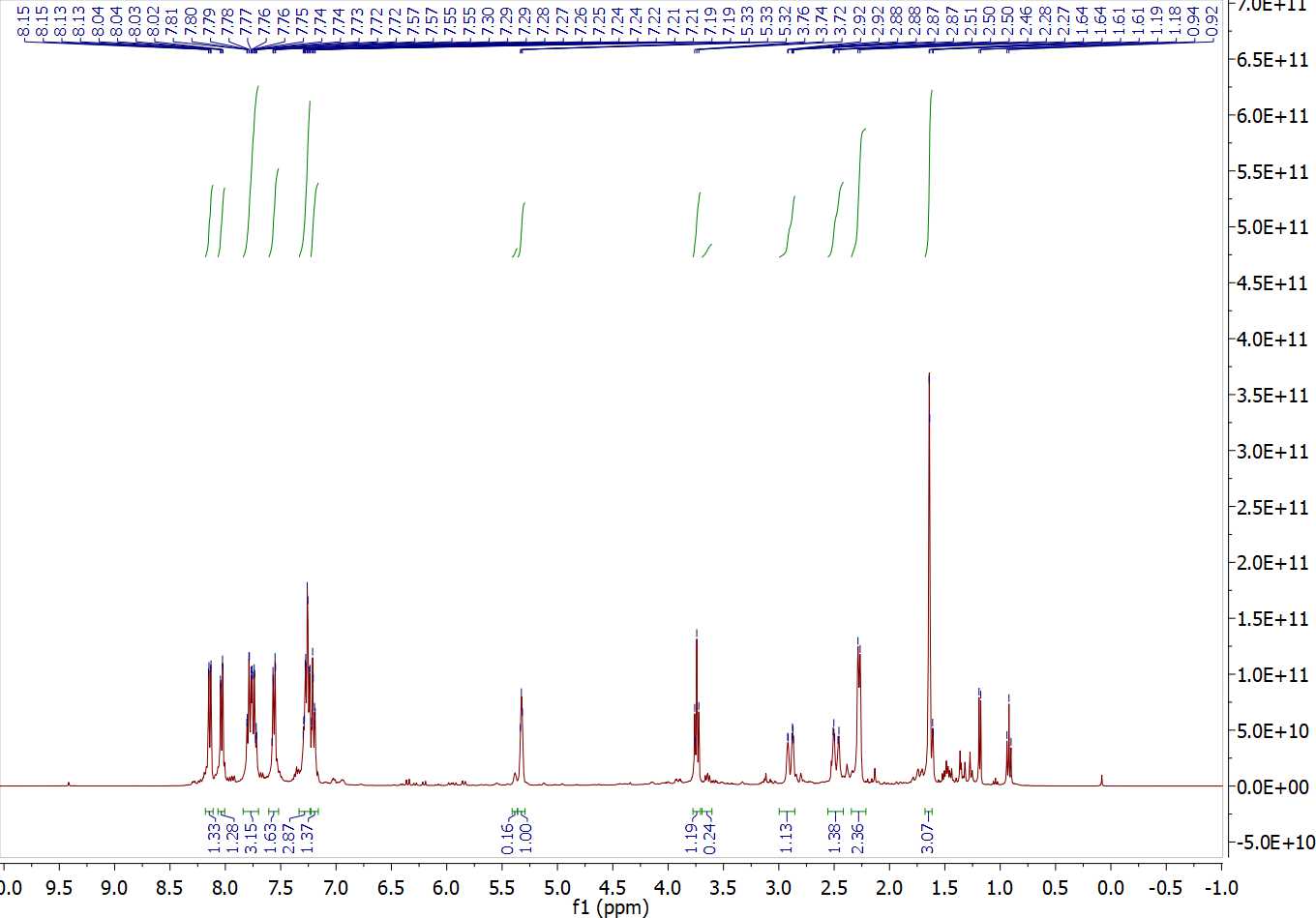


**Figure 27** ^1^H NMR (400 MHz, CDCl_3_) **8bb** Br Isoprene DA.


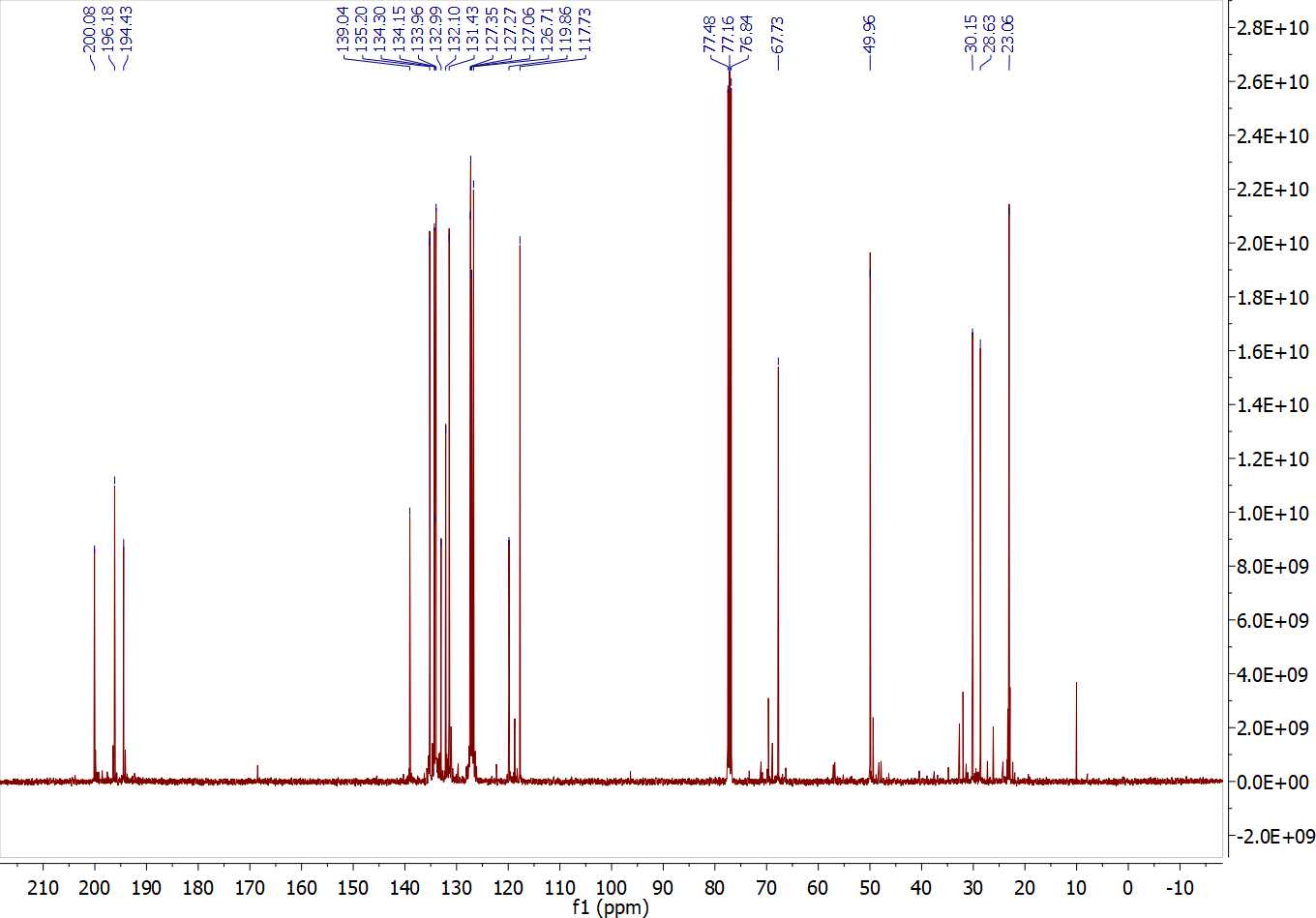


**Figure 28** ^13^C NMR (101 MHz, CDCl_3_) **8bb** Br Isoprene DA.


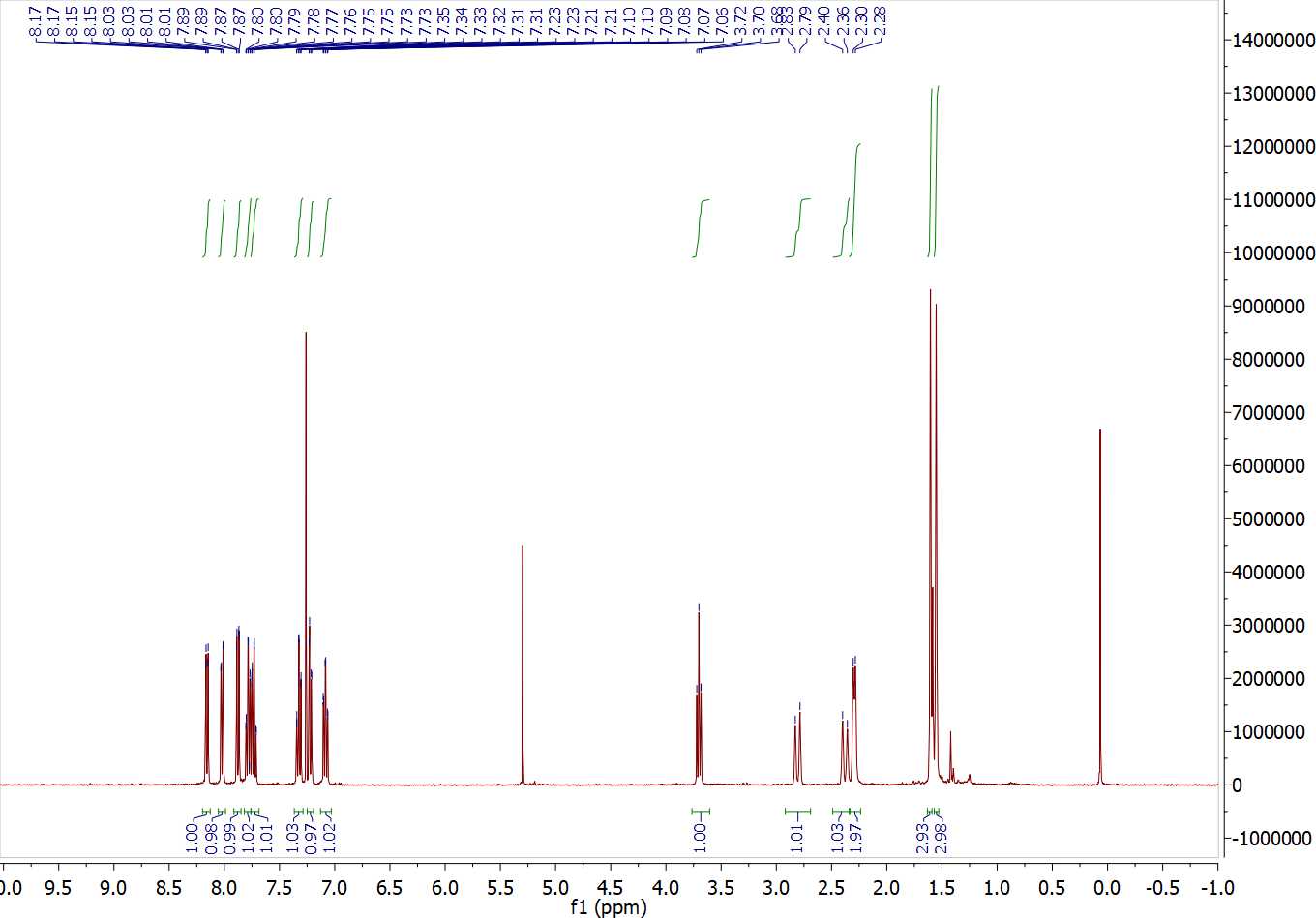


**Figure 29** ^1^H NMR (400 MHz, CDCl_3_) **8ac** Iodine Dimethyl DA.


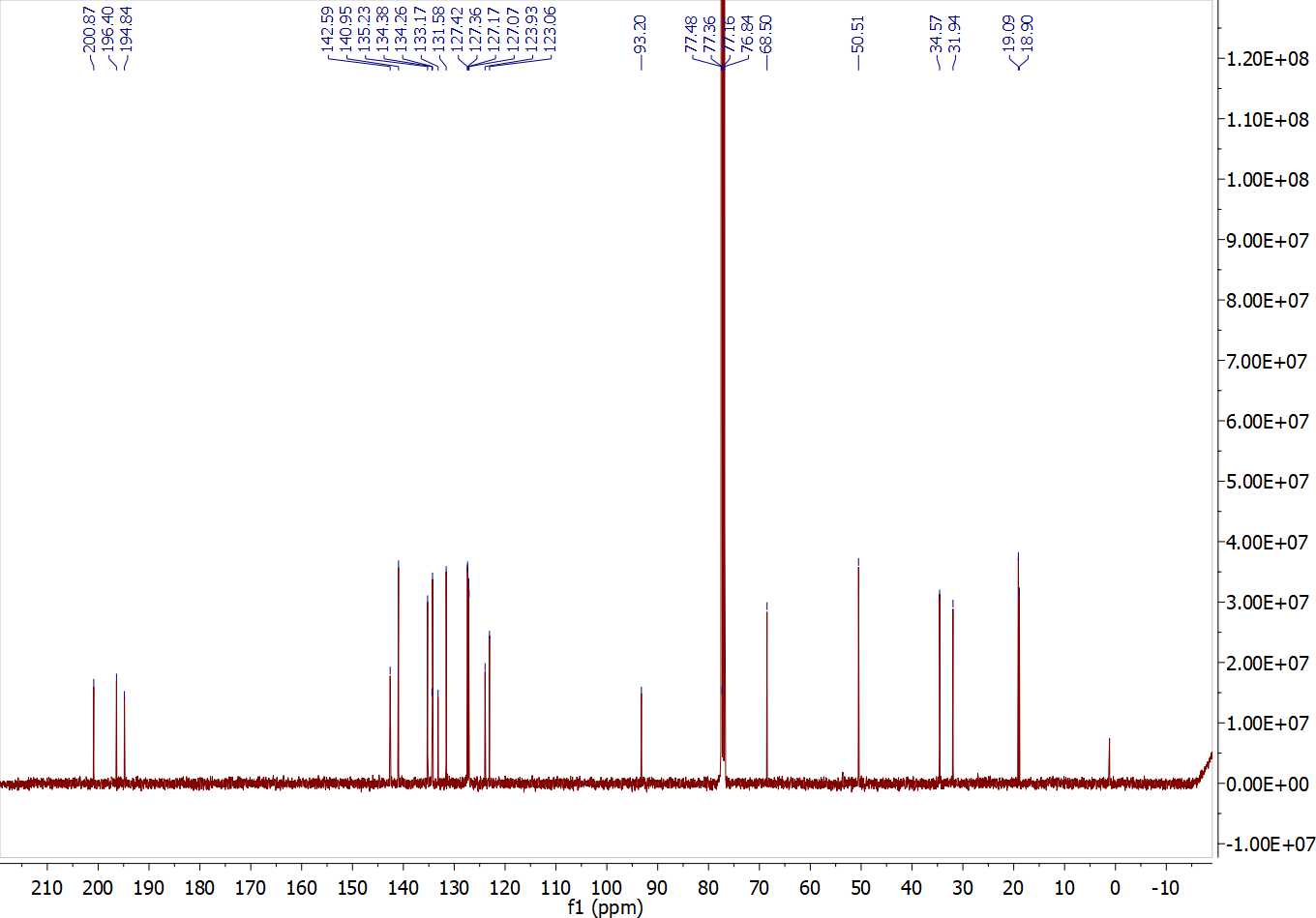


**Figure 30** ^13^C NMR (101 MHz, CDCl_3_) **8ac** Iodine Dimethyl DA.


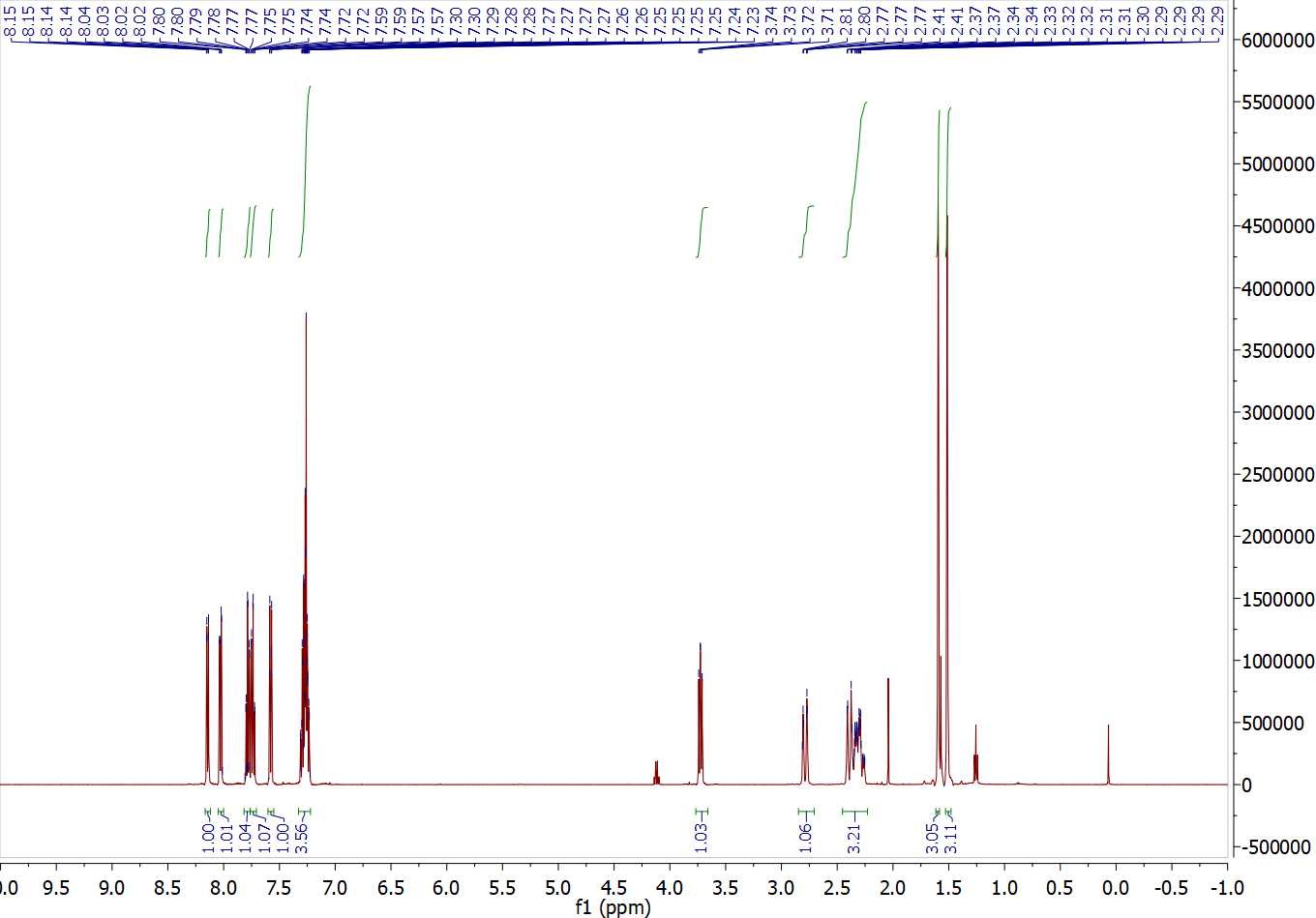


**Figure 31** ^1^H NMR (500 MHz, CDCl_3_) **8bc** Br Dimethyl DA.


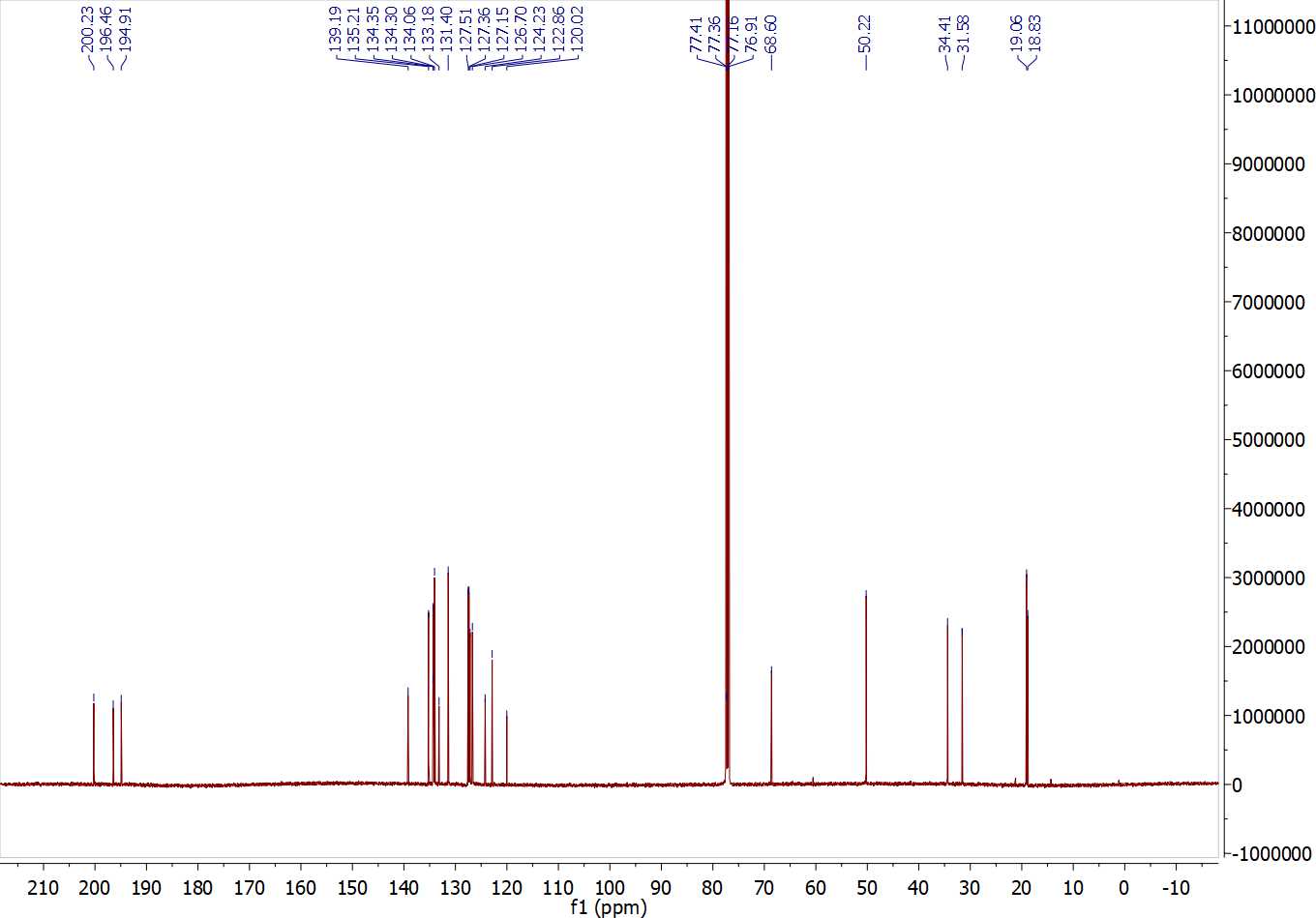


**Figure 32** ^13^C NMR (126 MHz, CDCl_3_) **8bc** Br Dimethyl DA.


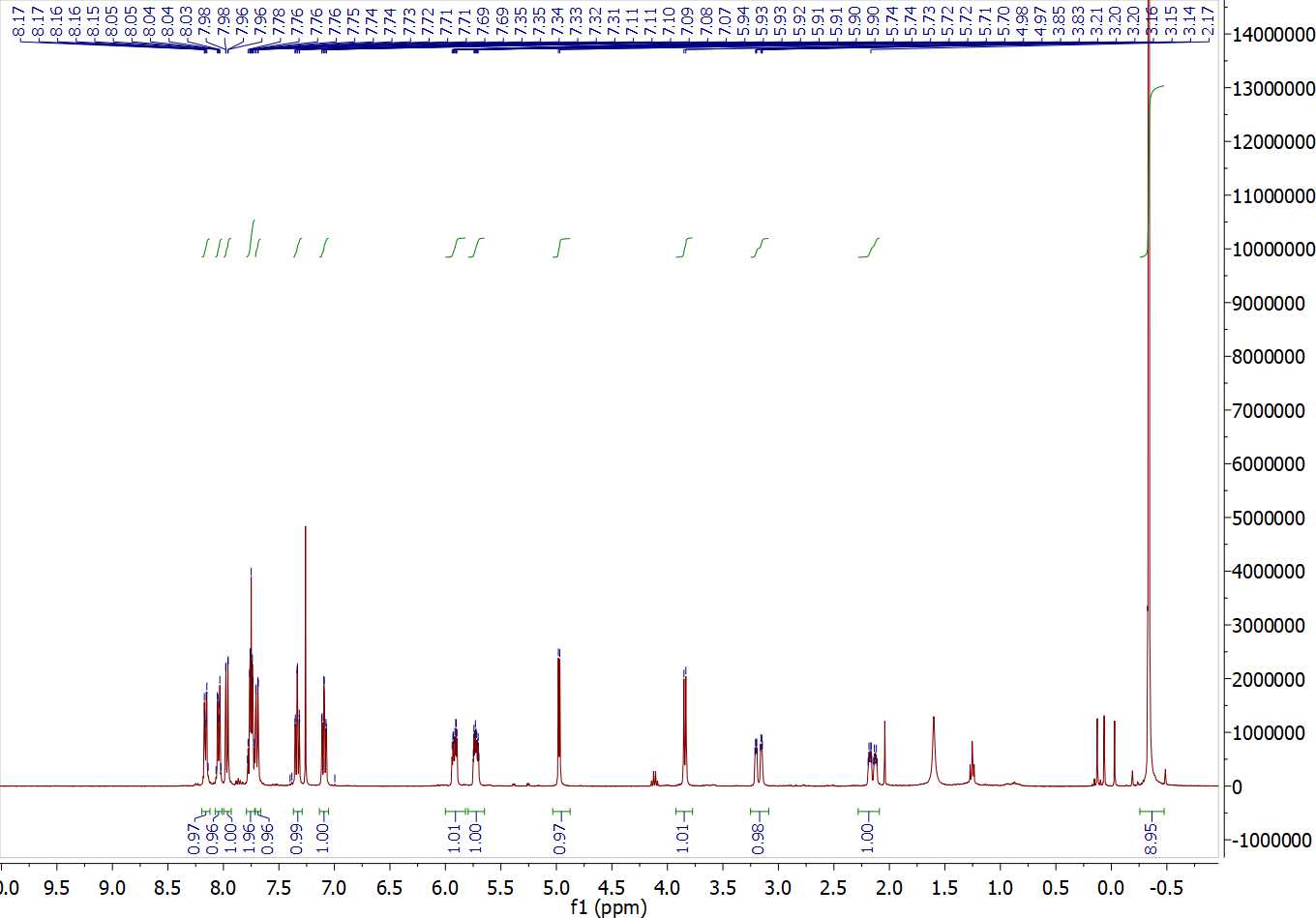


**Figure 33** ^1^H NMR (400 MHz, CDCl_3_) **9ad** Iodine TMS DA F1.


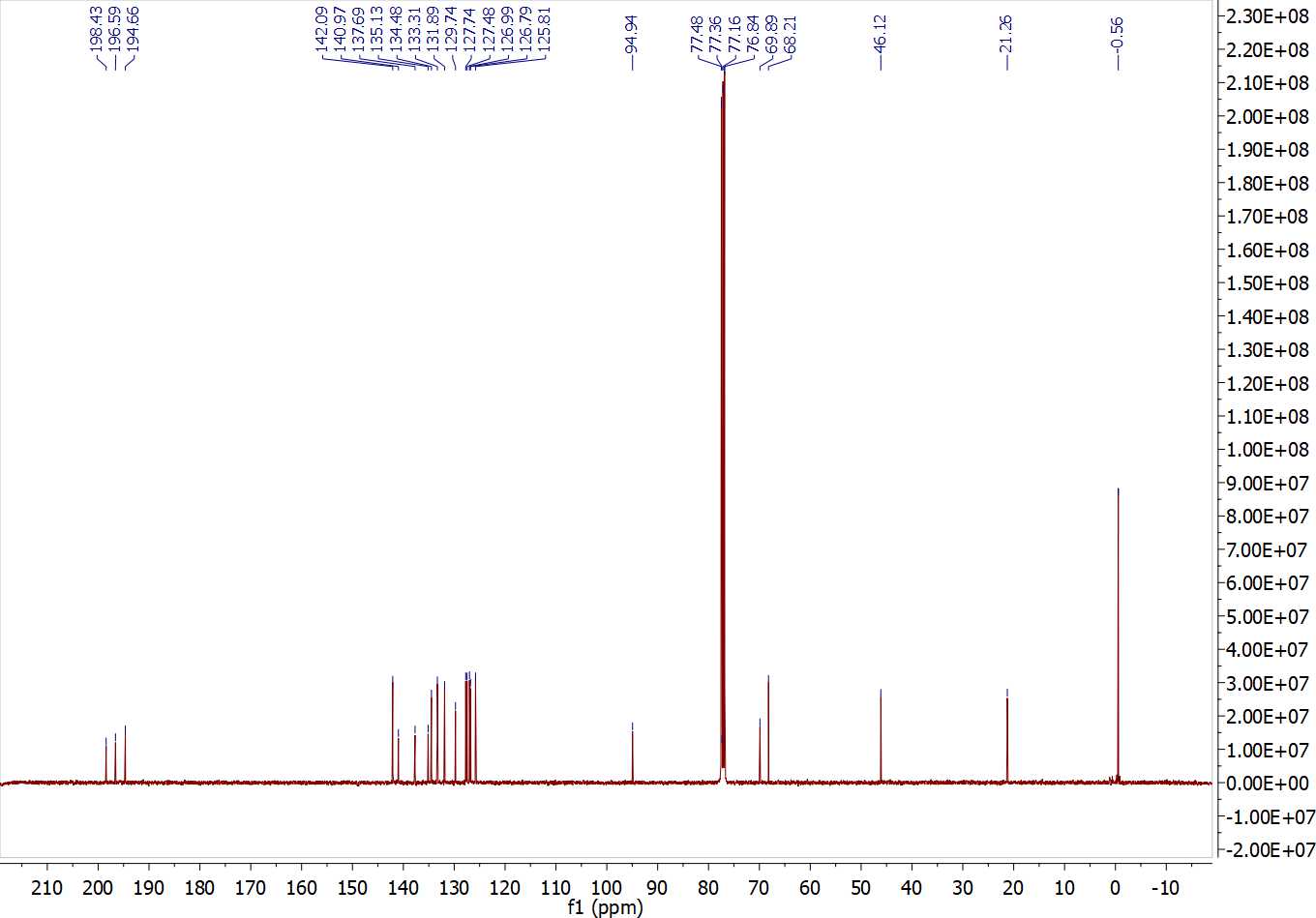


**Figure 34** ^13^C NMR (101 MHz, CDCl_3_) **9ad** Iodine TMS DA F1.


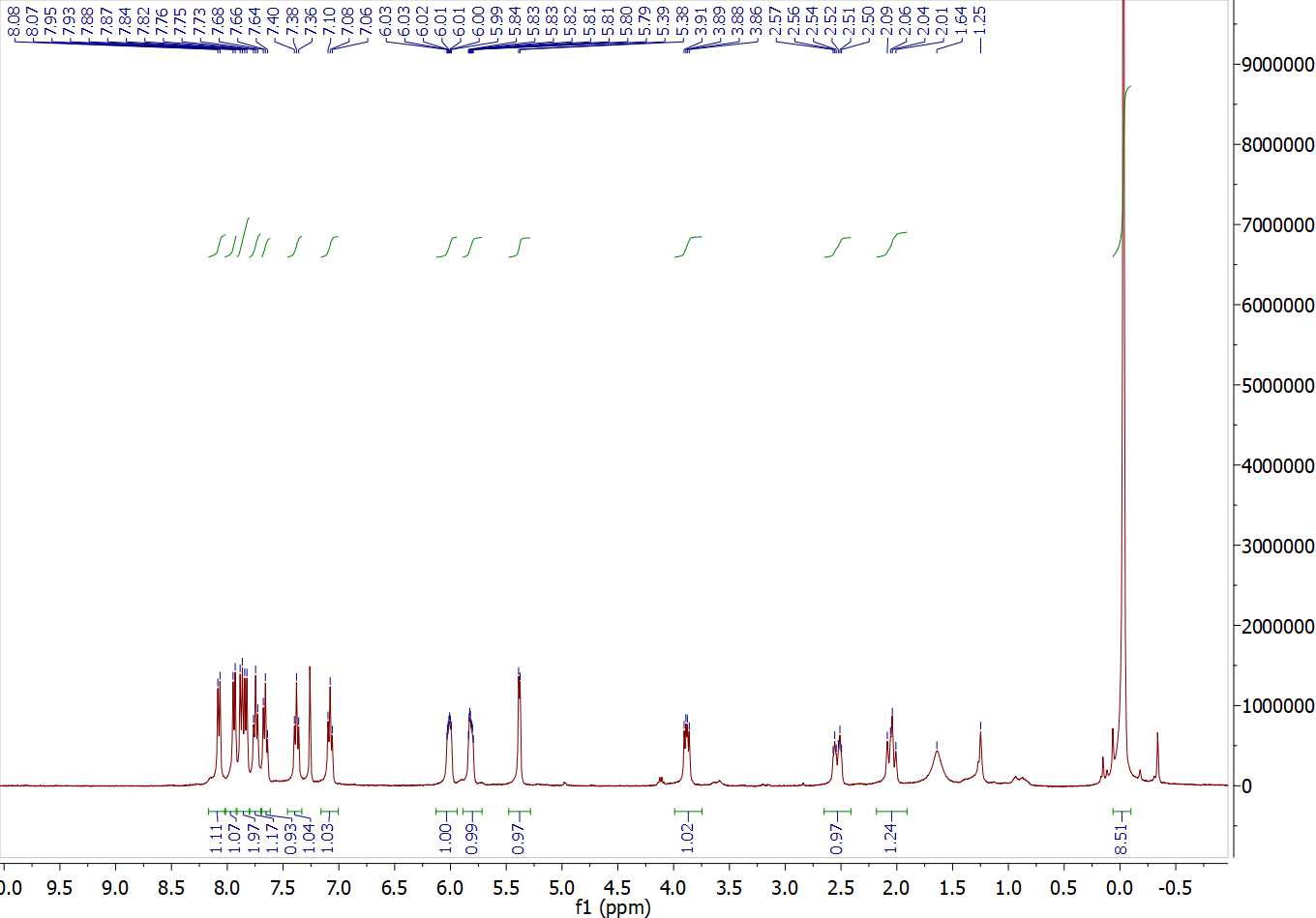


**Figure 35** ^1^H NMR (400 MHz, CDCl_3_) **8ad** Iodine TMS DA F2.


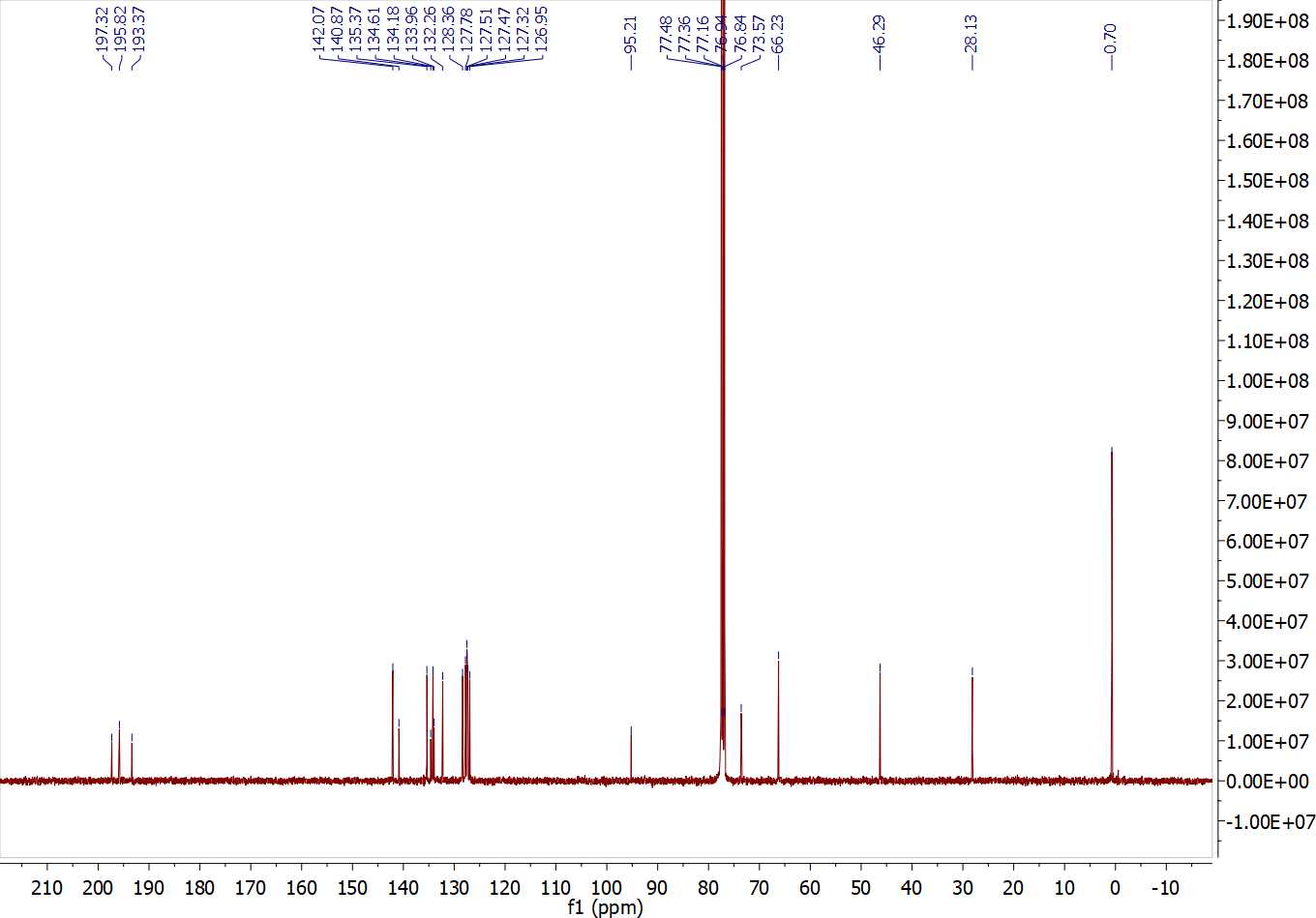


**Figure 36** ^13^C NMR (101 MHz, CDCl_3_) **8ad** Iodine TMS DA F2.


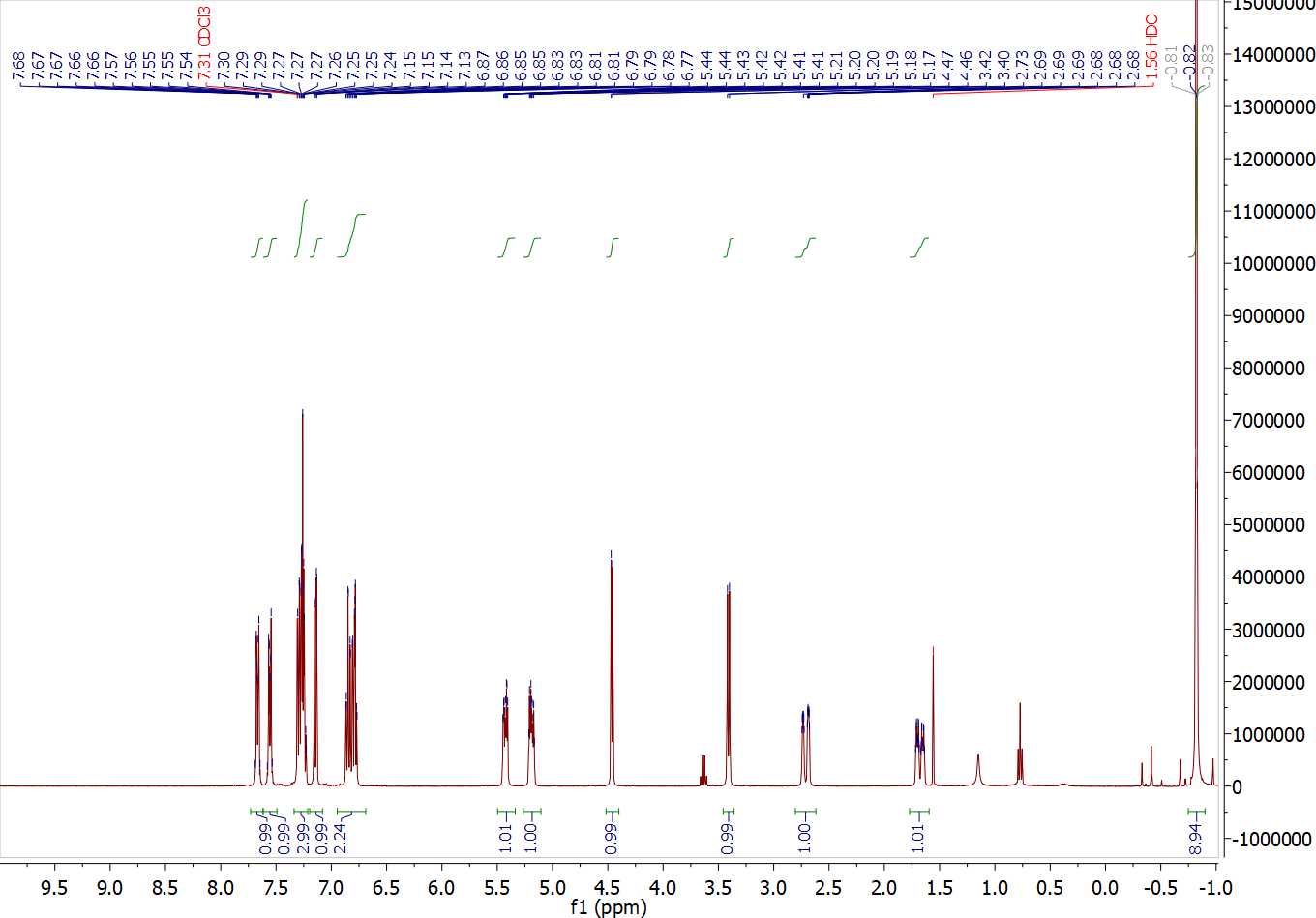


**Figure 37** ^1^H NMR (400 MHz, CDCl_3_) **9bd** Br TMS DA F1.


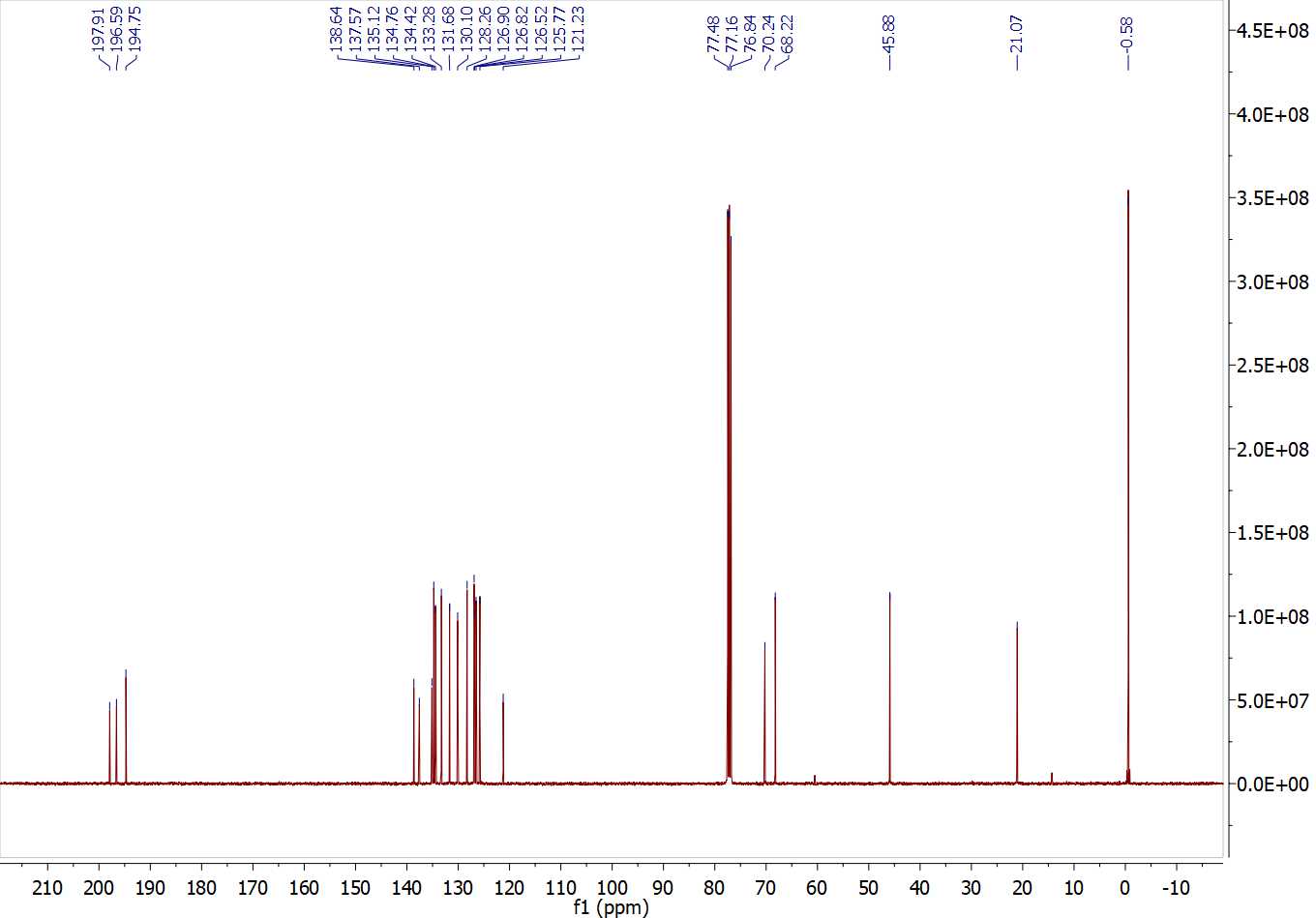


**Figure 38** ^13^C NMR (101 MHz, CDCl_3_) **9bd** Br TMS DA F1.


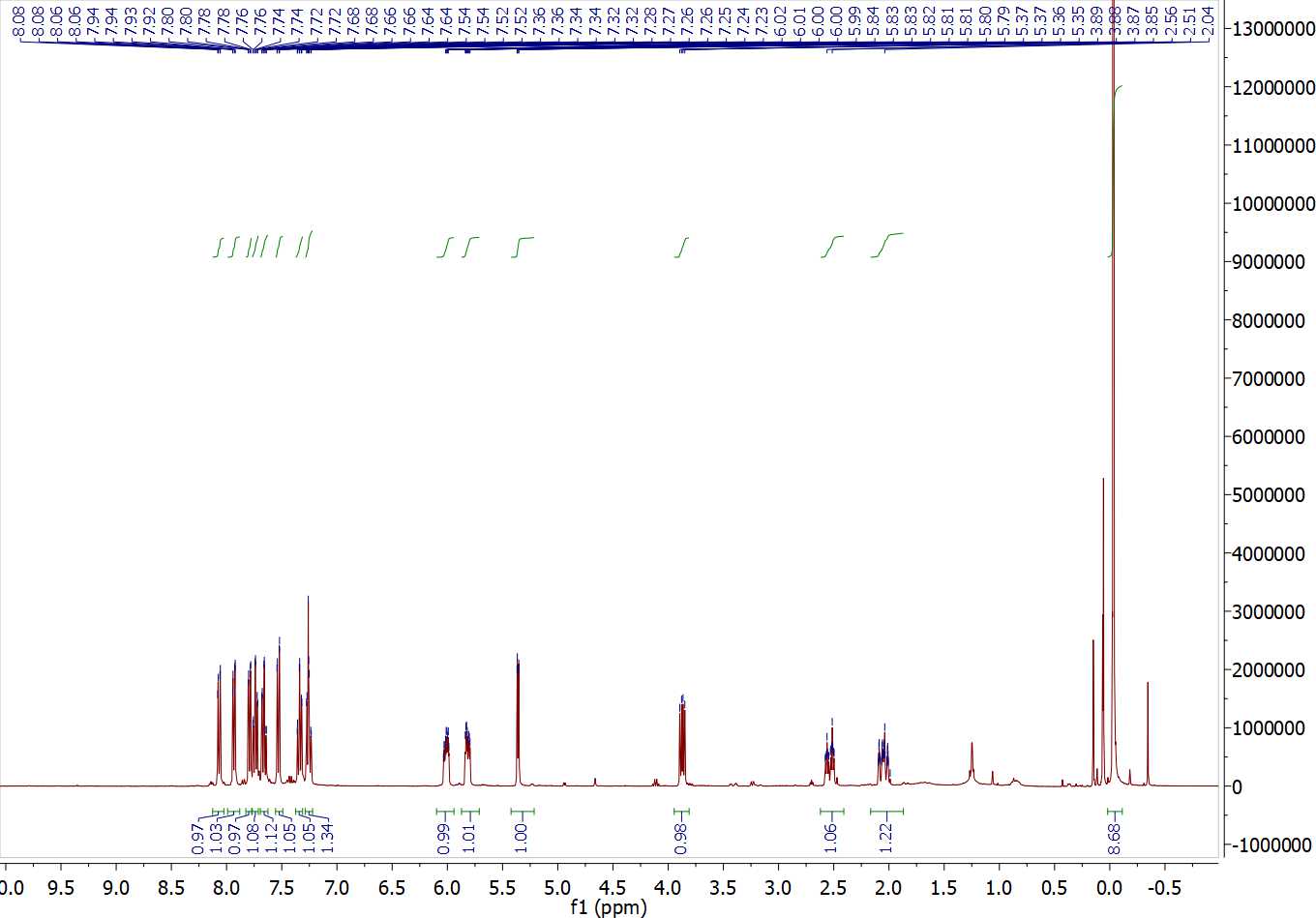


**Figure 39** ^1^H NMR (400 MHz, CDCl_3_) **8bd** Br TMS DA F2.


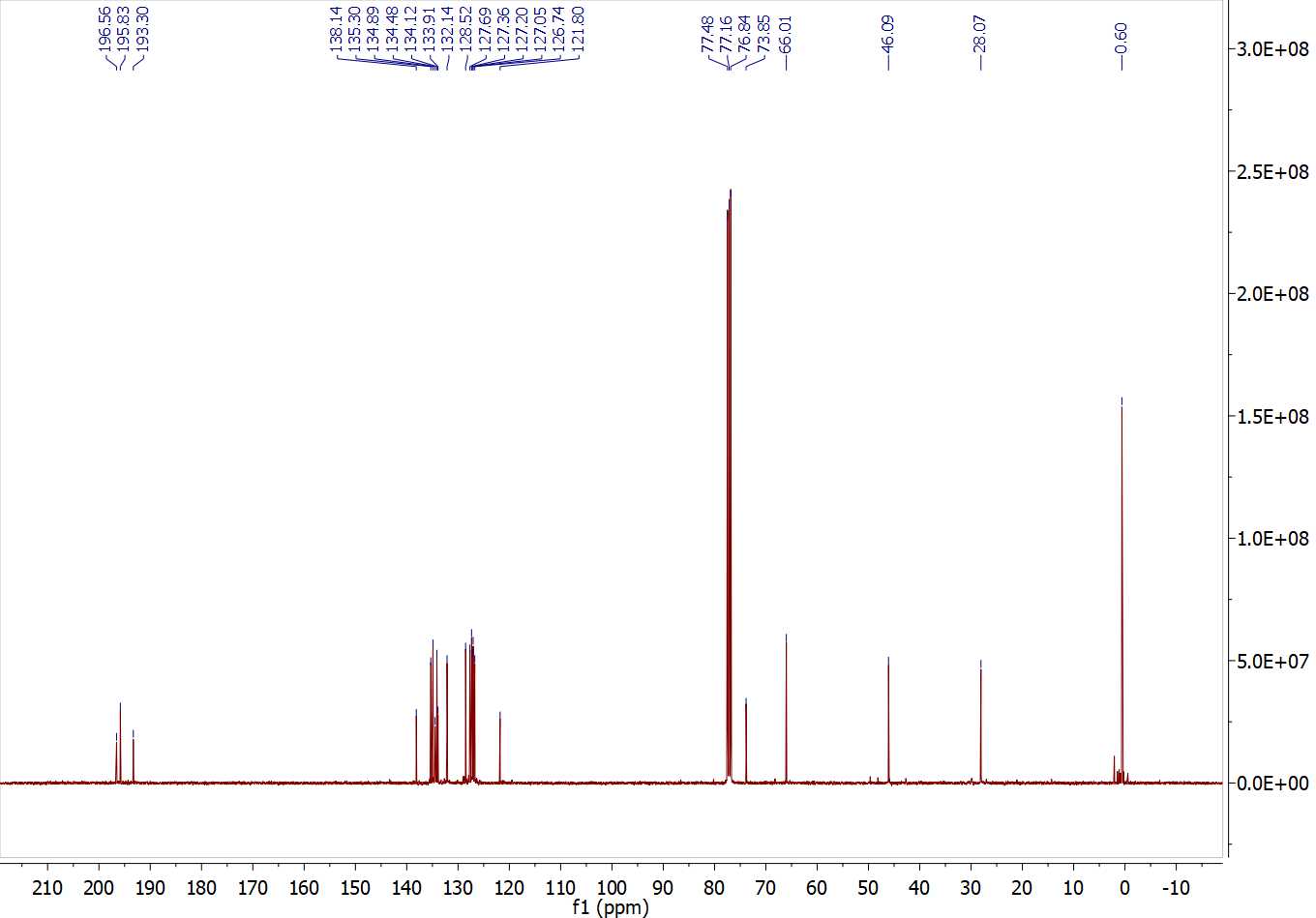


**Figure 40** ^13^C NMR (101 MHz, CDCl_3_) **8bd** Br TMS DA F2.


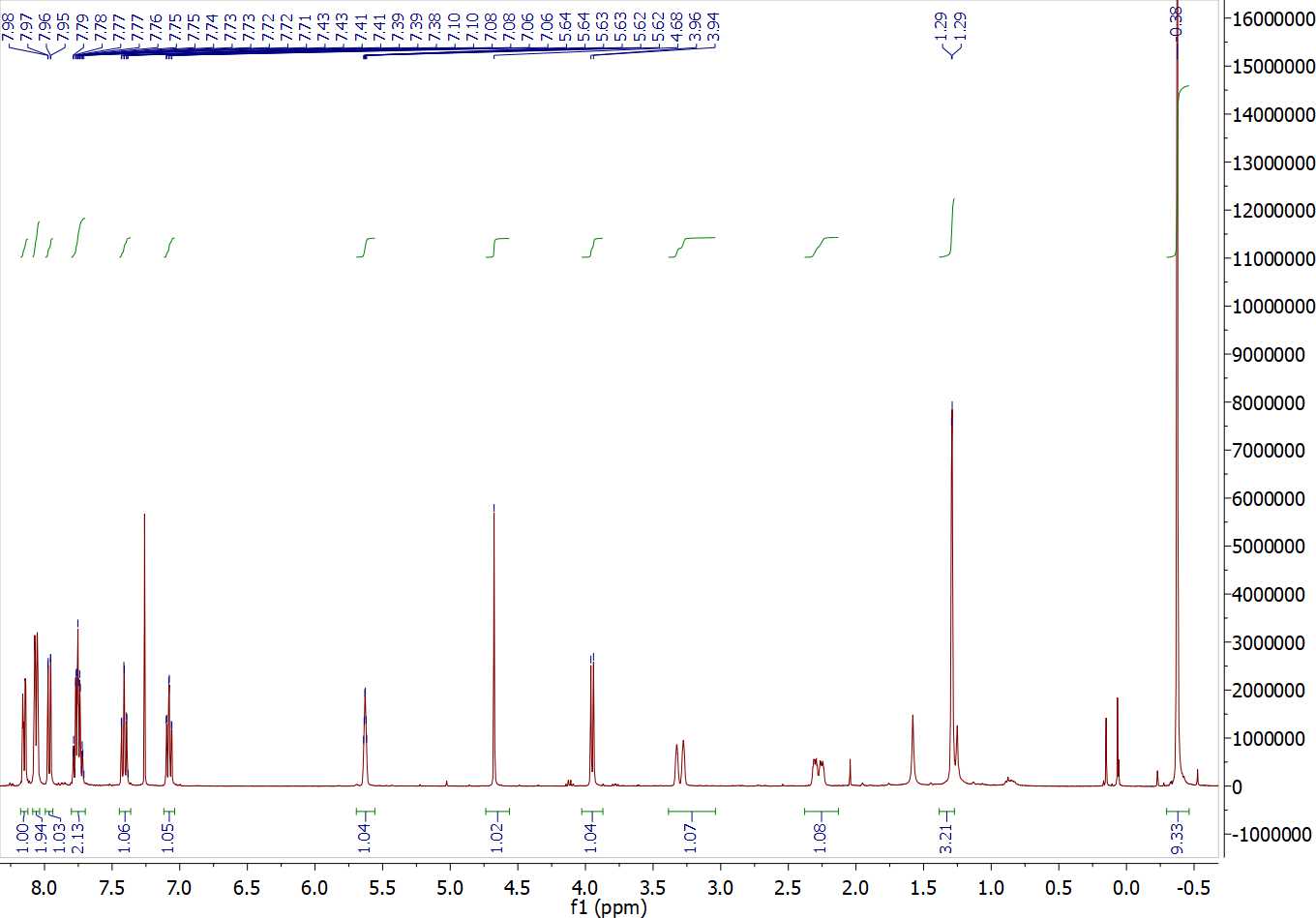


**Figure 41** ^1^H NMR (400 MHz, CDCl_3_) **9ae** Iodine Me TMS DA F1.


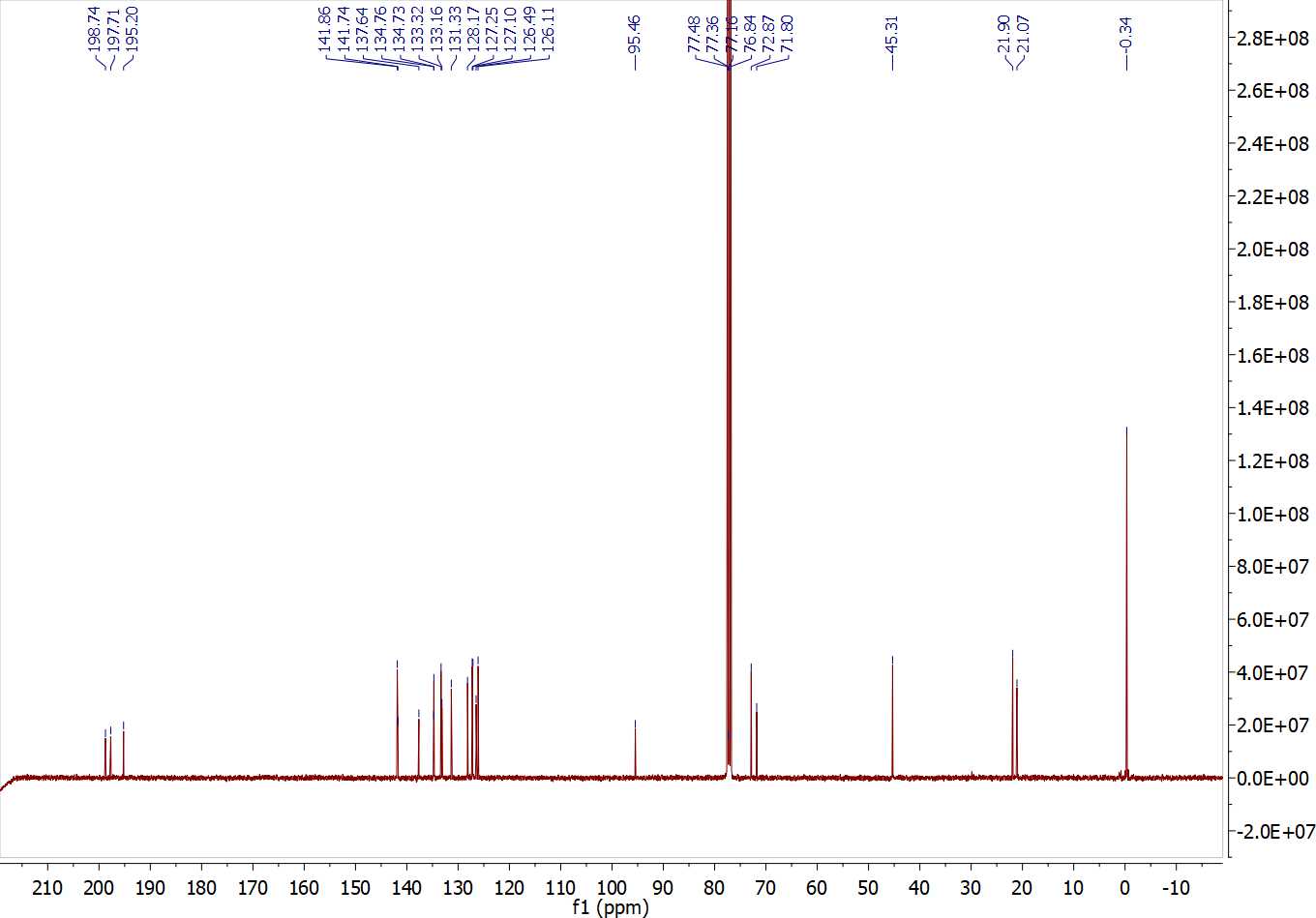


**Figure 42** ^13^C NMR (101 MHz, CDCl_3_) **9ae** Iodine Me TMS DA F1.


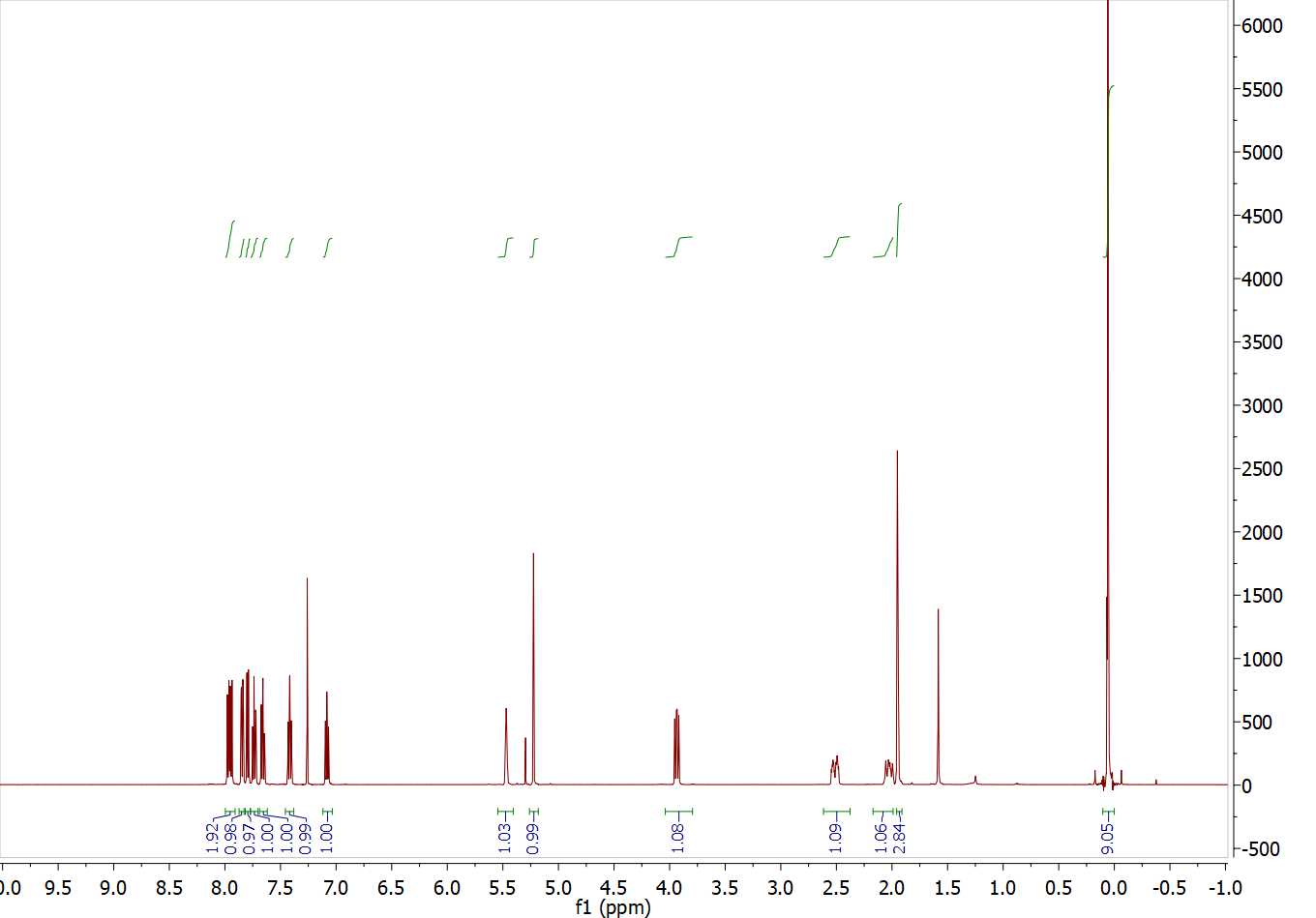


**Figure 43** ^1^H NMR (400 MHz, CDCl_3_) **8ae** Iodine Me TMS DA F2.


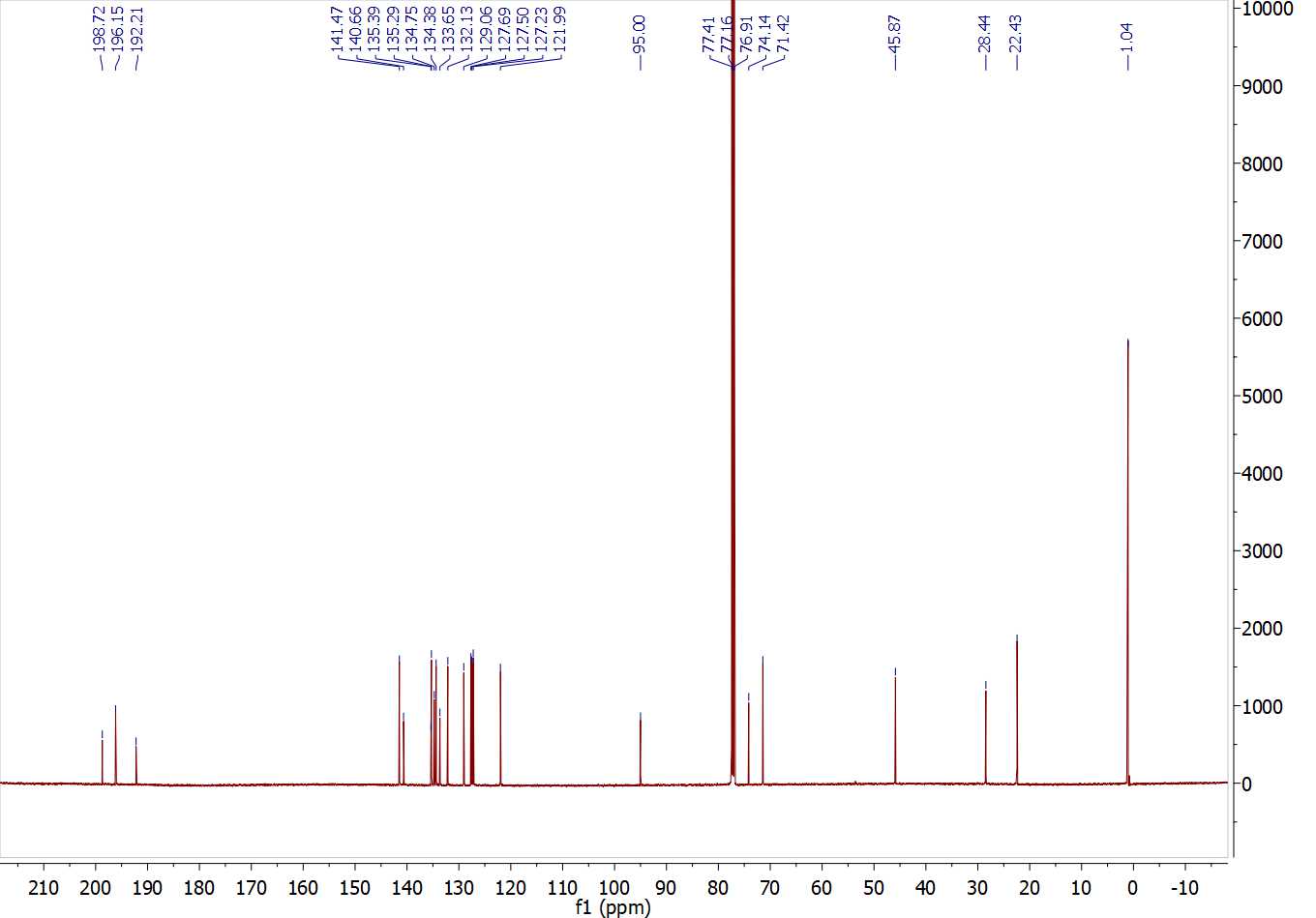


**Figure 44** ^13^C NMR (101 MHz, CDCl_3_) **8ae** Iodine Me TMS DA F2.


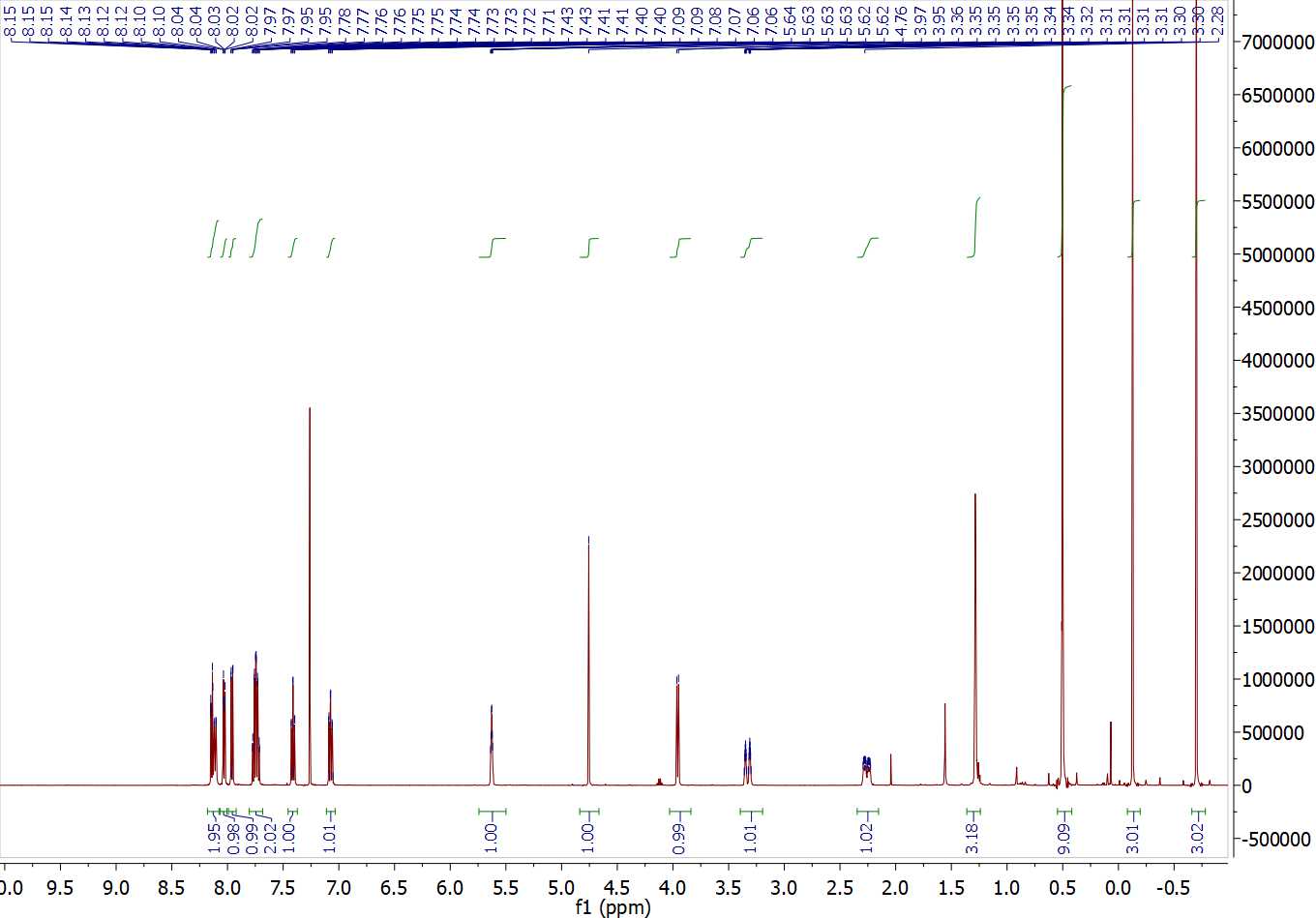


**Figure 45** ^1^H NMR (500 MHz, CDCl_3_) **9af** Iodine Me TBDMS DA F1.


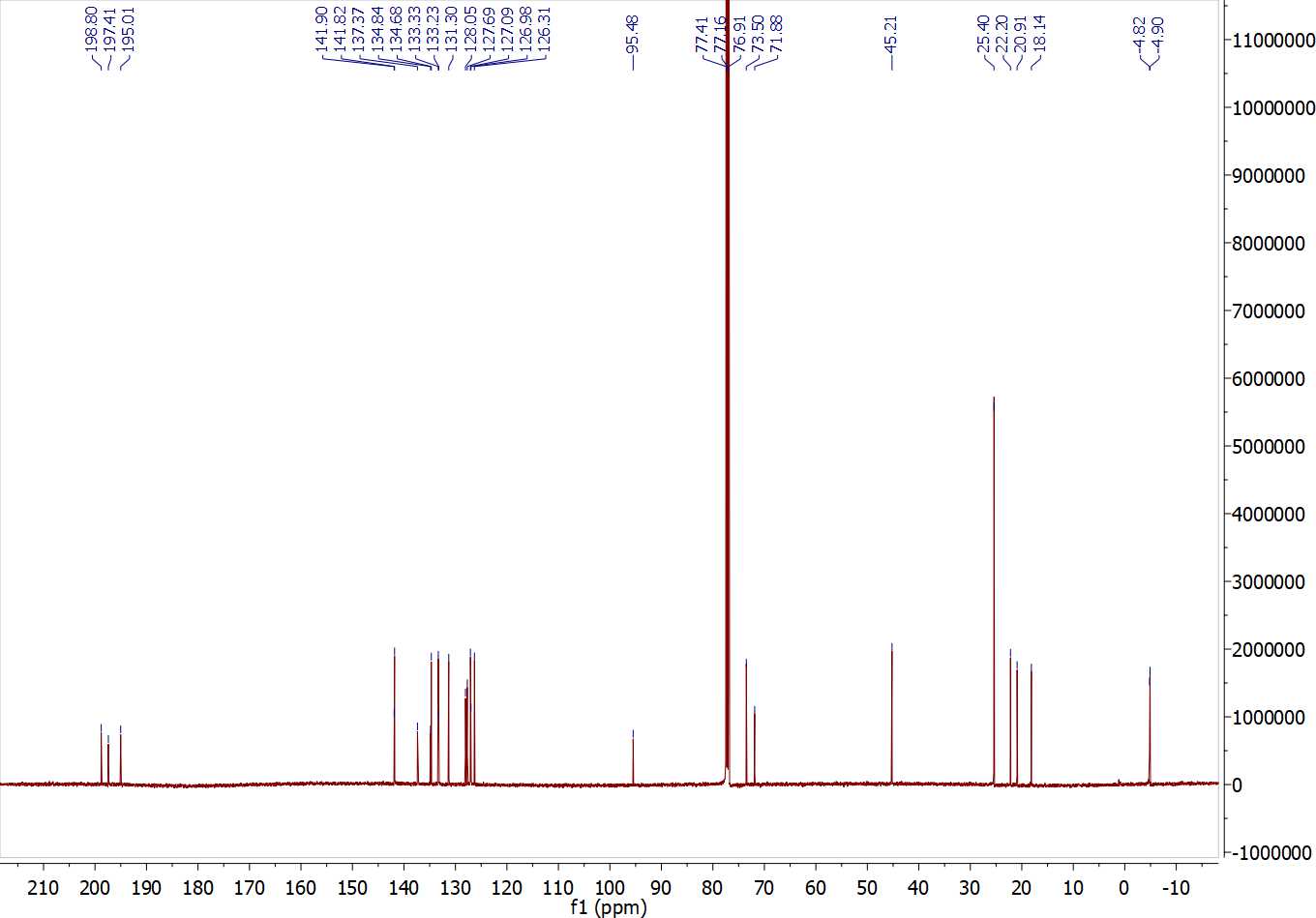


**Figure 46** ^13^C NMR (126 MHz, CDCl_3_) **9af** Iodine Me TBDMS DA F1.


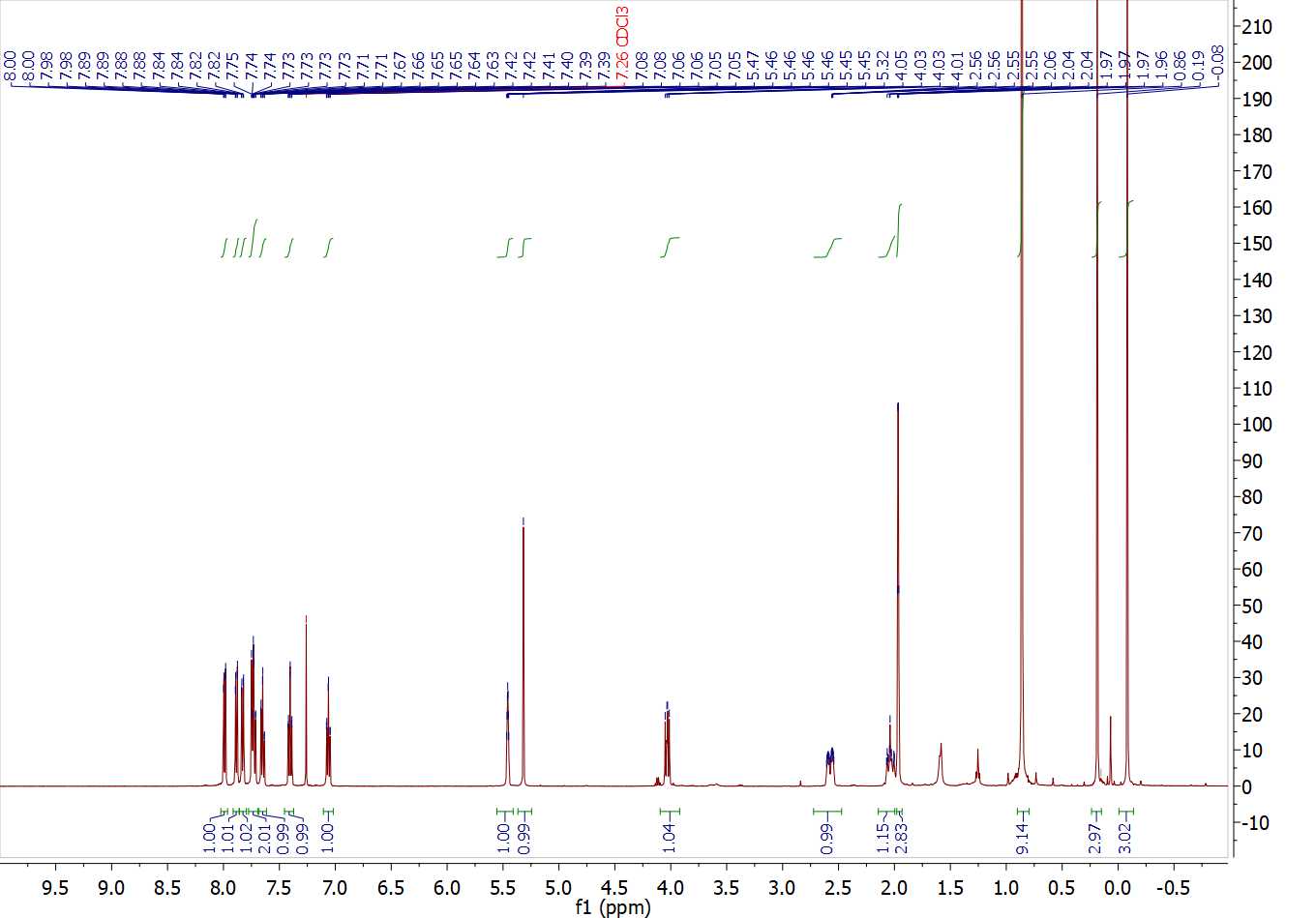


**Figure 47** ^1^H NMR (500 MHz, CDCl_3_) **8af** Iodine Me TBDMS DA F2.


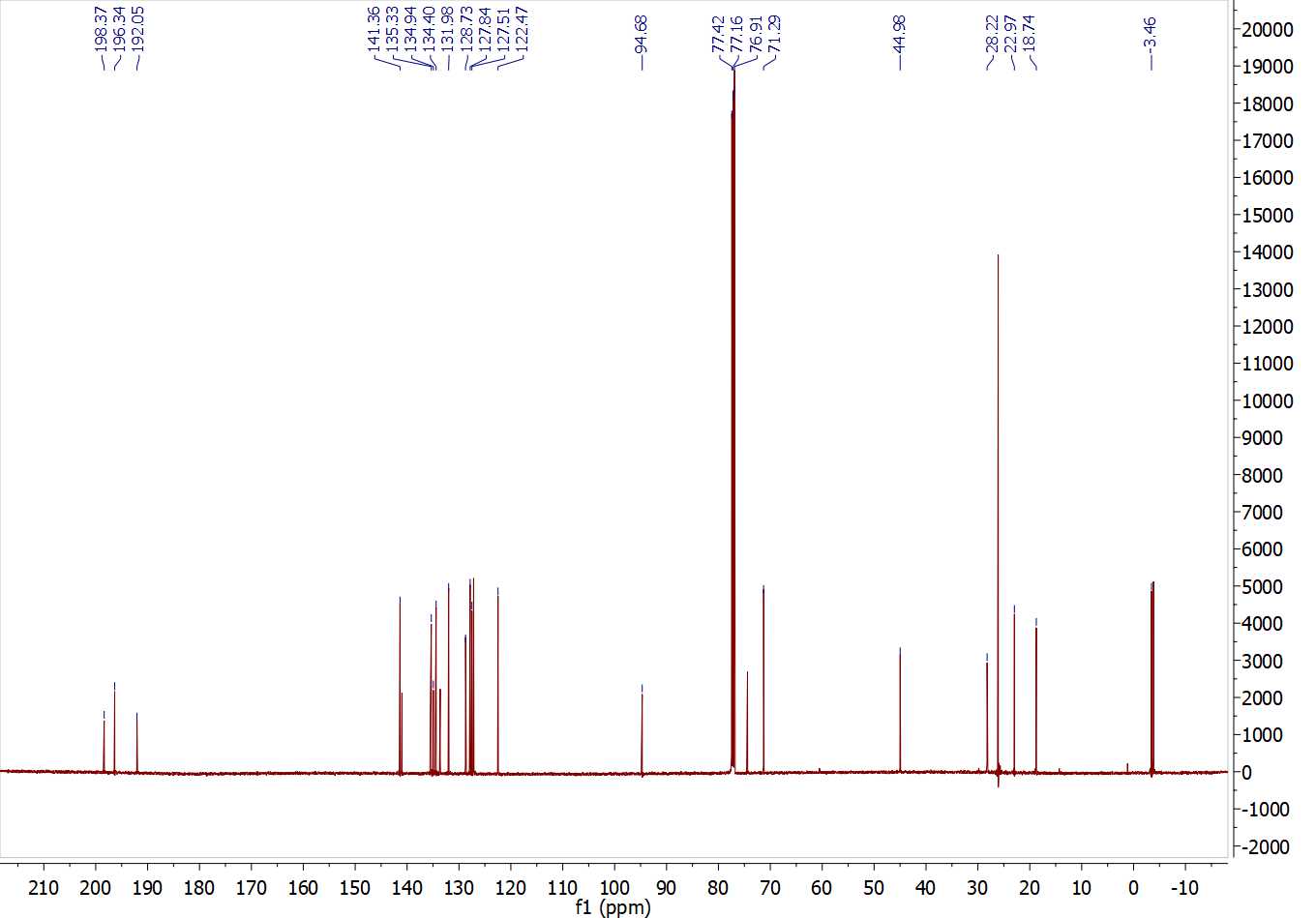


**Figure 48** ^13^C NMR (126 MHz, CDCl_3_) **8af** Iodine Me TBDMS DA F2.


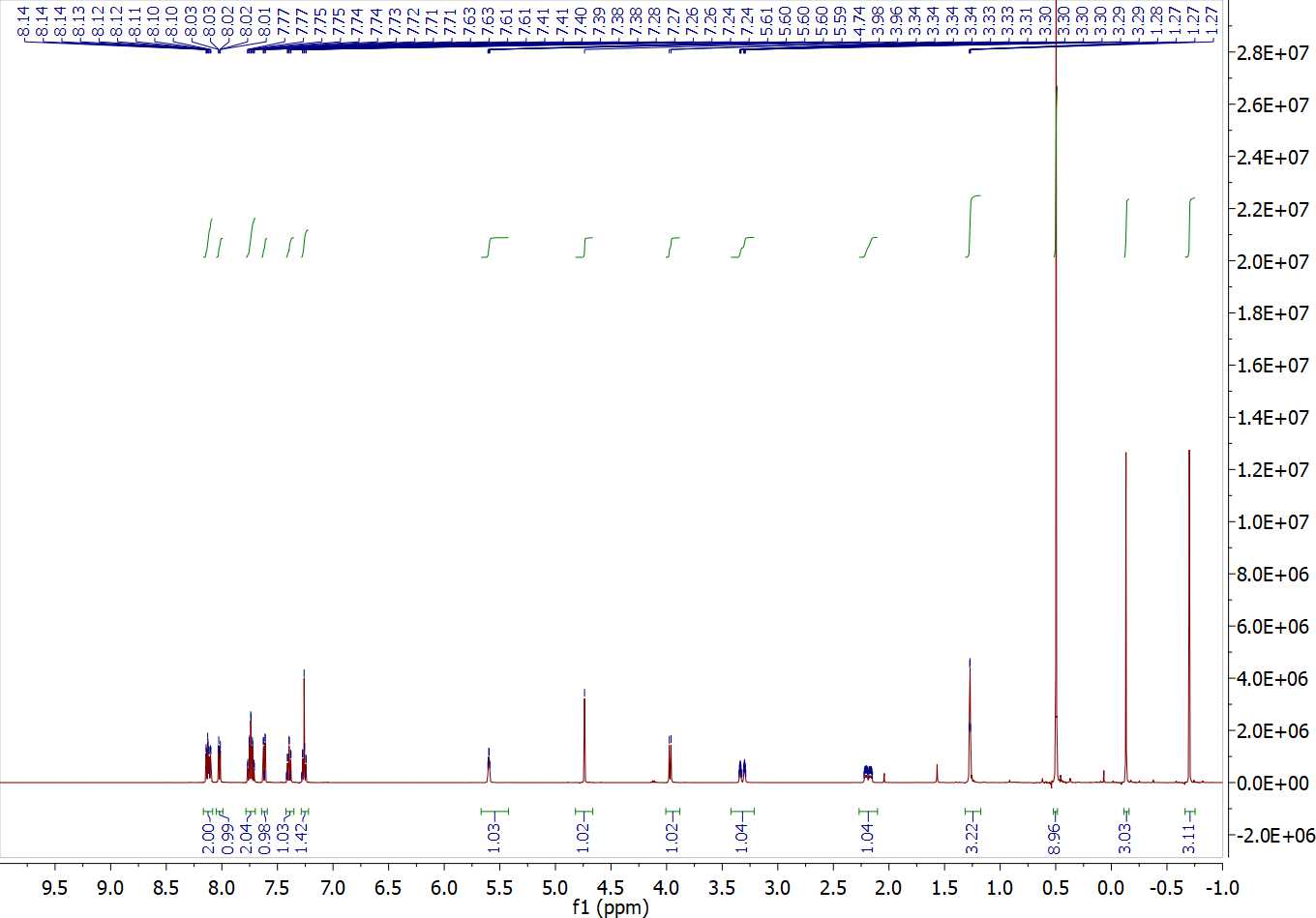


**Figure 49** ^1^H NMR (500 MHz, CDCl_3_) **8/9bf** Br Me TBDMS DA F1.


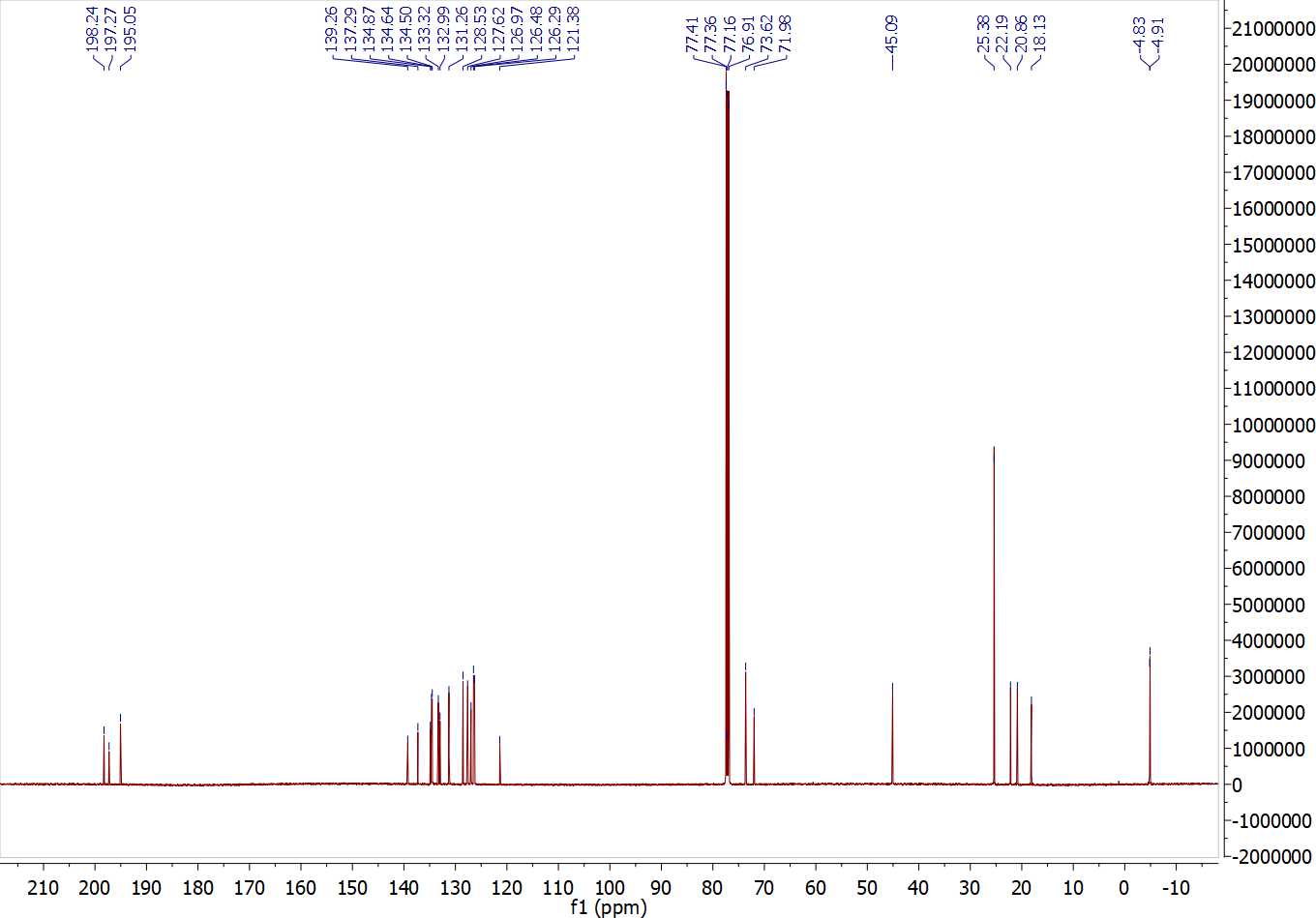


**Figure 50** ^13^C NMR (126 MHz, CDCl_3_) **8/9bf** Br Me TBDMS DA F1.


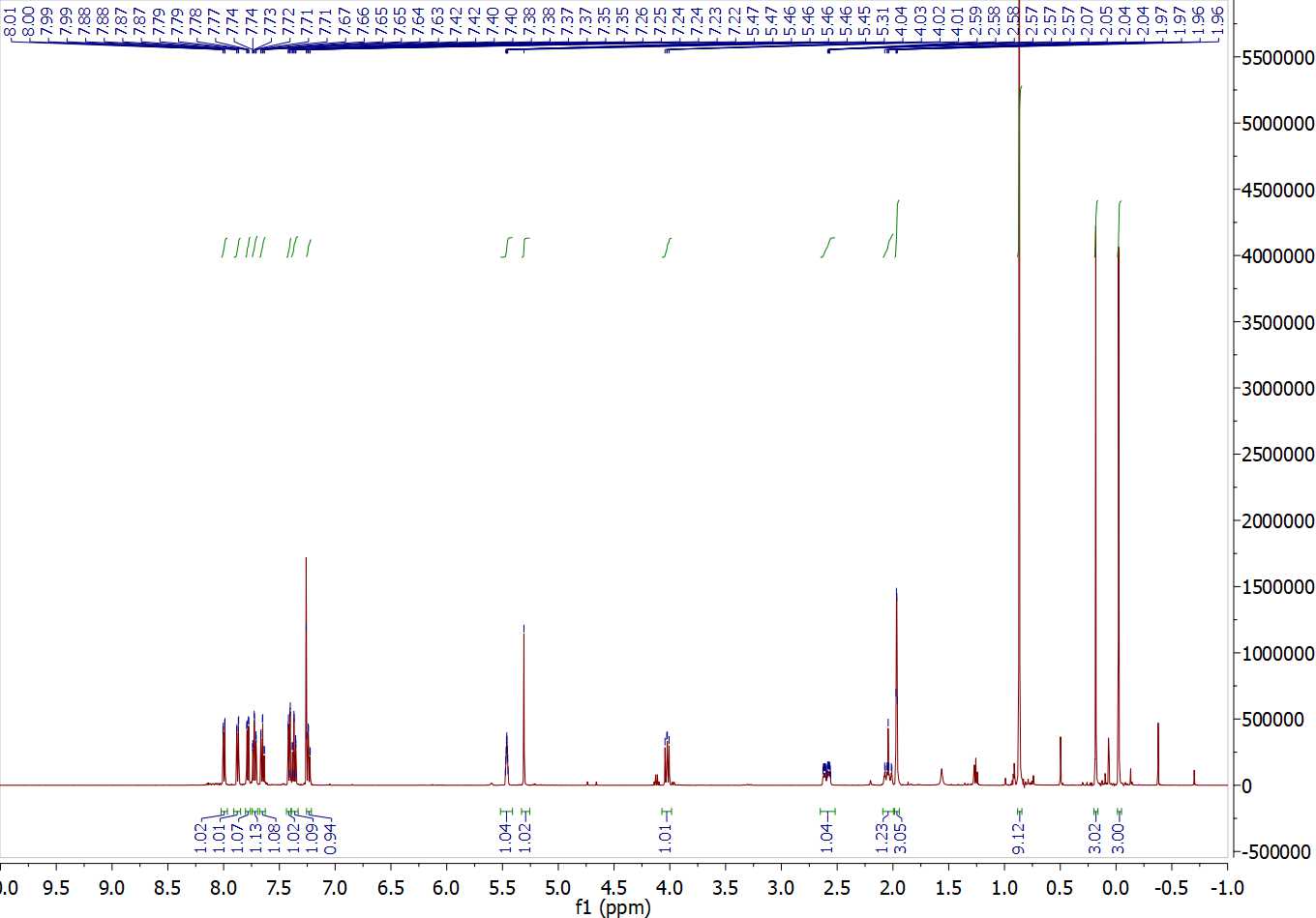


**Figure 51** ^1^H NMR (500 MHz, CDCl_3_) **8bf** Br Me TBDMS DA F2.


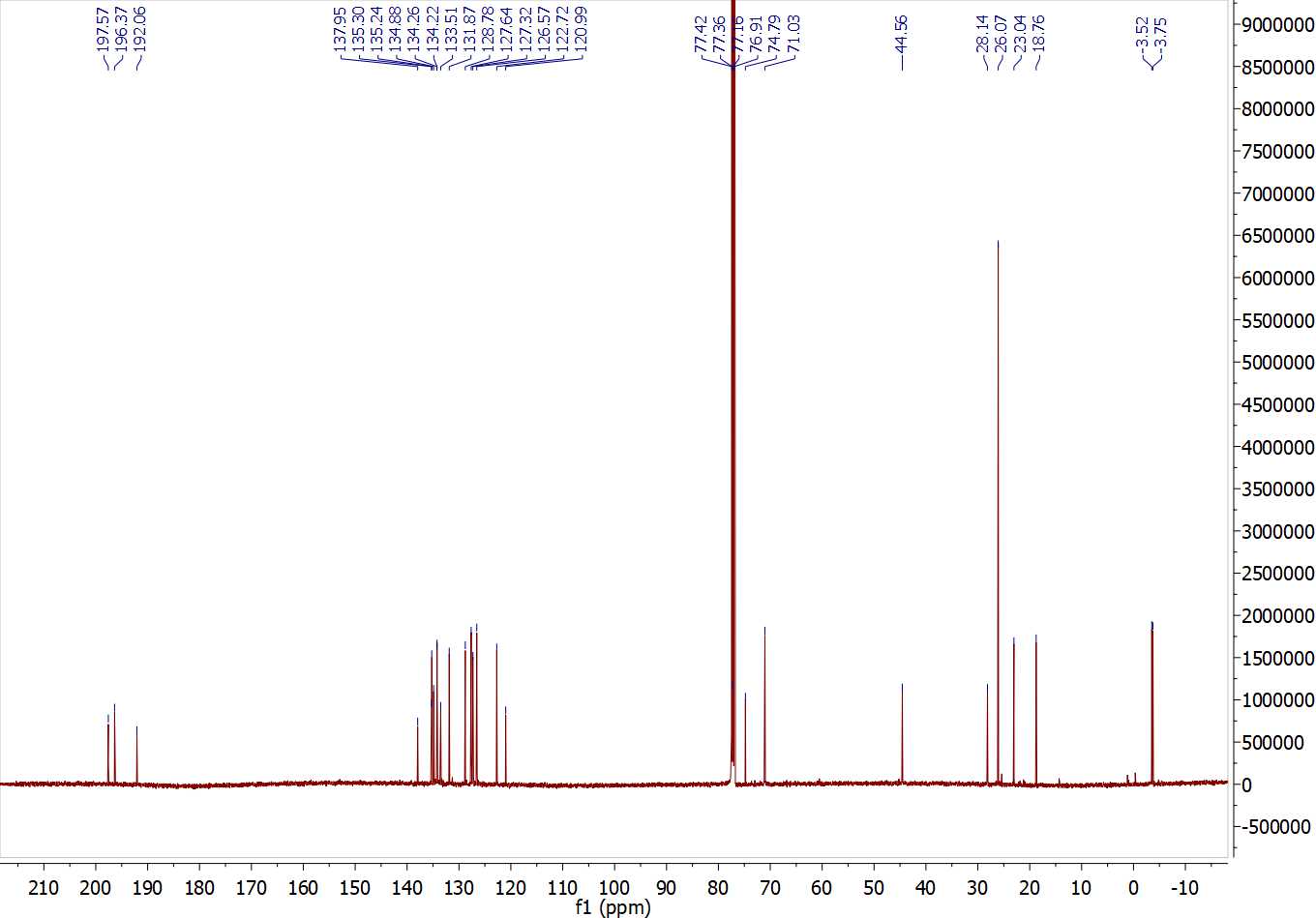


**Figure 52** ^13^C NMR (126 MHz, CDCl_3_) **8bf** Br Me TBDMS DA F2.


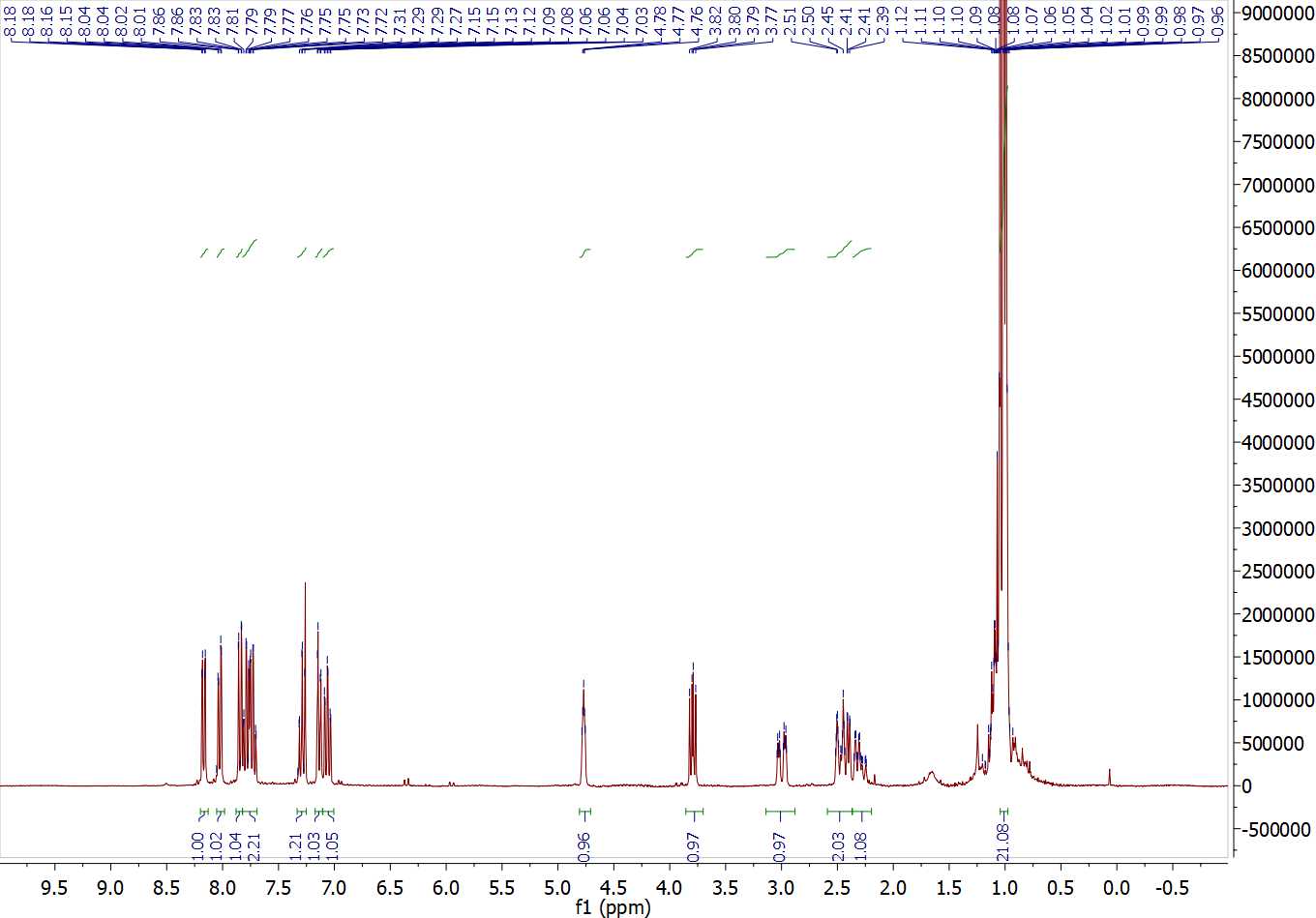


**Figure 53** ^1^H NMR (400 MHz, CDCl_3_) **8ag** Iodine TIPS DA.


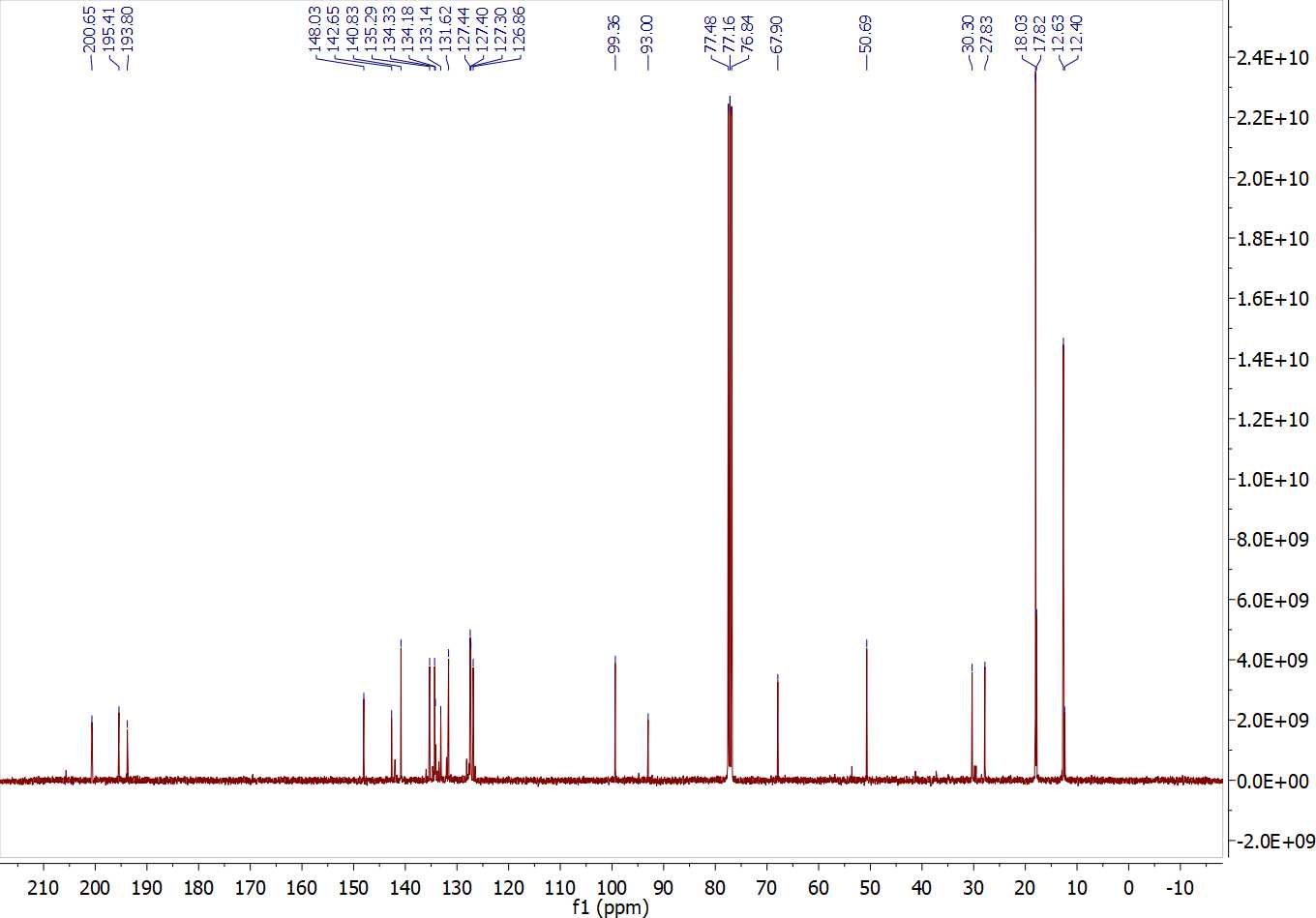


**Figure 54** ^13^C NMR (101 MHz, CDCl_3_) **8ag** Iodine TIPS DA.


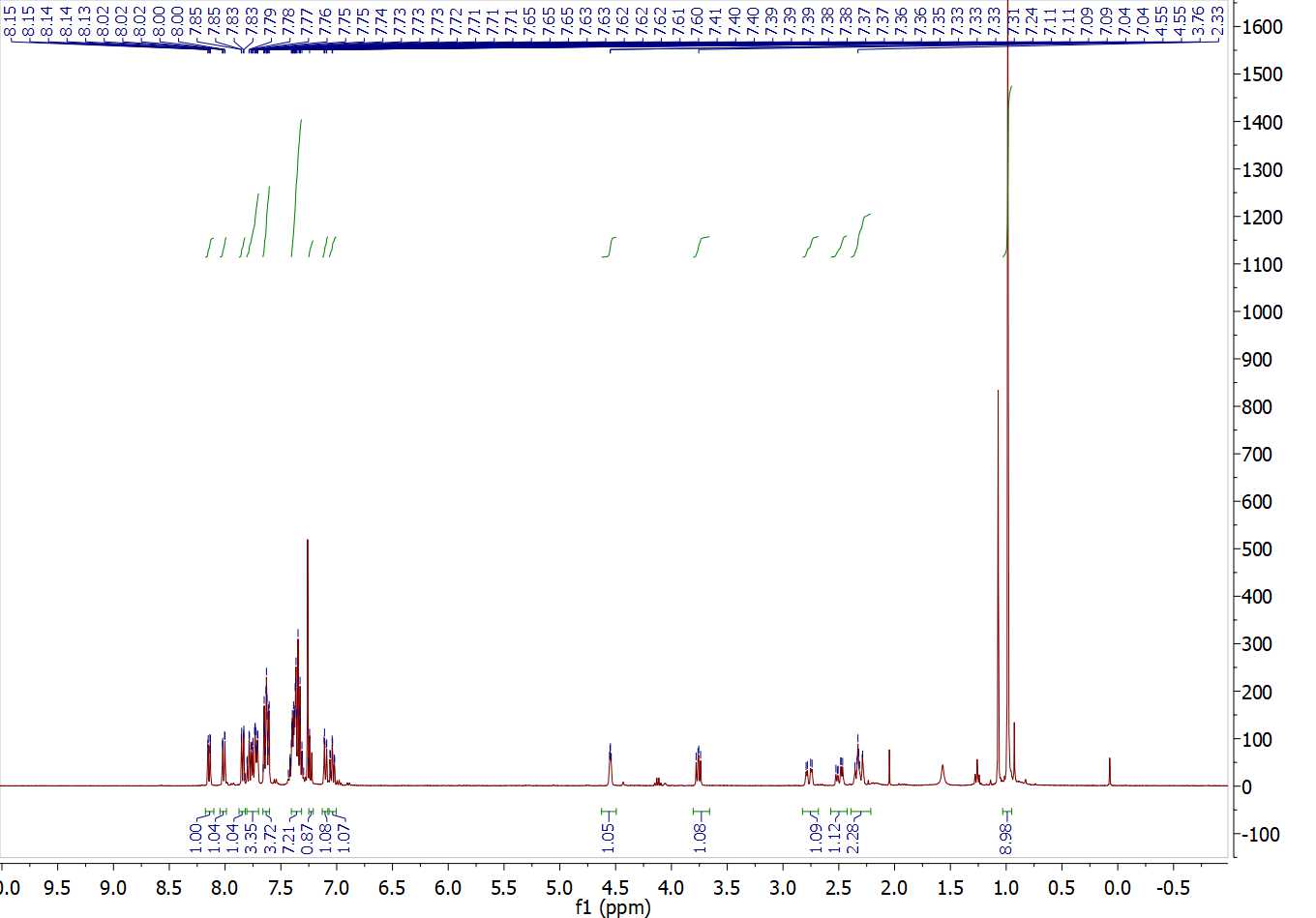


**Figure 55** ^1^H NMR (400 MHz, CDCl_3_) **8ah** Iodine TBDPS DA.


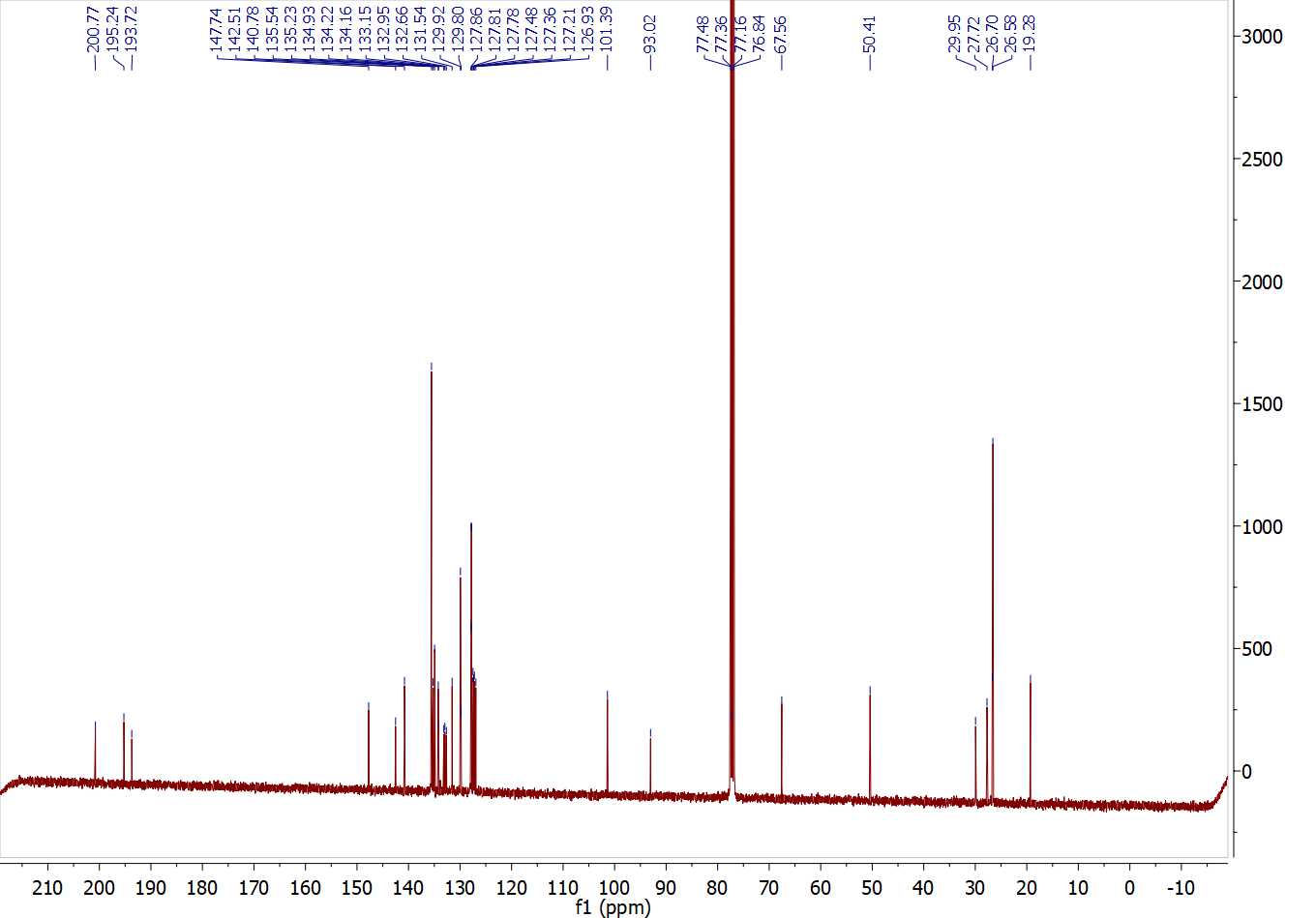


**Figure 56** ^13^C NMR (101 MHz, CDCl_3_) **8ah** Iodine TBDPS DA.


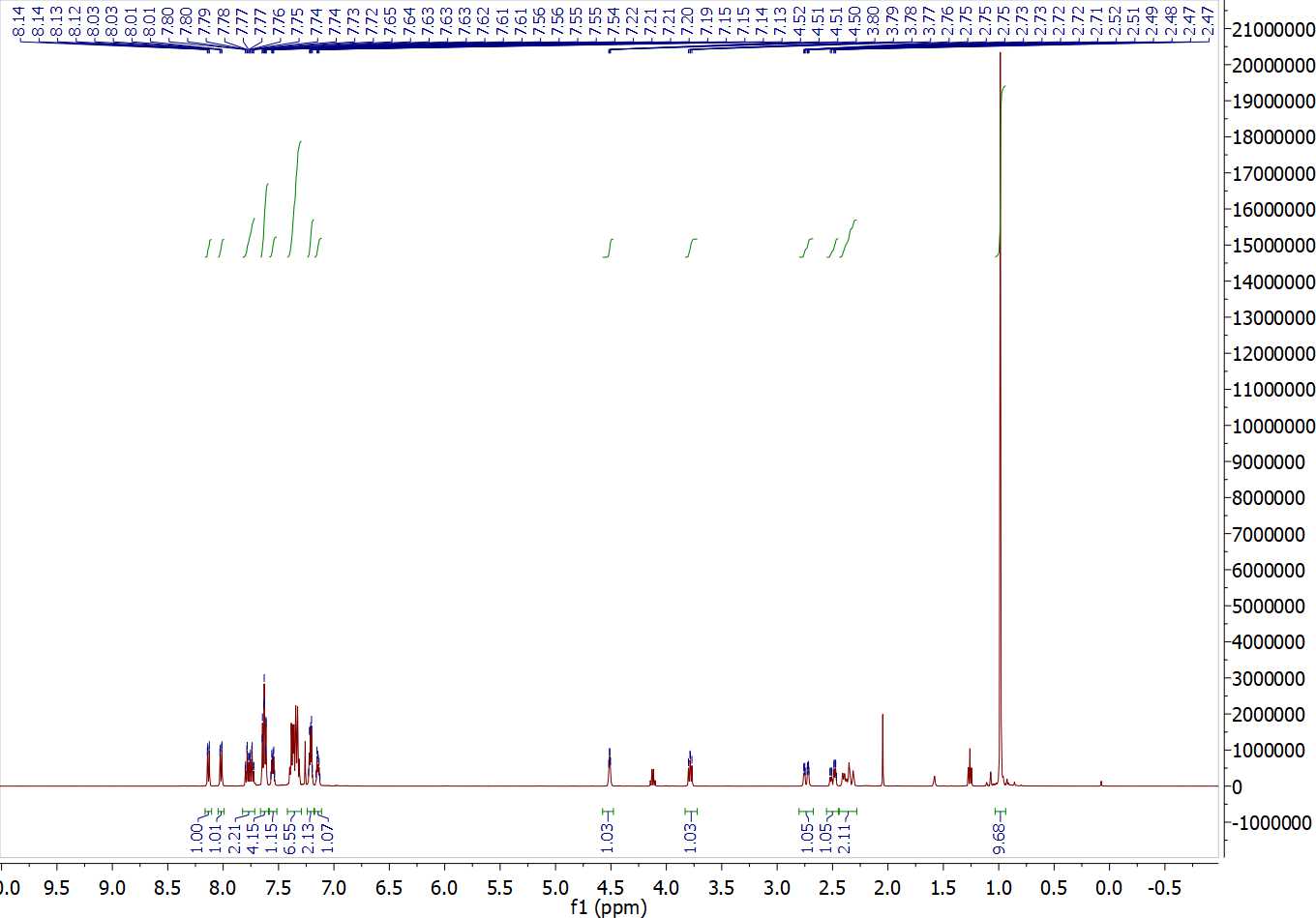


**Figure 57** ^1^H NMR (500 MHz, CDCl_3_) **8bh** Br TBDPS DA.


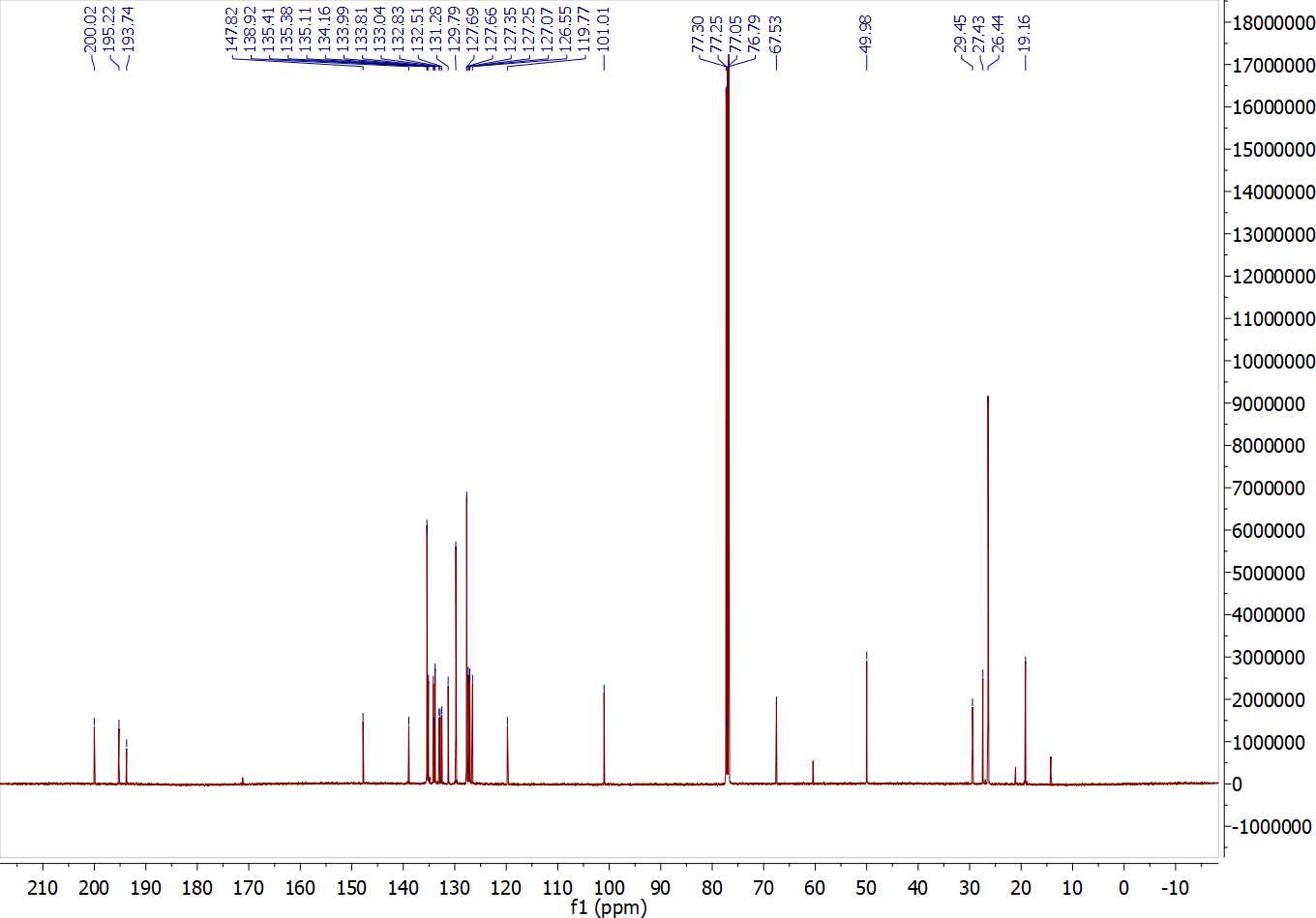


**Figure 58** ^13^C NMR (126 MHz, CDCl_3_) **8bh** Br TBDPS DA.


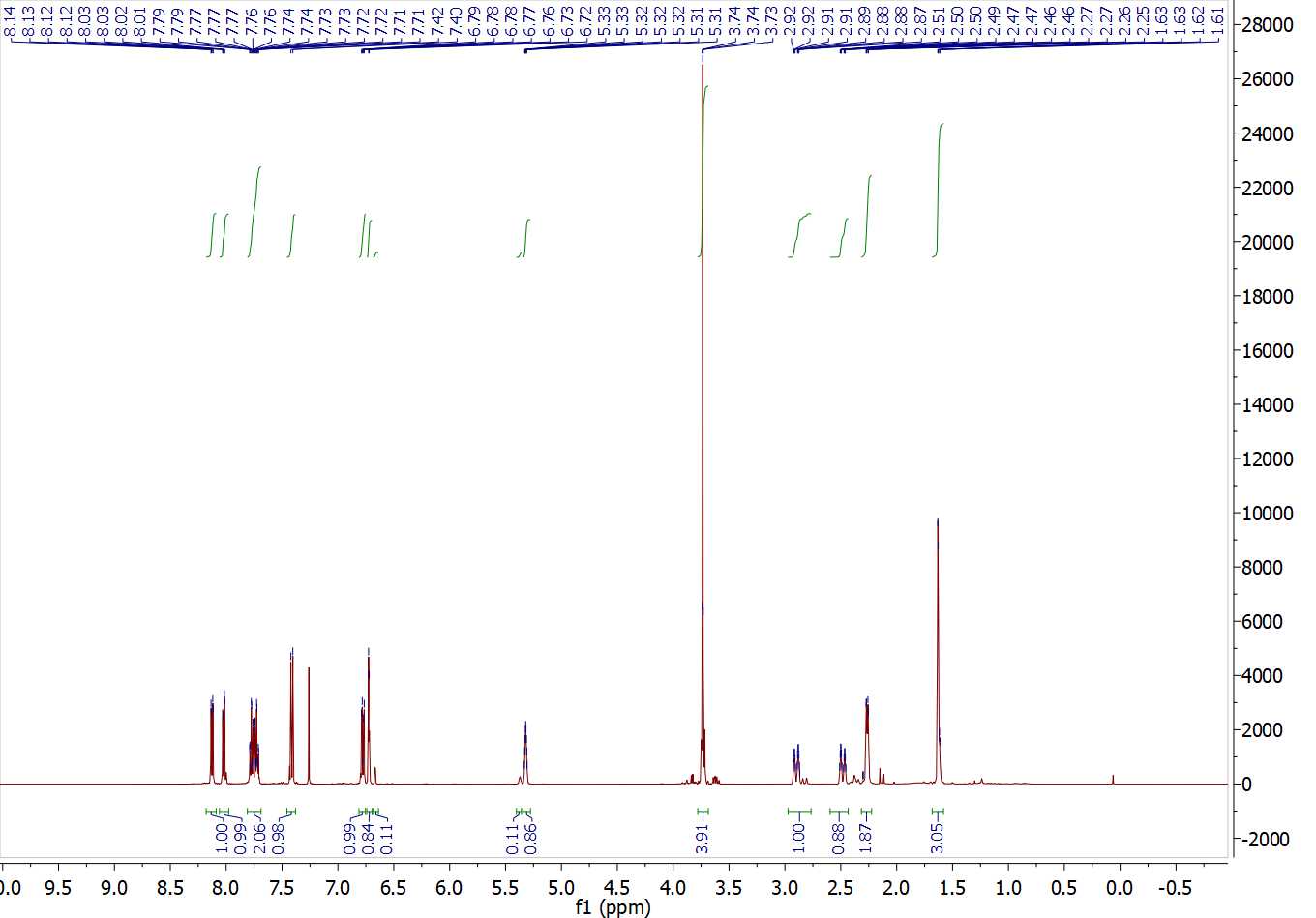


**Figure 59** ^1^H NMR (500 MHz, CDCl_3_) **8cb** Br MeO Isoprene DA.


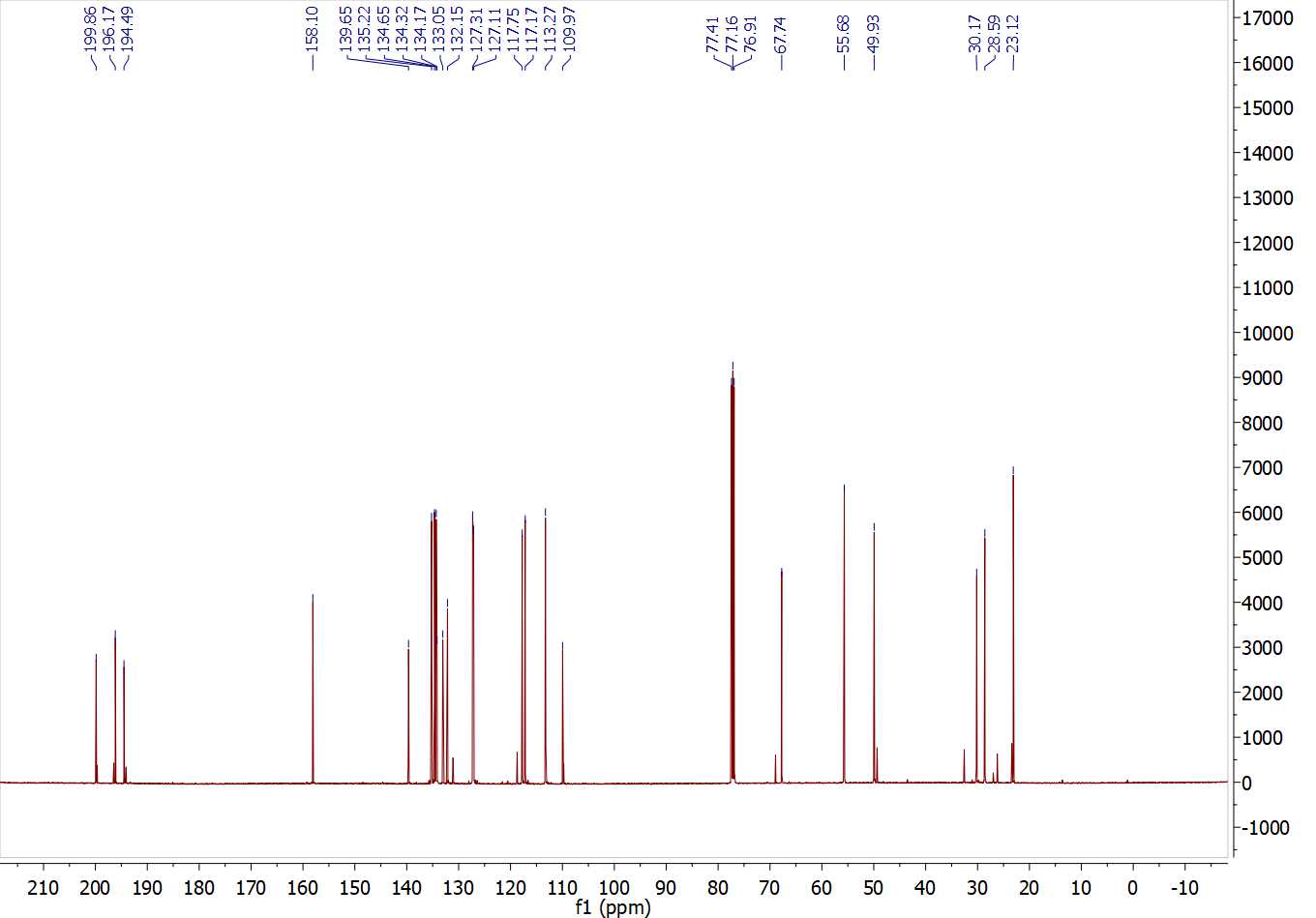


**Figure 60** ^13^C NMR (126 MHz, CDCl_3_) **8cb** Br MeO Isoprene DA.


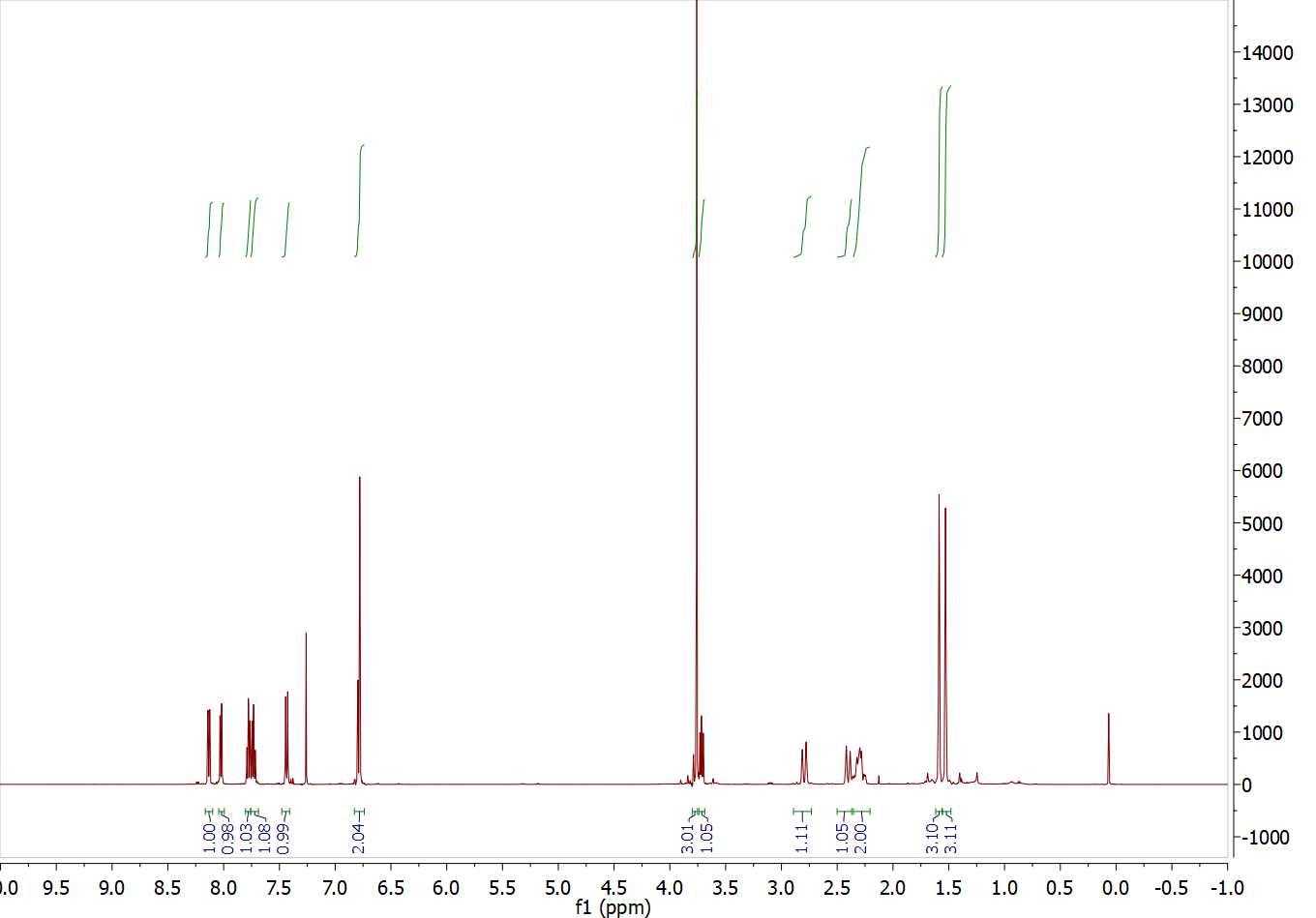


**Figure 61** ^1^H NMR (500 MHz, CDCl_3_) **8cc** Br MeO Dimethyl DA.


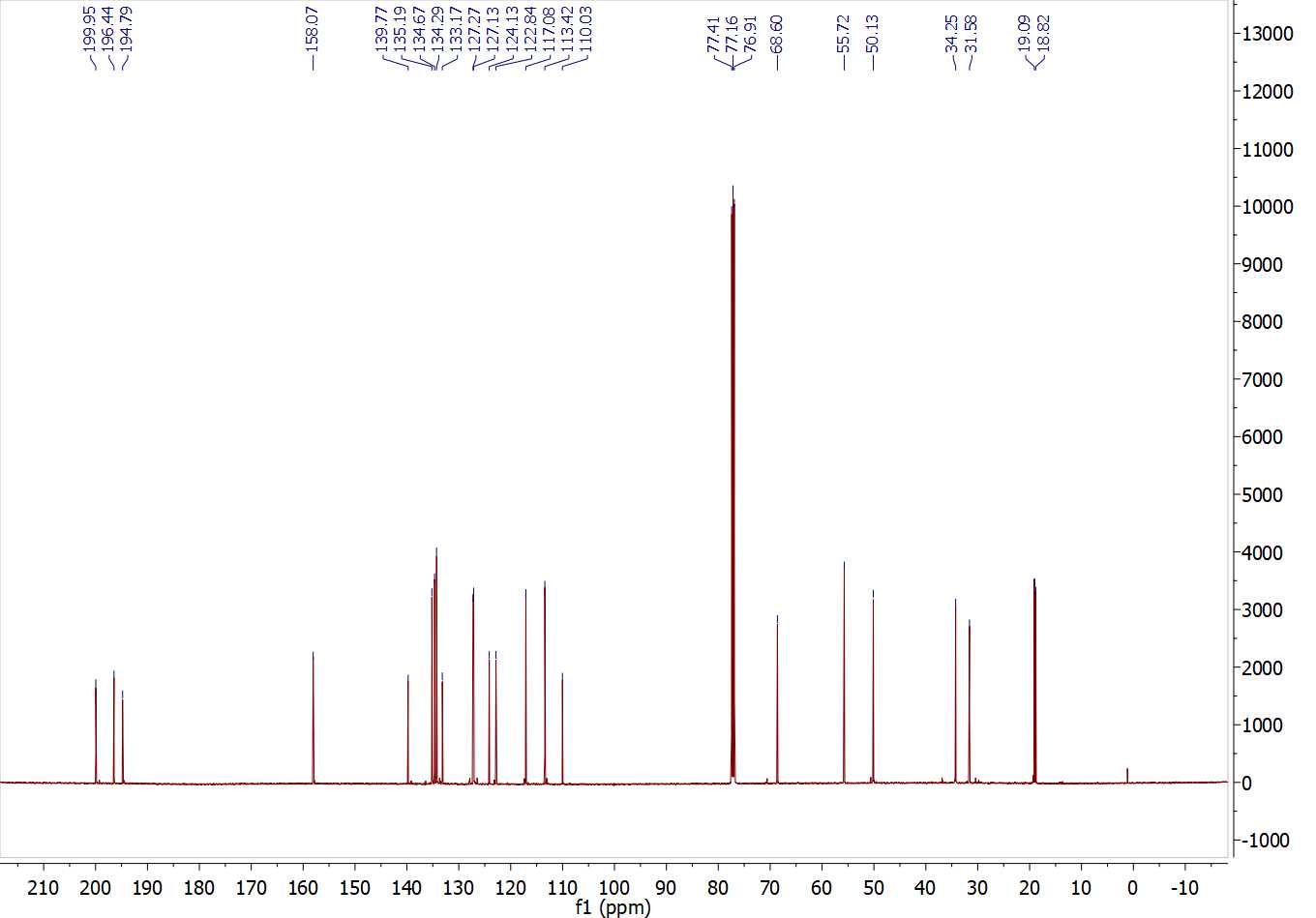


**Figure 62** ^13^C NMR (126 MHz, CDCl_3_) **8cc** Br MeO Dimethyl DA.


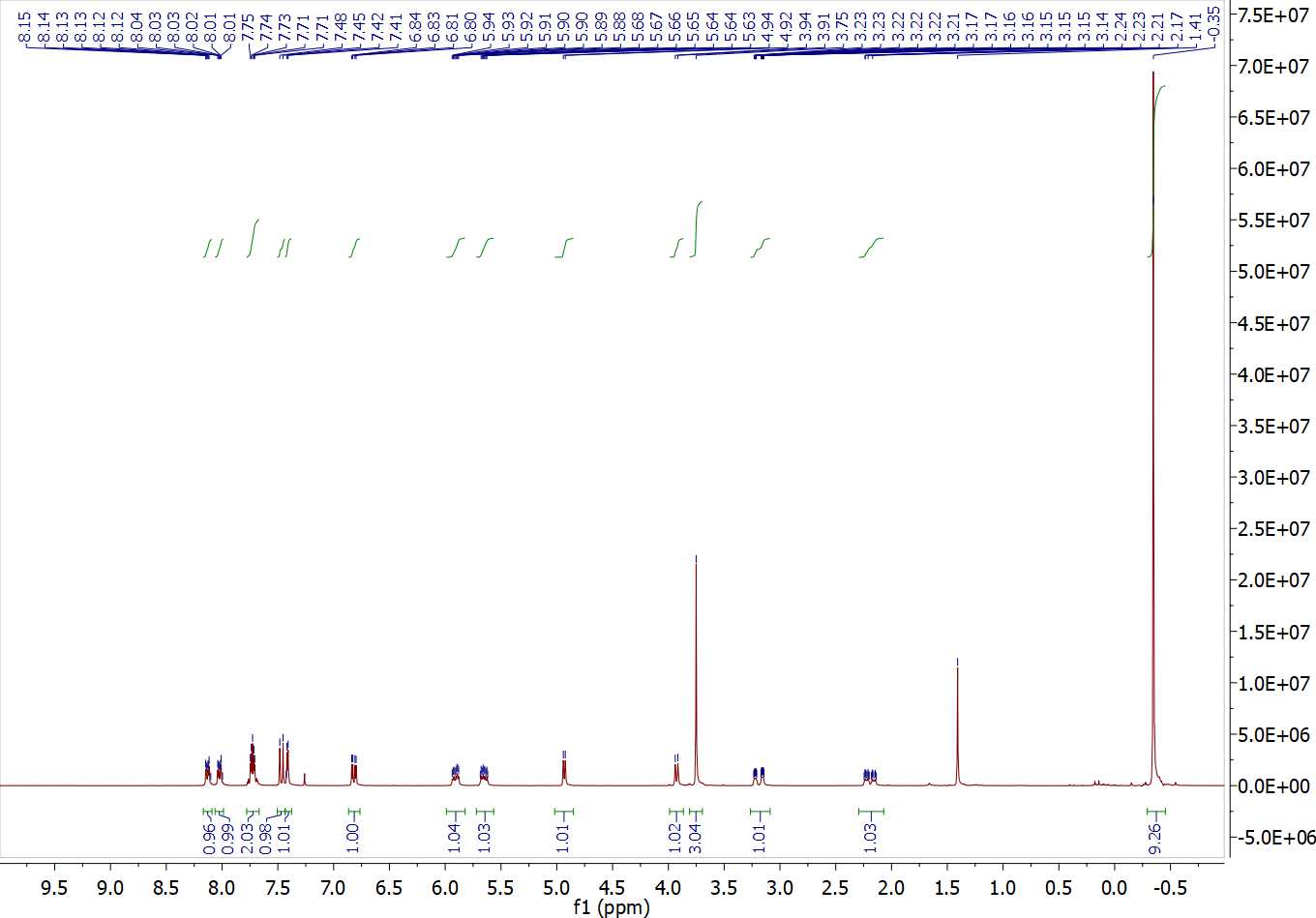


**Figure 63** ^1^H NMR (500 MHz, CDCl_3_) **9cd** Br MeO TMS DA F1.


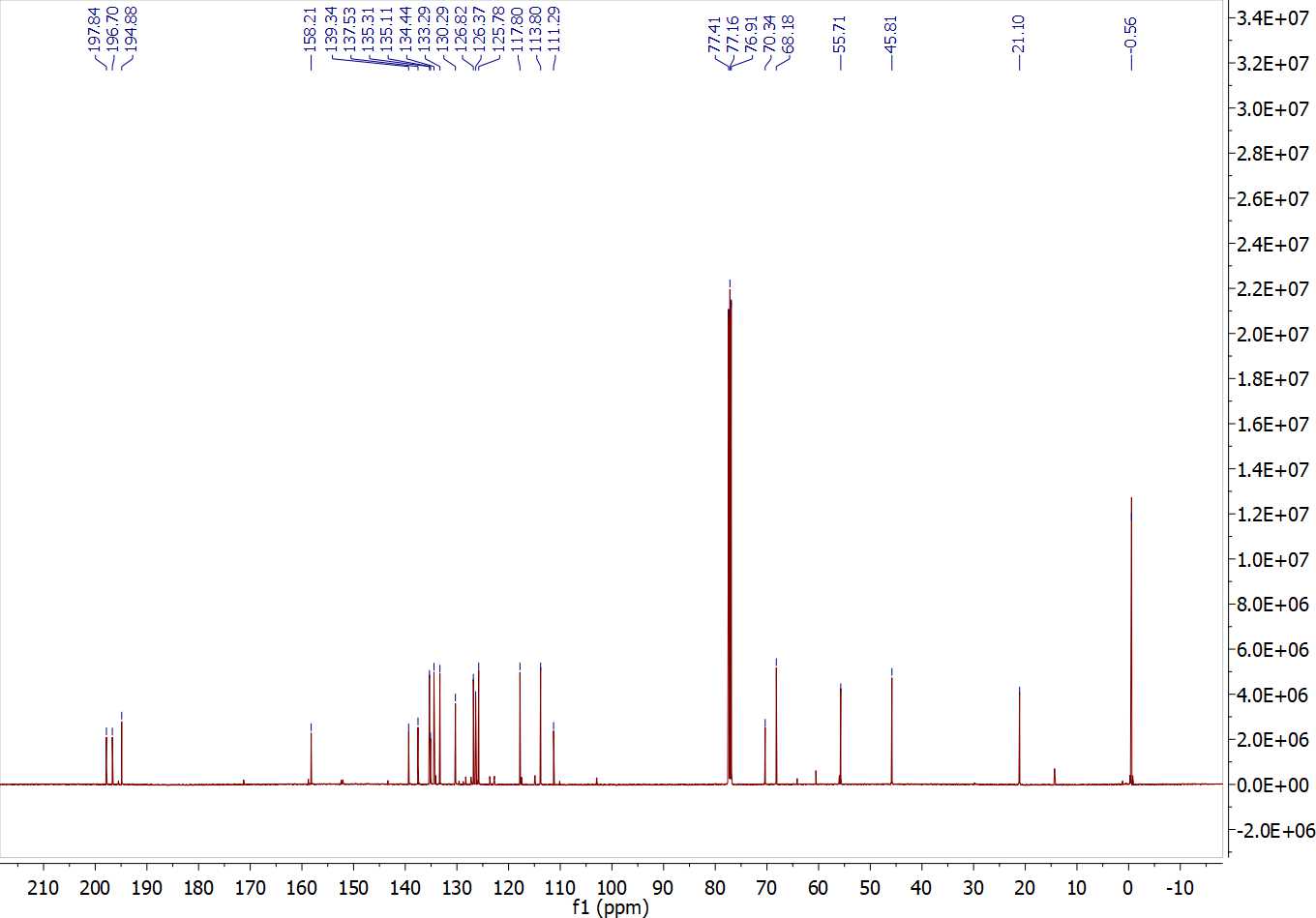


**Figure 64** ^13^C NMR (126 MHz, CDCl_3_) **9cd** Br MeO TMS DA F1.


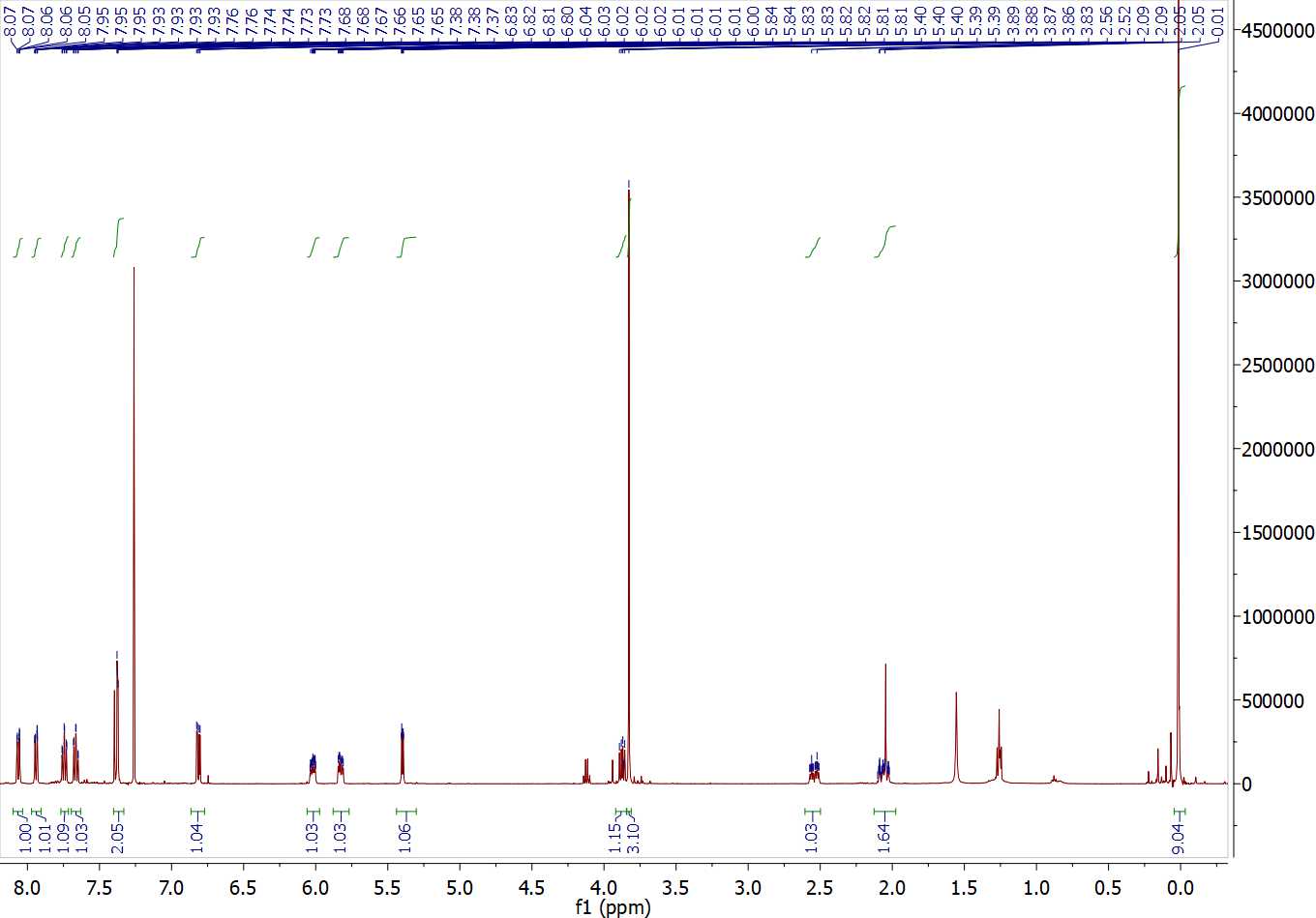


**Figure 65** ^1^H NMR (500 MHz, CDCl_3_) **8cd** Br MeO TMS DA F2.


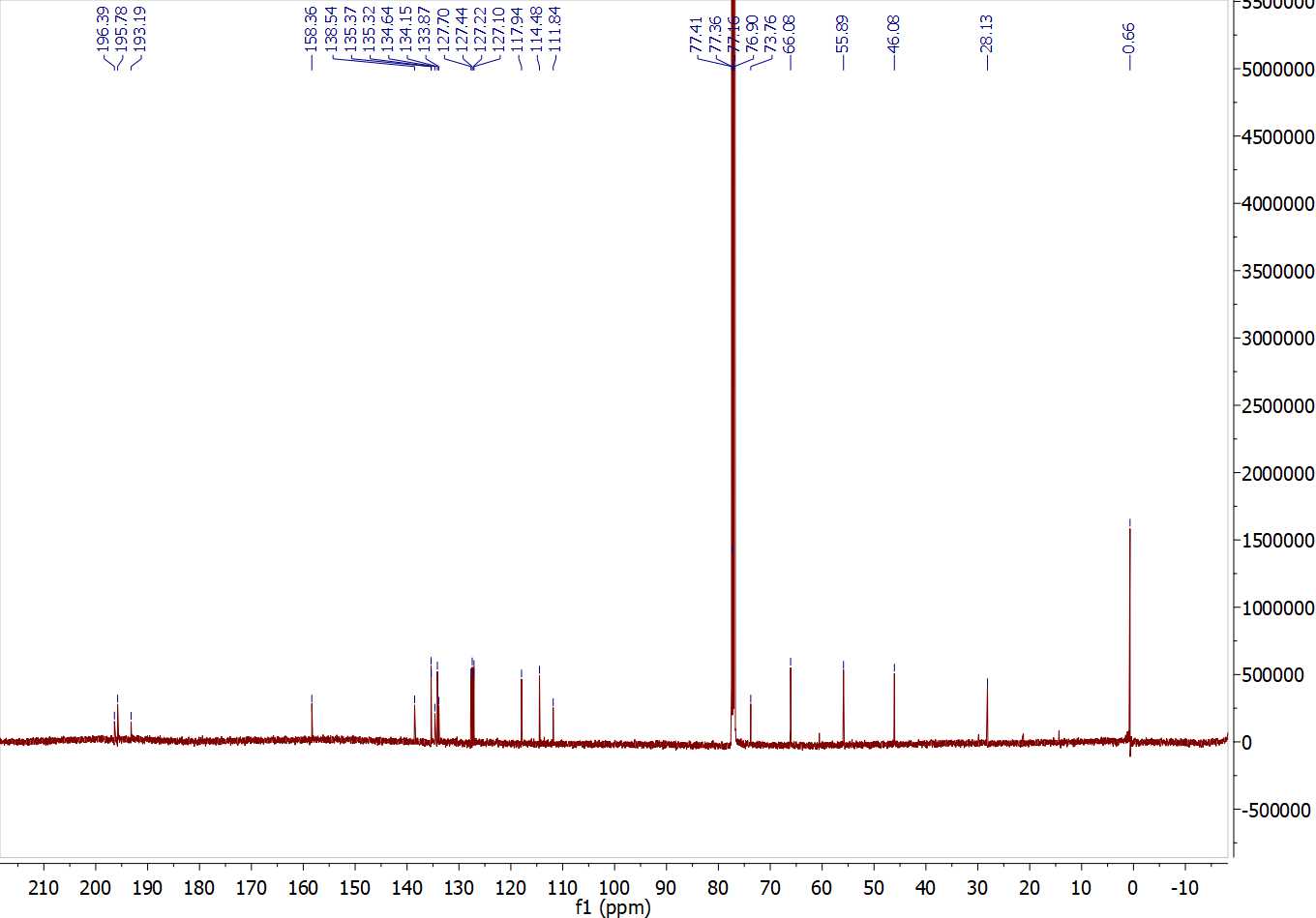


**Figure 66** ^13^C NMR (126 MHz, CDCl_3_) **8cd** Br MeO TMS DA F2.


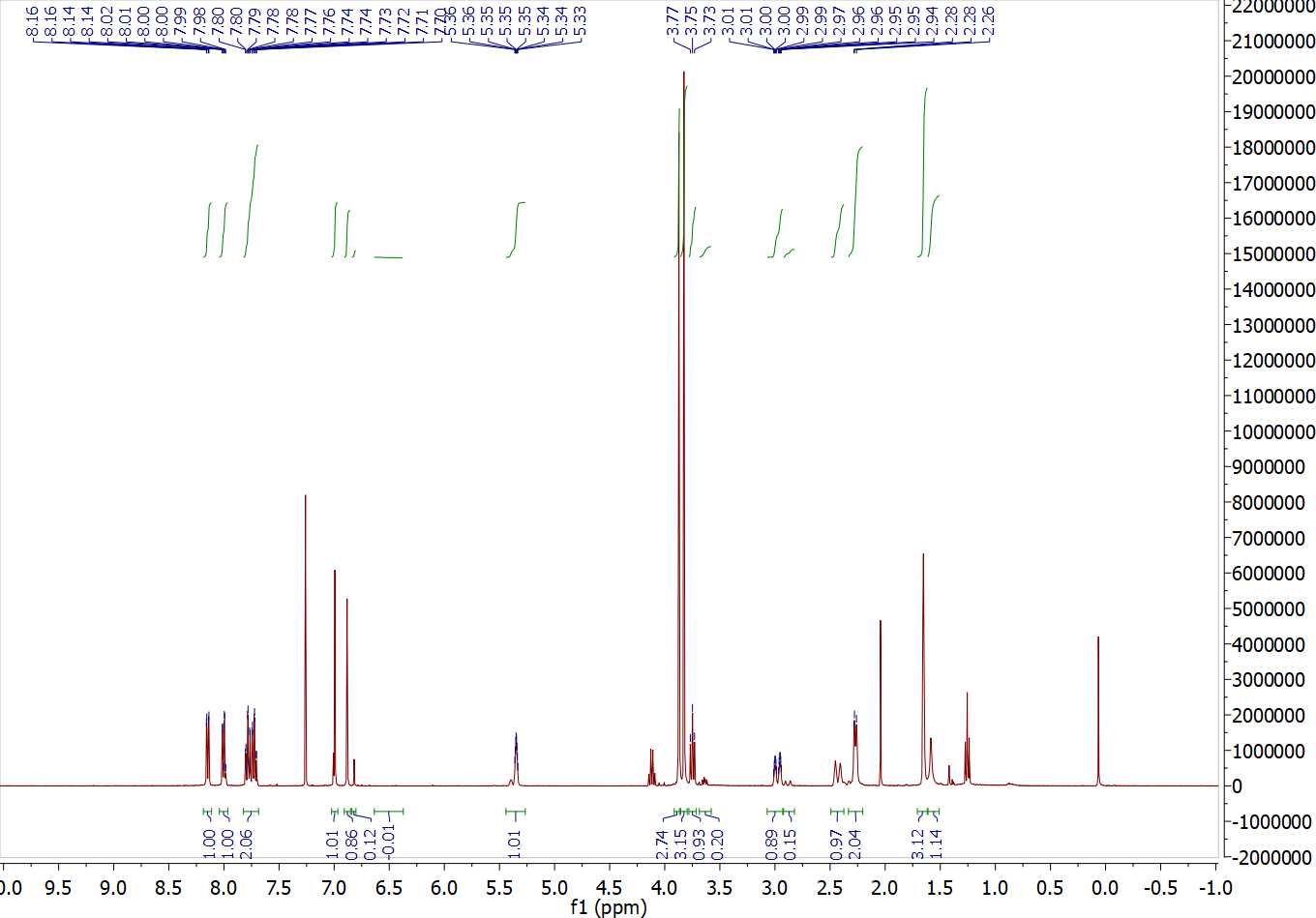


**Figure 67** ^1^H NMR (400 MHz, CDCl_3_) **8db** Br DiMeO Isoprene DA.


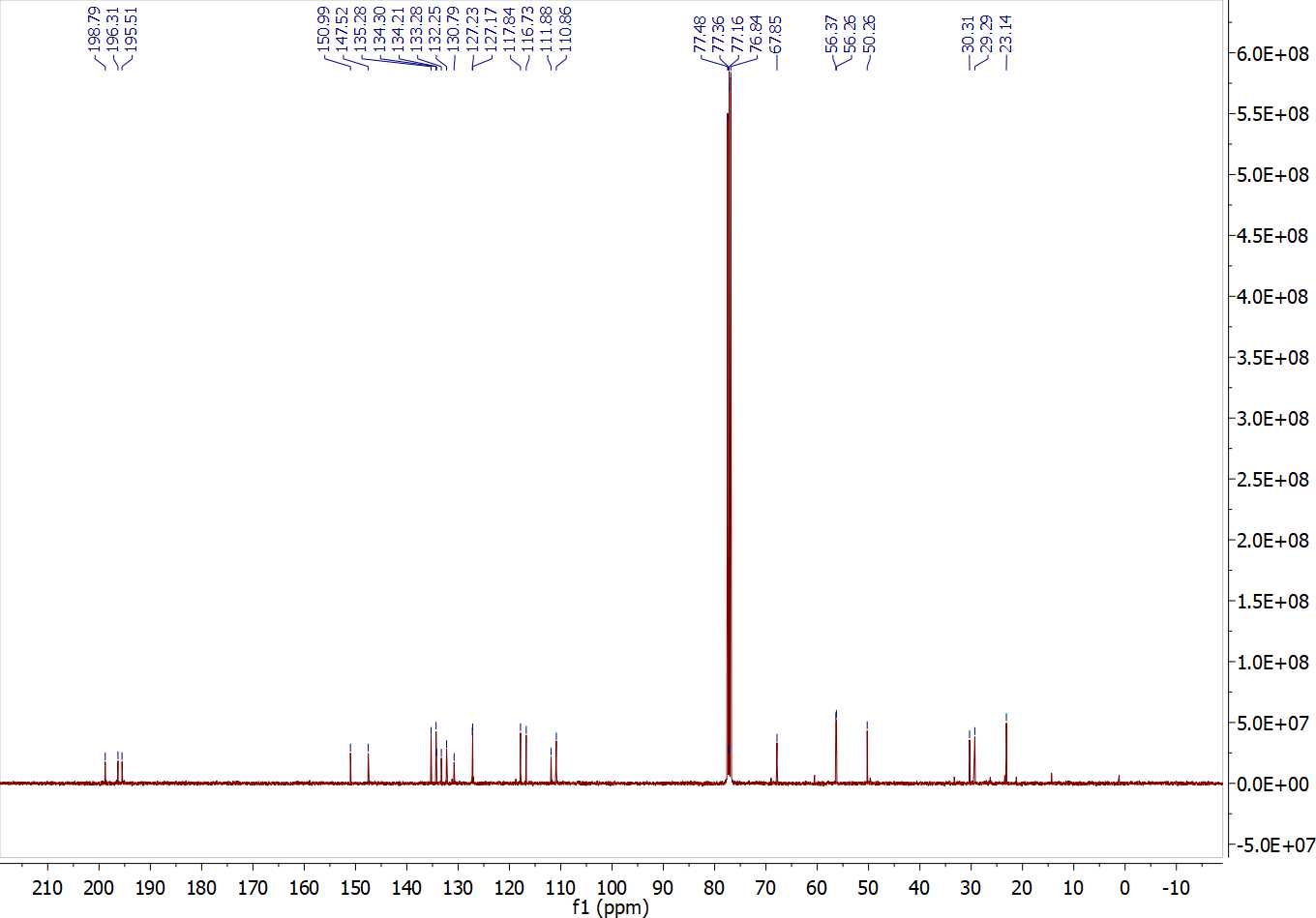


**Figure 68** ^13^C NMR (101 MHz, CDCl_3_) **8db** Br DiMeO Isoprene DA.


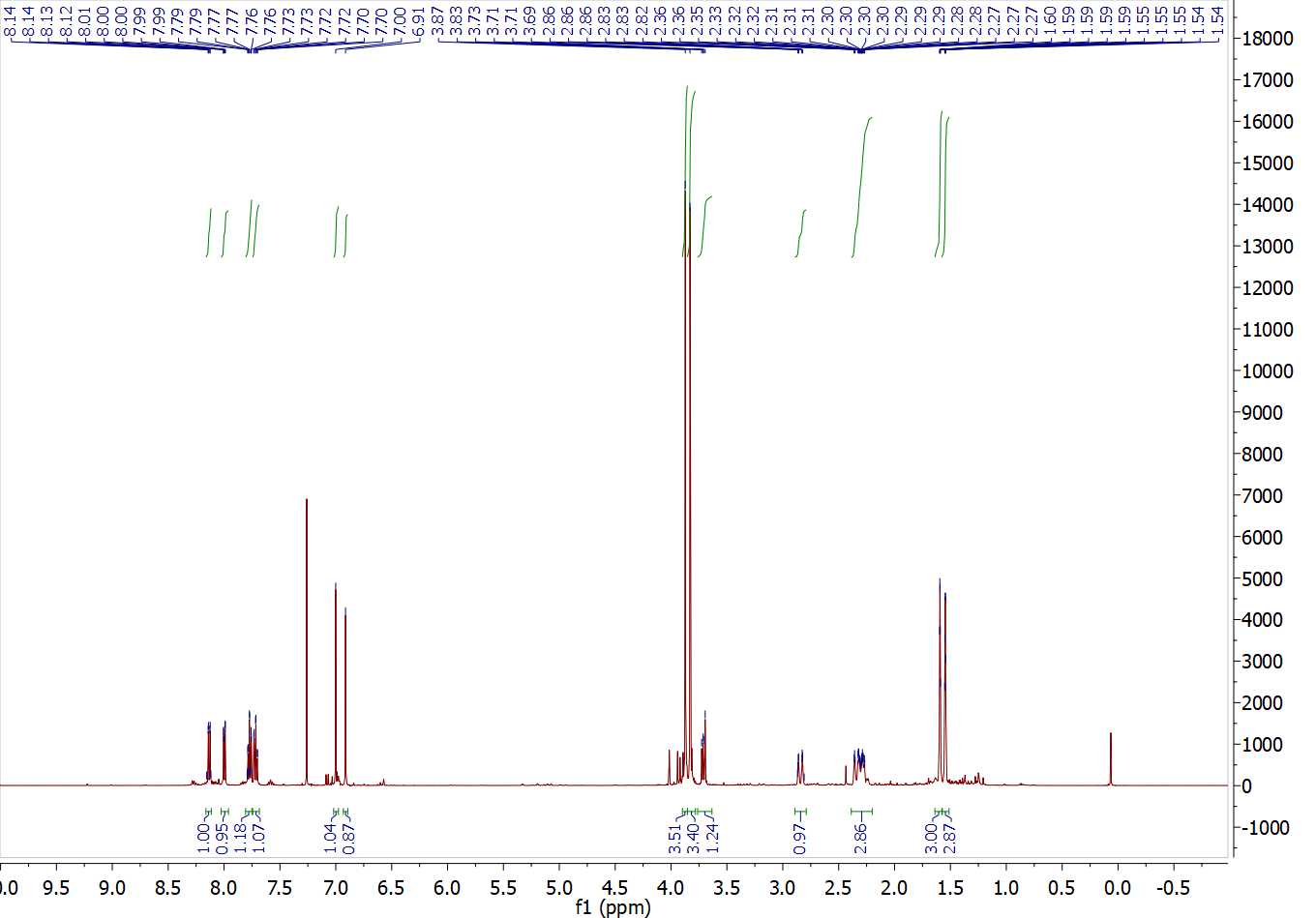


**Figure 69** ^1^H NMR (500 MHz, CDCl_3_) **8dc** Br DiMeO Dimethyl DA.


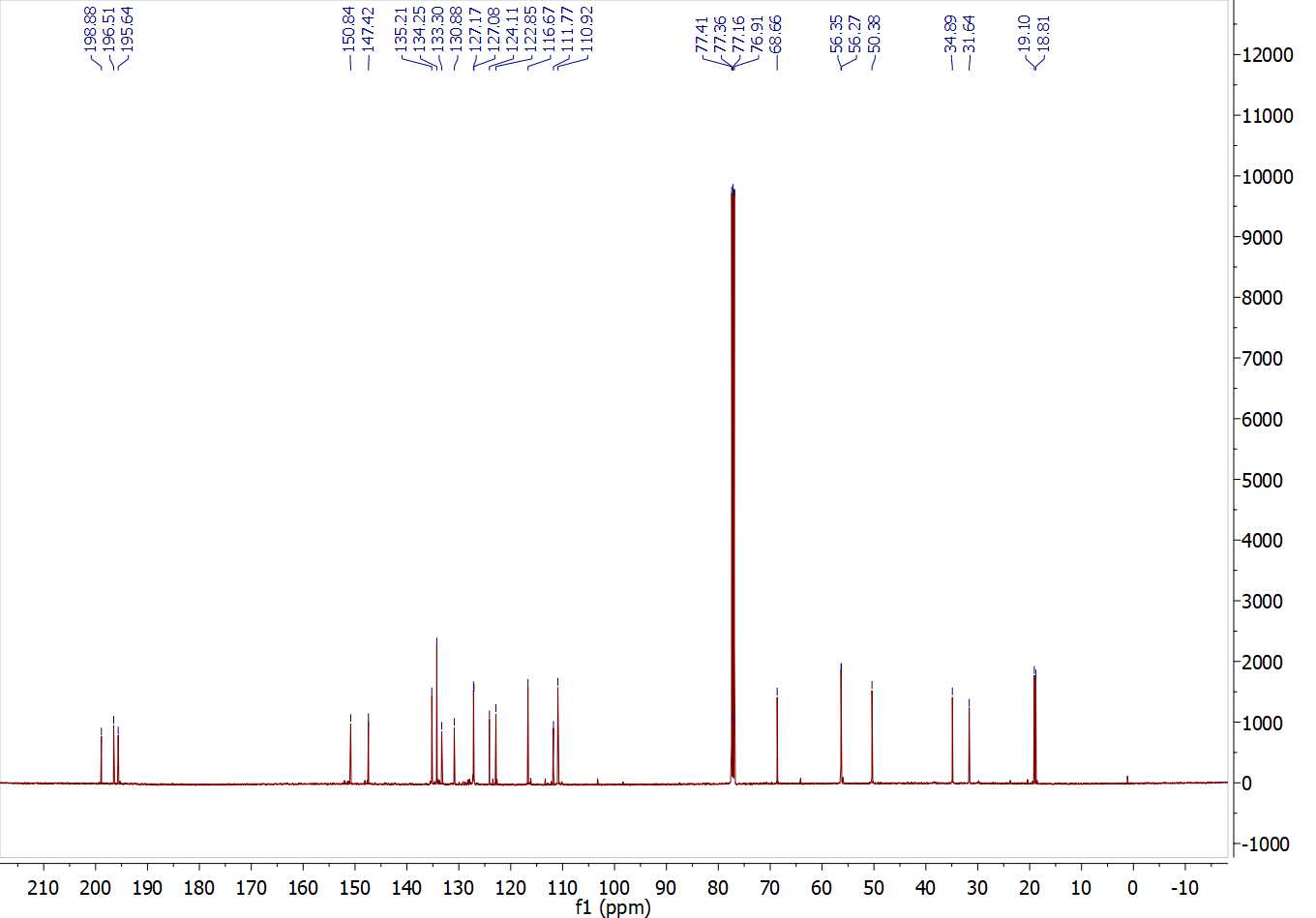


**Figure 70** ^13^C NMR (126 MHz, CDCl_3_) **8dc** Br DiMeO Dimethyl DA.


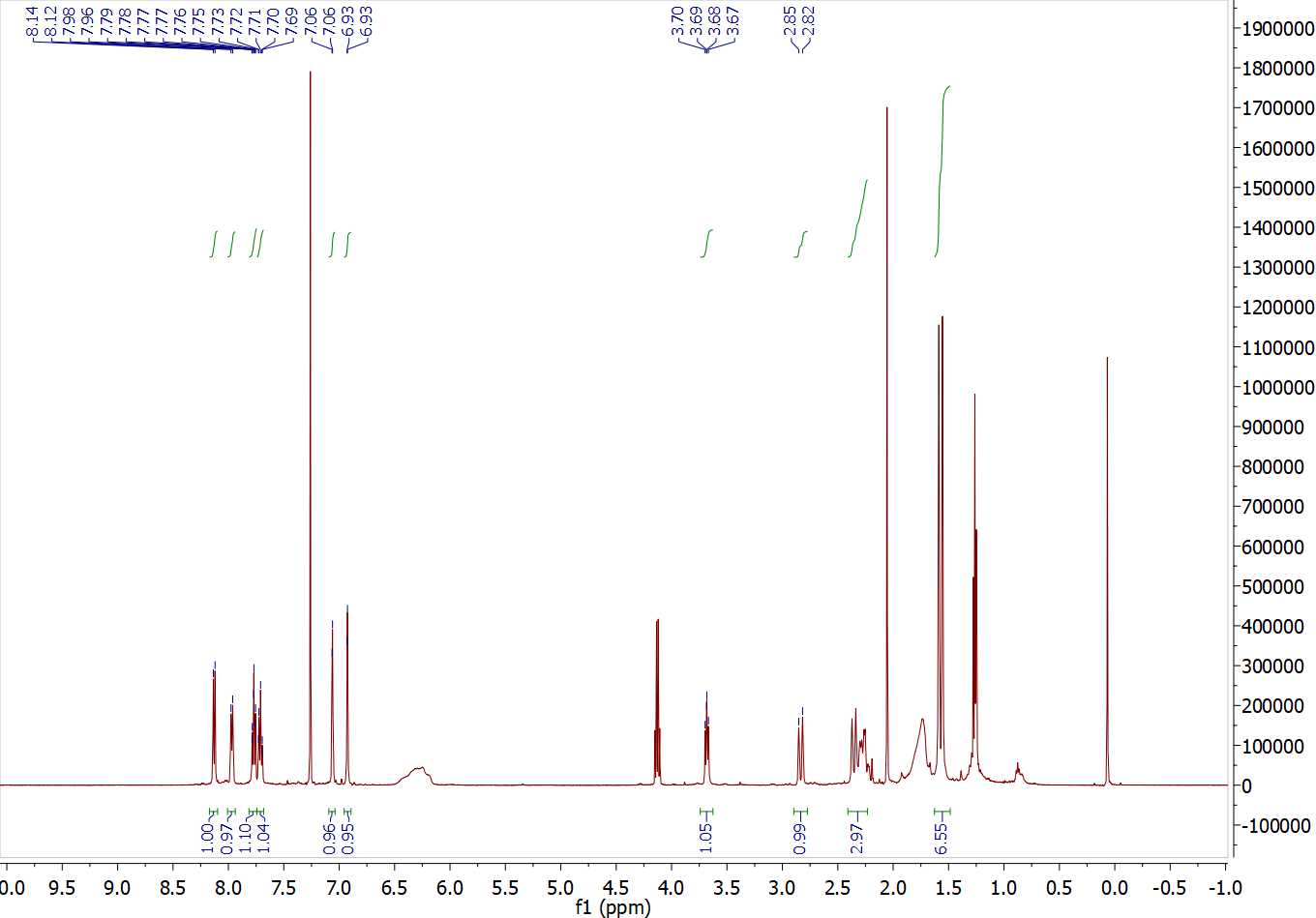


**Figure 71** ^1^H NMR (500 MHz, CDCl_3_) **8ec** Br DiOH Dimethyl DA.


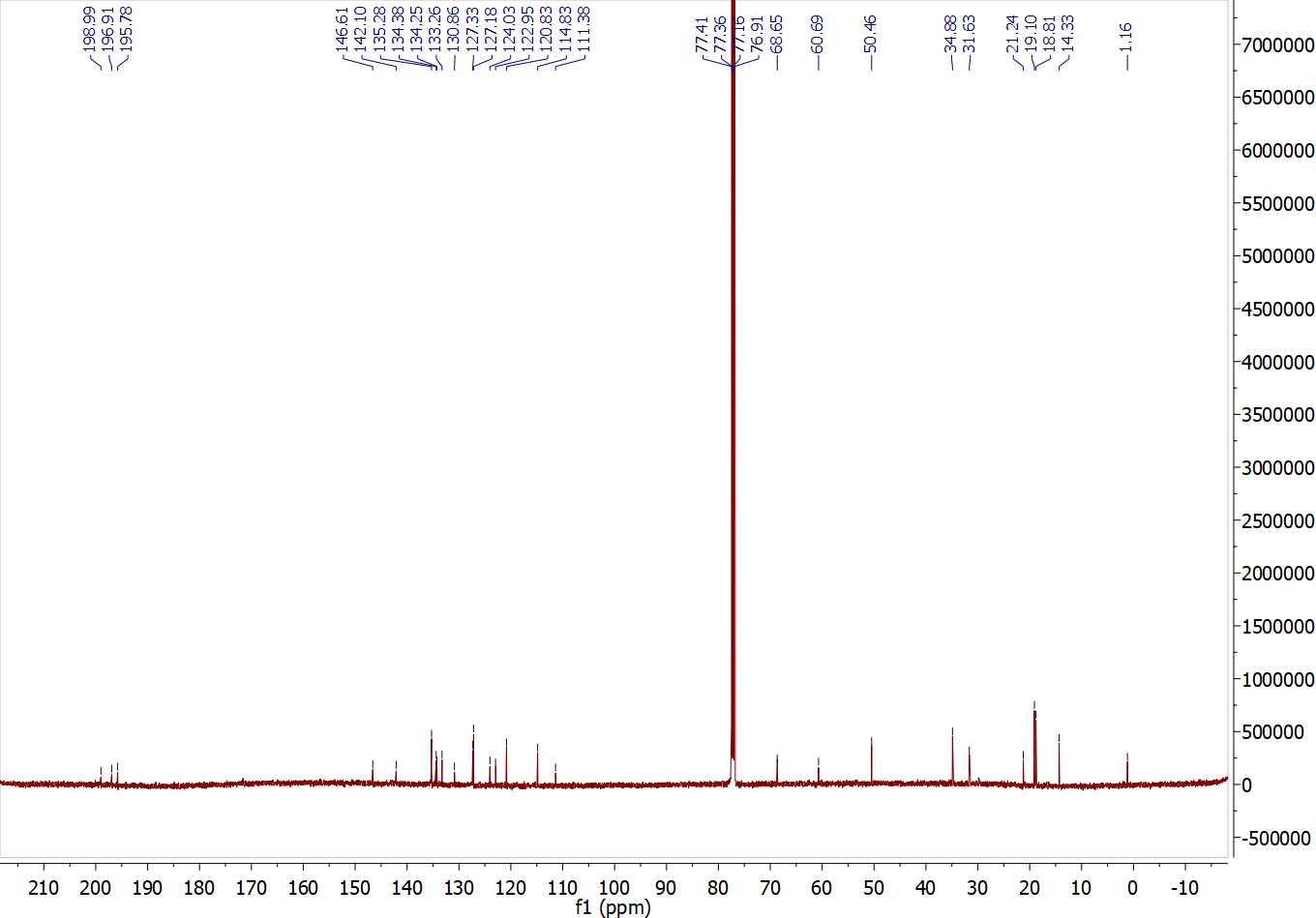


**Figure 72** ^13^C NMR (126 MHz, CDCl_3_) **8ec** Br DiOH Dimethyl DA.


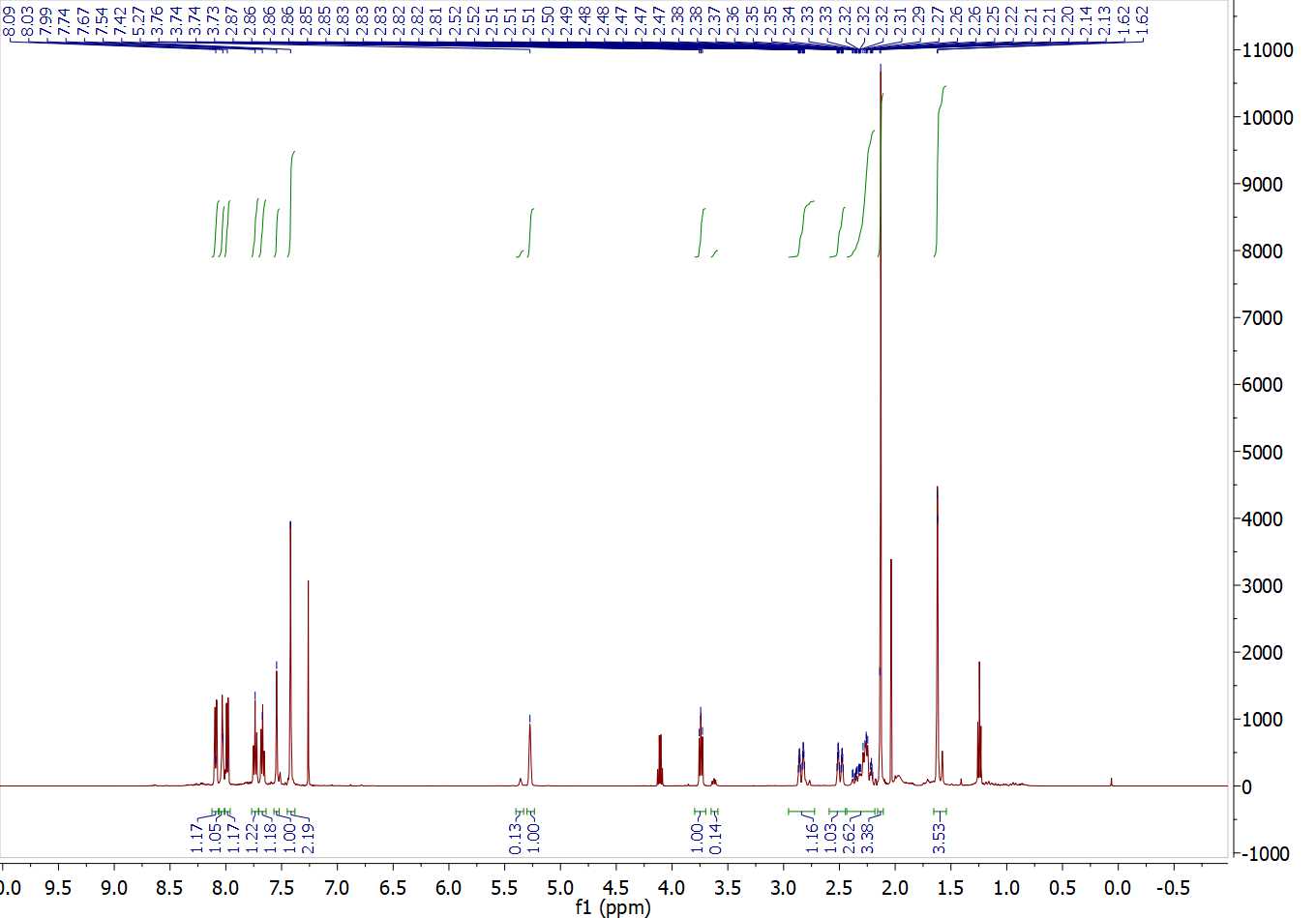


**Figure 73** ^1^H NMR (500 MHz, CDCl_3_) **8fb** Br NAc Isoprene DA.


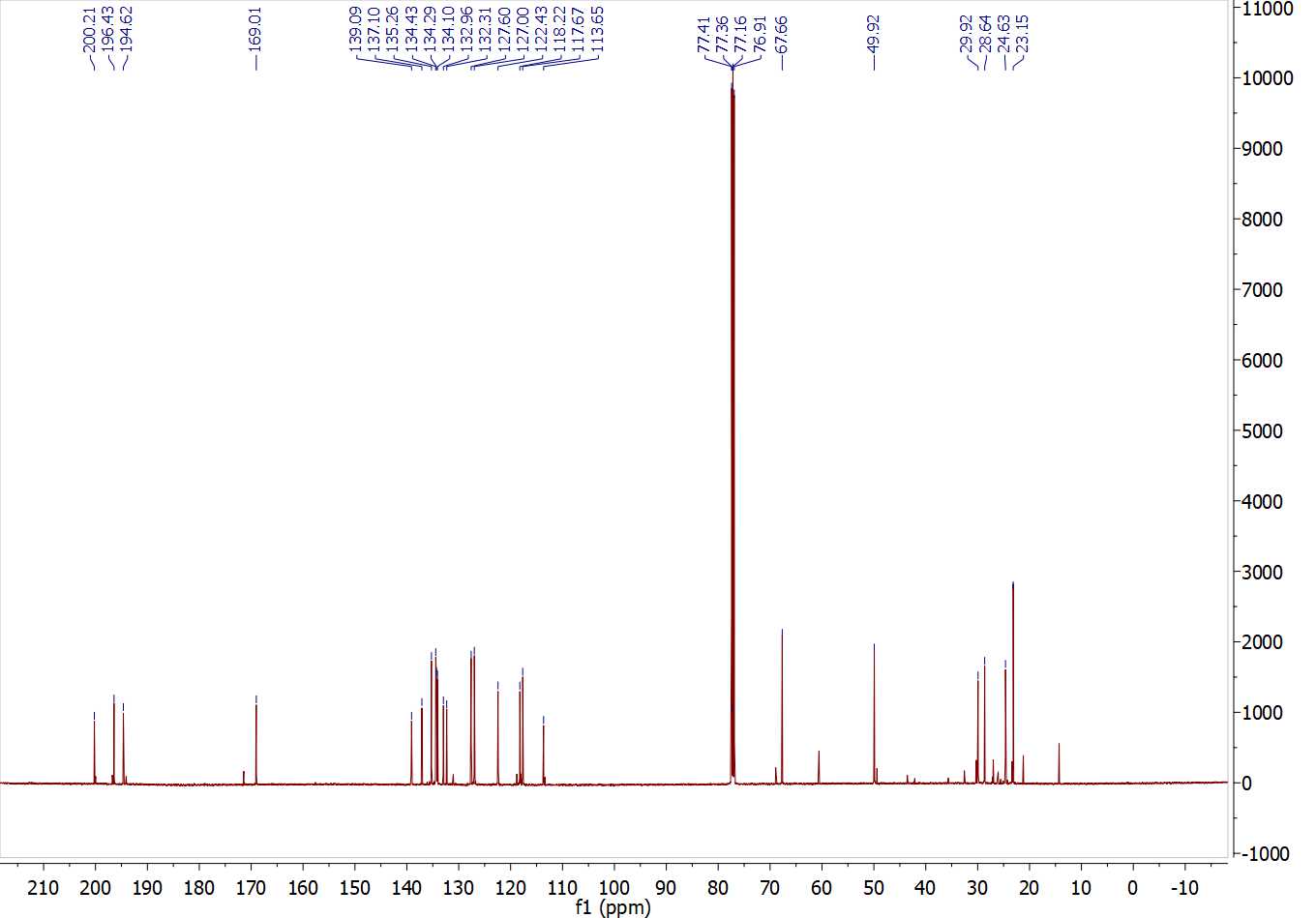


**Figure 74** ^13^C NMR (126 MHz, CDCl_3_) **8fb** Br NAc Isoprene DA.


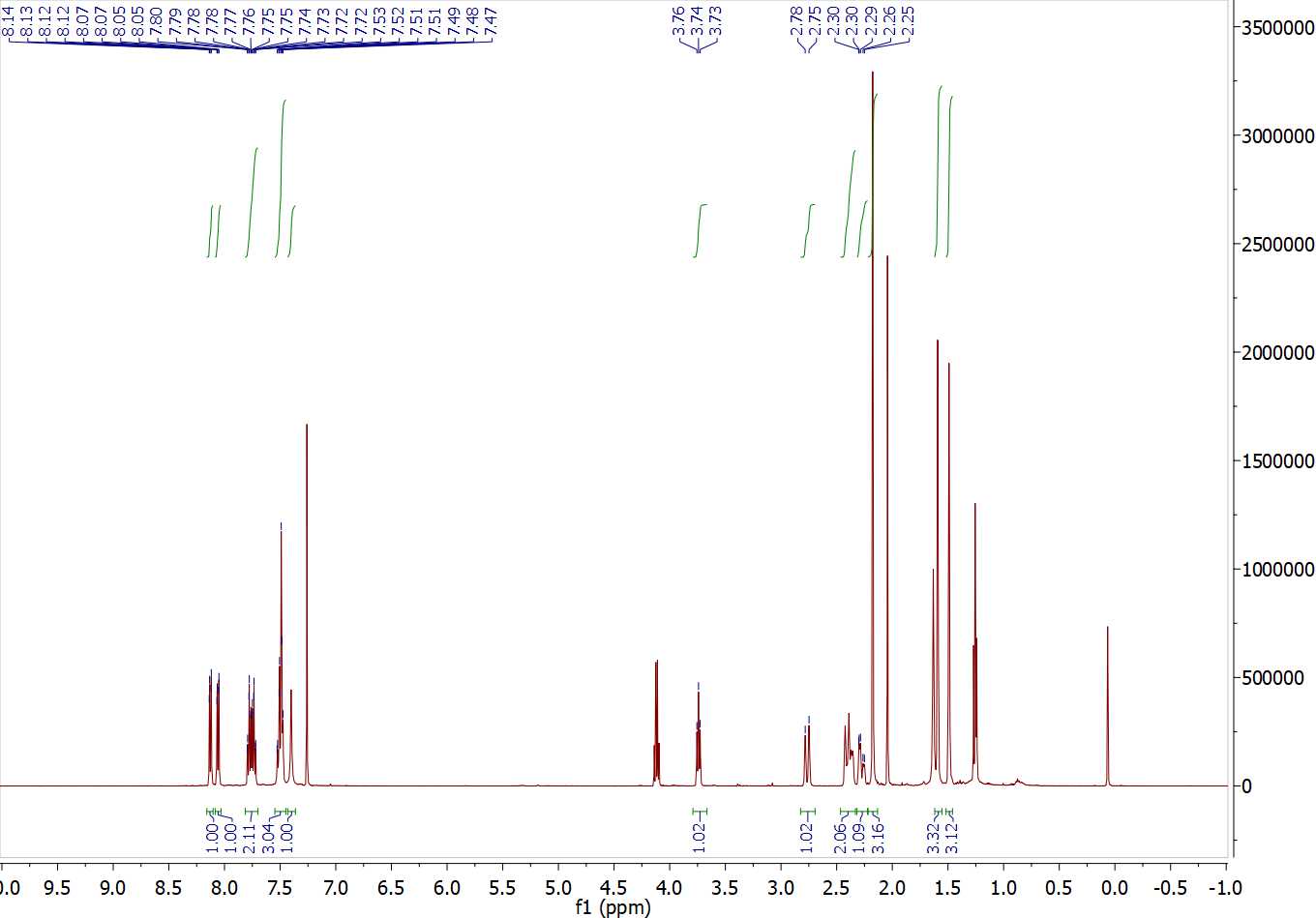


**Figure 75** ^1^H NMR (500 MHz, CDCl_3_) **8fc** Br NAc Dimethyl DA.


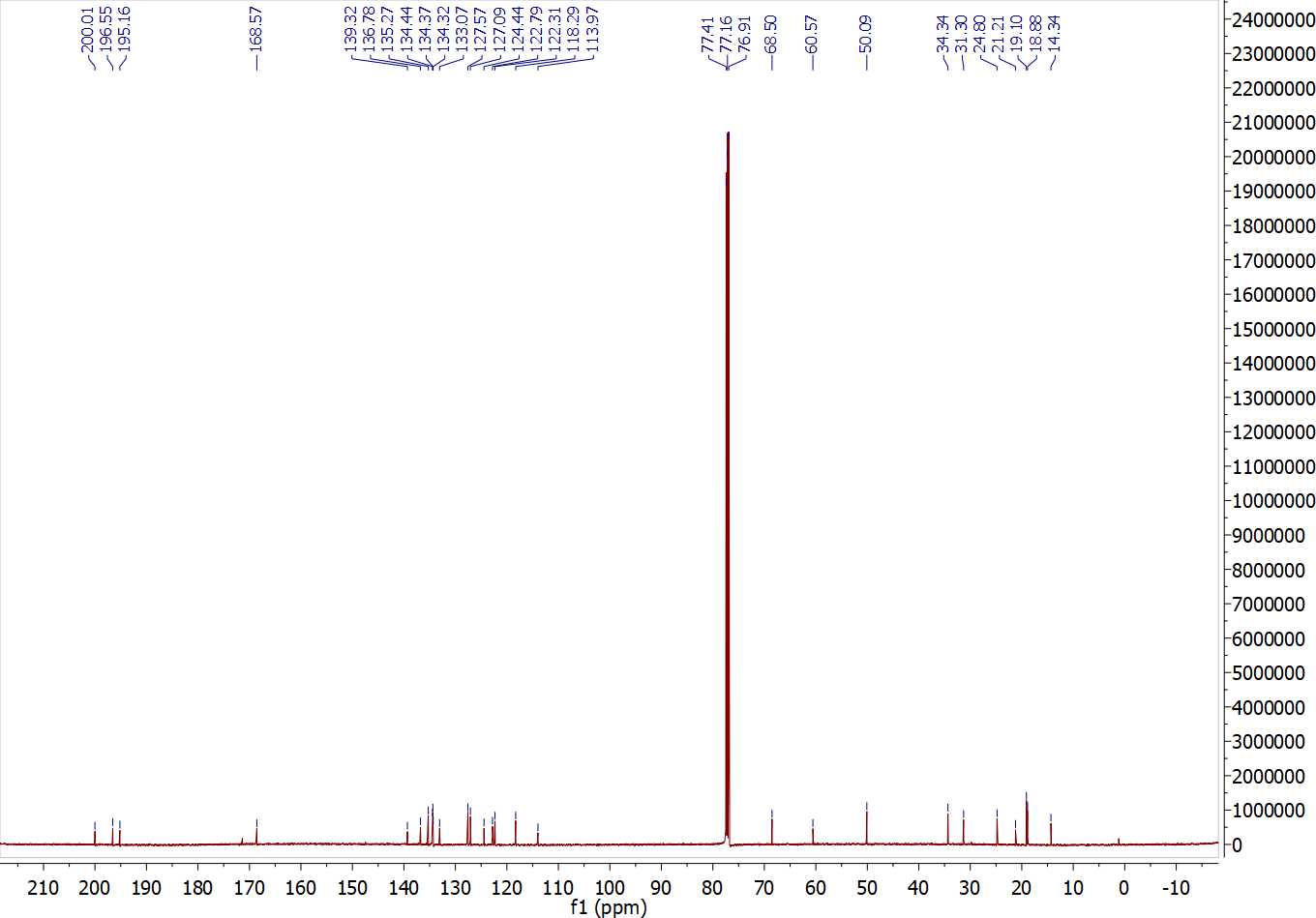


**Figure 76** ^13^C NMR (126 MHz, CDCl_3_) **8fc** Br NAc Dimethyl DA.


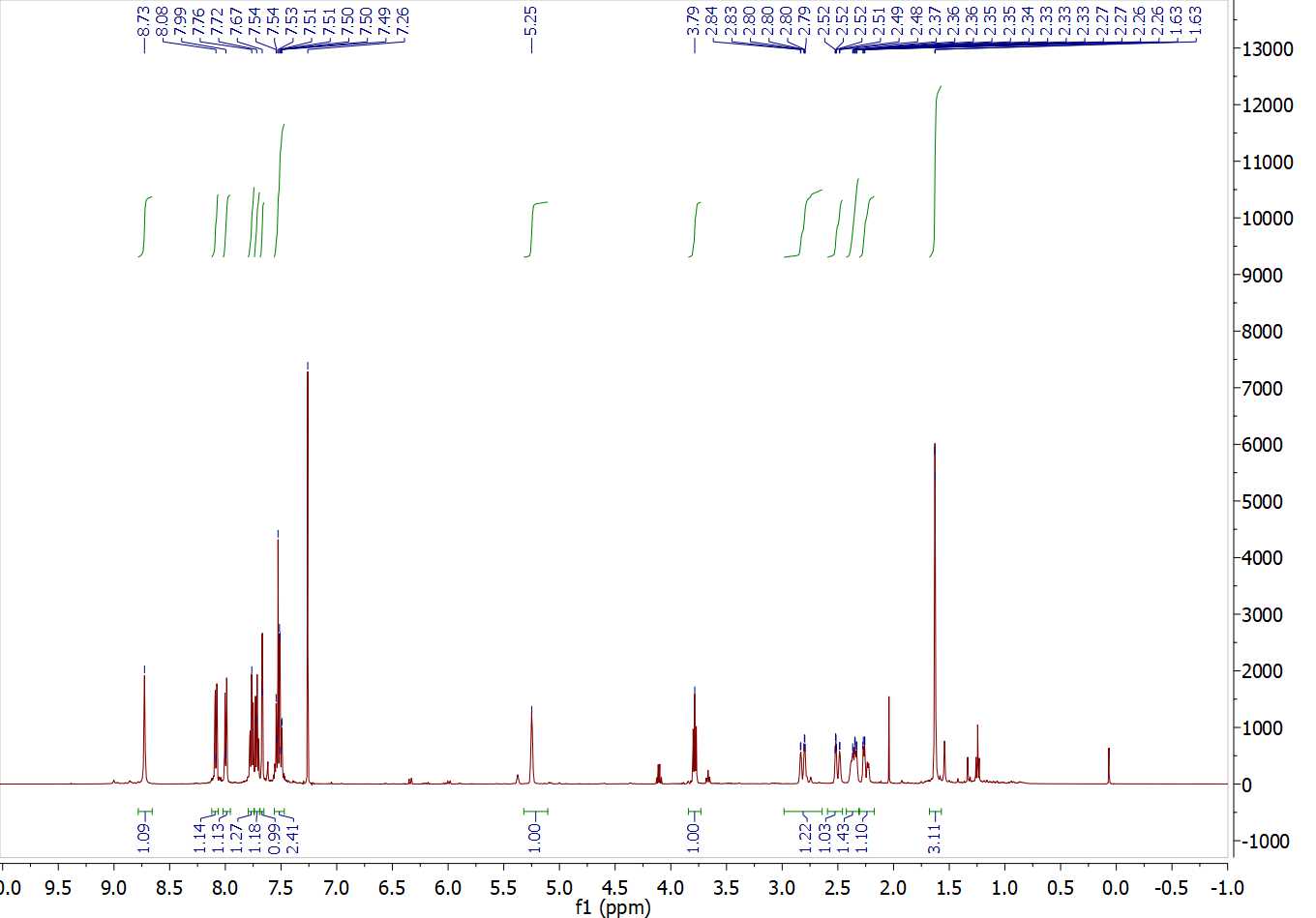


**Figure 77** ^1^H NMR (500 MHz, CDCl_3_) **8gb** Br CF_3_ Isoprene DA.


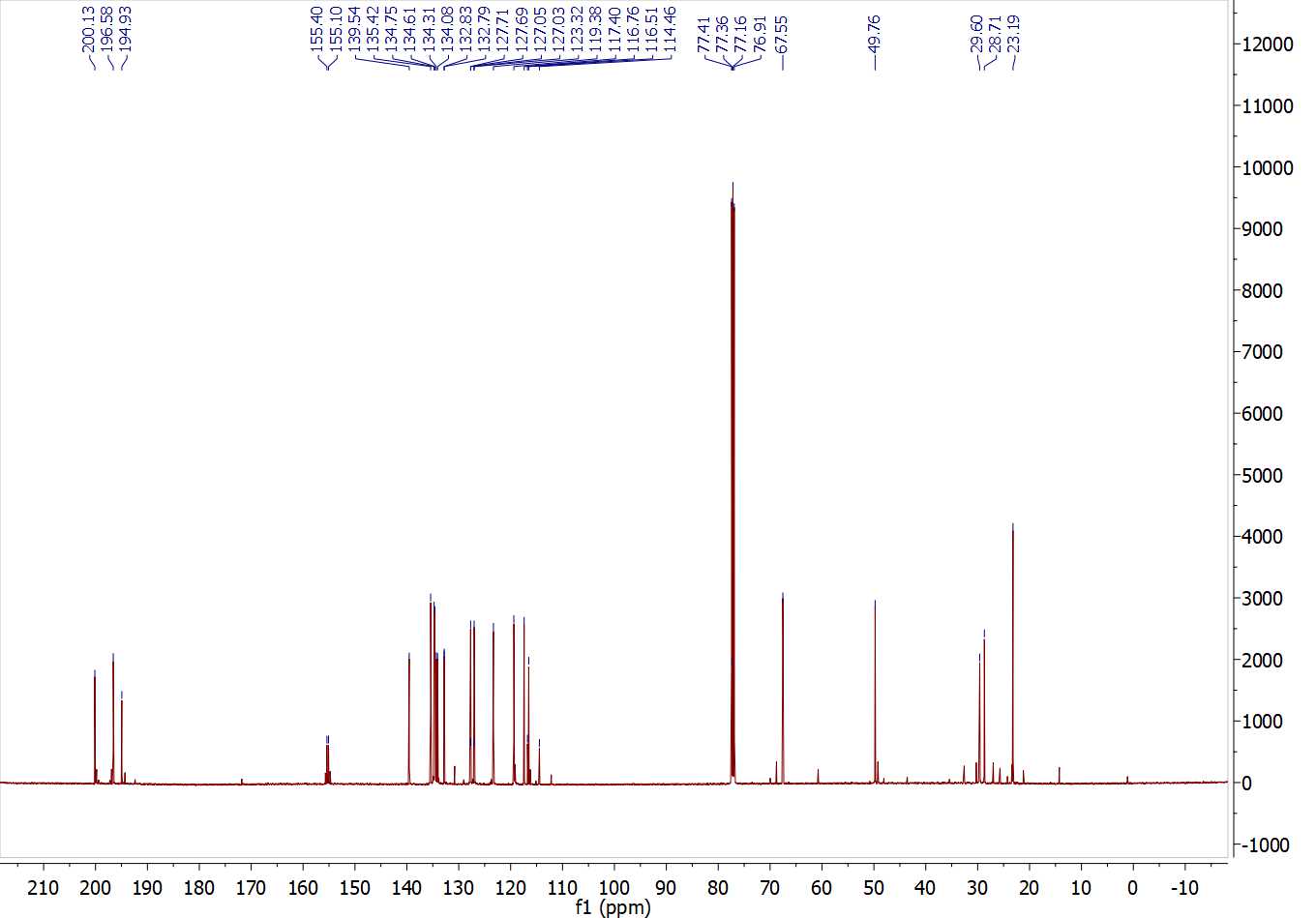


**Figure 78** ^13^C NMR (126 MHz, CDCl_3_) **8gb** Br CF_3_ Isoprene DA.


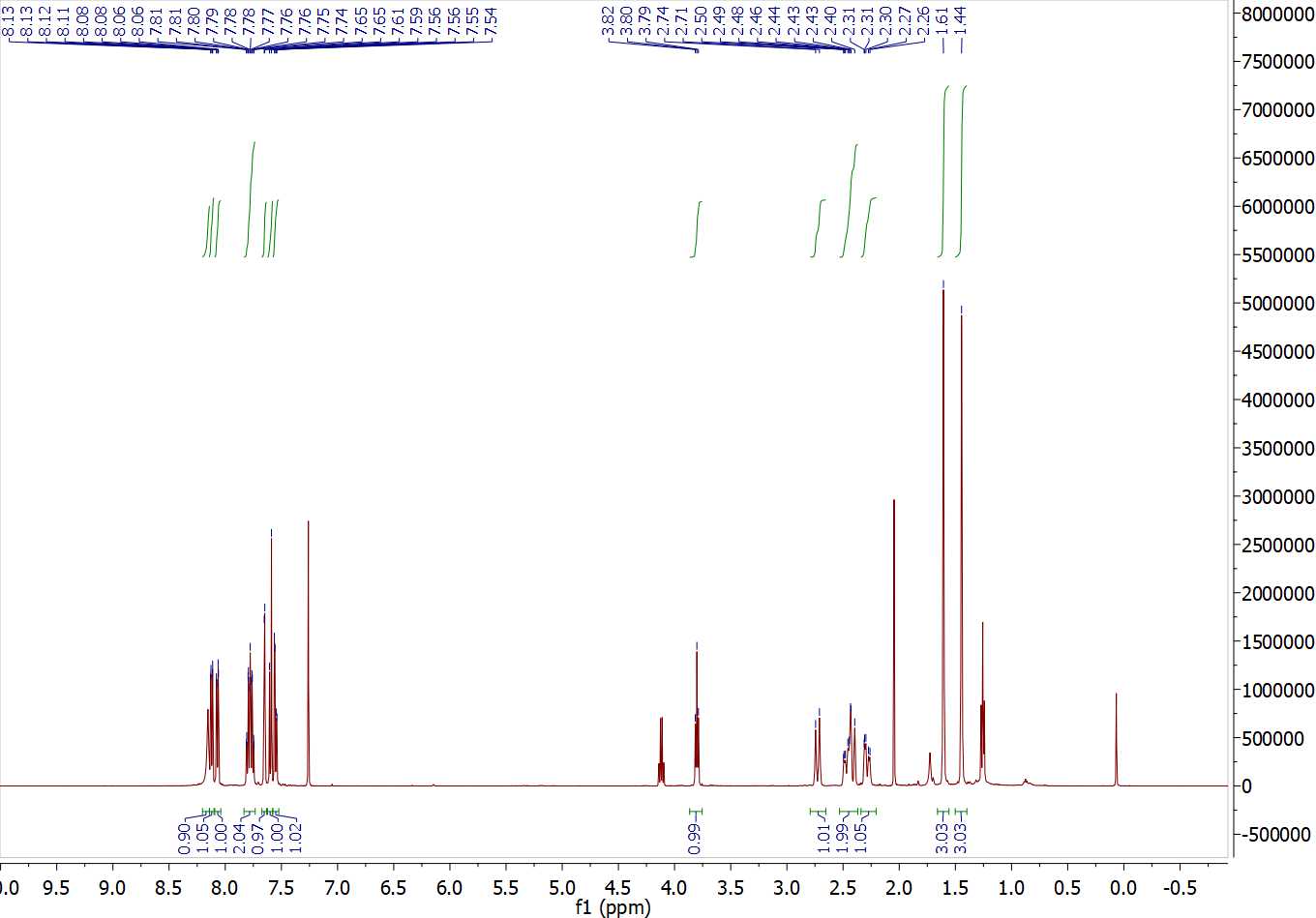


**Figure 79** ^1^H NMR (500 MHz, CDCl_3_) **8gc** Br CF_3_ Dimethyl DA.


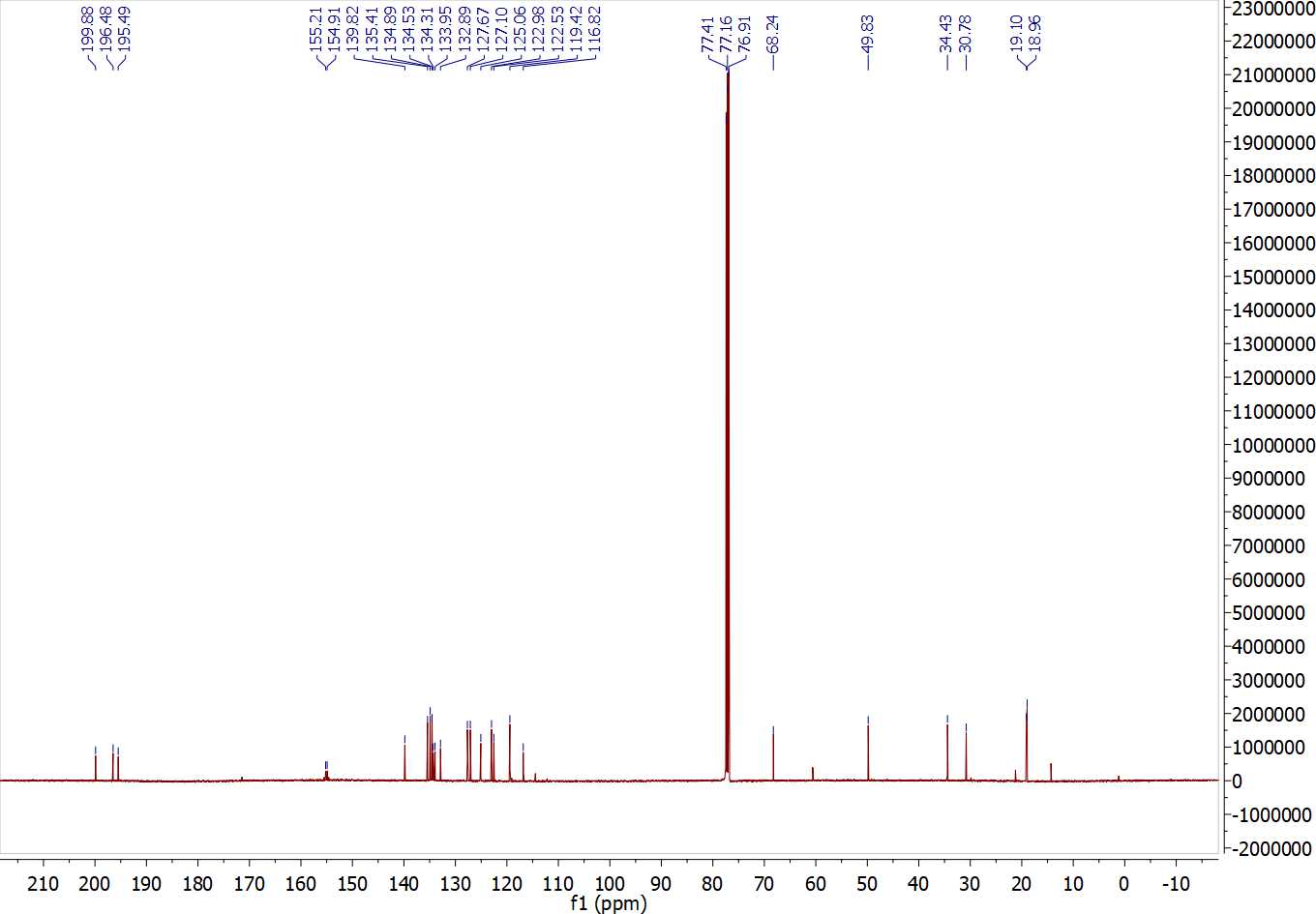


**Figure 80** ^13^C NMR (126 MHz, CDCl_3_) **8gc** Br CF_3_ Dimethyl DA.


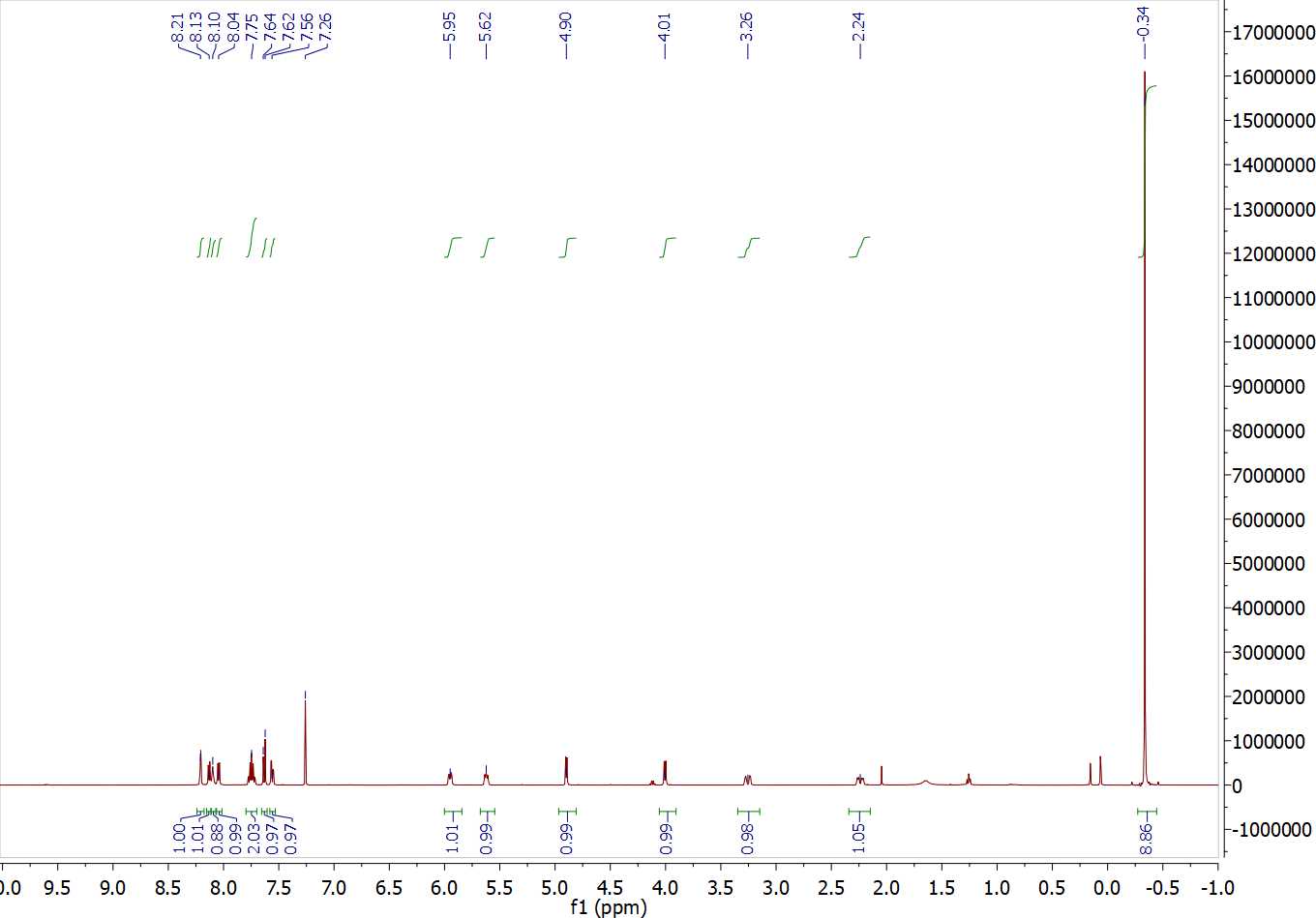


**Figure 81** ^1^H NMR (500 MHz, CDCl_3_) **9gd** Br CF_3_ TMS DA F1.


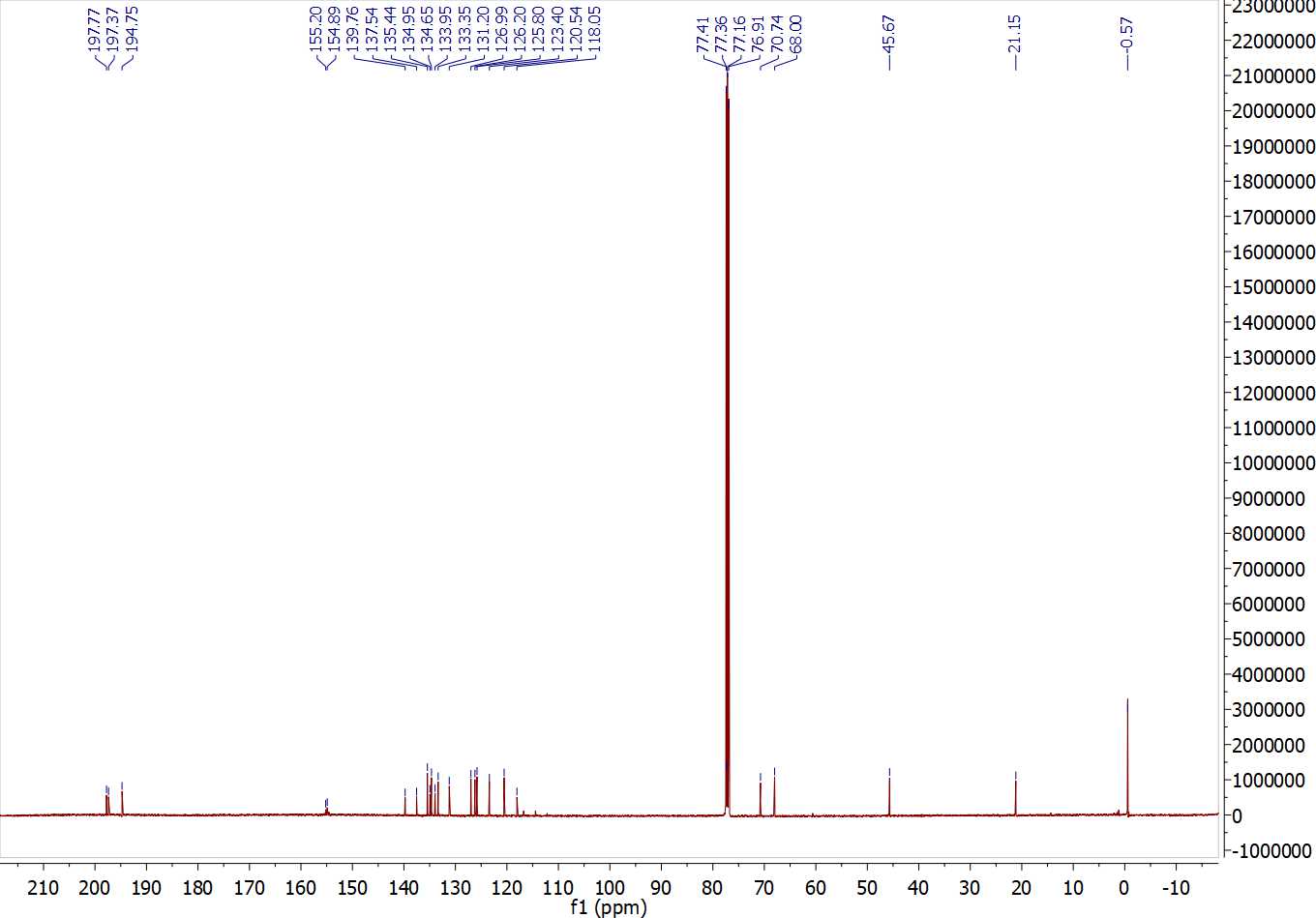


**Figure 82** ^13^C NMR (126 MHz, CDCl_3_) **9gd** Br CF_3_ TMS DA F1.


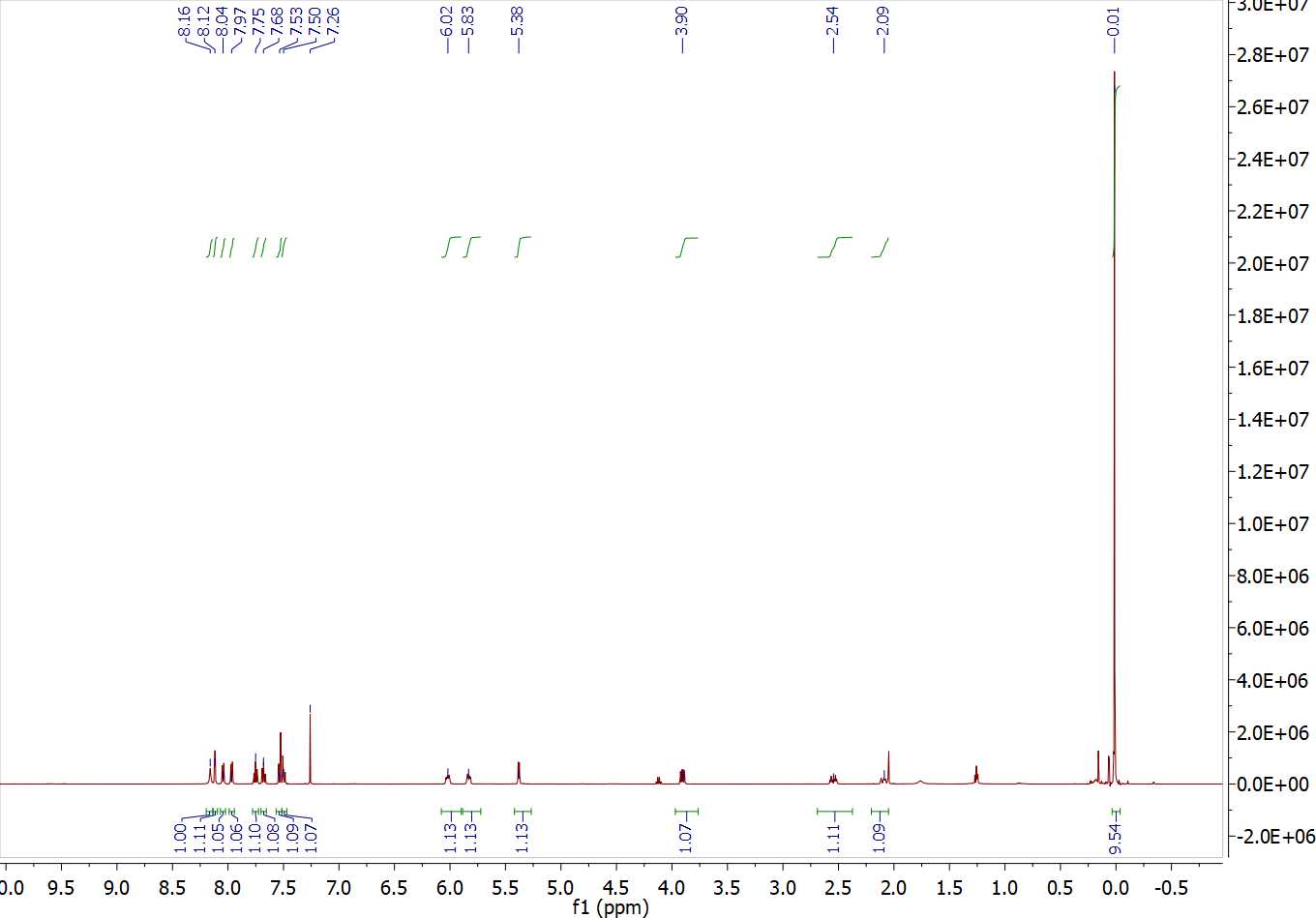


**Figure 83** ^1^H NMR (500 MHz, CDCl_3_) **8gd** Br CF_3_ TMS DA F2.


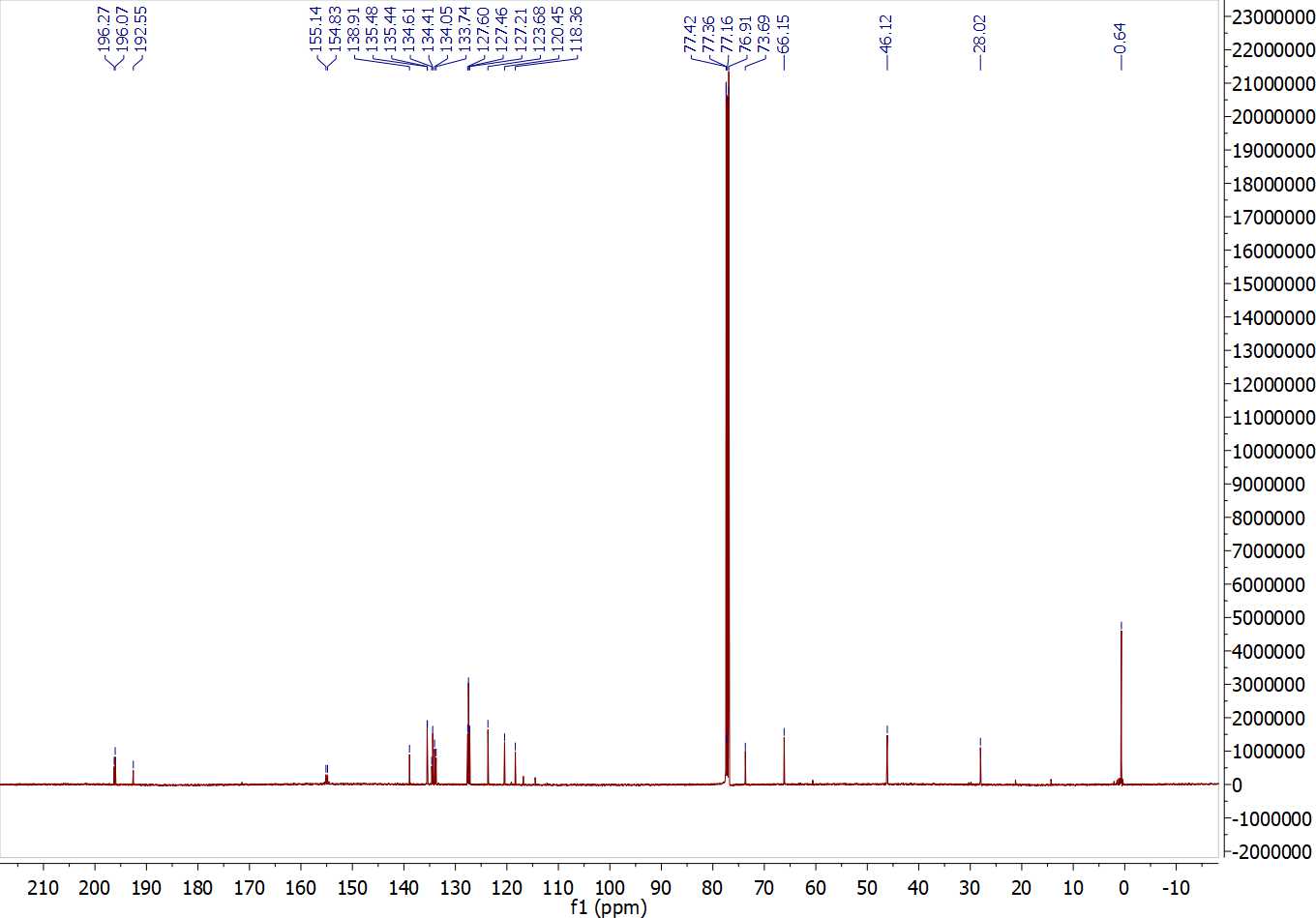


**Figure 84** ^13^C NMR (126 MHz, CDCl_3_) **8gd** Br CF_3_ TMS DA F2.


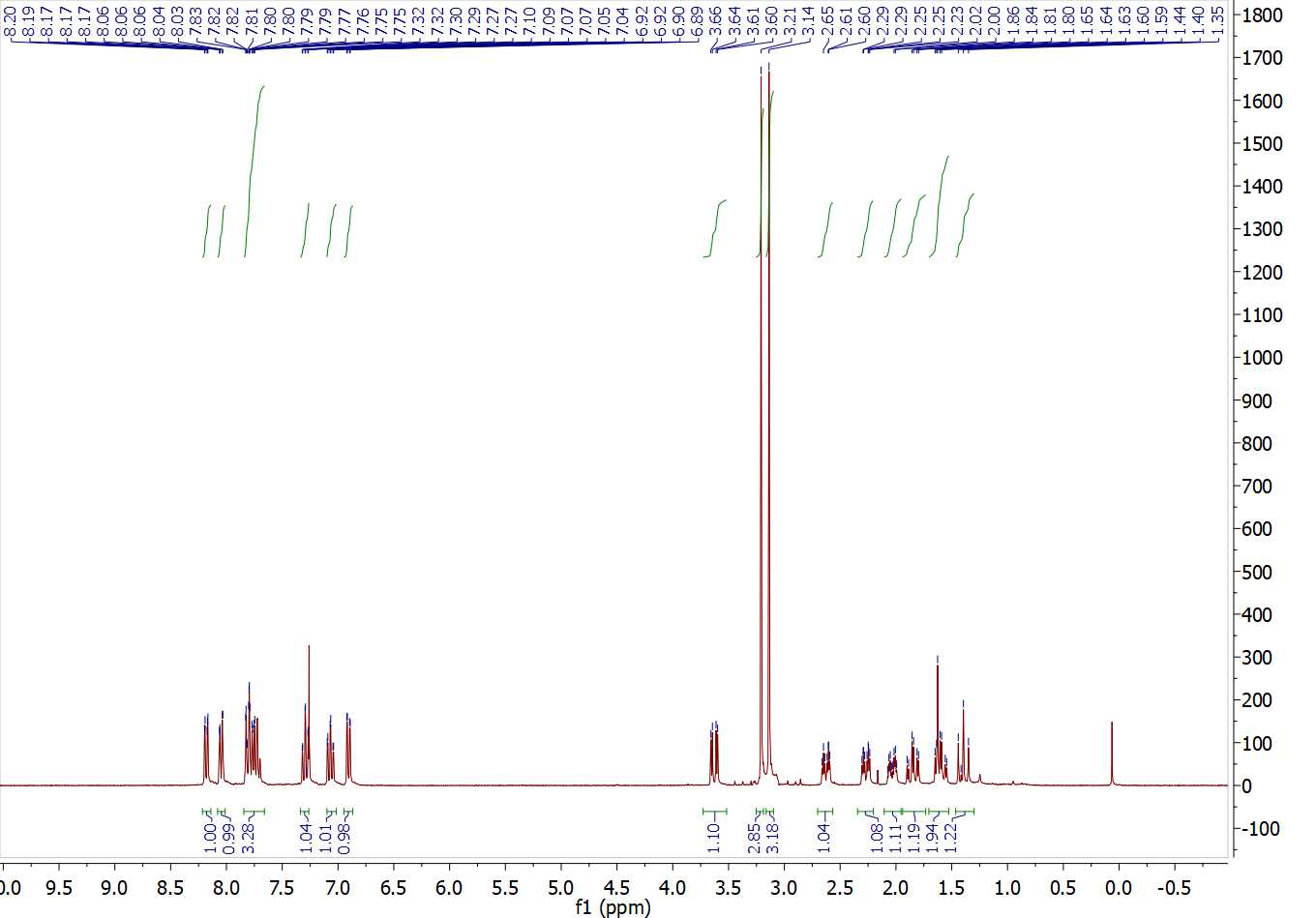


**Figure 85** ^1^H NMR (300 MHz, CDCl_3_) **10** Acetal.


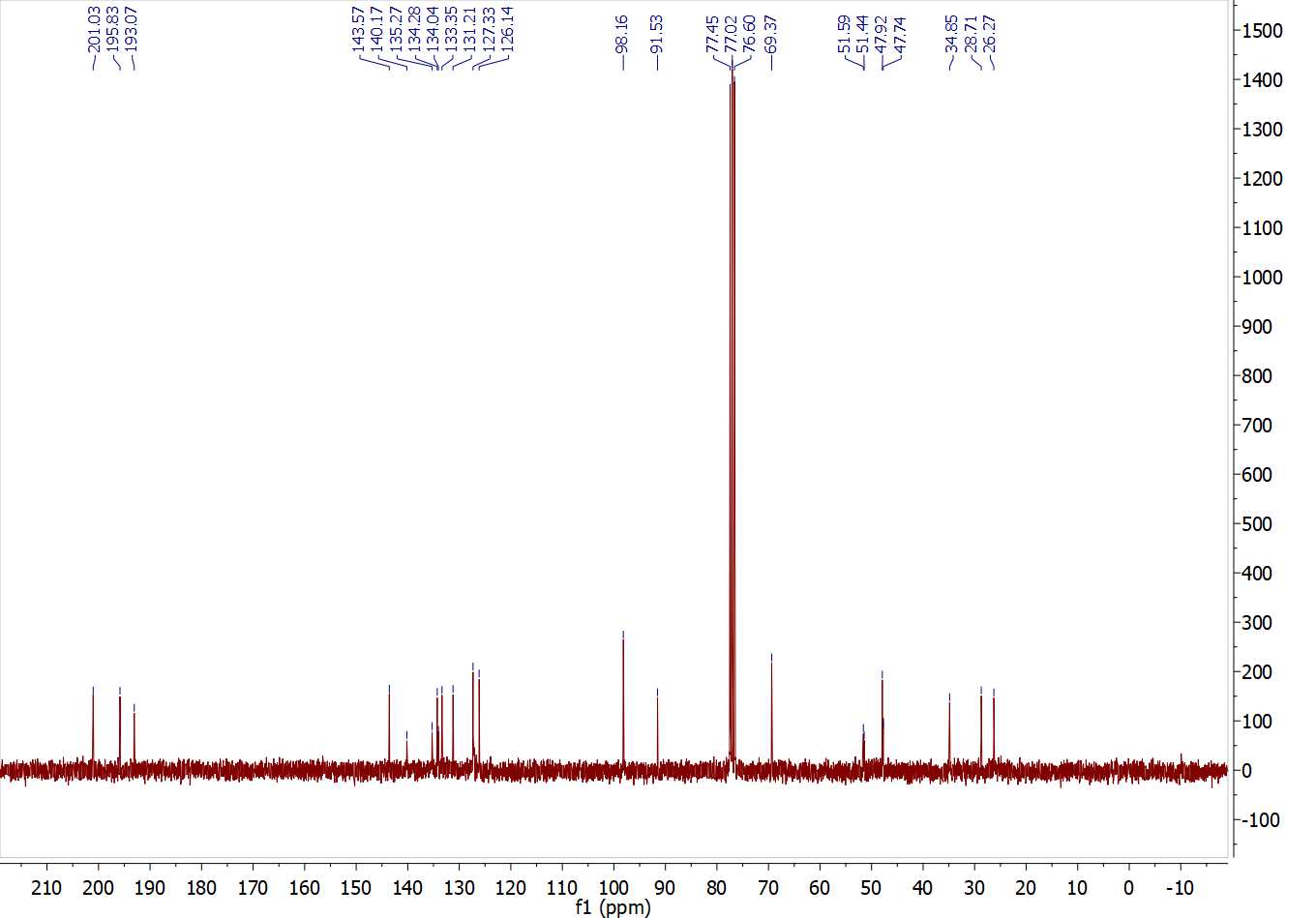


**Figure 86** ^13^C NMR (76 MHz, CDCl_3_) **10** Acetal.


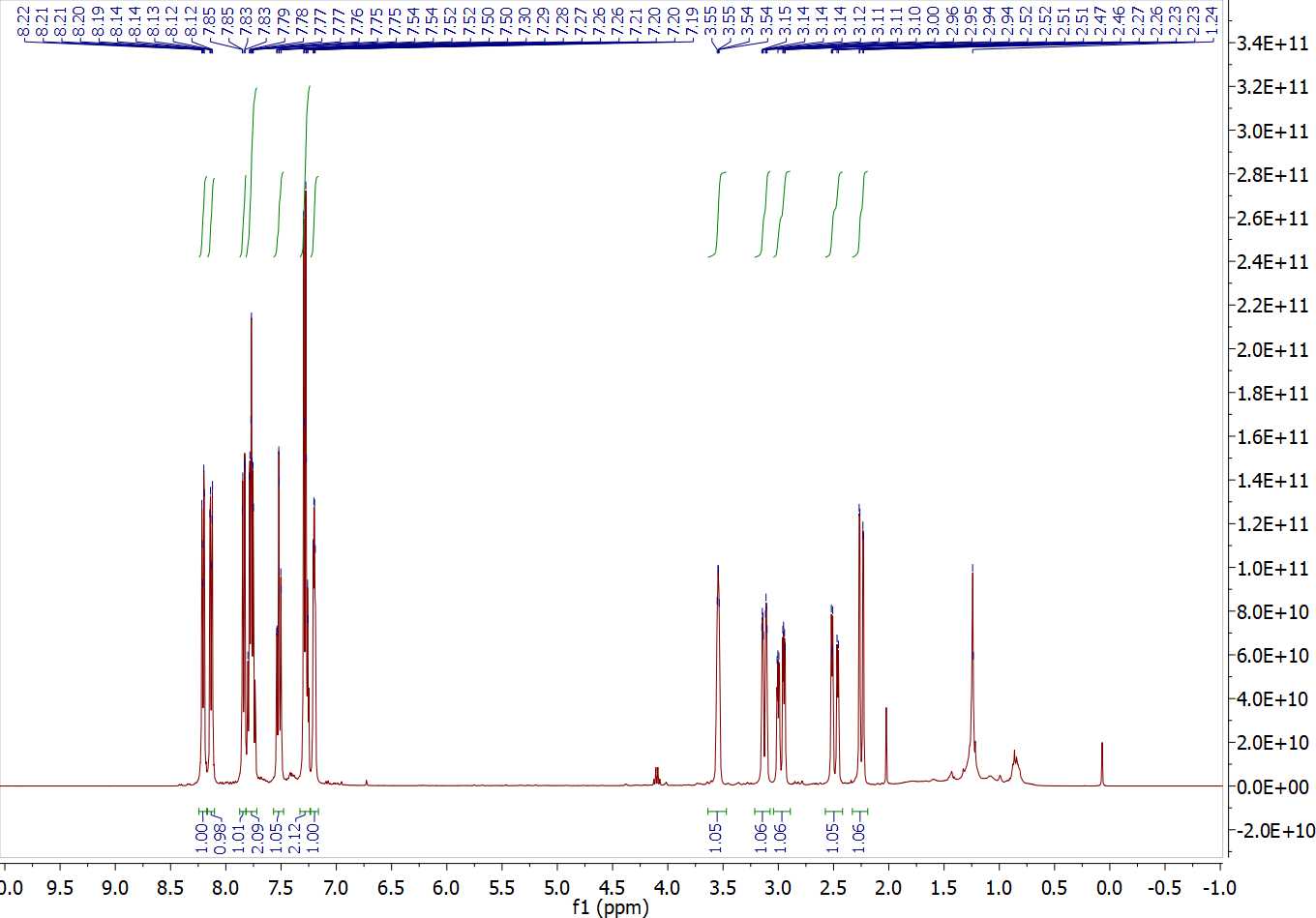


**Figure 87** ^1^H NMR (400 MHz, CDCl_3_) **12** Heck product.


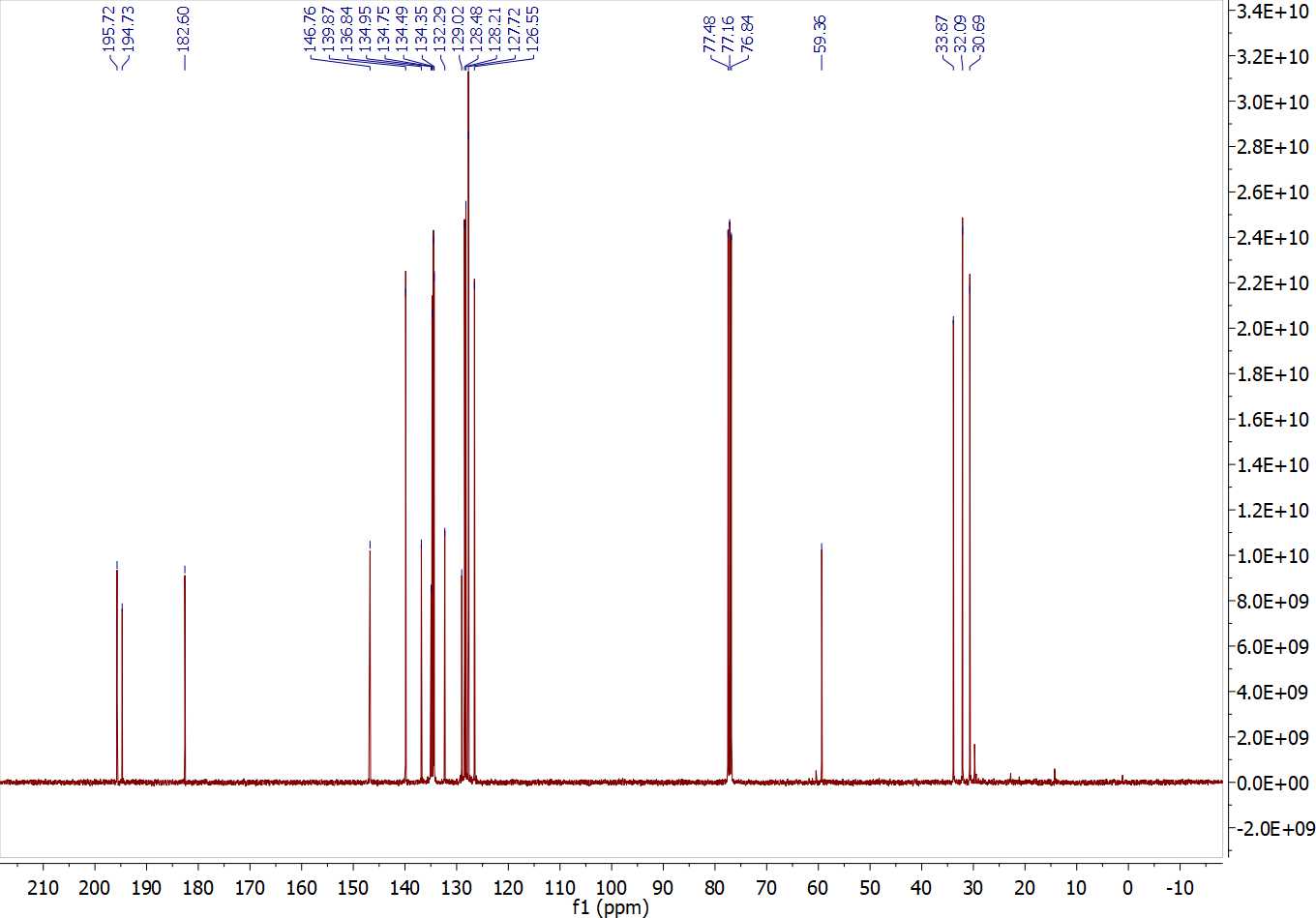


**Figure 88** ^13^C NMR (101 MHz, CDCl_3_) **12** Heck product.

## Crystallographic Information

### Crystallographic Data for Compounds 4b, 5a, 8aa, 8ae, 8af, 8bh, 9ad and 9af

The single-crystal X-ray diffraction study were carried out on a Bruker D8 Venture diffractometer with Photon100 detector (**8aa**, **9af**) or PhotonII detector (**4b**, **5a**, **9ad**, **8ae**, **8af**, **8bh**) at 123(2) K using Mo-Kα radiation (*λ* = 0.71073 Å) (**4b**, **8aa**, **5a**, **8ae**, **8bh**) or Cu-Kα radiation (*λ* = 1.54178 Å) (**9ad**, **9af**, **8af**). Direct Methods (SHELXS-97 for **8aa**, **9af**) [G. M. Sheldrick, *Acta Crystallogr.* 2008, **A64**, 112–122] or dual space methods (SHELXT for **4b**, **5a**, **9ad**, **8ae**, **8af**, **8bh**) [G. M. Sheldrick, *Acta Crystallogr.* 2015, **A71**, 3–8] were used for structure solution and refinement was carried out using SHELXL-2014 (full-matrix least-squares on *F^2^*) [G. M. Sheldrick, *Acta Crystallogr.* 2015, **C71**, 3–8]. Hydrogen atoms were localized by difference electron density determination and refined using a riding model. Semi-empirical absorption corrections were applied. For **8aa**, **8ae** and **9af** an extinction correction was applied. The absolute structure of **4b** was determined by the refinement of Parsons’ x-parameter [Parson, Flack, Wagner, *Acta Crystallogr.* 2013, **B69**, 249–259].

**4b** – **sb1081_hy**: colourless crystals, C_19_H_15_BrO_3_, *M*_r_ = 371.22, crystal size 0.18 × 0.09 × 0.03 mm, monoclinic, space group *P*c (No. 9), *a* = 18.5274(9) Å, *b* = 7.6936(3) Å, *c* = 10.7680(5) Å, *β* = 98.558(2)°, *V* = 1517.81(12) Å^3^, *Z* = 4, *ρ* = 1.625 Mg/m^-3^, *µ*(Mo-K_α_) = 2.72 mm^-1^, *F*(000) = 752, *2θ*_max_ = 55.0°, 54899 reflections, of which 6945 were independent (*R*_int_ = 0.038), 419 parameters, 2 restraints, *R*_1_ = 0.020 (for 6665 I > 2σ(I)), w*R*_2_ = 0.043 (all data), *S* = 1.04, largest diff. peak / hole = 0.24 / -0.19 e Å^-3^, x = -0.005(2).

**8aa** – **sb932_hy**: colourless crystals, C_21_H_21_IO_3_, *M*_r_ = 442.23, crystal size 0.24 × 0.18 × 0.06 mm, monoclinic, space group *P*2_1_/c (No. 14), *a* = 15.3153(7) Å, *b* = 8.2650(4) Å, *c* = 15.5386(8) Å, *β* = 118.249(2)°, *V* = 1732.63(15) Å^3^, *Z* = 4, *ρ* = 1.695 Mg/m^-3^, *µ*(Mo-K_α_) = 1.87 mm^-1^, *F*(000) = 872, *2θ*_max_ = 55.2°, 41293 reflections, of which 3998 were independent (*R*_int_ = 0.026), 227 parameters, *R*_1_ = 0.015 (for 3638 I > 2σ(I)), w*R*_2_ = 0.036 (all data), *S* = 1.06, largest diff. peak / hole = 0.43 / -0.30 e Å^-3^.

**5a** – **sb1080_hy**: yellow crystals, C_17_H_9_IO_3_, *M*_r_ = 388.14, crystal size 0.14 × 0.08 × 0.04 mm, monoclinic, space group *P*2_1_/c (No. 14), *a* = 5.5188(3) Å, *b* = 10.5469(6) Å, *c* = 23.2612(13) Å, *β* = 90.746(2)°, *V* = 1353.83(13) Å^3^, *Z* = 4, *ρ* = 1.904 Mg/m^-3^, *µ*(Mo-K_α_) = 2.37 mm^-1^, *F*(000) = 752, *2θ*_max_ = 55.0°, 34641 reflections, of which 3104 were independent (*R*_int_ = 0.043), 151 parameters, 21 restraints, *R*_1_ = 0.045 (for 2834 I > 2σ(I)), w*R*_2_ = 0.096 (all data), *S* = 1.16, largest diff. peak / hole = 1.29 / -1.55 e Å^-3^, the 2‑iodobenzoyl moiety is disordered (see cif-file for details).

**9ad** – **sb1077_hy**: colourless crystals, C_24_H_23_IO_4_Si, *M*_r_ = 530.41, crystal size 0.06 × 0.04 × 0.02 mm, monoclinic, space group *P*2_1_/c (No. 14), *a* = 17.1926(4) Å, *b* = 9.0111(2) Å, *c* = 15.3294(3) Å, *β* = 107.781(1)°, *V* = 2261.45(9) Å^3^, *Z* = 4, *ρ* = 1.558 Mg/m^-3^, *µ*(Cu-K_α_) = 11.86 mm^-1^, *F*(000) = 1064, *2θ*_max_ = 144.4°, 23892 reflections, of which 4439 were independent (*R*_int_ = 0.035), 271 parameters, *R*_1_ = 0.031 (for 3930 I > 2σ(I)), w*R*_2_ = 0.069 (all data), *S* = 1.03, largest diff. peak / hole = 1.47 / -1.05 e Å^-3^.

**8ae** – **sb1079_hy**: colourless crystals, C_25_H_25_IO_4_Si, *M*_r_ = 544.44, crystal size 0.20 × 0.16 × 0.10 mm, monoclinic, space group *P*2_1_/c (No. 14), *a* = 10.4516(4) Å, *b* = 12.0051(4) Å, *c* = 18.9173(7) Å, *β* = 101.819(1)°, *V* = 2323.28(15) Å^3^, *Z* = 4, *ρ* = 1.557 Mg/m^-3^, *µ*(Mo-K_α_) = 1.46 mm^-1^, *F*(000) = 1096, *2θ*_max_ = 55.0°, 56591 reflections, of which 5346 were independent (*R*_int_ = 0.026), 282 parameters, *R*_1_ = 0.017 (for 4996 I > 2σ(I)), w*R*_2_ = 0.043 (all data), *S* = 1.08, largest diff. peak / hole = 0.43 / -0.33 e Å^-3^.

**9af** – **sb898_hy**: colourless crystals, C_28_H_31_I_2_O_4_Si, *M*_r_ = 586.52, crystal size 0.16 × 0.12 × 0.08 mm, monoclinic, space group *P*2_1_/n (No. 14), *a* = 12.4501(8) Å, *b* = 9.4569(6) Å, *c* = 22.4286(14) Å, *β* = 101.113(2)°, *V* = 2591.2(3) Å^3^, *Z* = 4, *ρ* = 1.503 Mg/m^-3^, *µ*(Cu-K_α_) = 10.41 mm^-1^, *F*(000) = 1192, *2θ*_max_ = 144.4°, 28040 reflections, of which 5087 were independent (*R*_int_ = 0.024), 309 parameters, *R*_1_ = 0.021 (for 4969 I > 2σ(I)), w*R*_2_ = 0.052 (all data), *S* = 1.05, largest diff. peak / hole = 0.67 / -0.54 e Å^-3^.

**8af** – **sb1164_hy**: colourless crystals, C_28_H_31_IO_4_Si, *M*_r_ = 586.52, crystal size 0.12 × 0.03 × 0.01 mm, monoclinic, space group *P*2_1_/c (No. 14), *a* = 19.1112(6) Å, *b* = 7.9277(3) Å, *c* = 35.7482(11) Å, *β* = 101.728(2)°, *V* = 5303.1(3) Å^3^, *Z* = 8, *ρ* = 1.469 Mg/m^-3^, *µ*(Cu-K_α_) = 10.17 mm^-1^, *F*(000) = 2384, *2θ*_max_ = 144.6°, 59783 reflections, of which 10398 were independent (*R*_int_ = 0.131), 615 parameters, *R*_1_ = 0.081 (for 7491 I > 2σ(I)), w*R*_2_ = 0.173 (all data), *S* = 1.06, largest diff. peak / hole = 1.84 / -1.04 e Å^-3^.

**8bh** – **sb1078_hy**: yellow crystals, C_37_H_33_Br_2_O_4_Si, *M*_r_ = 649.63, crystal size 0.40 × 0.30 × 0.20 mm, monoclinic, space group *P*2_1_/c (No. 14), *a* = 19.7258(5) Å, *b* = 9.8370(3) Å, *c* = 16.3586(4) Å, *β* = 106.796(1)°, *V* = 3038.85(14) Å^3^, *Z* = 4, *ρ* = 1.420 Mg/m^-3^, *µ*(Mo-K_α_) = 1.43 mm^-1^, *F*(000) = 1344, *2θ*_max_ = 55.0°, 80591 reflections, of which 6966 were independent (*R*_int_ = 0.032), 388 parameters, *R*_1_ = 0.025 (for 6448 I > 2σ(I)), w*R*_2_ = 0.063 (all data), *S* = 1.05, largest diff. peak / hole = 0.34 / -0.49 e Å^-3^.

CCDC 1992178 (**4b**), 1992179 (**8aa**), 1992180 (**5a**), 1992181 (**9ad**), 1992182 (**8ae**), 1992183 (**9af**), 1992184 (**8af**), and 1992185 (**8bh**) contain the supplementary crystallographic data for this paper. These data can be obtained free of charge from The Cambridge Crystallographic Data Centre *via* [www.ccdc.cam.ac.uk/data_request/cif](http://www.ccdc.cam.ac.uk/data_request/cif).

a) SHELXS: G. M. Sheldrick, *Acta Crystallogr.* 2008, **A64**, 112–122; doi.org/10.1107/S0108767307043930.

b) SHELXT G. M. Sheldrick, *Acta Crystallogr.* 2015, **A71**, 3–8; doi.org/10.1107/S2053273314026370.

c) SHELXL-2014 and more recent: G. M. Sheldrick, *Acta Crystallogr.* 2015, **C71**, 3–8; doi.org/10.1107/S2053229614024218.

d) S. Parson, H. D. Flack, T. Wagner, *Acta Crystallogr.* 2013, **B69**, 249–259; doi.org/10.1107/S2052519213010014.





Fig. 1x. Molecular structure of **4b** (both crystallographic independent molecules shown, displacement parameters are drawn at 50 % probability level).





Fig. 2x. Molecular structure of **4b** (1^st^ crystallographic independent molecule shown, displacement parameters are drawn at 50 % probability level).





Fig. 3x. Molecular structure of **4b** (2^nd^ crystallographic independent molecule shown, displacement parameters are drawn at 50 % probability level).





Fig. 4x. Molecular structure of **8aa** (displacement parameters are drawn at 50 % probability level).





Fig. 5x. Molecular structure of **5a** (disorder is shown, displacement parameters are drawn at 50 % probability level).





Fig.6x. Molecular structure of **5a** (minor disordered parts omitted for clarity, displacement parameters are drawn at 50 % probability level).





Fig. 7x. Molecular structure of **sb1077_hy** (displacement parameters are drawn at 50 % probability level).





Fig. 8x. Molecular structure of **8ae** (displacement parameters are drawn at 50 % probability level).





Fig. 9x. Molecular structure of **9af** (displacement parameters are drawn at 50 % probability level).





Fig. 10x. Molecular structure of **8af** (both crystallographic independent molecules shown, displacement parameters are drawn at 50 % probability level).





Fig. 11x. Molecular structure of **8af** (1^st^ crystallographic independent molecule shown, displacement parameters are drawn at 50 % probability level).





Fig. 12x. Molecular structure of **8af** (2^nd^ crystallographic independent molecule shown, displacement parameters are drawn at 50 % probability level).

Fig. 13x. Molecular structure of **8bh** (displacement parameters are drawn at 50 % probability level).

### 4.2 Crystallographic Data for Compounds 4a, 4c–f, 5b, 5d, 8ac, 8ad, 9ae, 8ag, 8ba, 8bc, 8bd, 9bd, 9bf, 8fc, 8gc, 9gd and 12

Crystallographic data for compounds **4a**, **4c**–**f**, **5b**, **5d**, **8ac**, **8ad**, **9ae**, **8ag**, **8ba**, **8bc**, **8bd**, **9bd**, **9bf**, **8fc**, **8gc**, **9gd** and **12** reported in this paper have been deposited with the Cambridge Crystallographic Data Centre as supplementary information no. CCDC-1992874–1992893. Copies of the data can be obtained free of charge from <https://www.ccdc.cam.ac.uk/structures/>.

**Experimental Details**

Single crystal X-ray diffraction data of compounds compounds **4a**, **4c**–**f**, **5b**, **5d**, **8ac**, **8ad**, **9ae**, **8ag**, **8ba**, **8bc**, **8bd**, **9bd**, **9bf**, **8fc**, **8gc**, **9gd** and **12** were collected on a STOE STADI VARI diffractometer with monochromated Ga Kα (λ = 1.34143 Å), Cu Kα (λ = 1.54186 Å) or Mo Kα (λ = 0.71073) radiation at low temperature. Using Olex2 [1], the structures were solved with the ShelXT [2] structure solution program using Intrinsic Phasing and refined with the ShelXL [3] refinement package using Least Squares minimization. Refinement was performed with anisotropic temperature factors for all non-hydrogen atoms; hydrogen atoms were calculated on idealized positions. Crystal data and structure refinement details of compounds **4a**, **4c**–**f**, **5b**, **5d**, **8ac**, **8ad**, **9ae**, **8ag**, **8ba**, **8bc**, **8bd**, **9bd**, **9bf**, **8fc**, **8gc**, **9gd** and **12** are summarized in Table S1.

1. Dolomanov, O.V., Bourhis, L.J., Gildea, R.J, Howard, J.A.K. & Puschmann, H. (2009), J. Appl. Cryst. 42, 339–341.
2. Sheldrick, G.M. (2015). Acta Cryst. A71, 3–8.
3. Sheldrick, G.M. (2015). Acta Cryst. C71, 3–8.

Table S1: Crystal data and structure refinement details for compounds **4a**, **4c**–**f**, **5b**, **5d**, **8ac**, **8ad**, **9ae**, **8ag**, **8ba**, **8bc**, **8bd**, **9bd**, **9bf**, **8fc**, **8gc**, **9gd** and **12**.

| Name | **4c** | **5b** | **8ba** | **8ac** | **4a** |
| --- | --- | --- | --- | --- | --- |
| Identification code | JB234 | JB205-1 | JB249 | JB246 | QB093_tw |
| Empirical formula | C_20_H_17_BrO_4_ | C_17_H_9_O_3_Br | C_21_H_15_BrO_3_ | C_23_H_19_IO_3_ | C_19_H_15_IO_3_ |
| Formula weight | 401.24 | 341.15 | 395.24 | 470.28 | 418.21 |
| Temperature/K | 150 | 180.15 | 180.15 | 180.15 | 180.15 |
| Crystal system | monoclinic | monoclinic | monoclinic | monoclinic | monoclinic |
| Space group | *P*2_1_/*c* | *P*2_1_/*c* | *P*2_1_/*c* | *P*2_1_/*n* | *Pc* |
| a/Å | 8.0208(3) | 5.42720(10) | 14.4170(5) | 7.7683(5) | 18.4714(9) |
| b/Å | 10.7247(4) | 10.5371(4) | 7.8476(2) | 20.5514(14) | 8.0153(2) |
| c/Å | 20.3313(8) | 23.1383(6) | 15.3699(5) | 11.8395(9) | 10.8747(4) |
| α/° | 90 | 90 | 90 | 90 | 90 |
| β/° | 100.599(3) | 91.114(2) | 104.275(3) | 90.887(6) | 98.540(4) |
| γ/° | 90 | 90 | 90 | 90 | 90 |
| Volume/Å^3^ | 1719.07(11) | 1322.96(7) | 1685.24(9) | 1889.9(2) | 1592.19(11) |
| Z | 4 | 4 | 4 | 4 | 4 |
| ρ_calc_g/cm^3^ | 1.550 | 1.713 | 1.558 | 1.653 | 1.745 |
| μ/mm^‑1^ | 2.358 | 2.951 | 2.373 | 13.480 | 2.024 |
| F(000) | 816.0 | 680.0 | 800.0 | 936.0 | 824.0 |
| Radiation | GaKα (λ = 1.34143) | GaKα (λ = 1.34143) | GaKα (λ = 1.34143) | CuKα (λ = 1.54186) | MoKα (λ = 0.71073) |
| 2Θ range for data collection/° | 9.76 – 124.0 | 12.37 – 118.8 | 10.33 – 108.0 | 13.54 – 141.1 | 4.46 – 54.0 |
| Reflections collected | 10370 | 6867 | 6226 | 7433 | 5516 |
| Independent reflections | 4015 [R_int_ = 0.0170,  R_σ_ = 0.0139] | 2723 [R_int_ = 0.0214,  R_σ_ = 0.0161] | 2951 [R_int_ = 0.0171,  R_σ_ = 0.0131] | 3486 [R_int_ = 0.0704,  R_σ_ = 0.0571] | 5516 [R_int_ = merged,  R_σ_ = 0.0160] |
| Indep. Refl. with I ≥ 2σ (I) | 3681 | 2574 | 2778 | 3058 | 5347 |
| Data/restraints/parameters | 4015/0/295 | 2723/0/190 | 2951/0/226 | 3486/0/246 | 5516/2/420 |
| Goodness-of-fit on F^2^ | 1.098 | 1.106 | 1.034 | 1.466 | 1.032 |
| Final R indexes [I ≥ 2σ (I)] | R_1_ = 0.0272, wR_2_ = 0.0757 | R_1_ = 0.0454, wR_2_ = 0.1207 | R_1_ = 0.0328, wR_2_ = 0.0886 | R_1_ = 0.1294, wR_2_ = 0.3134 | R_1_ = 0.0275, wR_2_ = 0.0732 |
| Final R indexes [all data] | R_1_ = 0.0296, wR_2_ = 0.0773 | R_1_ = 0.0471, wR_2_ = 0.1220 | R_1_ = 0.0344, wR_2_ = 0.0897 | R_1_ = 0.1341, wR_2_ = 0.3224 | R_1_ = 0.0298, wR_2_ = 0.0768 |
| Largest diff. peak/hole / e Å^-3^ | 0.39/–0.47 | 1.43/–0.74 | 0.84/–1.09 | 6.39/–4.22 | 0.58/–0.70 |
| Flack parameter |  |  |  |  | 0.05(2) |
| CCDC number | 1992874 | 1992875 | 1992876 | 1992877 | 1992878 |

Table S1 (continued)

| Name | **4d** | **4e** | **4f** | **5d** | **8bc** |
| --- | --- | --- | --- | --- | --- |
| Identification code | JB265 | JB263-F3 | JB244 | JB254 | JB366 |
| Empirical formula | C_21_H_19_BrO_5_ | C_21_H_20_BrNO_5_ | C_21_H_15_BrF_3_NO_4_ | C_19_H_13_BrO_5_ | C_23_H_19_O_3_Br |
| Formula weight | 431.27 | 446.29 | 482.25 | 431.27 | 423.29 |
| Temperature/K | 150 | 150.15 | 150.15 | 180.15 | 150 |
| Crystal system | monoclinic | monoclinic | triclinic | monoclinic | orthorhombic |
| Space group | P2_1_/c | *P*2_1_/*c* | *P*¯1 | *P*2_1_/*c* | *Pca*2_1_ |
| a/Å | 12.4751(4) | 18.5103(5) | 8.0894(2) | 12.2153(4) | 15.1575(5) |
| b/Å | 13.4745(3) | 7.30020(10) | 10.0616(3) | 10.1360(2) | 16.7865(7) |
| c/Å | 12.7582(4) | 14.5567(3) | 12.6050(3) | 14.2168(5) | 7.2918(2) |
| α/° | 90 | 90 | 97.484(2) | 90 | 90 |
| β/° | 117.121(2) | 91.803(2) | 101.001(2) | 112.292(2) | 90 |
| γ/° | 90 | 90 | 103.175(2) | 90 | 90 |
| Volume/Å^3^ | 1908.79(10) | 1966.06(7) | 964.25(5) | 1628.69(9) | 1855.34(11) |
| Z | 4 | 4 | 2 | 4 | 4 |
| ρ_calc_g/cm^3^ | 1.501 | 1.508 | 1.661 | 1.759 | 1.515 |
| μ/mm^‑1^ | 2.176 | 2.136 | 2.334 | 2.551 | 2.180 |
| F(000) | 880.0 | 912.0 | 484.0 | 880.0 | 864.0 |
| Radiation | GaKα (λ = 1.34143) | GaKα (λ = 1.34143) | GaKα (λ = 1.34143) | GaKα (λ = 1.34143) | GaKα (λ = 1.34143) |
| 2Θ range for data collection/° | 8.86 – 125.0 | 4.16 – 115.0 | 6.32 – 115.0 | 6.80 – 118.4 | 6.84 – 125.0 |
| Reflections collected | 13459 | 15260 | 13918 | 7926 | 11374 |
| Independent reflections | 4551 [R_int_ = 0.0136,  R_σ_ = 0.0113] | 3978 [R_int_ = 0.0171,  R_σ_ = 0.0096] | 3925 [R_int_ = 0.0147,  R_σ_ = 0.0084] | 3418 [R_int_ = 0.0215,  R_σ_ = 0.0154] | 3836 [R_int_ = 0.0128,  R_σ_ = 0.0097] |
| Indep. Refl. with I ≥ 2σ (I) | 4196 | 3896 | 3852 | 3279 | 3768 |
| Data/restraints/parameters | 4551/0/248 | 3978/0/333 | 3925/0/331 | 3418/0/228 | 3836/1/247 |
| Goodness-of-fit on F^2^ | 1.074 | 1.066 | 1.051 | 1.085 | 1.054 |
| Final R indexes [I ≥ 2σ (I)] | R_1_ = 0.0293, wR_2_ = 0.0768 | R_1_ = 0.0256, wR_2_ = 0.0719 | R_1_ = 0.0234, wR_2_ = 0.0594 | R_1_ = 0.0406, wR_2_ = 0.1096 | R_1_ = 0.0260, wR_2_ = 0.0699 |
| Final R indexes [all data] | R_1_ = 0.0318, wR_2_ = 0.0794 | R_1_ = 0.0261, wR_2_ = 0.0722 | R_1_ = 0.0238, wR_2_ = 0.0597 | R_1_ = 0.0419, wR_2_ = 0.1108 | R_1_ = 0.0264, wR_2_ = 0.0702 |
| Largest diff. peak/hole / e Å^-3^ | 0.44/–0.55 | 0.36/–0.62 | 0.36/–0.43 | 0.44/–0.68 | 0.21/–0.58 |
| Flack parameter |  |  |  |  | 0.41(2) |
| CCDC number | 1992879 | 1992880 | 1992881 | 1992882 | 1992883 |

Table S1 (continued)

| Name | **8ad** | **9bd** | **8bd** | **9ae** | **9bf** |
| --- | --- | --- | --- | --- | --- |
| Identification code | JB252 F2.2.2 | JB211-F1 | JB243 F2.3 | JB228-F1 | JB369-F2 |
| Empirical formula | C_24_H_23_O_4_SiI | C_24_H_23_BrO_4_Si | C_24_H_23_BrO_4_Si | C_25_H_25_IO_4_Si | C_28_H_31_BrO_4_Si |
| Formula weight | 530.41 | 483.42 | 483.42 | 544.44 | 539.53 |
| Temperature/K | 180.15 | 180.15 | 150 | 180.15 | 150 |
| Crystal system | triclinic | monoclinic | triclinic | monoclinic | monoclinic |
| Space group | *P*¯1 | *P*2_1_/*n* | *P*¯1 | *P*2_1_/*n* | *P*2_1_/*n* |
| a/Å | 10.0925(3) | 13.2366(3) | 9.3741(5) | 13.5865(6) | 11.9246(2) |
| b/Å | 10.8789(3) | 8.60730(10) | 11.0638(6) | 8.6619(3) | 14.6618(2) |
| c/Å | 11.9319(3) | 19.3654(4) | 11.8758(6) | 19.7422(9) | 15.2528(3) |
| α/° | 116.360(2) | 90 | 69.145(4) | 90 | 90 |
| β/° | 102.313(2) | 91.976(2) | 74.144(4) | 95.241(4) | 104.8900(10) |
| γ/° | 93.973(2) | 90 | 85.795(4) | 90 | 90 |
| Volume/Å^3^ | 1126.55(6) | 2205.01(7) | 1106.80(11) | 2313.65(17) | 2577.19(8) |
| Z | 2 | 4 | 2 | 4 | 4 |
| ρ_calc_g/cm^3^ | 1.564 | 1.456 | 1.451 | 1.563 | 1.391 |
| μ/mm^‑1^ | 7.958 | 2.230 | 2.221 | 1.465 | 1.945 |
| F(000) | 532.0 | 992.0 | 496.0 | 1096.0 | 1120.0 |
| Radiation | GaKα (λ = 1.34143) | GaKα (λ = 1.34143) | GaKα (λ = 1.34143) | MoKα (λ = 0.71073) | GaKα (λ = 1.34143) |
| 2Θ range for data collection/° | 7.48 – 120.0 | 6.93 – 126.0 | 7.19 – 125.3 | 3.81 – 63.4 | 7.34 – 125.0 |
| Reflections collected | 12471 | 14639 | 13753 | 32349 | 37678 |
| Independent reflections | 4912 [R_int_ = 0.0113,  R_σ_ = 0.0086] | 5292 [R_int_ = 0.0156,  R_σ_ = 0.0116] | 5240 [R_int_ = 0.0147,  R_σ_ = 0.0138] | 7193 [R_int_ = 0.0275,  R_σ_ = 0.0237] | 6219 [R_int_ = 0.0136,  R_σ_ = 0.0082] |
| Indep. Refl. with I ≥ 2σ (I) | 4833 | 5163 | 4750 | 5542 | 5899 |
| Data/restraints/parameters | 4912/0/274 | 5292/0/274 | 5240/0/364 | 7193/0/380 | 6219/0/313 |
| Goodness-of-fit on F^2^ | 1.039 | 1.067 | 1.073 | 1.022 | 1.100 |
| Final R indexes [I ≥ 2σ (I)] | R_1_ = 0.0193, wR_2_ = 0.0505 | R_1_ = 0.0419, wR_2_ = 0.1077 | R_1_ = 0.0267, wR_2_ = 0.0728 | R_1_ = 0.0291, wR_2_ = 0.0653 | R_1_ = 0.0279, wR_2_ = 0.0741 |
| Final R indexes [all data] | R_1_ = 0.0196, wR_2_ = 0.0506 | R_1_ = 0.0426, wR_2_ = 0.1082 | R_1_ = 0.0298, wR_2_ = 0.0767 | R_1_ = 0.0450, wR_2_ = 0.0712 | R_1_ = 0.0290, wR_2_ = 0.0748 |
| Largest diff. peak/hole / e Å^-3^ | 0.82/–0.70 | 0.39–1.27 | 0.40/–0.46 | 0.96/–1.00 | 0.31/–0.52 |
| Flack parameter |  |  |  |  |  |
| CCDC number | 1992884 | 1992885 | 1992886 | 1992887 | 1992888 |

Table S1 (continued)

| Name | **8ag** | **8fc** | **8gc** | **9gd** | **12** |
| --- | --- | --- | --- | --- | --- |
| Identification code | JB201-F1 | JB274 | JB264 | JB280-F1 | JB375_C10 |
| Empirical formula | C_30_H_35_IO_4_Si | C_25_H_22_BrNO_4_ | C_25_H_19_NO_4_F_3_Br | C_26.33_H_23.33_BrClF_3_NO_5_Si | C_21_H_14_O_3_ |
| Formula weight | 614.57 | 480.34 | 534.32 | 634.24 | 314.32 |
| Temperature/K | 150.15 | 150 | 180.15 | 180.15 | 180.15 |
| Crystal system | monoclinic | hexagonal | monoclinic | trigonal | monoclinic |
| Space group | *P*2_1_/*n* | *P*6_5_ | *P*2_1_/*c* | *R*3*c* | *Pc* |
| a/Å | 9.0381(3) | 23.8314(3) | 19.8014(7) | 34.6858(6) | 15.8412(8) |
| b/Å | 11.1267(3) | 23.8314(3) | 13.6268(4) | 34.6858(6) | 7.9531(3) |
| c/Å | 27.9237(11) | 7.72040(10) | 9.0030(3) | 11.9046(2) | 11.9997(5) |
| α/° | 90 | 90 | 90 | 90 | 90 |
| β/° | 97.084(3) | 90 | 96.975(3) | 90 | 100.414(3) |
| γ/° | 90 | 120 | 90 | 120 | 90 |
| Volume/Å^3^ | 2786.69(16) | 3797.25(11) | 2411.30(14) | 12403.6(5) | 1486.90(11) |
| Z | 4 | 6 | 4 | 18 | 4 |
| ρ_calc_g/cm^3^ | 1.465 | 1.260 | 1.472 | 1.528 | 1.404 |
| μ/mm^‑1^ | 6.485 | 1.665 | 1.759 | 2.590 | 0.485 |
| F(000) | 1256.0 | 1476.0 | 1080.0 | 5784.0 | 656.0 |
| Radiation | GaKα (λ = 1.34143) | GaKα (λ = 1.34143) | MoKα (λ = 0.71073) | GaKα (λ = 1.34143) | GaKα (λ = 1.34143) |
| 2Θ range for data collection/° | 7.458 – 115.0 | 3.72 – 124.9 | 3.64 – 60.0 | 4.43–120.0 | 4.93–123.3 |
| Reflections collected | 20060 | 43479 | 31134 | 40108 | 17148 |
| Independent reflections | 5657 [R_int_ = 0.0275,  R_σ_ = 0.0174] | 4150 [R_int_ = 0.0203,  R_σ_ = 0.0114] | 7014 [R_int_ = 0.0204,  R_σ_ = 0.0190] | 4281 [R_int_ = 0.0306,  R_σ_ = 0.0176] | 5806 [R_int_ = 0.0663,  R_σ_ = 0.0539] |
| Indep. Refl. with I ≥ 2σ (I) | 5379 | 3941 | 5870 | 3975 | 3710 |
| Data/restraints/parameters | 5657/0/331 | 4150/1/283 | 7014/0/306 | 4281/2/341 | 5806/2/434 |
| Goodness-of-fit on F^2^ | 1.100 | 1.098 | 1.041 | 1.078 | 1.324 |
| Final R indexes [I ≥ 2σ (I)] | R_1_ = 0.0283, wR_2_ = 0.0812 | R_1_ = 0.0237, wR_2_ = 0.0677 | R_1_ = 0.0570, wR_2_ = 0.1523 | R_1_ = 0.0550, wR_2_ = 0.1540 | R_1_ = 0.1271, wR_2_ = 0.2919 |
| Final R indexes [all data] | R_1_ = 0.0301, wR_2_ = 0.0818 | R_1_ = 0.0251, wR_2_ = 0.0685 | R_1_ = 0.0673, wR_2_ = 0.1595 | R_1_ = 0.0585, wR_2_ = 0.1581 | R_1_ = 0.1642, wR_2_ = 0.3182 |
| Largest diff. peak/hole / e Å^-3^ | 1.46/–0.90 | 0.21/–0.29 | 1.81/–0.81 | 2.88/–1.61 | 1.07/–0.50 |
| Flack parameter |  | 0.015(15) |  | –0.02(3) | –0.9(10) |
| CCDC number | 1992889 | 1992890 | 1992891 | 1992892 | 1992893 |

Fig. 14x. Molecular structure of **4c** (displacement parameters are drawn at 50 % probability level).

Fig. 15x. Molecular structure of **5b** (displacement parameters are drawn at 50 % probability level).

Fig. 16x. Molecular structure of **8ba** (displacement parameters are drawn at 50 % probability level).

Fig. 17x. Molecular structure of **8ac** (displacement parameters are drawn at 50 % probability level).

Fig. 18x. Molecular structure of **4a** (both crystallographic independent molecules shown, displacement parameters are drawn at 50 % probability level).

Fig. 19x. Molecular structure of **4d** (displacement parameters are drawn at 50 % probability level).

Fig. 20x. Molecular structure of **4e** (including one lattice bound water molecule, displacement parameters are drawn at 50 % probability level).

Fig. 21x. Molecular structure of **4f** (displacement parameters are drawn at 50 % probability level).

Fig. 22x. Molecular structure of **5d** (displacement parameters are drawn at 50 % probability level).

Fig. 23x. Molecular structure of **8bc** (displacement parameters are drawn at 50 % probability level).

Fig. 24x. Molecular structure of **8ad** (displacement parameters are drawn at 50 % probability level).

Fig. 25x. Molecular structure of **9bd** (displacement parameters are drawn at 50 % probability level).

Fig. 26x. Molecular structure of **8bd** (displacement parameters are drawn at 50 % probability level).

Fig. 27x. Molecular structure of **9ae** (displacement parameters are drawn at 50 % probability level).

Fig. 28x. Molecular structure of **9bf** (displacement parameters are drawn at 50 % probability level).

Fig. 29x. Molecular structure of **8ag** (displacement parameters are drawn at 50 % probability level).

Fig. 30x. Molecular structure of **8fc** (displacement parameters are drawn at 50 % probability level).

Fig. 31x. Molecular structure of **8gc** (a disorder of CF_3_ group shown, displacement parameters are drawn at 50 % probability level).

Fig. 32x. Molecular structure of **9gd** (including one lattice bound chloroform molecule, displacement parameters are drawn at 50 % probability level).

Fig. 33x. Molecular structure of **12** (both crystallographic independent molecules are shown, displacement parameters are drawn at 50 % probability level).

1. M. Buccini and M. J. Piggott, *Organic Letters* **2014**, *16*, 2490–2493. [↑](#footnote-ref-1)
2. M. Buccini and M. J. Piggott, *Organic Letters* **2014**, *16*, 2490–2493. [↑](#footnote-ref-2)
